# Supplementary material for: Modular total syntheses of trans-clerodanes and sesquiterpene (hydro)quinones via tail-to-head cyclization and reductive coupling strategies
Source: Nat Commun. 2022 Nov 4;13:6633. doi: 10.1038/s41467-022-34404-4 (PMC9636166; doi:10.1038/s41467-022-34404-4)
Supplement: Supplementary file 2 — Supplementary Information [file 41467_2022_34404_MOESM2_ESM.pdf]

Supplementary Information

Modular total syntheses of *trans*-clerodanes and sesquiterpene (hydro)quinones via tail-to-head cyclization and reductive coupling strategies

Wenming Zhu[1,†], Qishuang Yin[1,†], Zhizheng Lou[1,] and Ming Yang[1,]*

[1]State Key Laboratory of Applied Organic Chemistry and College of Chemistry and Chemical Engineering, Lanzhou University, 222 South Tianshui Road, Lanzhou, Gansu Province 730000 (China)

[†]These authors contributed equally to this work.

*Email for correspondence: yangming@lzu.edu.cn

Table of Contents

I. Supplementary Methods S2

II. Supplementary Discussion S25

III. Supplementary References S122

I. Supplementary Methods

General Procedures. All reactions were carried out under an argon atmosphere with dry solvents under anhydrous conditions, unless otherwise noted. Tetrahydrofuran (THF), diethyl ether (Et_2O) and toluene were distilled immediately before use from sodium-benzophenone ketyl. Dimethylformamide (DMF) and dichloromethane (CH_2Cl_2) distilled from calcium hydride and stored under an argon atmosphere. Yields refer to chromatographically and spectroscopically (^1H and ^{13}C NMR) homogeneous materials, unless otherwise stated. Reagents were purchased at the highest commercial quality and used without further purification, unless otherwise stated. Reactions were magnetically stirred and monitored by thin-layer chromatography (TLC) carried out on 0.25 mm Xinnuo silica gel plates silica gel plates (60F-254) using UV light as visualizing agent, and an ethanolic solution of phosphomolybdic acid and cerium sulfate, and heat as developing agents. Steema silica gel (60, academic grade, particle size 0.040–0.063 mm) was used for flash column chromatography. Preparative thin-layer chromatography separations were carried out on 0.50 mm Xinuo silica gel plates (60F-254). NMR spectra were recorded on Bruker 600 MHz and 400 MHz instruments and calibrated using residual undeuterated solvent as an internal reference. The following abbreviations were used to explain the multiplicities: s = singlet, d = doublet, t = triplet, q = quartet, m = multiplet. IR spectra were recorded on a Perkin-Elmer 1000 series FT-IR spectrometer. High-resolution mass spectra (HRMS) were recorded on an Agilent 6244 Tof-MS using ESI (Electrospray Ionization) at the Analytical Testing Center of Lanzhou University.

Abbreviations. EtOAc = ethyl acetate, THF = tetrahydrofuran, MeCN = acetonitrile, DMF = *N*,*N*-dimethylformamide, DMSO = dimethyl sulfoxide, (*D*)-DET = Diethyl D-tartrate, (*L*)-DET = Diethyl L-tartrate, TBHP = butylhydroperoxid, TBSCl = *tert*-butylchlorodimethylsilane. Coll·HCl = 2,4,6-Collidine hydrogen chloride, NMO = 4-methylmorpholine N-oxide, TPAP = tetrapropylammonium perruthenate, NaHMDS = sodium bis(trimethylsilyl)amide, TBAF·$3H_2O$ = Tetrabutylammonium fluoride trihydrate, DIPEA = ethyldiisopropylamine, DMP = Dess–Martin periodinane, M.S. = molecular sieves, Co(Sal$^{t\text{-Bu}, t\text{-Bu}}$)Cl = (*R*,*R*)-*N*,*N'*-Bis(3,5-di-*tert*-butylsalicylidene)-1,2-cyclohexanediaminato cobalt(III) chloride, Salcomine, = *N*,*N'*-bis(salicylidene)ethylenediaminocobalt(II), HF·py = hydrogen fluoride pyridine, $NiBr_2$·DME = nickel bromide ethylene glycol dimethyl ether, *t*-BuOK = potassium tert-butoxide, *t*-BuONa = sodium tert-butoxide, PPh_3CH_3Br = methyltriphenylphosphonium bromide, *n*-BuSH = butanethiol, *n*-BuLi = *n*-butyllithium, HMPA = hexamethylphosphoramide, AcOH = acetic acid, *p*-TsOH·H_2O = *p*-toluenesulfonic acid monohydrate, NaH = sodium hydride, CSA = D-camphorsulfonic acid, $LiAlH_4$ = lithium aluminum hydride.

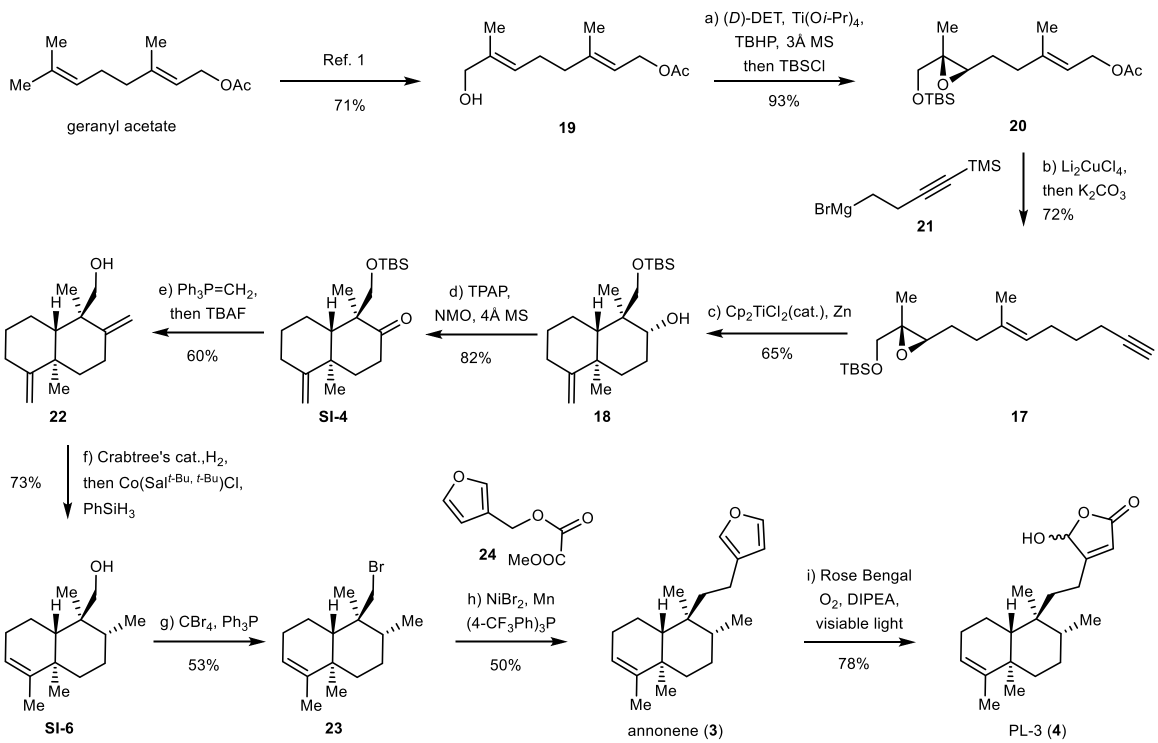

Supplementary Figure 1. Syntheses of annonene and PL3.

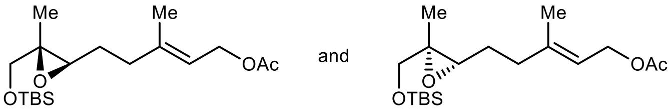

20 SI-1

Epoxides 20 and SI-1. To a stirred suspension of activated 3Å molecular sieves (5.60 g) in CH_2Cl_2 (100 mL) was added (*D*)-DET (0.777 g, 3.77 mmol, 0.1 equiv), Ti(O*i*-Pr)$_4$ (0.536 g, 1.88 mmol, 0.05 equiv) and TBHP (10.3 mL, 5.5 M in decane, 56.5 mmol, 1.5 equiv,) at -30 °C. The resultant mixture was stirred at -30 °C for 30 min before it was added a solution of alcohol **19**[1] (8.00 g, 37.7 mmol, 1.0 equiv) in CH_2Cl_2 (20 mL) dropwise at that temperature for 8 h. The reaction mixture was stirred at -30 °C for 2 h before it was quenched with triethanolamine (0.562 g, 3.77 mmol, 0.1 equiv) and stirred at 0 °C for 1 h. The resultant mixture was added imidazole (7.70 g, 113 mmol, 3.0 equiv) and TBSCl (11.4 g, 75.4 mmol, 2.0 equiv) at 0 °C before it was warmed to 23 °C and stirred at that temperature for 1 h. The reaction mixture was filtrated through Celite®. The filtrate was added water (200 mL) and extracted with CH_2Cl_2 (3 × 100 mL). The combined organic phases were washed with brine (150 mL), dried ($MgSO_4$), filtered, and concentrated. The resultant residue was purified by flash column chromatography (silica gel, EtOAc/petroleum ether, 1:30) to give epoxide **20** (12.0 g, 93% yield,) as a colorless oil. **20**: R_f = 0.50 (silica gel, EtOAc/petroleum ether, 1:10); $[a]_D^{25}$ = +7.1 (c = 0.5, CH_2Cl_2); IR (film) ν_{max} = 2957, 2929, 2858, 1743, 1597, 1472, 1463, 1382, 1366, 1249, 1232, 1097, 1023, 838, 778 cm^{-1}; ^{1}H NMR (400 MHz, $CDCl_3$) δ = 5.35 (t, *J* = 6.8 Hz, 1 H), 4.55 (d, *J* = 6.8 Hz, 2 H), 3.54 (s, 2 H), 2.81 (t, *J* = 5.7 Hz, 1 H), 2.23-2.08 (m, 2 H), 2.01 (s, 3 H), 1.69-1.63 (m, 5 H), 1.23 (s, 3 H) 0.86 (s, 9 H), 0.02 (s, 6 H) ppm; ^{13}C NMR (101 MHz, $CDCl_3$) δ = 171.1, 141.2, 119.0, 67.8, 61.3, 61.1, 60.4, 36.3, 26.7, 25.9, 21.1, 18.4, 16.6, 14.2, -5.3 ppm; HRMS (ESI) calcd for $C_{18}H_{35}O_4Si^+$ $[M + H]^+$ 343.2299, found 343.2297; **SI-1**: The Sharpless

epoxidation of alcohol **19** with (*L*)-DET delivered **SI-1** which is the enantiomer of **20**. The R_f, IR, ^{1}H and ^{13}C NMR, and HRMS of **SI-1** are the same to **20**; $[a]^{25}_{D}$ = −7.1 (c = 0.5, CH_2Cl_2).

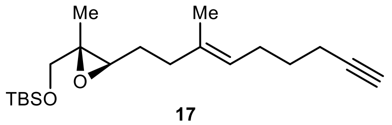

17

and

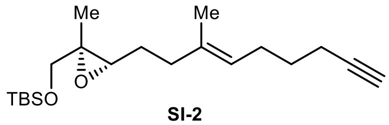

SI-2

Alkynes 17 and SI-2. To a solution of epoxide **20** (6.85 g, 20.0 mmol, 1.0 equiv) in THF (20 mL) was added Li_2CuCl_4 (20.0 mL, 0.1 M in THF, 2.00 mmol, 0.1 equiv) at 0 °C before a solution of Grignard reagent **21**[2] (37.5 mL, 0.8 M in THF, 30.0 mmol, 1.5 equiv) was added dropwise over 10 min at 0 °C. The resultant reaction was stirred at that temperature for 1 h before it was quenched with MeOH (125 mL). The resultant mixture was added K_2CO_3 (27.6 g, 200 mmol, 10.0 equiv) at 23 °C and stirred at that temperature for 12 h. The reaction contents were added saturated aqueous NH_4Cl (200 mL), filtrated, and extracted with EtOAc (4 × 200 mL). The combined organic phases were washed with brine (200 mL), dried ($MgSO_4$), filtered, and concentrated. The resultant residue was purified by flash column chromatography (silica gel, EtOAc/petroleum ether, 1:50) to give alkyne **17** (4.85 g, 72% yield,) as a colorless oil. **17**: R_f = 0.60 (silica gel, EtOAc/petroleum ether, 1:20); $[a]^{25}_{D}$ = +2.7 (c = 0.5, $CHCl_3$); IR (film) ν_{max} = 3315, 2956, 2928, 2857, 1599, 1463, 1379, 1362, 1252, 1132, 1097, 1007, 838, 778, 629 cm^{-1}; ^{1}H NMR (600 MHz, $CDCl_3$) δ = 5.14 (t, *J* = 7.1 Hz, 1 H), 3.56 (s, 2 H), 2.83 (t, *J* = 6.2 Hz, 1 H), 2.18-2.14 (m, 3 H), 2.12-2.07 (m, 3 H), 1.93 (t, *J* = 2.6 Hz, 1 H), 1.68-1.62 (m, 5 H), 1.58-1.53 (m, 2 H), 1.26 (s, 3 H), 0.88 (s, 9 H), 0.05 (s, 3 H), 0.04 (s, 3 H) ppm; ^{13}C NMR (101 MHz, $CDCl_3$) δ = 135.3, 124.1, 84.7, 68.4, 68.0, 61.1, 60.7, 36.5, 28.6, 27.0, 26.0, 18.5, 18.0, 16.1, 14.3, -5.2 ppm; HRMS (ESI) calcd for $C_{20}H_{37}O_2Si^+$ $[M + H]^+$ 337.2557, found 337.2555; **SI-2**: **SI-2** is the enantiomer of **17**, the R_f, IR, ^{1}H and ^{13}C NMR, and HRMS of **SI-2** are the same to **17**; $[a]^{25}_{D}$ = −1.2 (c = 0.5, $CHCl_3$).

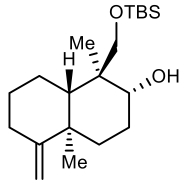

18

and

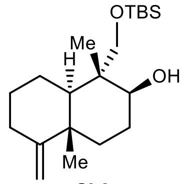

SI-3

Alcohols 18 and SI-3. To a stirred suspension of Cp_2TiCl_2 (1.85 g, 7.43 mmol, 0.2 equiv) in THF (600 mL) was added activated zinc powder (6.07 g, 92.8 mmol, 2.5 equiv) and 2,4,6-collidine·HCl (14.6 g, 92.8 mmol, 2.5 equiv) at 23 °C. The resultant mixture was stirred at that temperature for 10 min before a solution of alkyne **17** (12.5 g, 37.1 mmol, 1.0 equiv) in THF (20 mL) was added at 23 °C. The resultant reaction was warmed to 40 °C and stirred at that temperature for 3 h before it was poured into 1 M HCl (200 mL) at 0 °C. The resultant mixture was extracted with EtOAc (3 × 150 mL). The combined organic phases were washed with saturated aqueous $NaHCO_3$ (200 mL) and brine (200 mL), dried ($MgSO_4$), filtered, and concentrated. The resultant residue was purified by flash column chromatography (silica gel, EtOAc/petroleum ether, 1:30) to give alcohol **18** (8.17 g, 65% yield) as a colorless oil. **18**: R_f = 0.50 (silica gel, EtOAc/petroleum ether, 1:10); $[a]^{25}_{D}$ = +45.3 (c = 0.5, $CHCl_3$); IR (film) ν_{max} = 3454, 2930, 2858, 1636, 1472, 1464, 1447, 1387, 1378, 1362, 1252, 1130, 1094, 1074, 1050, 1021, 1005, 892, 872, 837, 776; ^{1}H NMR (600 MHz, $CDCl_3$) δ = 4.49 (d, *J* = 1.5 Hz, 2 H), 3.73 (s, 1 H), 3.63 (d, *J* = 9.3 Hz, 1 H), 3.58 (dd, *J* = 11.4, 4.6 Hz, 1 H), 3.31 (d, *J* = 9.3 Hz, 1 H), 2.27 (td, *J* = 13.7, 5.1 Hz, 1 H), 2.10-2.07 (m, 1 H), 1.86-1.82 (m, 1 H), 1.75-1.71 (m, 1 H), 1.69-1.60 (m, 2 H), 1.56 (dd, *J* = 13.3, 3.6 Hz, 1 H), 1.48 (td, *J* = 12.9, 3.8 Hz, 1 H), 1.40-1.37 (m, 1 H), 1.26-1.19 (m, 1 H), 1.06 (s, 3 H), 0.99 (dd, *J* = 12.4,

2.8 Hz, 1 H), 0.91 (s, 3 H), 0.88 (s, 9 H), 0.04 (s, 6 H) ppm; ^{13}C NMR (151 MHz, $CDCl_3$) δ = 159.3, 103.3, 77.1, 73.8, 48.2, 42.3, 39.6, 35.1, 33.0, 28.5, 26.6, 25.9, 22.4, 20.6, 18.2, 11.6, -5.6, -5.6 ppm; HRMS (ESI) calcd for $C_{20}H_{39}O_2Si^+$ $[M + H]^+$ 339.2714, found 339.2711; **SI-3**: **SI-3** is the enantiomer of **18**, the R_f, IR, ^{1}H and ^{13}C NMR, and HRMS of **SI-3** are the same to **18**; $[a]_D^{25}$ = −30.0 (c = 0.5, $CHCl_3$).

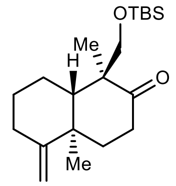

SI-4

and

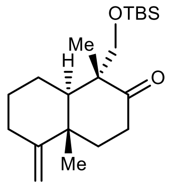

30

Ketones SI-4 and 30. To a stirred suspension of activated 4Å molecular sieves (10.0 g,) in CH_2Cl_2 (297 mL) was added alcohol **18** (10.0 g 29.5 mmol, 1.0 equiv) and NMO (6.92 g, 59.1 mmol, 2.0 equiv) at 0 °C. The resultant mixture was stirred at 0 °C for 10 min before TPAP (0.727 g, 2.07 mmol, 0.07 equiv) was added. The resultant reaction was stirred at 0 °C for 1 h before it was filtered. The filtrate was concentrated. The resultant residue was purified by flash column chromatography (silica gel, EtOAc/petroleum ether, 1:50) to give ketone **SI-4** (8.15 g, 82% yield) as a colorless oil. **SI-4**: R_f = 0.7 (silica gel, EtOAc/petroleum ether, 1:10); $[a]_D^{25}$ = +31.1 (c = 0.5, $CHCl_3$); IR (film) ν_{max} = 2930, 2858, 1738, 1707, 1637, 1598, 1464, 1384, 1362, 1252, 1193, 1124, 1097, 1007, 893, 838, 777 cm^{-1}; ^{1}H NMR (400 MHz, $CDCl_3$) δ = 4.63 (s, 1 H), 4.59 (s, 1 H), 3.72 (d, J = 9.2 Hz, 1 H), 3.20 (d, J = 9.2 Hz, 1 H), 2.54 (ddd, J = 18.1, 11.6, 8.1 Hz, 1 H), 2.43 (ddd, J = 18.1, 5.9, 3.2 Hz, 1 H), 2.33 (td, J = 13.5, 4.8 Hz, 1 H), 2.18 (dt, J = 13.5, 3.2 Hz, 1 H), 2.11 (dd, J = 9.1, 6.7 Hz, 1 H), 1.92-1.87 (m, 3 H), 1.53-1.47 (m, 2 H), 1.34-1.22 (m, 1 H), 1.13 (s, 3 H), 0.89 (s, 3 H), 0.82 (s, 9 H), -0.02 (s, 6 H) ppm; ^{13}C NMR (101 MHz, $CDCl_3$) δ = 215.9, 157.8, 104.8, 67.7, 52.7, 45.4, 38.9, 36.1, 33.9, 33.3, 28.4, 25.9, 23.4, 19.3, 18.2, 18.1, -5.5, -5.6 ppm; HRMS (ESI) calcd for $C_{20}H_{37}O_2Si$ $[M + H]^+$ 337.2557, found 337.2557; **30**: **30** is the enantiomer of **SI-4**, the R_f, IR, ^{1}H and ^{13}C NMR, and HRMS of **30** are the same to **SI-4**; $[a]_D^{25}$ = −24.9 (c = 0.5, $CHCl_3$).

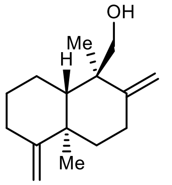

22

and

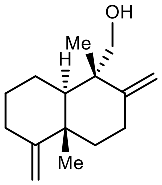

SI-5

Alcohols 22 and SI-5. To A solution of PPh_3CH_3Br (16.7 g, 46.8 mmol, 1.75 equiv) in THF (240 mL) was added NaHMDS (20.1 mL, 2.0 M in THF, 40.2 mmol, 1.5 equiv) dropwise at 0 °C. The resultant mixture was stirred at that temperature for 30 min before a solution of ketone **SI-4** (9.00 g, 26.7 mol, 1.0 equiv) in THF (20 mL) was added. The resulting mixture was warmed to 60 °C and stirred at that temperature for 15 h before it was cooled to 23 °C and added TBAF·$3H_2O$ (42.2 g, 134 mmol, 5.0 equiv). The resultant mixture was warmed to 60 °C and stirred for 4 h. The reaction mixture was quenched with saturated aqueous NH_4Cl (250 mL) and extracted with EtOAc (3 × 100 mL). The combined organic phases were washed with brine (200 mL), dried ($MgSO_4$), filtered, and concentrated. The resultant residue was purified by flash column chromatography (silica gel, EtOAc/petroleum ether, 1:25) to give alcohol **22.** (3.54 g, 60% yield) as a white solid. **22**: R_f = 0.40 (silica gel, EtOAc/petroleum ether, 1:10); $[a]_D^{25}$ = +85.7 (c = 0.5, $CHCl_3$); IR (film) ν_{max} = 3441, 3083, 2978, 2954, 2936, 2905, 2862, 1635, 1599, 1489, 1470, 1457, 1443, 1377, 1303, 1260, 1191, 1140, 1069, 1051, 1035, 996, 889, 872 cm^{-1}; ^{1}H NMR (400 MHz,

$CDCl_3$) δ = 4.85 (s, 1 H), 4.72 (s, 1 H), 4.51 (d, *J* = 11.4 Hz, 2 H), 3.57 (dd, *J* = 11.9, 3.0 Hz, 1 H), 3.46 (t, *J* = 11.0 Hz, 1 H), 2.54 (t, *J* = 13.8 Hz, 1 H), 2.37-2.22 (m, 2 H), 2.12 (d, *J* = 13.4 Hz, 1 H), 1.90-1.86 (m, 1 H), 1.75-1.68 (m, 1 H), 1.63-1.43 (m, 5 H), 1.39-1.32 (m, 1 H), 1.17 (s, 3 H), 0.89 (s, 3 H) ppm; ^{13}C NMR (101 MHz, $CDCl_3$) δ = 159.2, 153.6, 106.3, 103.4, 66.3, 46.4, 45.1, 39.9, 37.4, 33.0, 29.9, 28.4, 22.4, 20.6, 20.3 ppm; HRMS (ESI) calcd for $C_{15}H_{25}O^+$ $[M + H]^+$ 221.1900, found 221.1902; **SI-5**: **SI-5** is the enantiomer of **22**, the R_f, IR, ^1H and ^{13}C NMR, and HRMS of **SI-5** are the same to **22**; $[a]_D^{25}$ = −73.5 (c = 0.5, $CHCl_3$).

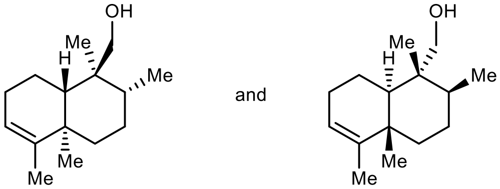

SI-6 and **25**

Alcohols SI-6 and 25. To a solution of alcohol **22** (1.57 g, 7.12 mmol 1.0 equiv) in CH_2Cl_2 (71 mL) was added $[Ir(COD)(PCy_3)(py)]^+[PF_6]^-$ (Crabtree's catalyst, 28.7 mg, 35.6 µmol, 0.005 equiv) at 23 °C. The resultant mixture was degassed before it was placed under an atmosphere of H_2 (balloon) and stirred for 2 h at 23 °C. The solvent was removed and the resultant residue was dissolved in benzene (50 mL). The reaction vessel was evacuated under vacuum and refilled with argon three times at −196 °C (liquid nitrogen bath). To this degassed solution was added a degassed solution of Co($Sal^{t\text{-Bu},\, t\text{-Bu}}$)Cl (0.226 g, 0.356 mmol, 0.05 equiv) in benzene (21 mL) and phenylsilane (0.154 g, 1.4 mmol, 0.2 equiv) at 23 °C and stirred at that temperature for 8 h. The resultant reaction was quenched with saturated aqueous NH_4Cl (50 mL) and extracted with EtOAc (3 × 50 mL). The combined organic phases were washed with brine (100 mL), dried ($MgSO_4$), filtered, and concentrated. The resultant residue was purified by flash column chromatography (silica gel, EtOAc/petroleum ether, 1:30→1:20) to give alcohol **SI-6** (1.16 g, 73% yield) as a colorless oil. **SI-6**: R_f = 0.42 (silica gel, EtOAc/petroleum ether, 1:10); $[a]_D^{25}$ = −65.6 (c = 0.5, CH_2Cl_2); IR (film) ν_{max} = 3358, 2958, 2927, 2858, 1598, 1478, 1459, 1438, 1382, 1259, 1177, 1133, 1075, 1051, 10039, 1019, 1001, 797, 788 cm^{-1}; ^1H NMR (400 MHz, $CDCl_3$) δ = 5.18 (s, 1 H), 3.44 (d, *J* = 11.6 Hz, 1 H), 3.31 (d, *J* = 11.6 Hz, 1 H), 2.07-1.98 (m, 2 H), 1.69 (dt, *J* = 13.1, 3.2 Hz, 1 H), 1.64 (d, *J* = 8.0 Hz, 1 H), 1.60 (s, 1 H), 1.57 (s, 3 H), 1.52-1.40 (m, 4 H), 1.26-1.17 (m, 1 H), 0.99 (s, 3 H), 0.81 (d, *J* = 6.8 Hz, 3 H), 0.56 (s, 3 H) ppm; ^{13}C NMR (101 MHz, $CDCl_3$) δ = 144.2, 120.6, 66.0, 44.3, 40.5, 38.1, 36.6 34.4, 27.4, 26.9, 20.0, 18.4, 18.1, 16.0, 13.4 ppm; HRMS (ESI) calcd for $C_{15}H_{27}O^+$ $[M + H]^+$ 223.2056, found 223.2057. **25**: **25** is the enantiomer of **SI-6**, the R_f, IR, ^1H and ^{13}C NMR, and HRMS of **25** are the same to **SI-6**; $[a]_D^{25}$ = +48.7 (c = 0.5, CH_2Cl_2).

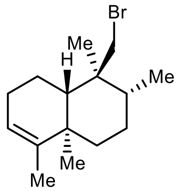

23

Bromide 23. To a solution of alcohol **SI-6** (2.22 g, 10.0 mmol, 1.0 equiv) in pyridine (100 mL) was added PPh_3 (7.87 g, 30.0 mmol, 3.0 equiv) and CBr_4 (6.63 g, 20.0 mmol, 2.0 equiv) at 0 °C. The resultant reaction was warmed to 60 °C and stirred at that temperature for 4 h before it was cooled to 0 °C. The reaction contents were diluted with EtOAc (100 mL) and quenched with saturated aqueous NH_4Cl

(100 mL) and extracted with EtOAc (3 × 80 mL). The combined organic phases were washed with brine (150 mL), dried ($MgSO_4$), filtered, and concentrated. The resultant residue was purified by flash column chromatography (silica gel, ppetroleum ether) to give bromide **23** (1.51 g, 53% yield) as a white solid. **23**: R_f = 0.75 (silica gel, petroleum ether); $[a]_D^{25}$ = −12.4 (c = 0.5, $CHCl_3$); IR (film) ν_{max} = 2998, 2961, 2926, 2857, 1597, 1462, 1450, 1438, 1382, 1255, 1238, 1188, 1131, 1075, 1020, 981, 922, 884, 853, 798, 665, 631 cm^{-1}; ^1H NMR (400 MHz, $CDCl_3$) δ = 5.21 (s, 1 H), 3.48 (d, *J* = 10.8 Hz, 1 H), 3.40 (d, *J* = 10.8 Hz, 1 H), 2.14-1.98 (m, 2 H), 1.70 (d, *J* = 12.4 Hz, 3 H), 1.59 (s, 3 H), 1.49-1.39 (m, 4 H), 1.23 (td, *J* = 12.4, 5.1 Hz, 1 H), 0.99 (s, 3 H), 0.82 (s, 3 H), 0.78 (d, *J* = 6.8 Hz, 3 H) ppm; ^{13}C NMR (101 MHz, $CDCl_3$) δ = 143.8, 120.9, 45.9, 43.4, 39.8, 38.0, 36.4, 35.7, 27.3, 26.7, 19.6, 18.3, 18.1, 15.7, 14.9 ppm; HRMS (ESI) calcd for $C_{15}H_{26}Br^+$ $[M + H]^+$ 285.1212, found 285.1196.

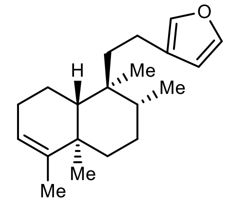

annonene (**3**)

Annonene (3). To an flame-dried Schlenk tube containing $NiBr_2$ (27.6 mg, 0.126 mmol, 0.3 equiv), $P(4\text{-}CF_3Ph)_3$ (58.8 mg, 0.126 mmol, 0.3 equiv), Mn (92.4 mg, 1.68 mmol, 4.0 equiv.) and oxalates **24** (0.232 g, 1.26 mmol, 3.0 equiv) was added a solution of bromide **23** (0.120 g, 0.421 mmol, 1.0 equiv) in DMSO/DMF (0.30 mL/0.30 mL). The resultant mixture was frozen in a liquid nitrogen bath, vacuumed and refilled with argon for three times. The reaction was stirred at 60 °C for 16 h before it was quenched with water (20 mL), extracted with EtOAc (3 ×20 mL). The combined organic phases were washed with brine (30 mL), dried ($MgSO_4$), filtered, and concentrated. The resultant residue was purified by flash column chromatography (silica gel, petroleum ether) to give annonene (**3**) (60.3 mg, 50% yield) as a colorless oil. Annonene (**3**): R_f = 0.50 (silica gel, petroleum ether); $[a]_D^{25}$ = −57.8 (c = 0.5, $CHCl_3$); IR (film) ν_{max} = 3407, 2960, 2925, 2855, 1655, 1596, 1501, 1458, 1438, 1383, 1260, 1161, 1095, 1076, 1066, 1026, 873, 798, 781 cm^{-1}; ^1H NMR (400 MHz, $CDCl_3$) δ = 7.34 (t, *J* = 1.7 Hz, 1 H), 7.20 (t, *J* = 1.7 Hz, 1 H), 6.26 (s, 1 H), 5.20 (s, 1 H), 2.35-2.18 (m, 2 H), 2.09-1.96 (m, 2 H),1.75-1.69 (m, 1 H), 1.67-1.59 (m, 5 H), 1.60-1.51 (m, 2 H), 1.49-1.46 (m, 1 H), 1.45-1.40 (m, 3 H), 1.24-1.17 (m, 1 H), 1.01 (s, 3 H), 0.83 (d, *J* = 6.6 Hz, 3 H), 0.74 (s, 3 H) ppm; ^{13}C NMR (101 MHz, $CDCl_3$) δ = 144.6, 142.8, 138.5, 126.0, 120.6, 111.2, 46.6, 38.9, 38.7, 38.3, 37.0, 36.4, 27.7, 27.0, 20.1, 18.5, 18.4, 18.3, 18.2, 16.2 ppm; HRMS (ESI) calcd for $C_{20}H_{31}O_3^+$ $[M + H]^+$ 287.2369, found 287.2369.

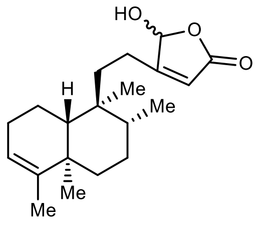

PL3 (**4**)

PL3 (4). To a solution of annonene (**3**) (57.3 mg, 0.200 mmol, 1.0 equiv) and DIPEA (0.258 g, 2.00 mmol, 10.0 equiv) in CH_2Cl_2 (15 mL) was added Rose bengal (6.1 mg, 0.006 mmol, 0.03 equiv) at 23 °C before it was bubbled with Oxygen for 10 min. The resultant mixture was irradiated with a tungsten lamp (200 W) at -78 °C for 3 h under oxygen atmosphere. The reaction mixture was filtrated through

Celite® and concentrated. The resultant residue was purified by flash column chromatography (silica gel, acetone/petroleum ether, 1:4→1:1) to give **PL3** (49.7 mg, 78% yield) as a white solid. **PL3**: R_f = 0.45 (silica gel, actone/petroleum ether, 1:2); $[a]_D^{25}$ = −50.8 (c = 0.5, $CHCl_3$); IR (film) $ν_{max}$ = 3357, 2960, 2927, 2873, 2860, 1741, 1647, 1597, 1457, 1437, 1384, 1262, 1176, 1128, 1076, 1042, 991, 954, 856, 798 cm^{-1}; ^{1}H NMR (400 MHz, $CDCl_3$) δ = 6.02 (s, 1 H), 5.82 (s, 1 H), 5.18 (s, 1 H), 5.04 (brs, 1 H), 2.43-2.14 (m, 2 H), 2.09-1.93 (m, 2 H), 1.73-1.62 (m, 2 H), 1.58 (s, 3 H), 1.55-1.47 (m, 3 H), 1.44 (d, *J* = 4.8 Hz, 3 H), 1.33 (d, *J* = 11.6 Hz, 1 H), 1.21-1.15 (m, 1 H), 1.00 (s, 3 H), 0.80 (t, *J* = 5.0 Hz, 3 H), 0.76 (s, 3 H) ppm; ^{13}C NMR (101 MHz, $CDCl_3$) δ = 172.1, 172.1, 171.1, 171.0, 144.5, 144.4, 120.6, 120.5, 117.0, 117.0, 99.4, 99.4, 46.6, 38.8, 38.8, 38.3, 36.8, 36.5, 36.4, 34.9, 34.8, 27.5, 26.9, 26.9, 21.5, 21.5, 20.0, 18.4, 18.4, 18.3, 18.3, 18.1, 16.1, 16.1 ppm; HRMS (ESI) calcd for$C_{20}H_{31}O_3^+$ $[M + H]^+$ 319.2268, found 319.2260.

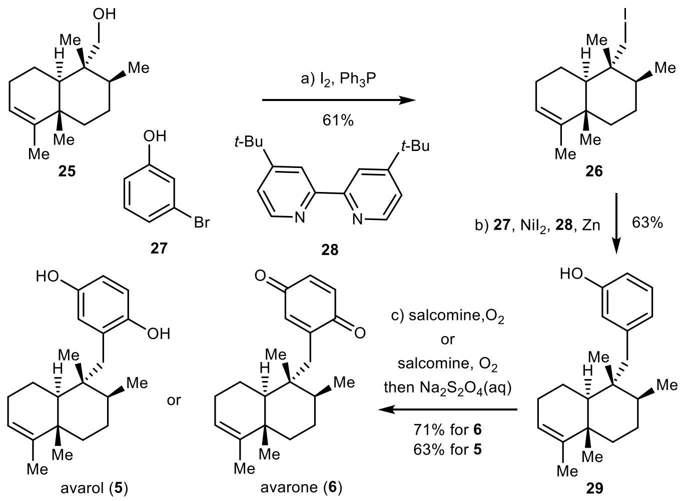

Supplementary Figure 2. Syntheses of avarone and avarol.

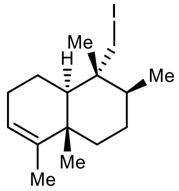

26

Iodide 26. To a solution of alcohol **25** (3.15 g, 14.2 mmol, 1.0 equiv) in benzene (140 mL) at 0 °C was added imidazole (2.89 g, 42.5 mmol, 3.0 equiv), PPh_3 (11.2 g, 42.5 mmol, 3.0 equiv) and I_2 (7.19 g, 28.3 mmol, 2.0 equiv). The reaction was heated to 50 °C and stirred at that temperature for 12 h before it was cooled to 0 °C. The resultant reaction was quenched with saturated aqueous NH_4Cl (100 mL), extracted with EtOAc (3 × 60 mL). The combined organic phases were washed with brine (100 mL), dried ($MgSO_4$), filtered, and concentrated. The resultant residue was purified by flash column chromatography (silica gel, petroleum ether) to give iodide **26** (2.87 g, 61% yield) as a white solid. **26**: R_f = 0.75 (silica gel, petroleum ether); $[a]_D^{25}$ = −25.2 (c = 0.5, $CHCl_3$); IR (film) $ν_{max}$ = 2958, 2925, 2871, 2855, 1598, 1460, 1425, 1379, 1218, 1160, 1017, 981, 852, 762 cm^{-1}; ^{1}H NMR (600 MHz, $CDCl_3$) δ = 5.21 (s, 1 H), 3.36 (d, *J* = 10.6 Hz, 1 H), 3.31 (d, *J* = 10.6 Hz, 1 H), 2.14-2.07 (m, 1 H), 2.06-2.01 (m, 1 H), 1.70 (dt, *J* = 12.8, 3.3 Hz, 1 H), 1.59 (s, 3 H), 1.55-1.52 (m, 2 H), 1.44-1.41 (m, 3 H), 1.38-1.33 (m, 1 H), 1.23 (td, *J* = 12.5, 5.0 Hz, 1 H), 0.98 (s, 3 H), 0.91 (s, 3 H), 0.75 (d, *J* = 6.7 Hz, 3 H) ppm; ^{13}C NMR (151 MHz, $CDCl_3$) δ =

143.8, 120.9, 48.5, 38.2, 38.0, 37.8, 36.5, 27.5, 26.8, 23.6, 19.3, 18.2, 18.1, 15.7, 13.8 ppm; HRMS (ESI) calcd for $C_{15}H_{26}I^+$ $[M + H]^+$ 333.1074, found 333.1076.

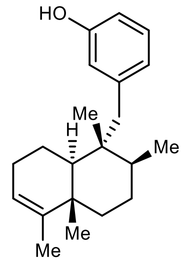

29

Phenol 29. To an flame-dried Schlenk tube containing NiI_2 (45.1 mg, 0.144 mmol, 0.2 equiv), 4,4'-di-*tert*-butyl-2,2'-bipyridine (**28**) (38.8 mg, 0.144 mmol, 0.2 equiv), Zn (0.142 g, 2.17 mmol, 3.0 equiv.) and 3-bromo-phenol (**27**) (0.375 g, 2.17 mmol, 3.0 equiv) was added a solution of iodide **26** (0.240 g, 0.722 mmol, 1.0 equiv) in DMAc (4.0 mL) at 23 °C. The reaction mixture was frozen in a liquid nitrogen bath, vacuumed and refilled with argon for three times. The resultant reaction was heated to 80 °C and stirred at that temperature for 16 h before it was cooled to 23 °C. The reaction contents were quenched with water (30 mL), extracted with EtOAc (3 ×30 mL). The combined organic phases were washed with brine (40 mL), dried ($MgSO_4$), filtered, and concentrated. The resultant residue was purified by flash column chromatography (silica gel, EtOAc/petroleum ether, 1:15→1:5) to give phenol **29** (0.136 g, 63% yield) as a colorless oil. **29**: R_f = 0.30 (silica gel, EtOAc/petroleum ether, 1:8); $[a]_D^{25}$ = +15.6 (c = 0.5, $CHCl_3$); IR (film) ν_{max} = 3356, 2957, 2926, 2858, 1612, 1587, 1490, 1457, 1381, 1259, 1157, 1022, 966, 875, 785, 758, 728, 696 cm^{-1}; ^1H NMR (400 MHz, $CDCl_3$) δ = 7.12 (t, *J* = 7.8 Hz, 1 H), 6.72 (d, *J* = 7.8 Hz, 1 H), 6.69-6.67 (dd, *J* = 7.8, 2.0 Hz, 1 H), 6.64 (t, *J* = 2.0 Hz, 1 H), 5.33 (s, 1 H), 5.15 (s, 1 H), 2.74 (d, *J* = 13.9 Hz, 1 H), 2.54 (d, *J* = 13.9 Hz, 1 H), 2.11-2.07 (m, 2 H), 2.00-1.96 (m, 1 H), 1.63-1.57 (m, 2 H), 1.52 (d, *J* = 1.6 Hz, 3 H), 1.48-1.35 (m, 3 H), 1.15 (d, *J* = 11.0 Hz, 1 H), 1.03 (s, 3 H), 1.00 (d, *J* = 6.4 Hz, 3 H), 0.98-0.93 (m, 1 H), 0.85 (s, 3 H) ppm; ^{13}C NMR (101 MHz, $CDCl_3$) δ = 154.9, 144.4, 140.9, 128.8, 123.6, 120.5, 117.9, 112.9, 45.4, 43.3, 41.1, 38.3, 36.1, 35.4, 27.4, 26.4, 20.1, 19.5, 18.2, 18.0, 16.8 ppm; HRMS (ESI) calcd for $C_{21}H_{31}O^+$ $[M + H]^+$ 299.2369, found 299.2367.

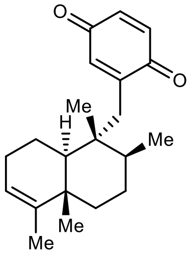

avarone (**6**)

Avarone (6). To a stirred solution of phenol **29** (0.113 g, 0.379 mmol, 1.0 equiv) in DMF (7.6 mL) was added salcomine (0.370 g, 1.14 mmol, 3.0 equiv) at 23 °C. The resultant mixture was degassed and refilled with oxygen. The reaction was stirred at 40 °C under an atmosphere of oxygen (balloon) for 2 days before it was quenched with half saturated aqueous NaCl (40 mL) and extracted with EtOAc (3 × 30 mL), dried ($MgSO_4$), filtered, and concentrated. The resultant residue was purified by flash column chromatography (silica gel, EtOAc/petroleum ether, 1:50) to afford avarone **(6)** (84.0 mg, 71% yield) as a yellow oil. Avarone **(6)**: R_f = 0.60 (silica gel, EtOAc/petroleum ether, 1:10); $[a]_D^{25}$ = +12.9 (c = 0.5, CH_2Cl_2); IR (film) ν_{max} = 2957, 2925, 2856, 1659, 1596, 1459, 1381, 1289, 1119, 1069, 912, 845, 821,

794 cm^{-1}; ^{1}H NMR (400 MHz, $CDCl_3$) δ = 6.75 (d, *J* = 10.0 Hz, 1 H), 6.70 (dd, *J* = 10.0, 2.2 Hz, 1 H), 6.50 (s, 1 H), 5.13 (s, 1 H), 2.64 (d, *J* = 13.5 Hz, 1 H), 2.43 (d, *J* = 13.5 Hz, 1 H), 2.05-2.01 (m, 1 H), 1.86-1.82 (m, 2 H), 1.65-1.62 (m, 1 H), 1.52-1.49 (m, 4 H), 1.38 (m, 2 H), 1.22-1.19 (m, 1 H), 1.06 -1.01 (m, 2 H), 0.99 (s, 3 H), 0.93 (d, *J* = 6.6 Hz, 3 H), 0.85 (s, 3 H) ppm; ^{13}C NMR (101 MHz, $CDCl_3$) δ = 187.5, 187.4, 147.5, 144.1, 137.3, 136.3, 136.1, 120.7, 47.1, 42.8, 38.6, 37.0, 36.2, 35.5, 27.5, 26.5, 20.2, 19.5, 18.2, 17.9, 16.8 ppm; HRMS (ESI) calcd for $C_{21}H_{29}O_2^+$ $[M + H]^+$ 313.2162, found 313.2157.

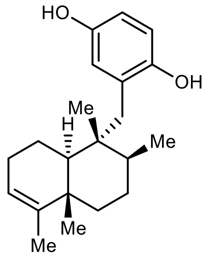

avarol (**5**)

Avarol (5). To a stirred solution of phenol **29** (0.113 g, 0.379 mmol, 1.0 equiv) in DMF (7.6 mL) was added salcomine (0.370 g, 1.14 mmol, 3.0 equiv) at 23 °C. The resultant mixture was degassed and refilled with oxygen. The reaction was stirred at 40 °C under an atmosphere of oxygen (balloon) for 2 days before it was quenched with saturated aqueous $Na_2S_2O_4$ (50 mL), extracted with EtOAc (3 × 50 mL), The combined organic phases were washed with brine (80 mL), dried ($MgSO_4$), filtered, and concentrated. The resultant residue was purified by flash column chromatography (silica gel, EtOAc/petroleum ether, 1:2→1:1) to give avarol **(5)** (75.0 mg, 63% yield) as a white solid. Avarol **(5)**: R_f = 0.6 (silica gel, EtOAc/petroleum ether, 1:1); $[a]_D^{25}$ = +9.2 (c = 0.8, CH_2Cl_2); IR (film) ν_{max} = 3375, 2957, 2926, 2856, 1711, 1654, 1599, 1504, 1452, 1381, 1195, 1123, 1037, 969, 877, 807, 754 cm^{-1}; ^{1}H NMR (600 MHz, $CDCl_3$) δ = 6.60 (d, *J* = 8.5 Hz, 1 H), 6.58 (d, *J* = 3.0 Hz, 1 H), 6.55 (dd, *J* = 8.5, 3.0 Hz, 1 H), 5.14 (s, 1 H), 4.43 (brs, 1 H), 4.39 (brs, 1 H), 2.68 (d, *J* = 14.3 Hz, 1 H), 2.57 (d, *J* = 14.3 Hz, 1 H), 2.08-2.06 (m, 2 H), 2.00-1.97 (m, 1 H), 1.60-1.57 (m, 2 H), 1.51 (d, *J* = 1.2, 3 H), 1.48-1.44 (m, 1 H), 1.39-1.37 (m, 2 H), 1.26 (t, *J* = 7.1 Hz, 1 H), 1.23 (d, *J* = 12.0 Hz, 1 H), 1.02 (s, 3 H), 1.00 (d, *J* = 6.5 Hz, 3 H), 0.86 (s, 3 H) ppm; ^{13}C NMR (101 MHz, $CDCl_3$) δ = 148.8 (2 C), 144.5, 126.7, 120.6, 119.8, 116.3, 114.0, 46.0, 41.9, 38.5, 37.7, 36.2, 35.9, 27.9, 26.8, 20.2, 19.9, 18.3, 17.8, 17.6 ppm; HRMS (ESI) calcd for $C_{21}H_{31}O_2^+$ $[M + H]^+$ 315.2319, found 315.2324.

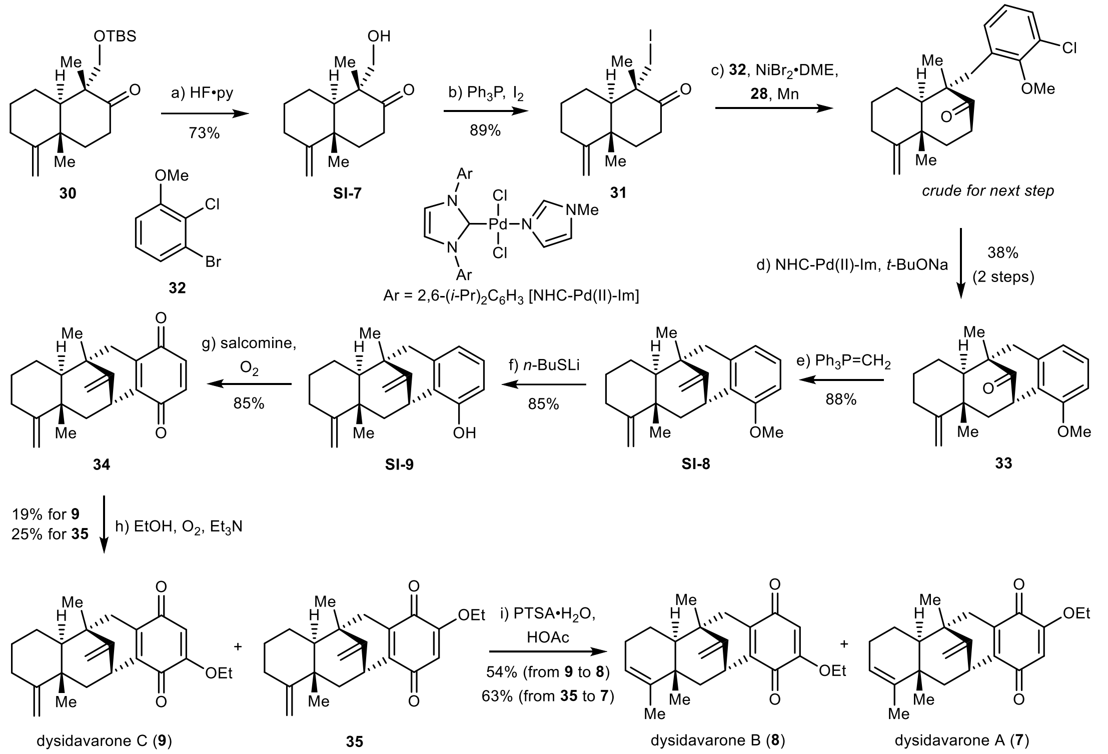

Supplementary Figure 3. Syntheses of dysidavarones A-C.

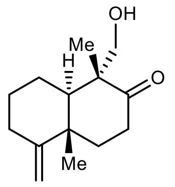

SI-7

Alcohol SI-7. To a solution of ketone **30** (0.893 g, 2.65 mmol, 1.0 equiv) in THF (21 mL) was added HF·py (5.3 mL) at 0 °C. The resultant reaction was stirred at that temperature for 5 min before it was warmed to 40 °C. Upon completion, the resultant mixture was poured into saturated aqueous $NaHCO_3$ (80 mL), and extracted with EtOAc (3 × 80 mL). The combined organic phases were washed with brine (100 mL), dried ($MgSO_4$), filtered, and concentrated. The resultant residue was purified by flash column chromatography (silica gel, EtOAc/ppetroleum ether, 1:10→1:5) to give alcohol **SI-7** (0.431 g, 73%) as a white solid. **SI-7**: R_f = 0.65 (silica gel, EtOAc/petroleum ether, 1:2); $[\alpha]_D^{23}$ = −110.4 (c = 0.5 in $CHCl_3$); IR (film) ν_{max} = 3449, 3087, 2932, 2864, 1696, 1629, 1597, 1382, 1314, 1265, 1191, 1049, 1159, 893, 801, 727, 541, 475 cm^{-1}; ^1H NMR (400 MHz, $CDCl_3$) δ = 4.58 (s, 1 H), 4.52 (s, 1 H), 3.63 (d, *J* = 11.5 Hz, 1 H), 3.32 (d, *J* = 11.2 Hz, 1 H), 2.79-2.70 (m, 1 H), 2.54 (s, 1 H), 2.37-2.29 (m, 2 H), 2.17-2.13 (m, 1 H), 1.98-1.82 (m, 3 H), 1.75 (dd, *J* = 11.6, 3.6 Hz, 1 H), 1.63-1.53 (m, 2 H), 1.38-1.29 (m, 1 H), 1.27 (s, 3 H), 1.00 (s, 3 H) ppm; ^{13}C NMR (101 MHz, $CDCl_3$) δ = 218.3, 157.4, 104.2, 66.4, 52.9, 47.3, 39.1, 36.0, 35.8, 32.8, 27.9, 22.6, 19.9, 17.0 ppm; HRMS (ESI) calcd for $C_{14}H_{23}O_2^+$ [M + H]$^+$ 223.1693, found 223.1693.

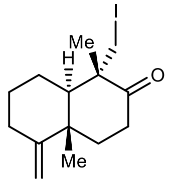

31

Iodide 31. To a solution of alcohol **SI-7** (0.760 g, 3.42 mmol, 1.0 equiv) in benzene (170 mL) was added PPh_3 (1.79 g, 6.84 mmol, 2.0 equiv), imidazole (0.931 g, 13.7 mmol, 4.0 equiv), and I_2 (1.74 g, 6.84 mmol, 2.0 equiv) at 0 °C. The resultant mixture was stirred at 0 °C for 5 minutes before it was warmed to 23 °C and stirred at that temperature for 24 h. The reaction mixture was quenched with saturated aqueous $NaHCO_3$ (100 mL), and extracted with EtOAc (3 × 100 mL). The combined organic phases were washed with brine (100 mL), dried ($MgSO_4$), filtered, and concentrated. The resultant residue was purified by flash column chromatography (silica gel, EtOAc/petroleum ether, 1:20) to give Iodide **31** (1.01 g, 89%) as a white solid. **31**: R_f = 0.35 (silica gel, EtOAc/petroleum ether, 1:10); $[\alpha]_D^{23}$ = −34.3 (c = 0.5 in $CHCl_3$), IR (film) ν_{max} = 3086, 2931, 2862, 1707, 1636, 1452, 1412, 1375, 1316, 1262, 1195, 1103, 1021, 954, 896, 805, 717, 638, 595, 533 cm^{-1}; ^1H NMR (400 MHz, $CDCl_3$) δ = 4.66 (s, 1 H), 4.61 (s, 1 H), 3.64 (d, *J* = 9.8 Hz, 1 H), 2.99 (d, *J* = 9.8 Hz, 1 H), 2.67-2.58 (m, 1 H), 2.50-2.44 (m, 1 H), 2.39-2.31 (m, 1 H), 2.23-2.18 (m, 1 H), 2.03 (dd, *J* = 12.3, 2.8 Hz, 1 H), 1.95-1.91 (m, 3 H), 1.63-1.51 (m, 1 H), 1.44-1.32 (m, 2 H), 1.23 (s, 3 H), 1.19 (s, 3 H) ppm; ^{13}C NMR (101 MHz, $CDCl_3$) δ= 212.7, 157.2, 104.9, 50.9, 49.9, 38.9, 35.1, 34.0, 32.9, 27.9, 23.0, 21.1, 18.9, 15.1 ppm; HRMS (ESI) calcd for $C_{14}H_{22}IO^+$ $[M + H]^+$ 333.0710, found 333.0707.

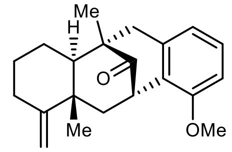

33

Methoxybenzene 33. To an flame-dried Schlenk tube containing $NiBr_2$·DME (37.2 mg, 0.120 mmol, 0.2 equiv), 4,4'-di-*tert*-butyl-2,2'-bipyridine (**28**) (32.3 mg, 0.120 mmol, 0.2 equiv), Mn (99.1 mg, 1.81 mmol, 3.0 equiv) and 1-bromo-2-chloro-3-methoxybenzene (**32**) (0.400 g, 1.81 mmol, 3.0 equiv) was added a solution of iodide **31** (0.200 g, 0.602 mmol, 1.0 equiv) in DMAc (3.0 mL) at 23 °C. The reaction mixture was frozen in a liquid nitrogen bath, vacuumed and refilled with argon for three times. The resultant reaction was stirred at 25 °C for 24 h before it was quenched with water (20 mL), extracted with EtOAc (3 × 20 mL). The combined organic phases were washed with brine (30 mL), dried ($MgSO_4$), filtered, and concentrated. Pressing forward without any further purification. The resultant residue was dissolved in 1,4-dioxane (6.0 mL), and NHC-Pd(II)-Im (39.1 mg, 60.2 μmol, 0.1 equiv) and *t*-BuONa (0.174 g, 1.81 mmol, 3.0 equiv) were added sequentially at 23 °C. The reaction mixture was frozen in a liquid nitrogen bath, vacuumed and refilled with argon for three times. The resultant mixture was heated to 110 °C and stirred at that temperature for 6 h before it was cooled to 23 °C. The resultant reaction mixture was quenched with saturated aqueous NH_4Cl (30 mL), and extracted with EtOAc (3 × 50 mL). The combined organic phases were washed with brine (80 mL), dried ($MgSO_4$), filtered, and concentrated. The resultant residue was purified by flash column chromatography (silica gel, EtOAc/petroleum ether, 1:40) to give methoxybenzene **33** (71.0 mg, 38% for 2 steps) as a white solid. **33**: R_f = 0.60 (silica gel, EtOAc/petroleum ether, 1:10); $[\alpha]_D^{24}$ = −4.1 (c = 0.5 in $CHCl_3$); IR (film) ν_{max} = 3741, 3444, 2959, 2923,

2856, 1722, 1592, 1460, 1416, 1260, 1091, 1026, 868, 801, 687, 617, 472 cm^{-1}; ^{1}H NMR (600 MHz, $CDCl_3$) δ = 7.13 (t, *J* = 7.9 Hz, 1 H), 6.73 (d, *J* = 8.1 Hz, 1 H), 6.61 (d, *J* = 7.6 Hz, 1 H), 4.58 (s, 1 H), 4.56 (s, 1 H), 3.84-3.83 (m, 4 H), 3.10 (d, *J* = 16.4 Hz, 1 H), 2.77 (d, *J* = 16.4 Hz, 1 H), 2.41-2.34 (m, 2 H), 2.28-2.18 (m, 2 H), 2.10 (d, *J* = 13.8 Hz, 1 H), 1.81 (d, *J* = 12.6 Hz, 1 H), 1.67 (d, *J* = 13.1 Hz, 1 H), 1.60-1.52 (m, 2 H), 1.33-1.26 (m, 1 H), 1.18 (s, 3 H), 0.99 (s, 3 H) ppm; ^{13}C NMR (151 MHz, $CDCl_3$) δ = 220.4, 157.3, 156.8, 134.4, 132.3, 127.5, 120.7, 108.3, 106.5, 55.6, 49.7, 48.9, 48.0, 45.3, 42.8, 38.9, 33.2, 27.5, 24.9, 21.6, 18.0 ppm; HRMS (ESI) calcd for $C_{21}H_{27}O_2^+$ $[M + H]^+$ 311.2006, found 311.2004.

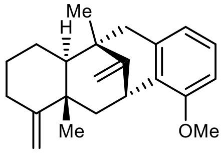

SI-8

Alkene SI-8. To a suspension of PPh_3CH_3Br (1.15 g, 3.22 mmol, 10.0 equiv) in toluene (25 mL) was added *t*-BuOK (0.361 g, 3.22 mmol, 10.0 equiv) at 23 °C. The resultant reaction mixture was heated to 90 °C and stirred at that temperature for 1 h before it was cooled to 23 °C. The resultant mixture was added a solution of methoxybenzene **33** (0.100 g, 0.322 mol 1.0 equiv) in toluene (7.0 mL) at 23 °C. The resultant reaction was heated to 120 °C and stirred at that temperature for 8 h before it was cooled to 23 °C. The reaction contents were quenched with saturated aqueous NH_4Cl (80 mL), and extracted with EtOAc (3 × 50 mL). The combined organic layer was washed with brine (100 mL), dried ($MgSO_4$), filtered, and concentrated. The resultant residue was purified by flash column chromatography (silica gel, petroleum ether) to give alkene **SI-8** (87.4 mg, 88% yield) as a white solid. **SI-8**: R_f = 0.20 (silica gel, petroleum ether); $[\alpha]_D^{23}$ = +28.1 (c = 0.5 in $CHCl_3$); IR (film) ν_{max} = 3739, 3449, 3080, 2928, 1635, 1588, 1464, 1375, 1324, 1255, 1216, 1124, 1081, 1029, 887, 777, 741, 622, 518, 470, 423 cm^{-1}; ^{1}H NMR (400 MHz, $CDCl_3$) δ = 7.07 (t, *J* = 7.9 Hz, 1 H), 6.70 (d, *J* = 8.1 Hz, 1 H), 6.60 (d, *J* = 7.6 Hz, 1 H), 4.92 (d, *J* = 1.4 Hz, 1 H), 4.82 (d, *J* = 1.4 Hz, 1 H), 4.52 (s, 1 H), 4.51 (s, 1 H), 3.97 (dd, *J* = 8.0, 3.0 Hz, 1 H), 3.87 (s, 3 H), 2.85 (d, *J* = 15.9 Hz, 1 H), 2.47 (d, *J* = 15.9 Hz, 1 H), 2.27-2.18 (m 1 H), 2.15-2.04 (m, 3 H), 1.80-1.67 (m, 3 H), 1.61-1.50 (m, 1 H), 1.24 (s, 3 H), 1.14 (s, 3 H), 1.12-1.03 (m, 1 H) ppm; ^{13}C NMR (101 MHz, $CDCl_3$) δ = 159.1, 156.3, 155.5, 136.7, 134.1, 126.4, 121.4, 107.7, 105.7, 103.8, 55.5, 48.9, 47.2, 44.0, 39.9, 38.9, 38.3, 33.3, 27.5, 25.1, 22.5, 21.2 ppm; HRMS (ESI) calcd for $C_{22}H_{29}O^+$ $[M + H]^+$ 309.2213, found 309.2210.

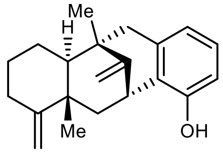

SI-9

Phenol SI-9. To a solution of *n*-BuSH (0.208 g, 2.31 mmol, 7.5 equiv) in HMPA (3.1 mL) was added *n*-BuLi (0.96 mL, 2.4 M in pentane, 2.31 mmol, 7.5 equiv) dropwise at 0 °C. The resultant mixture was stirred at 0 °C for 30 min before a solution of alkene **SI-8** (95.0 mg, 0.308 mmol, 1.0 equiv) was added. The resultant reaction mixture was stirred at 0 °C for 10 min and allowed to heat to 110 °C. Upon completion, the reaction was quenched with saturated aqueous NH_4Cl (30 mL), and extracted with EtOAc (3 × 30 mL). The combined organic layer was washed with brine (3 × 30 mL), dried ($MgSO_4$), filtered, and concentrated. The resultant residue was purified by flash column chromatography (silica gel, EtOAc/petroleum ether, 1:30→1:10) to give phenol **SI-9** (77.1 mg, 85% yield) as a colorless oil. **SI-9**:

R_f = 0.30 (silica gel, EtOAc/petroleum ether, 1:10); $[\alpha]_D^{23}$ = +24.0 (c = 0.5 in $CHCl_3$); IR (film) ν_{max} = 3451, 2925, 2860, 1715, 1590, 1459, 1376, 1313, 1262, 1189, 1092, 1027, 885, 800, 742, 617, 519, 476 cm^{-1}; ^1H NMR (400 MHz, $CDCl_3$) δ = 6.97 (t, J = 7.8 Hz, 1 H), 6.60 (d, J = 8.0 Hz, 1 H), 6.58 (d, J = 7.8 Hz, 1 H), 4.94 (d, J = 1.0 Hz, 1 H), 4.84 (d, J = 1.1 Hz, 1 H), 4.70 (s, 1 H), 4.51 (d, J = 1.7 Hz, 2 H), 3.90 (dd, J = 7.2, 3.6 Hz, 1 H), 2.85 (d, J = 16.0 Hz, 1 H), 2.46 (d, J = 16.0 Hz, 1 H), 2.22-2.17 (m, 3 H), 2.08-2.03 (m, 1 H), 1.80-1.65 (m, 3 H), 1.60-1.53 (m, 1 H), 1.24 (s, 3 H), 1.14 (s, 3 H), 1.11-1.03 (m, 1 H) ppm; ^{13}C NMR (101 MHz, $CDCl_3$) δ = 158.9, 154.9, 152.2, 137.3, 132.1, 126.6, 121.8, 112.7, 105.8, 104.2, 48.8, 47.2, 43.7, 39.9, 39.0, 38.4, 33.3, 27.5, 25.1, 22.5, 21.2 ppm; HRMS (ESI) calcd for $C_{21}H_{27}O^+$ $[M + H]^+$295.2056, found 295.2055.

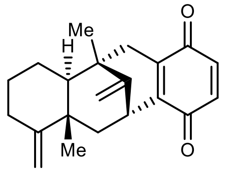

34

Quinone 34. To a solution of phenol **SI-9** (77.1 mg, 0.262 mmol, 1.0 equiv) in MeCN (10 mL) was added salcomine (0.255 g, 0.786 mmol, 3.0 equiv) at 23 °C. The resultant mixture was degassed and refilled with oxygen. The reaction was stirred at 23 °C under an atmosphere of oxygen (balloon) for 3 h before it was quenched with saturated aqueous NH_4Cl (30 mL), and extracted with EtOAc (3 × 30 mL). The combined organic phases were washed with brine (50.0 mL), dried ($MgSO_4$), filtered, and concentrated. The resultant residue was purified by flash column chromatography (silica gel, EtOAc/petroleum ether, 1:30) to give quinone **34** (68.6 mg, 85%) as a yellow solid. **34**: R_f = 0.60 (silica gel, EtOAc/ petroleum ether, 1:10); $[\alpha]_D^{23}$ = +37.9 (c = 0.5 in $CHCl_3$); IR (film) ν_{max} = 3750, 3451, 2924, 2857, 1735, 1649, 1598, 1459, 1383, 1307, 1260, 1095, 1027, 800, 615, 522, 475, 422 cm^{-1}; ^1H NMR (400 MHz, $CDCl_3$) δ = 6.72 (d, J = 10.1 Hz, 1 H), 6.67 (d, J = 10.0 Hz, 1 H), 4.92 (s, 1 H), 4.86 (s, 1 H), 4.54 (s, 1 H), 4.49 (s, 1 H), 3.73 (d, J = 10.6 Hz, 1 H), 2.73 (d, J = 18.9 Hz, 1 H), 2.25-2.04 (m, 3 H), 2.00-1.92 (m, 2 H), 1.83-1.78 (m, 1 H), 1.71-1.66(m, 1 H), 1.58-1.52 (m, 1 H), 1.50-1.43 (m, 1 H), 1.24 (s, 3 H), 1.15-1.10 (m, 1 H), 1.08 (s, 3 H) ppm; ^{13}C NMR (101 MHz, $CDCl_3$) δ = 188.1, 186.7, 158.2, 152.0, 148.8, 140.8, 136.7, 136.4, 106.2, 105.9, 49.3, 43.3, 42.1, 39.4, 38.3, 37.4, 33.2, 27.6, 25.0, 21.9, 20.8 ppm; HRMS (ESI) calcd for $C_{21}H_{25}O_2^+$ $[M + H]^+$ 309.1849, found 309.1848.

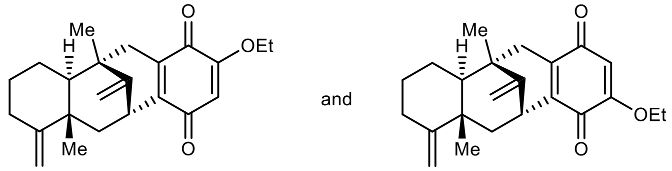

35 dysidavarone C (**9**)

Quinone 35 and Dysidavarone C (9). To a solution of quinone **34** (16.8 mg, 0.054 mmol, 1.0 equiv) in EtOH (2.0 mL) was added Et_3N (0.110 g, 1.09 mmol, 20.0 equiv) at 23 °C under oxygen. The resultant mixture was then heated to 30 °C and stirred at that temperature for 12 h before it was quenched with saturated aqueous NH_4Cl (5 mL) and extracted with EtOAc (3 × 10 mL). The combined organic phases were washed with brine (10 mL), dried ($MgSO_4$), filtered, and concentrated. The resultant residue was subjected to HPLC (Shimadzu CBM-20A, Shimadzu Prep C18 column, 5 μm, 10 × 250 mm) using acetonitrile/water (80:20, 3 mL/min) as eluent to give quinone **35** (t_R = 36.80 min) and dysidavarone C (**9**) (t_R = 38.40 min). After removal of the solvent under vacuum, quinone **35** (4.9 mg, 25% yield) as a yellow

solid and dysidavarone C **(9)** (3.6 mg, 19% yield) as a yellow solid were obtained. Quinone **35**: R_f = 0.25 (silica gel, EtOAc/petroleum ether, 1:10); $[\alpha]_D^{22}$ = −9.1 (c = 0.5 in $CHCl_3$); IR (film) ν_{max} = 3452, 2924, 2856, 1597, 1461, 1416, 1313, 1260, 1211, 1120, 1031, 861, 797, 687, 622, 517, 477, 422 cm^{-1}; ^1H NMR (400 MHz, $CDCl_3$) δ = 5.85 (s, 1 H), 4.91 (s, 1 H), 4.85 (s, 1 H), 4.54 (s, 1 H), 4.49 (s, 1 H), 3.98 (q, *J* = 7.0 Hz, 2 H), 3.75 (d, *J* = 10.6 Hz, 1 H), 2.72 (d, *J* = 18.6 Hz, 1 H), 2.21-2.04 (m, 3 H), 1.99-1.94 (m, 2 H), 1.84-1.77 (m, 1 H), 1.70-1.66 (m, 1 H), 1.55-1.51 (m, 1 H), 1.49-1.42 (m, 4 H), 1.23 (s, 3 H), 1.13-1.09 (m, 1H), 1.08 (s, 3 H) ppm; ^{13}C NMR (101 MHz, $CDCl_3$) δ = 186.8, 182.9, 158.3, 157.9, 152.2, 149.1, 138.8, 107.8, 106.2, 105.8, 65.2, 49.4, 43.0, 42.2, 39.4, 38.4, 37.4, 33.2, 27.6, 25.0, 21.9, 20.8, 14.0 ppm; HRMS (ESI) calcd for $C_{23}H_{29}O_3^+$ $[M + H]^+$ 353.2111, found 353.2110. Dysidavarone C **(9)**: R_f = 0.25 (silica gel, EtOAc/petroleum ether, 1:10); $[\alpha]_D^{15}$ = +55.1 (c = 0.3 in MeOH); IR (film) ν_{max} = 3082, 2926, 2855, 1670, 1643, 1601, 1445, 1380, 1340, 1304, 1267, 1214, 1110, 1032, 963, 893, 849, 811, 738, 626, 518, 477, 418 cm^{-1}; ^1H NMR (400 MHz, $CDCl_3$) δ = 5.81 (s, 1H), 4.91 (s, 1 H), 4.85 (s, 1 H), 4.54 (s, 1 H), 4.48 (s, 1 H), 3.98 (q, *J* = 7.0 Hz, 2 H), 3.75 (d, *J* = 10.5 Hz, 1 H), 2.74 (d, *J* = 19.1 Hz, 1 H), 2.24-2.04 (m, 3 H), 1.99-1.92 (m, 2 H), 1.83-1.78 (m, 1 H), 1.70-1.66 (m, 1 H), 1.55-1.51 (m, 1 H), 1.49-1.46 (m, 4 H), 1.23 (s, 3 H), 1.13-1.09 (m, 1H), 1.08 (s, 3 H) ppm; ^{13}C NMR (101 MHz, $CDCl_3$) δ = 188.2, 181.5, 158.3, 158.1, 152.2, 146.8, 141.2, 107.5, 106.2, 105.8, 65.2, 49.3, 43.3, 41.9, 39.4, 38.3, 37.2, 33.2, 27.6, 25.0, 22.0, 20.8, 14.0 ppm; HRMS (ESI) calcd for $C_{23}H_{29}O_3^+$ $[M + H]^+$ 353.2111, found 353.2111.

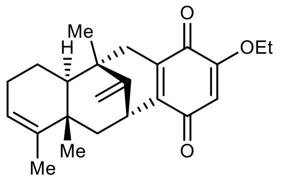

dysidavarone A (**7**)

Dysidavarone A (7). To a solution of quinone **35** (4.9 mg, 0.014 mmol, 1.0 equiv) in AcOH (1.0 mL) was added *p*-TsOH·H_2O (0.5 mg, 0.003 mmol, 0.2 equiv) at 23 °C. The reaction was stirred at 40 °C for 6 h before it was poured into saturated aqueous $NaHCO_3$ (10 mL), and extracted with EtOAc (3 × 10 mL). The combined organic phases were washed with saturated aqueous $NaHCO_3$ (10 mL) and brine (10 mL), dried ($MgSO_4$), filtered, and concentrated. The resultant residue was purified by flash column chromatography (silica gel, EtOAc/petroleum ether, 1:10→1:5) to give dysidavarone A **(7)** (3.1 mg, 63%) as a yellow solid. Dysidavarone A **(7)**: R_f = 0.25 (silica gel, EtOAc/petroleum ether, 1:10); $[\alpha]_D^{15}$ = +74.8 (c = 0.3 in MeOH); IR (film) ν_{max} = 2926, 2856, 1737, 1671, 1643, 1601, 1451, 1378, 1341, 1270, 1218, 1159, 1102, 1033, 892, 852, 797, 736, 700, 674, 628, 576, 525, 465, 418 cm^{-1}; ^1H NMR (400 MHz, $CDCl_3$) δ = 5.83 (s, 1 H), 5.07 (s, 1 H), 4.90 (s, 1 H), 4.84 (s, 1 H), 3.98 (q, *J* = 7.0 Hz, 2 H), 3.70 (d, *J* = 10.6 Hz, 1 H), 2.76 (d, *J* = 18.6 Hz, 1 H), 2.18-2.12 (m, 1 H), 2.02-1.97 (m, 2 H), 1.88-1.80 (m, 1 H), 1.69-1.65 (m, 2 H), 1.53-1.50 (m, 4 H), 1.48-1.44 (m, 4 H), 1.24 (s, 3 H), 1.04 (s, 3 H) ppm; ^{13}C NMR (151 MHz, CDCl3) δ = 186.8, 182.9, 157.9, 152.7, 149.2, 142.8, 139.4, 119.6, 107.7, 105.7, 65.2, 47.4, 43.1, 42.0, 38.0, 37.8, 37.3, 26.6, 20.6, 20.5, 19.9, 18.2, 14.0 ppm; HRMS (ESI) calcd for $C_{23}H_{29}O_3^+$ $[M + H]^+$ 353.2111, found 353.2111.

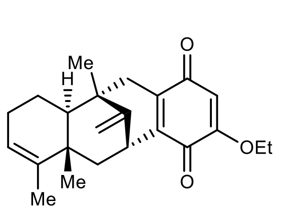

dysidavarone B (**8**)

Dysidavarone B (8). To a solution of dysidavarone C (**9**, 3.7 mg, 0.010 mmol, 1.0 equiv) in AcOH (1.0 mL) was added *p*-TsOH·H_2O (0.4 mg, 2.10 μmol, 0.2 equiv) at 23 °C. The reaction was stirred at 40 °C for 6 h before it was poured into saturated aqueous $NaHCO_3$ (10 mL), and extracted with EtOAc (3 × 10 mL). The combined organic phases were washed with saturated aqueous $NaHCO_3$ (10 mL) and brine (10 mL), dried ($MgSO_4$), filtered, and concentrated. The resultant residue was purified by flash column chromatography (silica gel, EtOAc/petroleum ether, 1:10→1:5) to give dysidavarone B (**8**, 2.0 mg, 54%) as a yellow solid. Dysidavarone B (**8**): R_f = 0.25 (silica gel, EtOAc/petroleum ether, 1:10); $[\alpha]_D^{15}$ = +48.7 (c = 0.2 in MeOH); IR (film) ν_{max} = 2926, 2856, 1733, 1670, 1644, 1601, 1454, 1381, 1343, 1306, 1266, 1211, 1107, 1033, 970, 894, 848, 804, 738, 512, 464, 418 cm^{-1}; ^{1}H NMR (600 MHz, CDCl3) δ = 5.81 (s, 1 H), 5.08 (s, 1 H), 4.90 (s, 1H), 4.84 (s, 1H), 3.97 (q, *J* = 7.1 Hz, 2 H), 3.70 (d, *J* = 10.5 Hz, 1 H), 2.78 (d, *J* = 19.1 Hz, 1 H), 2.15 (dd, *J* = 13.8, 10.4 Hz, 1 H), 2.02-1.98 (m, 2 H), 1.88-1.82 (m, 1 H), 1.69-1.64 (m, 2 H), 1.54-1.51 (m, 4 H), 1.48-1.45 (m, 4 H), 1.24 (s, 3 H), 1.04 (s, 3 H) ppm; ^{13}C NMR (151 MHz, CDCl3) δ = 188.2, 181.4, 158.0, 152.7, 146.8, 142.8, 141.8, 119.6, 107.5, 105.7, 65.2, 47.3, 43.3, 41.7, 37.9, 37.6, 37.2, 26.5, 20.6 (2C), 19.9, 18.1, 14.0 ppm; HRMS (ESI) calcd for $C_{23}H_{29}O_3^+$ $[M + H]^+$ 353.2111, found 353.2111.

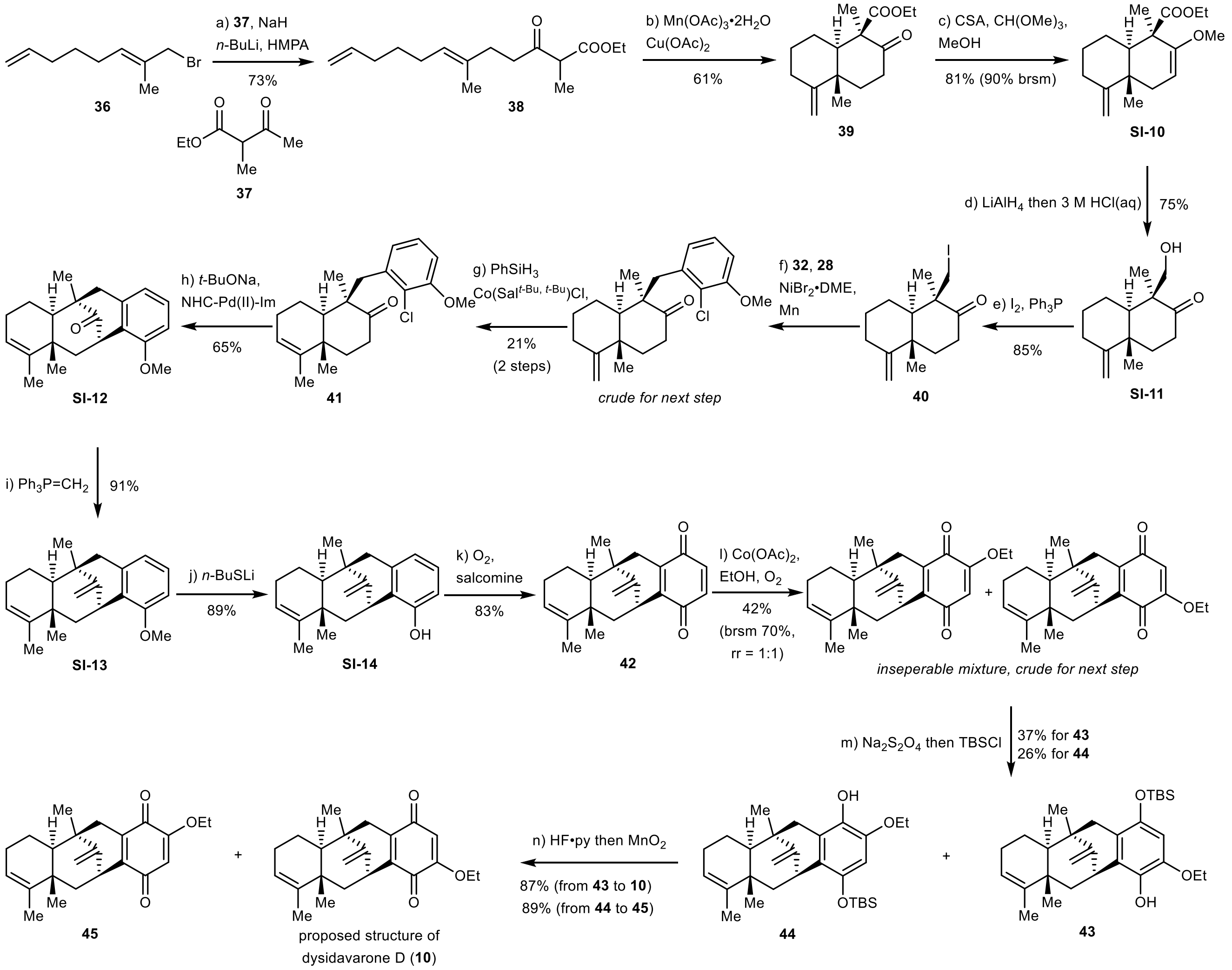

Supplementary Figure 4. Synthesis of proposed structure of dysidavarone D.

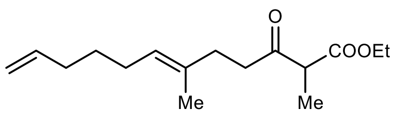

38

Keto ester 38. To a suspension of NaH (2.00 g, 60% dispersion in mineral oil, 50.0 mmol, 5.0 equiv) in THF (85 mL) was added HMPA (2.1 mL, 12.0 mmol, 1.2 equiv) and ethyl 2-methylacetoacetate (**37**) (7.21 g, 50.0 mmol, 5.0 equiv) dropwise over 30 min at 0 °C. The resultant mixture was stirred at 0 °C for 10 min before *n*-BuLi (21 mL, 2.4 M in hexanes, 50.0 mmol, 5.0 equiv) was added dropwise over 30 min. The reaction mixture was stirred at 0 °C for 1 h before a solution of the allylic bromide **36**[3] (2.03 g, 10.0 mmol, 1.0 equiv) in THF (15 mL) was added dropwise over 20 min. The resultant reaction was allowed to warm to 23 °C and stirred at that teperature for 1h before it was quenched with saturated aqueous NH_4Cl (100 mL) and extracted with EtOAc (3 × 100 mL). The combined organic phases were washed with brine (2 × 150 mL), dried ($MgSO_4$), filtered, and concentrated. The resultant residue was purified by flash column chromatography (silica gel, EtOAc/petroleum ether, 1:30) to give ester **38** (1.94 g, 73% yield) as a colorless oil. **38**: R_f = 0.55 (silica gel, EtOAc/petroleum ether, 10:1); IR (film) ν_{max} = 3078, 2981, 2929, 2858, 1746, 1717, 1641, 1455, 1412, 1377, 1323, 1240, 1192, 1121, 1069, 993, 911, 860 cm^{-1}; ^1H NMR (600 MHz, $CDCl_3$) δ = 5.77 (td, *J* = 16.8, 6.7 Hz, 1 H), 5.10 (t, *J* = 7.3 Hz, 1 H), 4.97 (d, *J* = 17.2 Hz, 1 H), 4.91 (d, *J* = 10.2 Hz, 1 H), 4.16 (q, *J* = 7.0 Hz, 2 H), 3.50 (q, *J* = 7.1 Hz, 1 H), 2.67-2.55 (m, 2 H), 2.24 (t, *J* = 7.6 Hz, 2 H), 2.01 (q, *J* = 7.0 Hz, 2 H), 1.96 (q, *J* = 7.3 Hz, 2 H), 1.57 (s, 3 H), 1.41-1.36 (m, 2 H), 1.30 (d, *J* = 7.2 Hz, 3 H), 1.24 (t, *J* = 7.1 Hz, 3 H) ppm; ^{13}C NMR (151 MHz, $CDCl_3$) δ = 205.6, 170.7, 138.9, 133.6, 125.2, 114.5, 61.4, 53.0, 40.2, 33.4, 29.0, 27.4, 16.1, 14.2, 12.8 ppm; HRMS (ESI) calcd for $C_{16}H_{27}O_3^+$ $[M + H]^+$ 267.1955, found 267.1955.

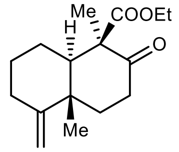

39

Ester 39. A suspension of $Mn(OAc)_3 \cdot 3H_2O$ (3.91 g, 14.6 mmol, 2.0 equiv), $Cu(OAc)_2$ (1.46 g, 8.01 mmol, 1.1 equiv), and keto ester **38** (1.94 g, 7.28 mmol, 1.0 equiv) in AcOH (73 mL) was frozen in a liquid nitrogen bath, vacuumed and refilled with argon for three times. The resultant reaction mixture was allowed to warm to 40 °C and stirred at that temperature for 24 h before it was diluted with H_2O (100 mL) and extracted with EtOAc (5 × 100 mL). The combined organic phases were washed with saturated aqueous $NaHCO_3$ (200 mL), brine (200 mL), dried ($MgSO_4$), filtered, and concentrated. The resultant residue was purified by flash column chromatography (silica gel, EtOAc/petroleum ether, 1:30) to give ester **39** (1.17 g, 61% yield) as a colorless oil. **39**: R_f = 0.55 (silica gel, EtOAc/petroleum ether, 10:1); IR (film) ν_{max} = 2981, 2939, 2858, 1714, 1635, 1447, 1377, 1320, 1257, 1220, 1193, 1097, 1024, 896, 869 cm^{-1}; ^1H NMR (400 MHz, $CDCl_3$) δ = 4.61 (s, 1 H), 4.55 (s, 1 H), 4.17-4.08 (m, 2 H), 3.02 (td, *J* = 14.8, 6.1 Hz, 1 H), 2.48 (ddd, *J* = 14.6, 4.5, 2.7 Hz, 1 H), 2.30 (td, *J* = 13.7, 4.7 Hz, 1 H), 2.16-2.11 (m, 1 H), 2.04-1.96 (m, 2 H), 1.93-1.79 (m, 3 H), 1.35 (dd, *J* = 12.4, 3.0 Hz, 1 H), 1.30 (s, 3 H), 1.24 (t, *J* = 7.1 Hz, 3 H), 1.20 (dd, *J* = 7.1, 3.1 Hz, 1 H), 1.15 (s, 3 H) ppm; ^{13}C NMR (101 MHz, $CDCl_3$) δ = 208.6, 173.4, 156.9, 105.5, 61.2, 57.8, 56.1, 39.8, 37.4, 37.3, 33.0, 28.4, 24.3, 21.3, 18.4, 14.0 ppm; HRMS (ESI) calcd for $C_{16}H_{25}O_3^+$ $[M + H]^+$ 265.1798, found 265.1798.

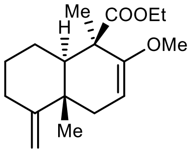

SI-10

Vinyl methyl ether SI-10. To a solution of ester **39** (2.21 g, 8.36 mmol, 1.0 equiv) in MeOH (84 mL) was added $CH(OMe)_3$ (6.9 mL, 62.7 mmol, 7.5 equiv) and CSA (0.194 g, 0.836 mmol, 0.1 equiv) at 23 °C. The reaction mixture was stirred at that temperature for 4.5 h before it was quenched with saturated aqueous $NaHCO_3$ (100 mL) and extracted with EtOAc (3 × 100 mL). The combined organic phases were washed with brine (150 mL), dried ($MgSO_4$), filtered, and concentrated. The resultant residue was purified by flash column chromatography (silica gel, EtOAc/petroleum ether, 1:50) to recover ester **39** (0.219 g) and afford vinyl methyl ether **SI-10** (1.89 g, 81% yield, 90% yield brsm) as a colorless oil. **SI-10**: R_f = 0.70 (silica gel, EtOAc/petroleum ether, 1:10); IR (film) ν_{max} = 2982, 2936, 2859, 1731, 1676, 1636, 1466, 1446, 1379, 1313, 1226, 1199, 1161, 1150, 1103, 1046, 1024, 894, 793, 732 cm^{-1}; ^1H NMR (400 MHz, $CDCl_3$) δ = 4.72 (d, *J* = 7.0 Hz, 1 H), 4.58 (s, 1 H), 4.54 (s, 1 H), 4.15-4.06 (m, 2 H), 3.51 (s, 3 H), 2.31-2.24 (m, 2 H), 2.13-2.06 (m, 2 H), 1.87-1.85 (m, 1 H), 1.78 (d, *J* = 7.9 Hz, 1 H), 1.41-1.38 (m, 1 H), 1.30 (s, 3 H), 1.25-1.21 (m, 5 H), 1.12 (s, 3 H) ppm; ^{13}C NMR (101 MHz, $CDCl_3$) δ = 173.9, 158.0, 156.3, 104.4, 92.9, 60.4, 54.7, 52.4, 49.9, 38.9, 35.2, 33.0, 28.1, 25.4, 24.1, 20.3, 14.2 ppm; HRMS (ESI) calcd for $C_{17}H_{27}O_3^+$ $[M + H]^+$ 279.1955, found 279.1952.

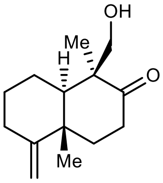

SI-11

Alcohol SI-11. To a suspension of $LiAlH_4$ (1.24 g, 32.8 mmol, 4.0 equiv) in THF (50 mL) was added vinyl methyl ether **SI-10** (2.28 g, 8.19 mmol, 1.0 equiv) in THF (10 mL) dropwise over 10 min at 0 °C. The resultant reaction was allowed to warm to 23 °C and stirred at that temperature for 15 h before it was quenched with 3 M HCl (50 mL) dropwise at 0 °C. The reaction mixture was warmed to 23 °C and stirred at that temperature for 1 h. The layers were separated and the aqueous layer was extracted with EtOAc (3 × 50 mL). The combined organic phases were washed with saturated aqueous $NaHCO_3$ (100 mL), brine (100 mL), dried ($MgSO_4$), filtered, and concentrated. The resultant residue was purified by flash column chromatography (silica gel, EtOAc/petroleum ether, 1:10→1:3) to give alcohol **SI-11** (1.37 g, 75% yield) as a white solid. **SI-11**: R_f = 0.40 (silica gel, EtOAc/petroleum ether, 1:3); IR (film) ν_{max} = 3445, 3085, 2934, 2858, 1707, 1636, 1447, 1378, 1316, 1226, 1123, 1106, 1050, 1036, 988, 942, 892, 869, 848, 746, 701 cm^{-1}; ^1H NMR (600 MHz, $CDCl_3$); δ = 4.59 (s, 1 H), 4.54 (s, 1 H), 3.94 (d, *J* = 11.2 Hz, 1 H), 3.53 (d, *J* = 11.2 Hz, 1 H), 2.72-2.66 (m, 1 H), 2.52-2.48 (m, 1 H), 2.37 (s, 1 H), 2.30 (td, *J* = 13.7, 4.9 Hz, 1 H), 2.16-2.13 (m, 1 H), 1.97-1.89 (m, 3 H), 1.68-1.66 (m, 1 H), 1.58-1.50 (m, 2 H), 1.27-1.22 (m, 1 H), 1.18 (s, 3 H), 1.14 (s, 3 H) ppm; ^{13}C NMR (151 MHz, $CDCl_3$) δ = 217.1, 157.4, 104.6, 65.6, 54.8, 53.1, 39.3, 35.5, 35.3, 32.8, 28.4, 23.1, 21.2, 20.2 ppm; HRMS (ESI) calcd for $C_{14}H_{23}O_2^+$ $[M + H]^+$ 223.1693, found 223.1693.

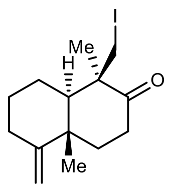

40

Iodide 40. To a solution of alcohol **SI-11** (1.93 g, 8.68 mmol, 1.0 equiv) in benzene (60 mL) was added PPh_3 (2.96 g, 11.3 mmol, 1.3 equiv), imidazole (1.18 g, 17.4 mmol, 2.0 equiv) and I_2 (2.64 g, 10.4 mmol 1.2 equiv) at 0 °C. The reaction mixture was warmed to 40 °C and stirred at that temperature for 15 h before it was cooled to 0 °C. The reaction contents were quenched with EtOAc (100 mL) and saturated aqueous $NaHCO_3$ (150 mL), and extracted with EtOAc (3 × 80 mL). The combined organic phases were washed with brine (100 mL), dried ($MgSO_4$), filtered, and concentrated. The resultant residue was purified by flash column chromatography (silica gel, CH_2Cl_2/petroleum ether, 1:50→EtOAc/petroleum ether, 1:30) to give iodide **40** (2.45 g, 85% yield) as a white solid. **40**: R_f = 0.55 (silica gel, EtOAc/petroleum ether, 1:10); IR (film) ν_{max} = 3085, 2934, 2857, 1712, 1636, 1598, 1447, 1378, 1339, 1311, 1282, 1207, 1192, 1107, 1017, 978, 893, 863, 720, 637 cm^{-1}; ^1H NMR (400 MHz, $CDCl_3$) δ = 4.57 (s, 1 H), 4.51 (s, 1 H), 3.75 (d, *J* = 10.3 Hz, 1 H), 3.19 (d, *J* = 10.3 Hz, 1 H), 2.75 (td, *J* = 14.2, 5.4 Hz, 1 H), 2.41 (dt, *J* = 13.6, 3.5 Hz, 1 H), 2.32 (td, *J* = 13.8, 5.2 Hz, 1 H), 2.16 (dd, *J* = 13.8, 4.0 Hz, 1 H), 2.03-1.87 (m, 3 H), 1.71-1.61 (m, 2 H), 1.57 (dd, *J* = 11.4, 3.7 Hz, 1 H), 1.34 (s, 3 H), 1.31-1.23 (m, 1 H), 1.15 (s, 3 H) ppm; ^{13}C NMR (151 MHz, $CDCl_3$) δ = 211.7, 157.3, 104.2, 55.4, 52.6, 39.8, 37.6, 35.3, 32.6, 28.0, 23.6, 22.8, 20.6, 11.3 ppm; HRMS (ESI) calcd for $C_{14}H_{22}IO^+$ $[M + H]^+$ 333.0710, found 333.0709.

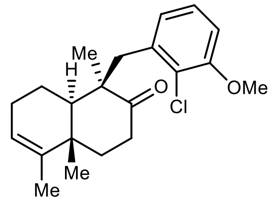

41

Chloride 41. To an flame-dried Schlenk tube containing $NiBr_2$•DME (0.279 g, 0.903 mmol, 1.5 equiv), 4,4'-di-*tert*-butyl-2,2'-bipyridine (**28**) (0.242 g, 0.903 mmol, 1.5 equiv), Mn (99.1 mg, 1.81 mmol, 3.0 equiv.) and 1-bromo-2-chloro-3-methoxybenzene (**32**) (0.400 g, 1.81 mmol, 3.0 equiv) was added a solution of iodide **40** (0.200 g, 0.602 mmol, 1.0 equiv) in DMAc (3.0 mL) at 23 °C. The reaction mixture was frozen in a liquid nitrogen bath, vacuumed and refilled with argon for three times. The resultant reaction was heated to 50 °C and stirred at that temperature for 17 h before it was quenched with water (20 mL), extracted with EtOAc (3 × 20 mL). The combined organic phases were washed with brine (30 mL), dried ($MgSO_4$), filtered, and concentrated. The resultant residue was purified by flash column chromatography (silica gel, EtOAc/petroleum ether, 1:50→1:10) to give coupling product along with some minor impurities (83.0 mg combined) as a pale yellow oil. Pressing forward, the coupling product was dissolved in benzene (1.4 mL) and frozen in a liquid nitrogen bath, vacuumed and refilled with argon for three times. To this solution was added a degassed solution of Co($Sal^{t\text{-Bu},\, t\text{-Bu}}$)Cl (7.7 mg, 12.0 μmol, 0.05 eq) in benzene (1.0 mL) and phenylsilane (5.9 μL, 47.9 μmol, 0.2 equiv) at 23 °C. The reaction mixture was rapidly formed a clear red-orange solution and stirred at 23 °C for 24 h before it was quenched with saturated aqueous NH_4Cl (10 mL) and extracted with EtOAc (3 × 10 mL). The combined organic phases were washed with brine (20 mL), dried ($MgSO_4$), filtered, and concentrated. The resultant residue was purified by flash column chromatography (silica gel, EtOAc/petroleum ether, 1:30→1:20) to give

chloride **41** (43.9 mg, 21% yield for two steps) as a white solid. **41**: R_f = 0.34 (silica gel, EtOAc/petroleum ether, 1:10); IR (film) ν_{max} = 3444, 2955, 2926, 2870, 2855, 1704, 1596, 1576, 1471, 1435, 1378, 1288, 1267, 1112, 1091, 1070, 1046, 1018, 799, 782, 770, 722 cm^{-1}; ^1H NMR (400 MHz, $CDCl_3$) δ = 7.08 (t, *J* = 8.0 Hz, 1 H), 6.77 (dd, *J* = 8.2, 1.2 Hz, 1 H), 6.53 (dd, *J* = 7.7, 1.3 Hz, 1 H), 5.28 (s, 1 H), 3.86 (s, 3 H), 3.24-3.11 (m, 3 H), 2.43 (ddd, *J* = 14.7, 4.1, 2.8 Hz, 1 H), 2.23-2.06 (m, 3 H), 1.91-1.85 (m, 2 H), 1.72-1.58 (m, 5 H), 1.40 (s, 3 H), 0.93 (s, 3 H) ppm; ^{13}C NMR (101 MHz, $CDCl_3$) δ = 214.8, 155.1, 142.2, 137.7, 126.4, 123.8, 123.4, 121.7, 110.0, 56.3, 54.7, 53.2, 38.0, 37.1, 35.9, 35.8, 27.3, 20.5, 20.1, 19.7, 18.3 ppm; HRMS (ESI) calcd for $C_{21}H_{28}ClO_2^+$ $[M + H]^+$ 347.1772, found 347.1771.

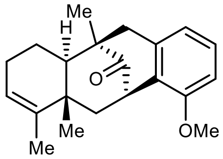

SI-12

Methoxybenzene SI-12. To a solution of chloride **41** (0.150 g, 0.432 mmol, 1.0 equiv) in 1,4-dioxane (8.6 mL) was sequentially added NHC-Pd(II)-Im (28.1 mg, 43.2 µmol, 0.1 equiv) and *t*-BuONa (0.125 g, 1.30 mmol, 3.0 equiv) at 25 °C. The resultant mixture was then frozen in a liquid nitrogen bath, vacuumed and refilled with argon for three times. The reaction mixture was heated to 110 °C and stirred at that temperature for 6 h before it was cooled to 23 °C. The reaction contents were quenched with saturated aqueous NH_4Cl (20 mL), and extracted with EtOAc (3 × 20 mL). The combined organic phases were washed with brine (40 mL), dried ($MgSO_4$), filtered, and concentrated. The resultant residue was purified by flash column chromatography (silica gel, EtOAc/petroleum ether, 1:40) to give methoxybenzene **SI-12** (87.3 mg, 65% yield) as a white solid. **SI-12**: R_f = 0.55 (silica gel, EtOAc/petroleum ether, 1:10); IR (film) ν_{max} = 3426, 2956, 2925, 2870, 2853, 1723, 1588, 1460, 1378, 1263, 1253, 1123, 1097, 1017, 852, 780, 731 cm^{-1}; ^1H NMR (400 MHz, $CDCl_3$) δ = 7.12 (t, *J* = 7.9 Hz, 1 H), 6.68 (d, *J* = 8.1 Hz, 1 H), 6.61 (d, *J* = 7.7 Hz, 1 H), 5.02 (s, 1 H), 3.87 (dd, *J* = 4.6, 2.9 Hz, 1 H), 3.82 (s, 3 H), 3.50 (d, *J* = 18.1 Hz, 1 H), 2.87 (d, *J* = 18.1 Hz, 1 H), 2.29 (dd, *J* = 13.5, 2.8 Hz, 1 H), 2.14-2.05 (m, 2 H), 2.00 (dd, *J* = 13.4, 4.8 Hz, 1 H), 1.82-1.72 (m, 3 H), 1.57 (d, *J* = 1.0 Hz, 3 H), 1.17 (s, 3 H), 0.78 (s, 3 H) ppm; ^{13}C NMR (151 MHz, $CDCl_3$) δ = 218.0, 156.6, 145.4, 137.4, 128.2, 127.6, 119.4, 119.0, 107.8, 55.8, 55.6, 47.6, 44.9, 43.8, 39.7, 37.5, 26.3, 23.1, 21.5, 18.6, 18.4 ppm; HRMS (ESI) calcd for $C_{21}H_{27}O_2^+$ $[M + H]^+$ 311.2006, found 311.2005.

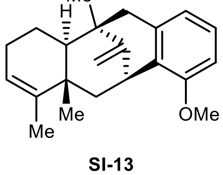

SI-13

Alkene SI-13. To a solution of PPh_3CH_3Br (1.61 g, 4.51 mmol, 10.0 equiv) in toluene (35 mL) was added *t*-BuOK (0.506 g, 4.51 mmol, 10.0 equiv) at 23 °C. The resultant mixture was heated to 90 °C and stirred at that temperature for 1 h before it was cooled to 23 °C. To this reaction mixture was added a solution of methoxybenzene **SI-12** (0.140 g, 0.451 mmol 1.0 equiv) in toluene (10 mL). The resultant reaction mixture was heated to 120 °C and stirred at that temperature for 8 h before it was cooled to 23 °C. The reaction contents were quenched with saturated aqueous NH_4Cl (50 mL) and extracted with EtOAc (3 × 50 mL). The combined organic phases were washed with brine (80 mL), dried ($MgSO_4$), filtered, and

concentrated. The resultant residue was purified by flash column chromatography (silica gel, CH_2Cl_2/ petroleum ether, 1:50→EtOAc/petroleum ether, 1:50) to give alkene **SI-13** (126.6 mg, 91% yield) as a white solid. **SI-13**: R_f = 0.60 (silica gel, EtOAc/petroleum ether, 1:50); IR (film) ν_{max} = 3443, 3083, 2956, 2926, 2872, 2853, 1648, 1601, 1588, 1470, 1439, 1380, 1263, 1253, 1243, 1210, 1111, 1078, 1051, 890, 851, 778, 757, 728 cm^{-1}; ^1H NMR (400 MHz, $CDCl_3$) δ = 7.06 (t, *J* = 7.9 Hz, 1 H), 6.65 (d, *J* = 8.1 Hz, 1 H), 6.60 (d, *J* = 7.6 Hz, 1 H), 4.99 (s, 1 H), 4.84 (d, *J* = 1.3 Hz, 1 H), 4.71 (d, *J* = 1.5 Hz, 1 H), 3.96 (dd, *J* = 4.6, 2.8 Hz, 1 H), 3.85 (s, 3 H), 3.10 (d, *J* = 18.0 Hz, 1 H), 2.64 (d, *J* = 18.0 Hz, 1 H), 2.14-2.09 (m, 3 H), 1.86-1.76 (m, 2 H), 1.68-1.62 (m, 2 H), 1.56 (q, *J* = 2.0 Hz, 3 H), 1.24 (s, 3 H), 0.61 (s, 3 H) ppm; ^{13}C NMR (101 MHz, $CDCl_3$) δ = 157.7, 156.1, 146.8, 139.3, 131.0, 126.5, 119.9, 118.4, 107.2, 102.0, 55.5, 55.2, 43.6, 39.9, 39.0, 38.9, 37.8, 26.9, 26.5, 21.9, 18.7, 18.4 ppm; HRMS (ESI) calcd for $C_{22}H_{29}O^+$ [M + H]$^+$ 309.2213, found 309.2213.

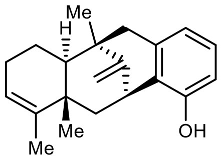

SI-14

Phenol SI-14. To a solution of *n*-BuSH (0.307 g, 3.40 mmol, 7.5 equiv) in HMPA (6.0 mL) was added *n*-BuLi (1.42 mL, 2.4 M in pentane, 3.40 mmol, 7.5 equiv) dropwise at 0 °C. The resultant mixture was stirred at 0 °C for 30 min before a solution of alkene **SI-13** (0.140 g, 0.454 mmol, 1.0 equiv) was added. The resultant reaction contents were stirred at 0 °C for 10 min and allowed to heated to110 °C. Upon completion, the reaction mixture was cooled to 23 °C and quenched with saturated aqueous NH_4Cl (30 mL), and extracted with Et_2O (3 × 30 mL). The combined organic layer was washed with brine (3 × 50 mL), dried ($MgSO_4$), filtered, and concentrated. The resultant residue was purified by flash column chromatography (silica gel, EtOAc/petroleum ether, 1:30→1:10) to give phenol **SI-14** (0.119 g, 89% yield) as a colorless oil. **SI-14**: R_f = 0.35 (silica gel, EtOAc/petroleum ether, 1:10); IR (film) ν_{max} = 3407, 3082, 3020, 2960, 2926, 2853, 1648, 1588, 1465, 1436, 1381, 1374, 1306, 1271, 1255, 1203, 1094, 1007, 979, 891, 778, 760, 728, 706 cm^{-1}; ^1H NMR (600 MHz, $CDCl_3$) δ = 6.97 (t, *J* = 7.7 Hz, 1 H), 6.59 (d, *J* = 7.6 Hz, 1 H), 6.56 (d, *J* = 7.8 Hz, 1 H), 5.00 (s, 1 H), 4.86 (s, 1 H), 4.74 (s, 1 H), 3.90 (dd, *J* = 4.4, 2.9 Hz, 1 H), 3.10 (d, *J* = 18.0 Hz, 1 H), 2.65 (d, *J* = 18.0 Hz, 1 H), 2.17 (dd, *J* = 12.9, 2.7 Hz, 1 H), 2.10-2.07 (m, 2 H), 1.86-1.81 (m, 2 H), 1.68-1.62 (m, 2 H), 1.56 (d, *J* = 1.6 Hz, 3 H), 1.25 (s, 3 H), 0.64 (s, 3 H) ppm; ^{13}C NMR (151 MHz, $CDCl_3$) δ = 157.2, 152.0, 146.6, 139.9, 129.2, 126.6, 120.2, 118.5, 112.1, 102.3, 55.1, 43.5, 40.1, 39.0, 38.8, 37.8, 26.8, 26.5, 21.9, 18.6, 18.4 ppm; HRMS (ESI) calcd for $C_{21}H_{27}O^+$ [M + H]$^+$ 295.2056, found 295.2056.

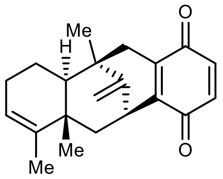

42

Quinone 42. To a solution of phenol **SI-14** (130.0 mg, 0.442 mmol, 1.0 equiv) in MeCN (18 mL) was added salcomine (0.431 g, 1.32 mmol, 3.0 equiv) at 23 °C under oxygen. The reaction mixture was stirred at that temperature for 3 h under a balloon of oxygen. The resultant reaction contents were quenched with saturated aqueous NH_4Cl (40 mL), and extracted with EtOAc (3 × 40 mL). The combined organic

phases were washed with brine (60 mL), dried ($MgSO_4$), filtered, and concentrated. The resultant residue was purified by flash column chromatography (silica gel, EtOAc/petroleum ether, 1:20→1:10) to give quinone **42** (0.113 g, 83%) as a yellow solid. **42**: R_f = 0.60 (silica gel, EtOAc/petroleum ether, 1:10); IR (film) ν_{max} = 2956, 2926, 2855, 1653, 1600, 1460, 1376, 1298, 1118, 1093, 1025, 1013, 896, 845, 804 cm^{-1}; ^1H NMR (400 MHz, $CDCl_3$) δ = 6.72 (d, *J* = 10.1 Hz, 1 H), 6.67 (d, *J* = 10.1 Hz, 1 H), 4.99 (s, 1 H), 4.81 (s, 1 H), 4.73 (s, 1 H), 3.66-3.65 (m, 1 H), 2.82 (d, *J* = 21.0 Hz, 1 H), 2.24 (d, *J* = 21.0 Hz, 1 H), 2.07-2.03 (m, 3 H), 1.86-1.80 (m, 2 H), 1.63-1.57 (m, 2 H), 1.53 (d, *J* = 1.4 Hz, 3 H), 1.24 (s, 3 H), 0.95 (s, 3 H) ppm; ^{13}C NMR (101 MHz, $CDCl_3$) δ = 187.0, 186.4, 154.7, 146.5, 146.2, 144.0, 136.6, 136.5, 118.8, 103.4, 55.0, 43.3, 39.3, 38.5, 37.9, 34.0, 26.3, 26.3, 23.1, 18.5, 18.4 ppm; HRMS (ESI) calcd for $C_{21}H_{25}O_2^+$ $[M + H]^+$ 309.1849, found 309.1843.

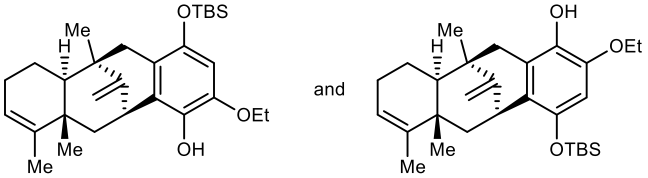

43 **44**

Mono silyl ethers 43 and 44. To an flame-dried Schlenk tube containing $Co(OAc)_2$ (17.2 mg, 97.3 µmol, 3.0 equiv) was added a solution of quinone **42** (10.0 mg, 32.4 µmol, 1.0 equiv) in EtOH (2.0 mL) at 23 °C. The resultant mixture was purged with oxygen. The reaction mixture was heated to 160 °C and stirred at that temperature for 10 h before it was concentrated. The resultant residue was purified by flash column chromatography (silica gel, EtOAc/petroleum ether, 1:50→1:20) to recover quinone **42** (4.0 mg) and afford the mixture of inseparable quinones (4.8 mg, 42% yield, 70% yield brsm, rr = 1:1) as a yellow oil which contain the proposed structure of dysidavarone D (**10**). Pressing forward, a mixture of quinones (30.0 mg, 85.1 µmol, 1.0 equiv, rr = 1:1) was dissolved in THF (5.0 mL). To this solution was added saturated aqueous $Na_2S_2O_4$ (10 mL) at 23 °C and stirred at that temperature for 1 h. Upon completion, the reaction contents were extracted with EtOAc (3 × 10 mL). The combined organic phases were washed with brine (20 mL), dried ($MgSO_4$), filtered, and concentrated. Pressing forward without any further purification, the resultant residue was dissolved in CH_2Cl_2 (1.0 mL). To this solution was added imidazole (29.0 mg, 0.426 mmol, 5.0 equiv) and TBSCl (38.5 mg, 0.255 mmol, 3.0 equiv) sequentially at -10 °C. The reaction mixture was stirred at that temperature for 10 min before it was quenched with H_2O (5.0 mL), and extracted with CH_2Cl_2 (3 × 10 mL). The combined organic phases were washed with brine (20 mL), dried ($MgSO_4$), filtered, and concentrated. The resultant residue was purified by flash column chromatography (silica gel, EtOAc/petroleum ether, 1:40→1:5) to give mono silyl ether **43** (14.8 mg, 37% yield) as a colorless oil and mono silyl ether **44** (10.4 mg, 26% yield) as a colorless oil. **43**: R_f = 0.58 (silica gel, EtOAc/petroleum ether, 1:10); IR (film) ν_{max} = 3557, 2959, 2930, 2898, 2858, 1491, 1473, 1345, 1253, 1231, 1131, 1107, 1059, 839, 779 cm^{-1}; ^1H NMR (600 MHz, $CDCl_3$) δ = 6.21 (s, 1 H), 5.30 (s, 1 H), 4.98 (s, 1 H), 4.82 (s, 1 H), 4.69 (s, 1 H), 4.04-3.98 (m, 2 H), 3.94 (dd, *J* = 4.3, 2.9 Hz, 1 H), 2.87 (d, *J* = 18.1 Hz, 1 H), 2.34 (d, *J* = 18.1 Hz, 1 H), 2.17 (dd, *J* = 12.9, 2.7 Hz, 1 H), 2.10-2.04 (m, 2 H), 1.83 (dd, *J* = 11.8, 7.6 Hz, 1 H), 1.78 (dd, *J* = 12.9, 4.7 Hz, 1 H), 1.66-1.57 (m, 2 H), 1.55 (s, 3 H), 1.42 (t, *J* = 7.0 Hz, 3 H), 1.23 (s, 3 H), 1.00 (s, 9 H), 0.66 (s, 3 H), 0.16 (s, 3 H), 0.15 (s, 3 H) ppm; ^{13}C NMR (151 MHz, $CDCl_3$) δ = 157.6, 146.8, 145.0, 143.3, 136.3, 129.6, 121.6, 118.4, 102.0, 101.7, 64.7, 55.2, 43.5, 40.4, 38.4, 37.8, 34.5, 27.1, 26.5, 26.0, 21.8, 18.7, 18.4, 18.4, 15.1, -3.8 ppm; HRMS (ESI) calcd for $C_{29}H_{45}O_3Si^+$ $[M + H]^+$ 469.3132, found 469.3121. **44**: R_f = 0.54 (silica gel, EtOAc/petroleum ether, 1:10); IR (film) ν_{max} = 3556, 2958, 2930, 2898, 2858, 1490, 1474, 1259, 1234, 1125, 1107, 1059, 873, 840, 778 cm^{-1}; ^1H NMR (600 MHz, $CDCl_3$) δ = 6.26 (s, 1 H), 5.27 (s, 1 H), 4.98 (s, 1 H), 4.77 (s, 1 H), 4.68 (s, 1

H), 4.04-3.96 (m, 2 H), 3.83-3.82 (m, 1 H), 3.00 (d, *J* = 18.4 Hz, 1 H), 2.46 (d, *J* = 18.4 Hz, 1 H), 2.10-2.03 (m, 3 H), 1.83 (dd, *J* = 12.6, 7.5 Hz, 1 H), 1.70-1.65 (m, 2 H), 1.55 (s, 1 H), 1.53 (d, *J* = 0.8 Hz, 3 H), 1.41 (t, *J* = 7.0 Hz, 3 H), 1.26 (s, 3 H), 1.04 (s, 9 H), 0.64 (s, 3 H), 0.24 (s, 3 H), 0.20 (s, 3H) ppm; ^{13}C NMR (151 MHz, $CDCl_3$) δ = 158.0, 146.7, 144.3, 143.1, 136.8, 126.0, 125.1, 118.5, 101.6, 101.5, 64.7, 55.2, 43.7, 40.3, 38.4, 37.7, 33.8, 26.9, 26.6, 26.0, 21.7, 18.7, 18.4, 18.2, 15.1, -3.7, -3.9 ppm; HRMS (ESI) calcd for $C_{29}H_{45}O_3Si^+$ $[M + H]^+$ 469.3132, found 469.3125.

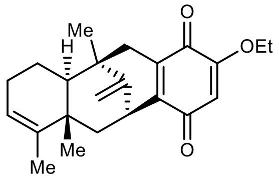

45

Quinone 45. To a solution of mono silyl ether **44** (10.0 mg, 21.3 µmol, 1.0 equiv) in THF (0.3 mL) was added HF·py (0.15 mL) at 23 °C. The resultant reaction mixture was stirred at that temperature for 1 h before it was quenched with saturated aqueous $NaHCO_3$ (20 mL), and extracted with EtOAc (3 × 10 mL). The combined organic phases were washed with brine (20 mL), dried ($MgSO_4$), filtered, and concentrated. Pressing forward without any further purification. The resultant residue was dissolved in CH_2Cl_2 (1.0 mL). To this solution was added MnO_2 (9.3 mg, 0.107 mmol, 5.0 equiv) at 23 °C. The resultant reaction mixture was stirred at that temperature for 15 min before it was concentrated. The resultant residue was purified by flash column chromatography (silica gel, EtOAc/petroleum ether, 1:20) to give quinone **45** (6.7 mg, 89% yield) as a yellow oil. **45**: R_f = 0.35 (silica gel, EtOAc/petroleum ether, 1:10); IR (film) ν_{max} = 2956, 2927, 2855, 1672, 1651, 1635, 1604, 1454, 1374, 1347, 1277, 1230, 1211, 1125, 1036, 898, 852, 795, 755 cm^{-1}; ^{1}H NMR (400 MHz, $CDCl_3$) δ = 5.86 (s, 1 H), 4.99 (s, 1 H), 4.80 (s, 1 H), 4.72 (s, 1 H), 3.98 (q, *J* = 7.0 Hz, 2 H), 3.67 (dd, *J* = 4.3, 2.7 Hz, 1 H), 2.82 (d, *J* = 20.7 Hz, 1 H), 2.23 (d, *J* = 20.7 Hz, 1 H), 2.08-2.02 (m, 3 H), 1.83 (dd, *J* = 13.3, 4.8 Hz, 2 H), 1.63-1.56 (m, 2 H), 1.53 (d, *J* = 1.4 Hz, 3 H), 1.47 (t, *J* = 7.0 Hz, 3 H), 1.24 (s, 3 H), 0.96 (s, 3 H) ppm; ^{13}C NMR (101 MHz, $CDCl_3$) δ = 186.6, 181.7, 157.9, 154.8, 146.9, 146.3, 141.9, 118.8, 107.6, 103.3, 65.2, 55.0, 43.5, 39.4, 38.6, 37.9, 33.7, 26.3, 26.3, 23.2, 18.5, 18.4, 14.0 ppm; HRMS (ESI) calcd for $C_{23}H_{29}O_3^+$ $[M + H]^+$ 353.2111, found 353.2110.

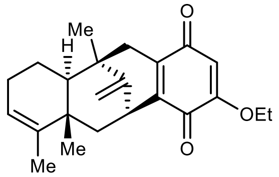

proposed structure of
dysidavarone D (**10**)

Proposed structure of dysidavarone D (10). To a solution of mono silyl ether **43** (10.0 mg, 21.3 µmol, 1.0 equiv) in THF (0.3 mL) was added HF·py(0.15 mL) at 23 °C. The reaction mixture was stirred at that pemperature for 1 h before it was quenched with saturated aqueous $NaHCO_3$ (20 mL), and extracted with EtOAc (3 × 10 mL). The combined organic phases were washed with brine (20 mL), dried ($MgSO_4$), filtered, and concentrated. Pressing forward without any further purification. The resultant residue was dissolved in CH_2Cl_2 (1.0 mL). To this solution was added MnO_2 (9.3 mg, 0.107 mmol, 5.0 equiv) at 23 °C. The resultant mixture was stirred at that temperature for 15 min before it was concentrated. The resultant residue was purified by flash column chromatography (silica gel, EtOAc/petroleum ether, 1:20) to give

proposed structure of dysidavarone D (**10**) (6.5 mg, 87% yield) as a yellow oil. Proposed structure of dysidavarone D (**10**): R_f = 0.35 (silica gel, EtOAc/petroleum ether, 1:10); IR (film) ν_{max} = 2934, 2855, 1670, 1652, 1635, 1603, 1455, 1443, 1386, 1307, 1228, 1211, 1126, 1038, 944, 897, 848, 807, 734 cm^{-1}; ^{1}H NMR (400 MHz, $CDCl_3$) δ = 5.80 (s, 1 H), 4.99 (s, 1 H), 4.80 (s, 1 H), 4.72 (s, 1 H), 3.98 (q, *J* = 7.0 Hz, 2 H), 3.69-3.67 (m, 1 H), 2.83 (d, *J* = 21.2 Hz, 1 H), 2.25 (d, *J* = 21.2 Hz, 1 H), 2.08-2.01 (m, 3 H), 1.81 (dd, *J* = 13.3, 4.9 Hz, 2 H), 1.62-1.56 (m, 2 H), 1.53 (d, *J* = 1.5 Hz, 3 H), 1.48 (t, *J* = 7.0 Hz, 3 H), 1.23 (s, 3 H), 0.96 (s, 3 H) ppm; ^{13}C NMR (101 MHz, $CDCl_3$) δ = 187.2, 181.3, 158.0, 154.9, 146.3, 144.4, 144.3, 118.8, 107.5, 103.2, 65.2, 55.0, 43.0, 39.1, 38.5, 37.9, 34.1, 26.3, 26.2, 23.2, 18.5, 18.3, 14.0 ppm; HRMS (ESI) calcd for $C_{23}H_{29}O_3^+$ $[M + H]^+$ 353.2111, found 353.2111.

II Supplementary Discussion

Supplementary Figure 5. ^{1}H NMR Spectrum of 20 (400 MHz, $CDCl_3$)

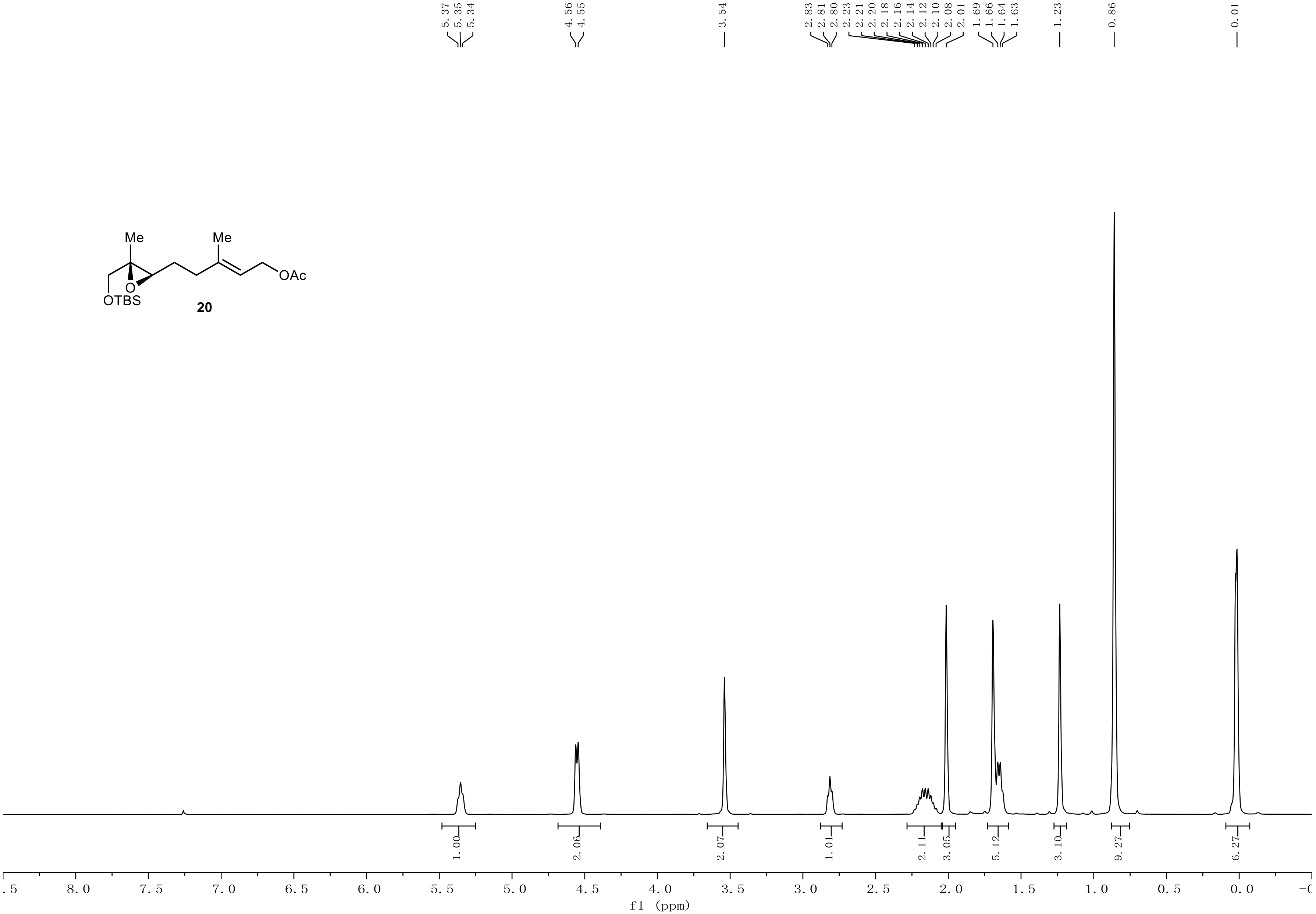

Supplementary Figure 6. ^{13}C NMR Spectrum of 20 (101 MHz, $CDCl_3$)

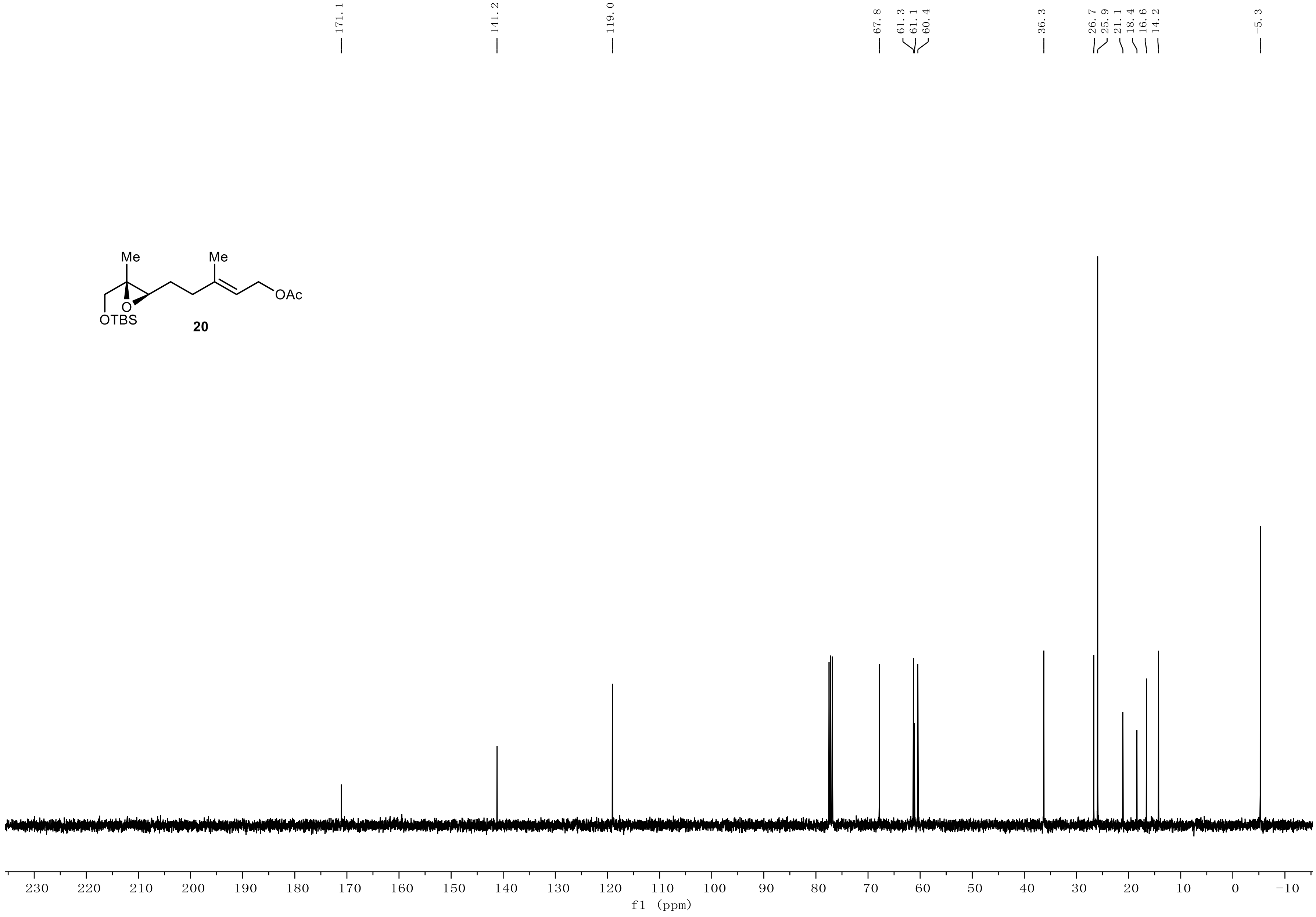

Supplementary Figure 7. ^{1}H NMR Spectrum of 17 (600 MHz, $CDCl_3$)

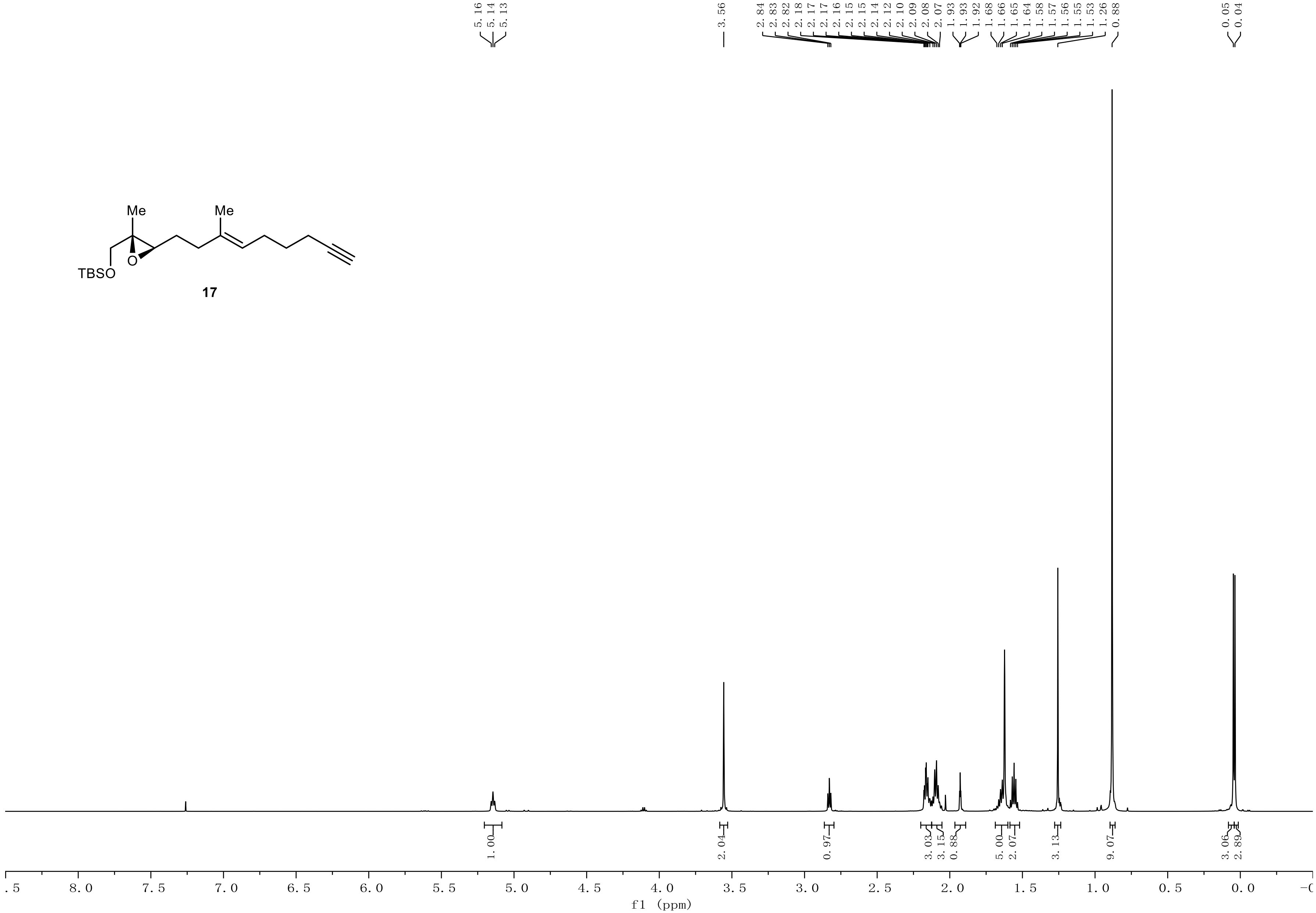

Supplementary Figure 8. ^{13}C NMR Spectrum of 17 (101 MHz, $CDCl_3$)

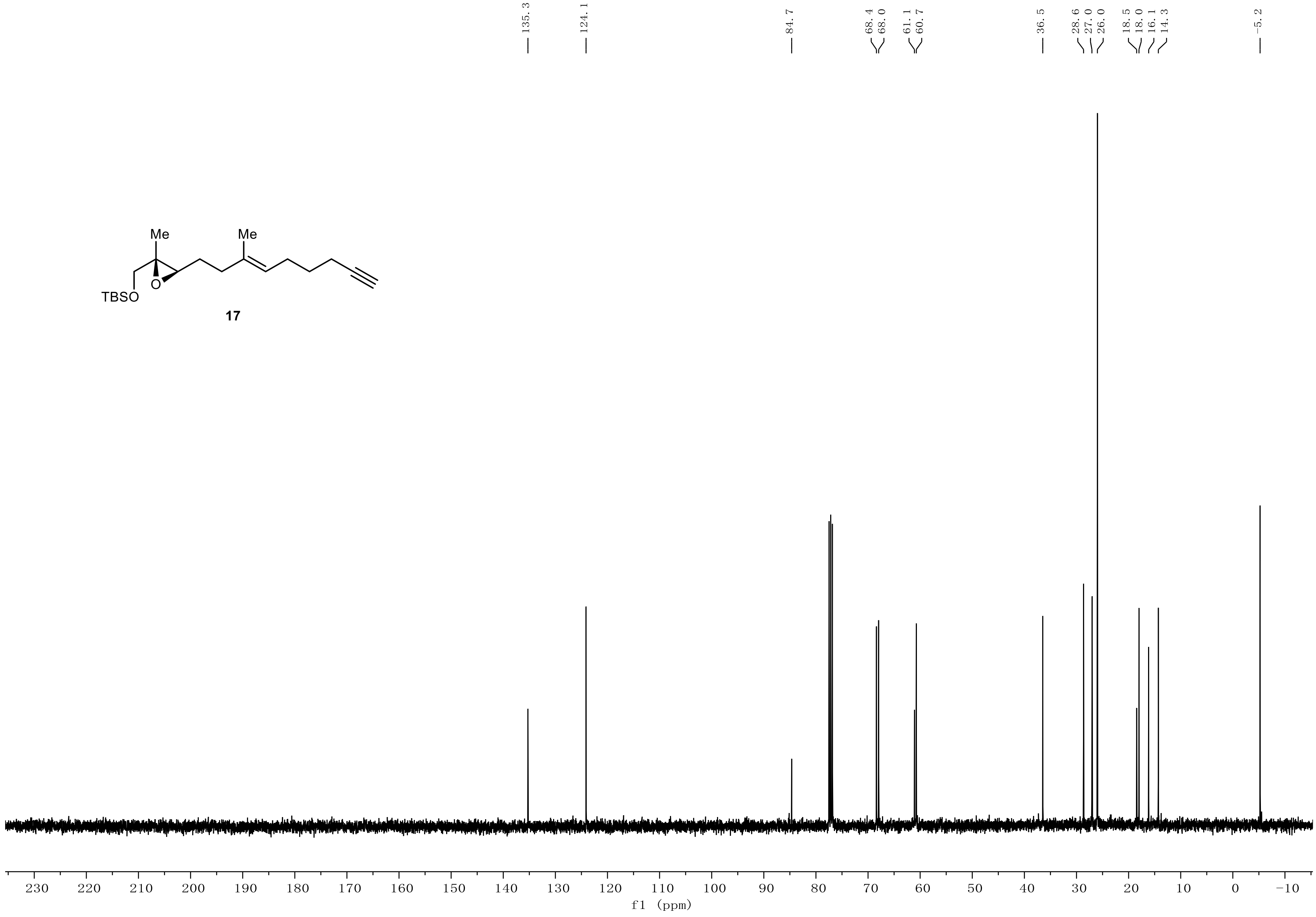

Supplementary Figure 9. ^{1}H NMR Spectrum of 18 (600 MHz, $CDCl_3$)

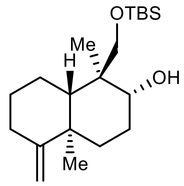

18

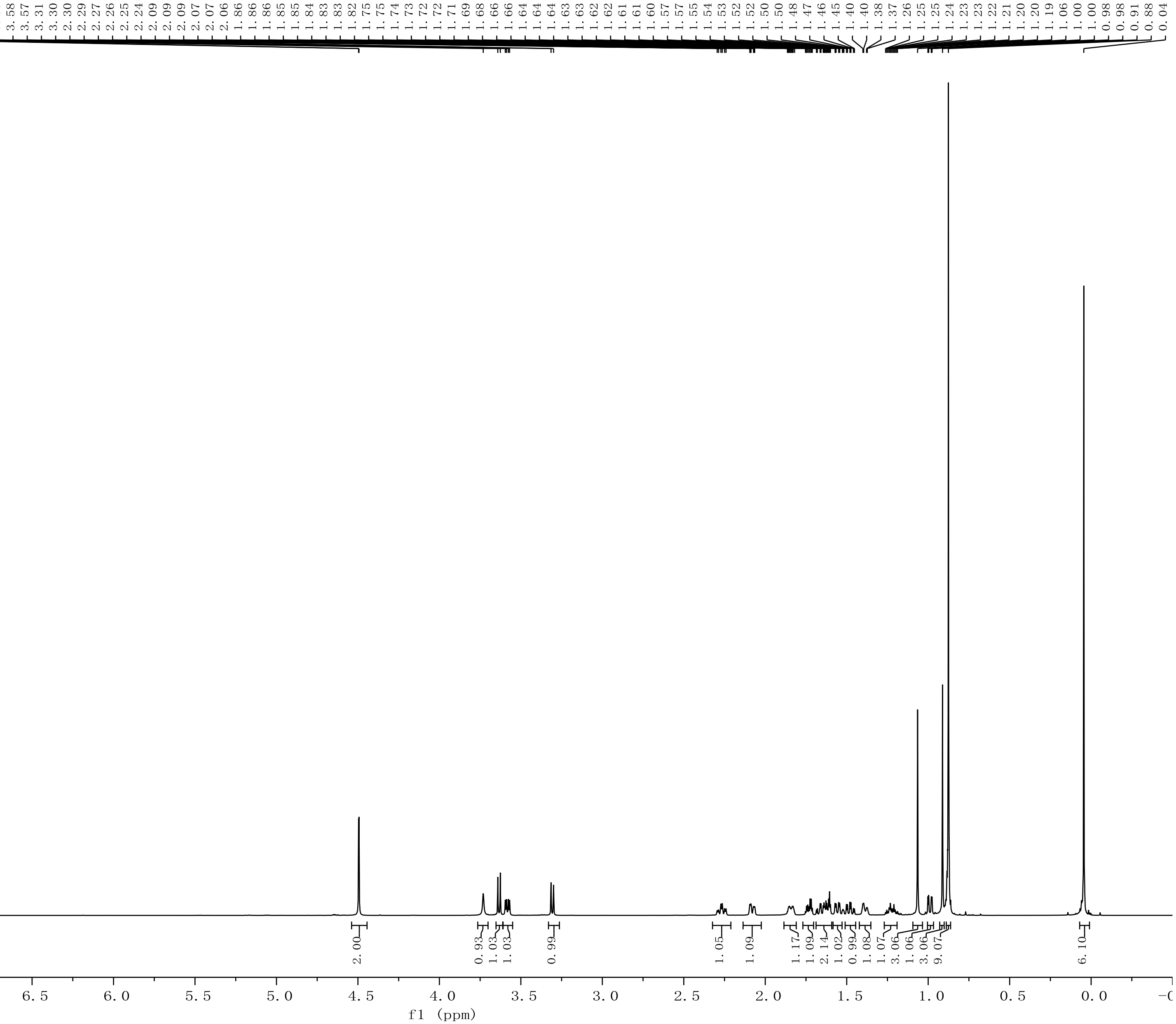

Supplementary Figure 10. ^{13}C NMR Spectrum of 18 (151 MHz, $CDCl_3$)

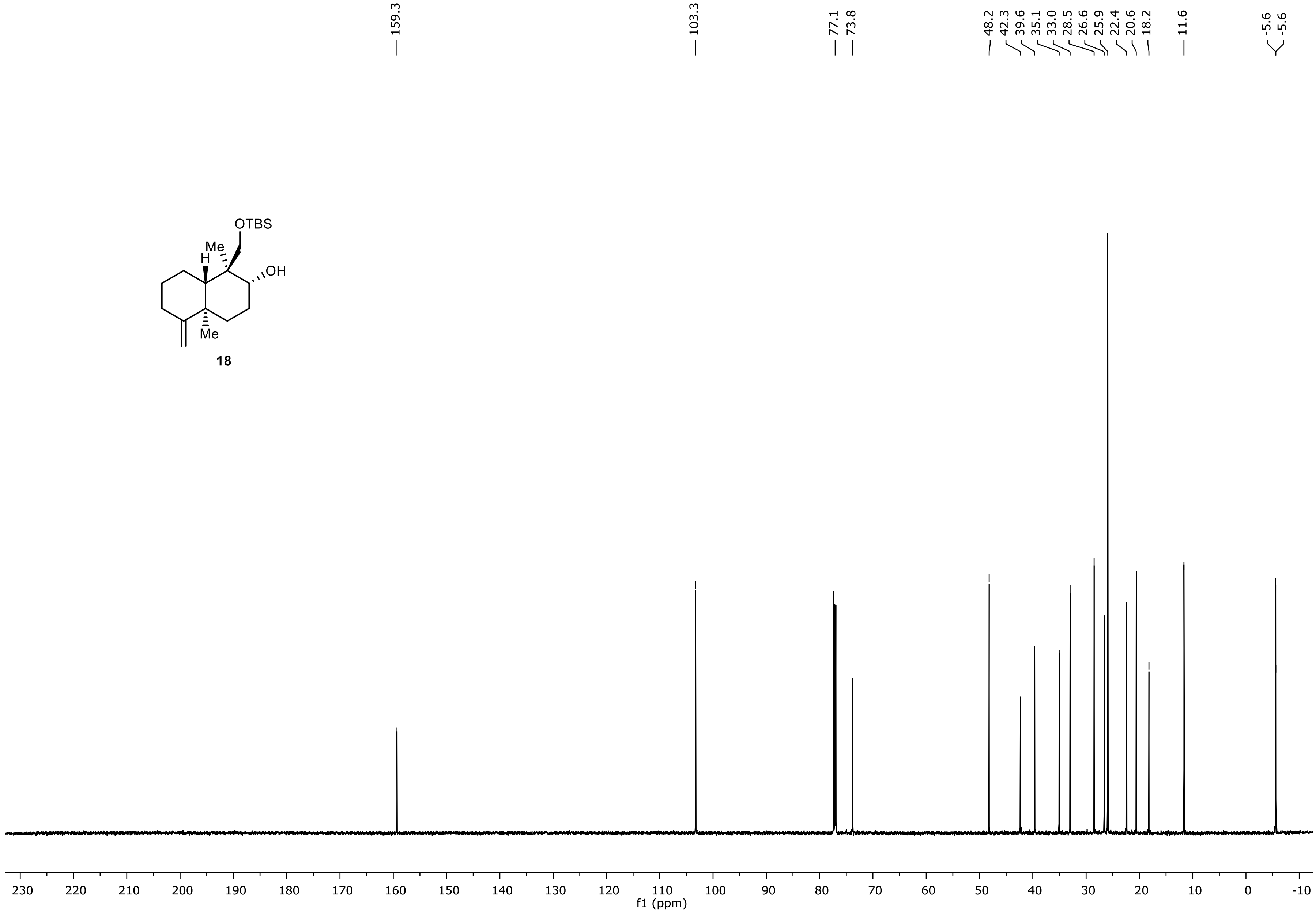

Supplementary Figure 11. ^{1}H NMR Spectrum of SI-4 (400 MHz, $CDCl_3$)

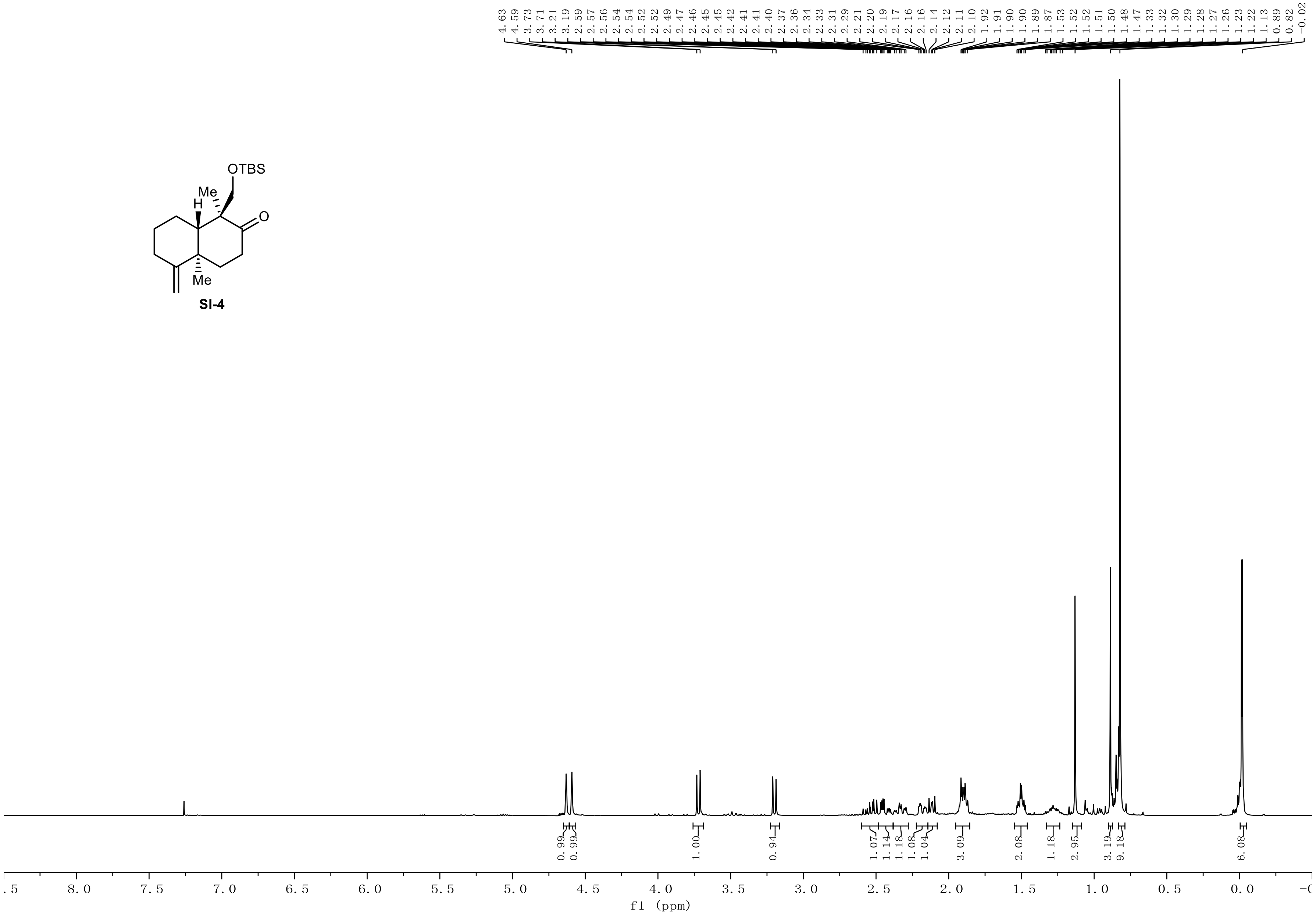

Supplementary Figure 12. ^{13}C NMR Spectrum of SI-4 (101 MHz, $CDCl_3$)

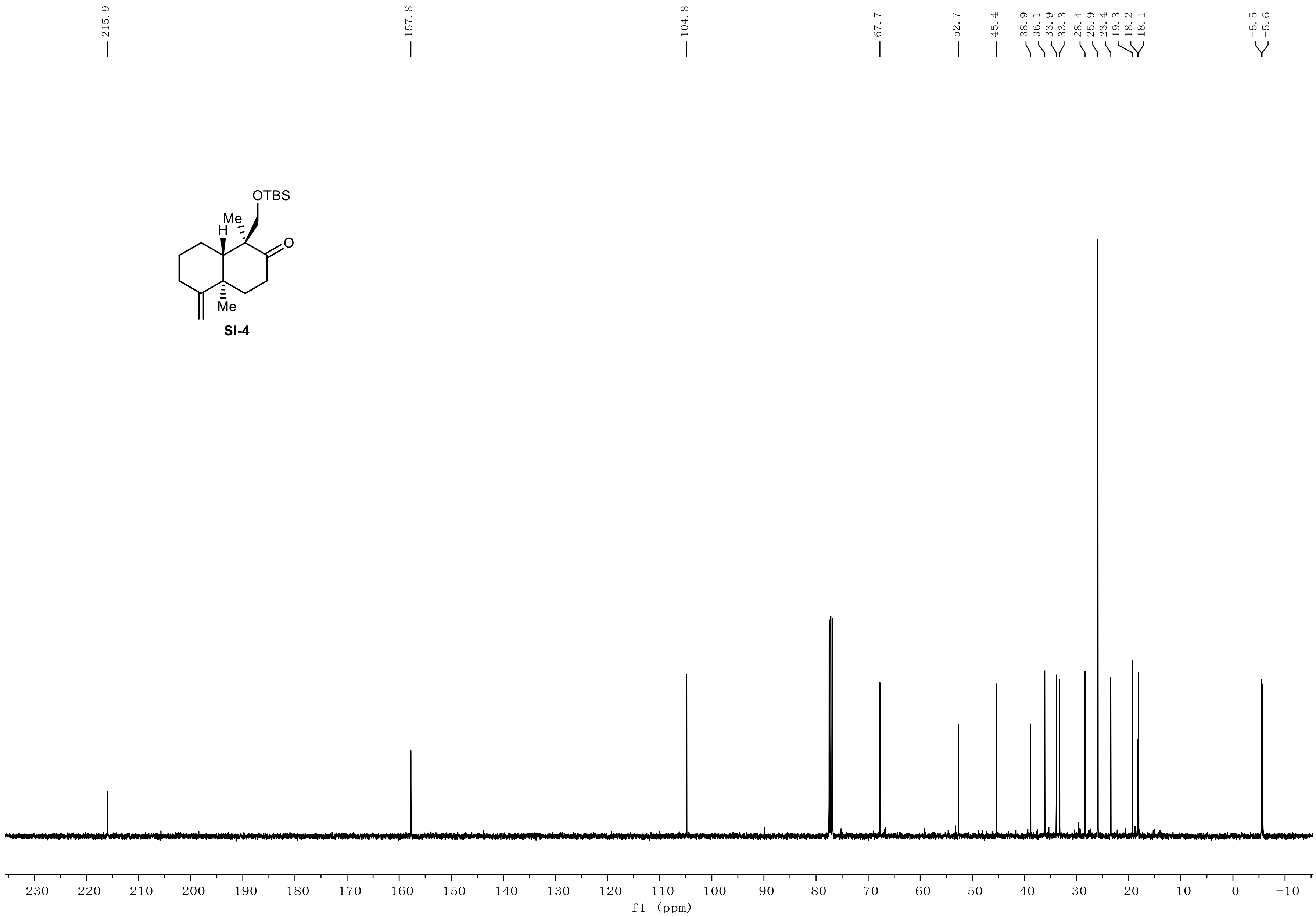

Supplementary Figure 13. ^{1}H NMR Spectrum of 22 (400 MHz, $CDCl_3$)

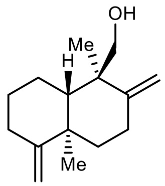

22

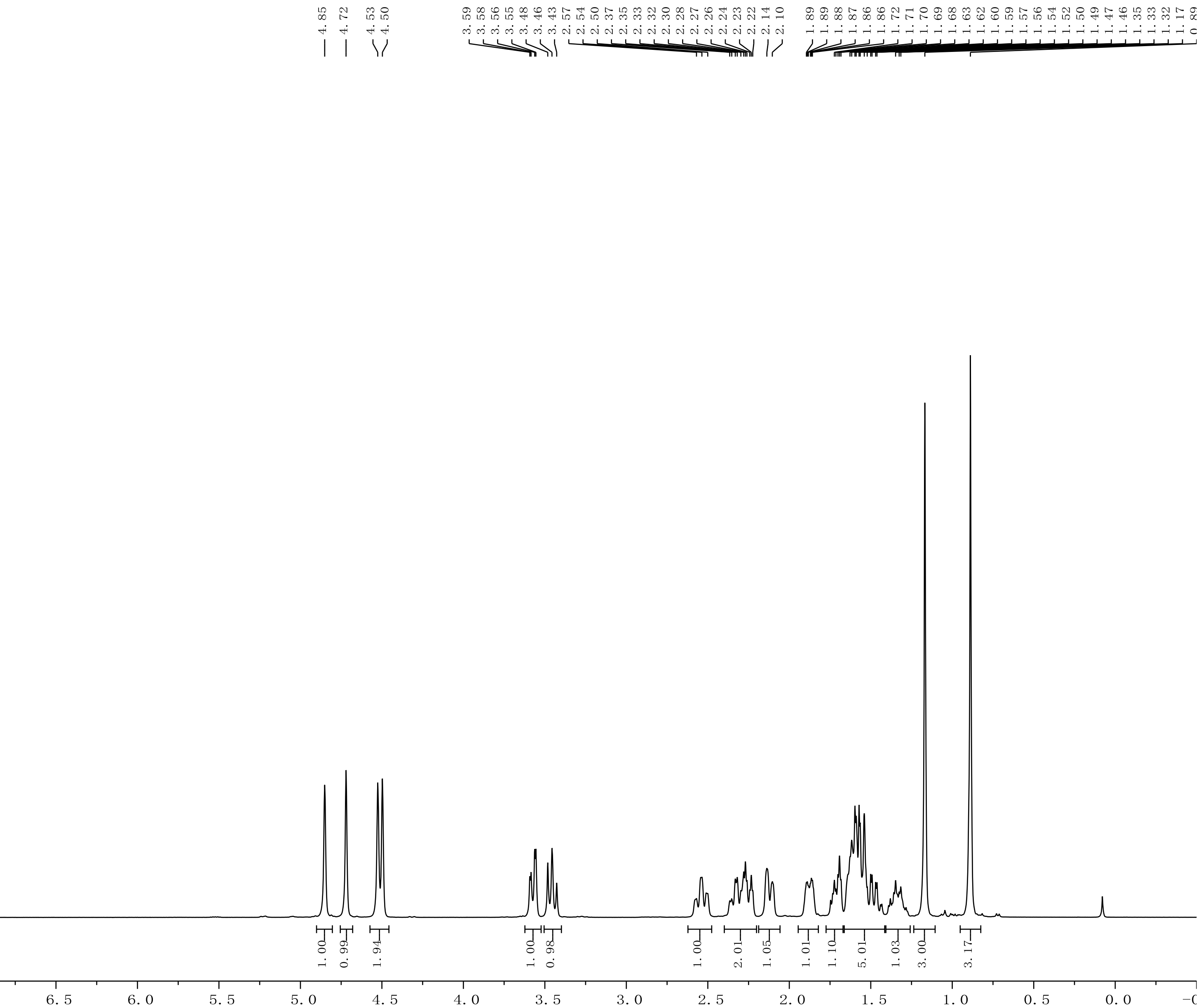

Supplementary Figure 14. ^{13}C NMR Spectrum of 22 (101 MHz, $CDCl_3$)

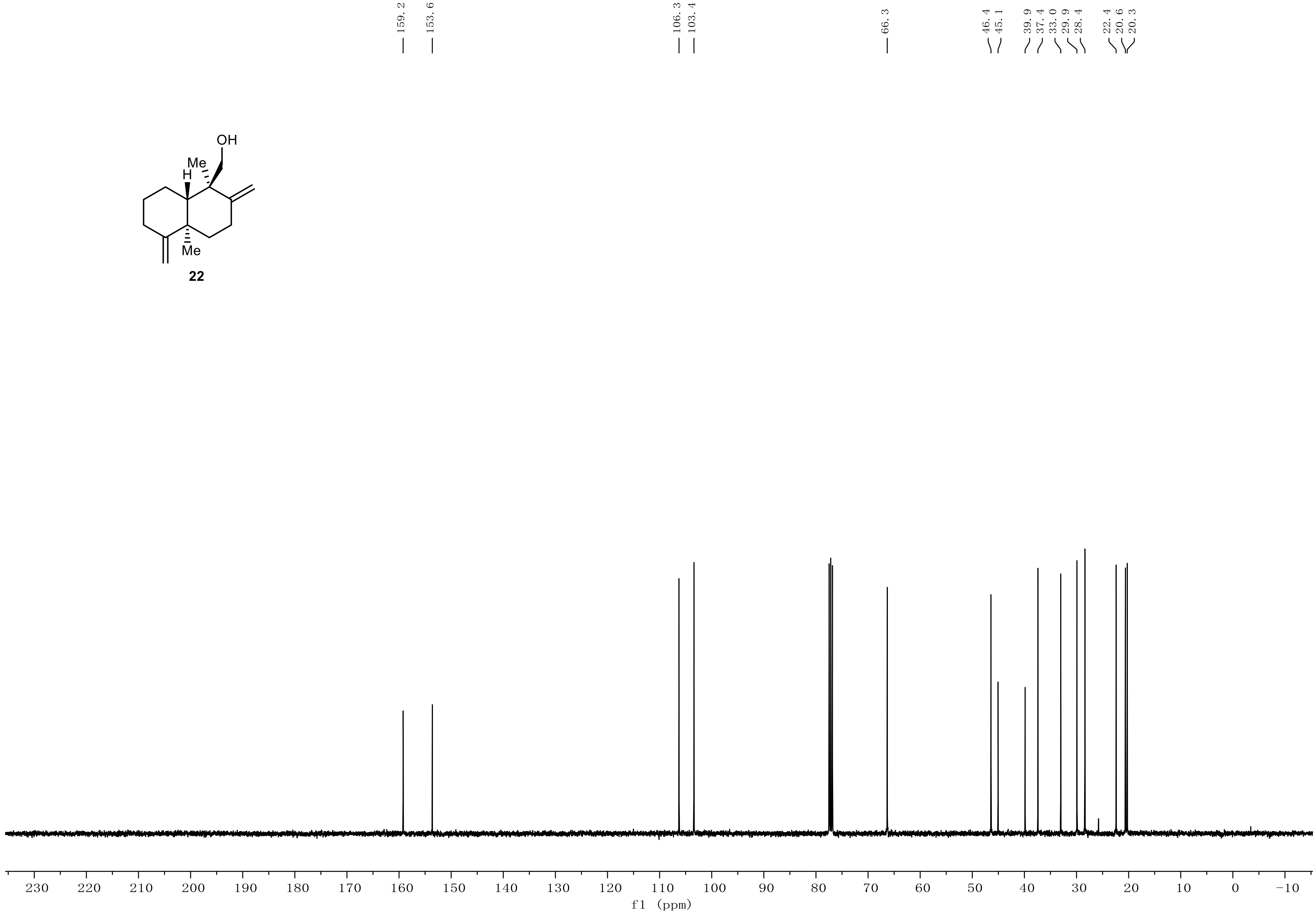

Supplementary Figure 15. ^{1}H NMR Spectrum of SI-6 (400 MHz, $CDCl_3$)

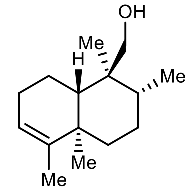

SI-6

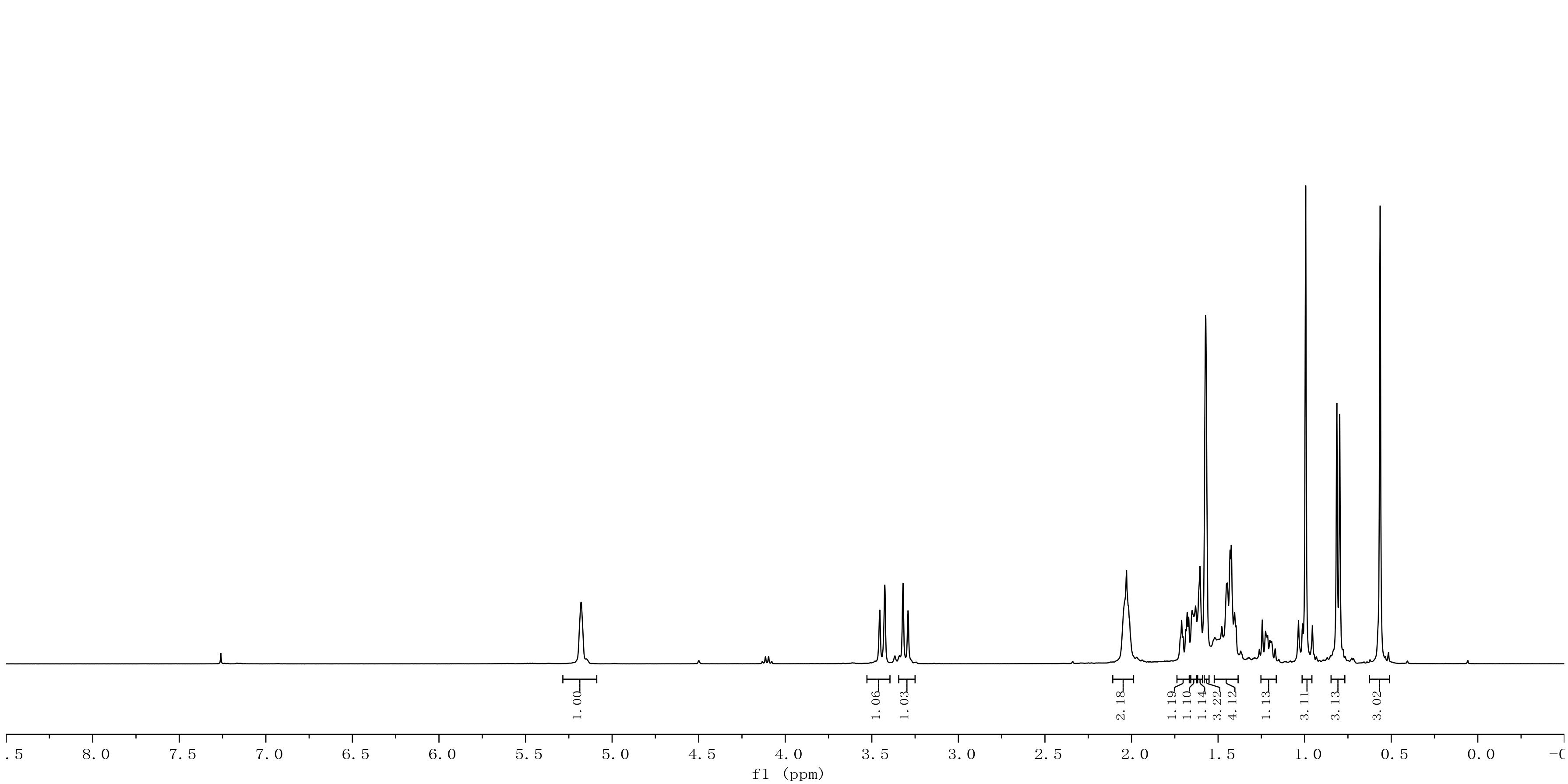

Supplementary Figure 16. ^{13}C NMR Spectrum of SI-6 (101 MHz, $CDCl_3$)

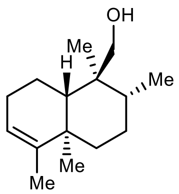

SI-6

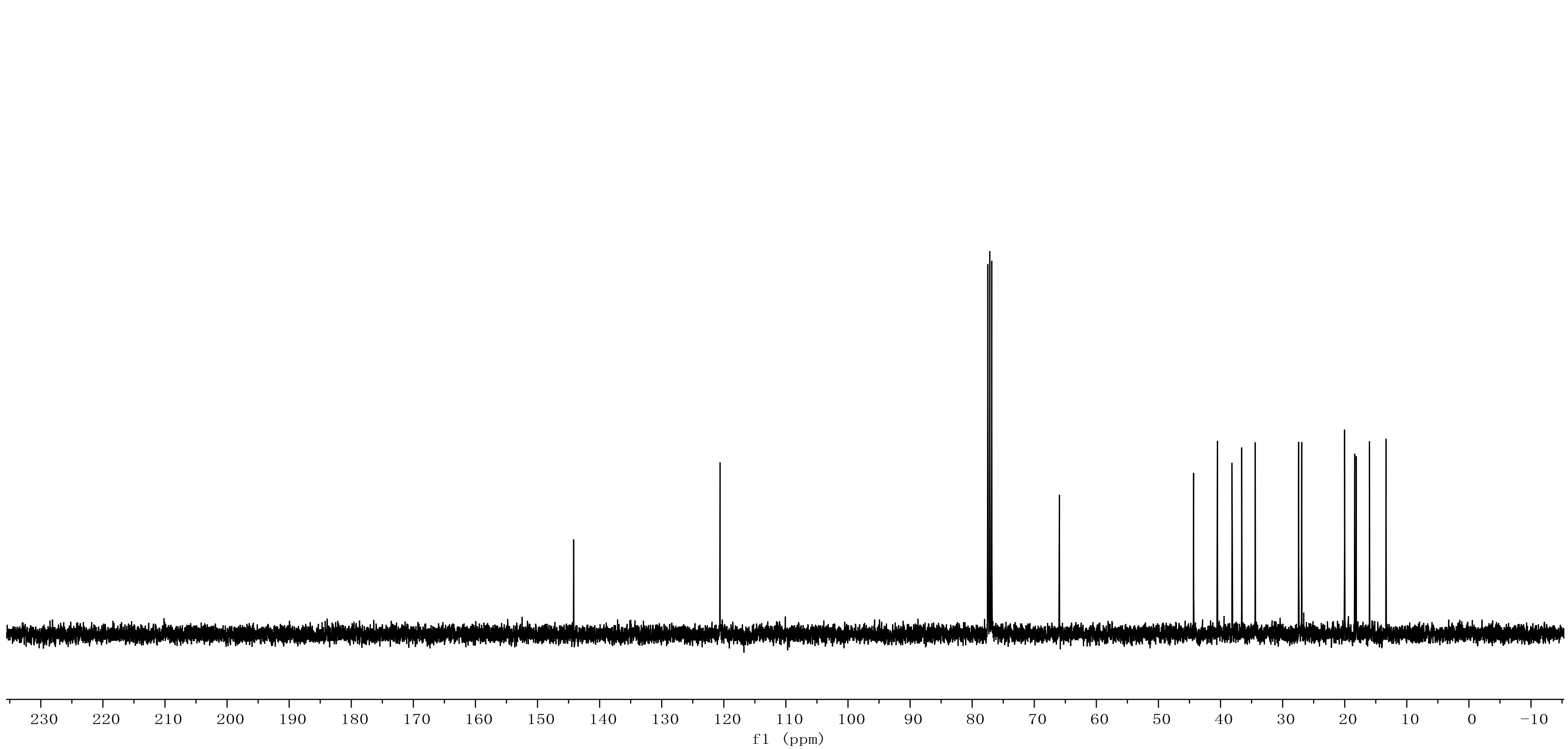

Supplementary Figure 17. ^{1}H NMR Spectrum of 23 (400 MHz, $CDCl_3$)

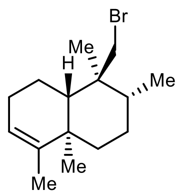

23

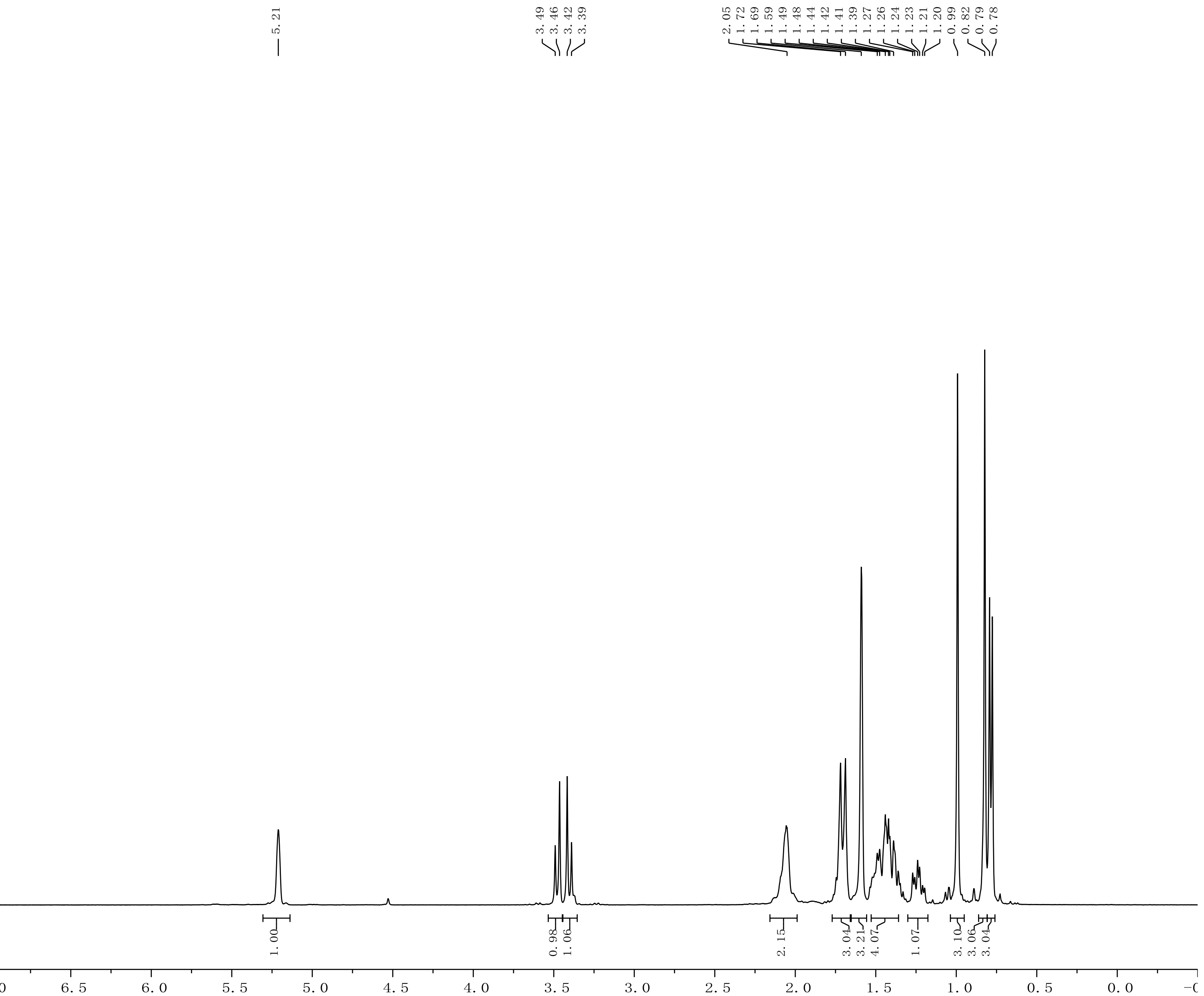

Supplementary Figure 18. ^{13}C NMR Spectrum of 23 (101 MHz, $CDCl_3$)

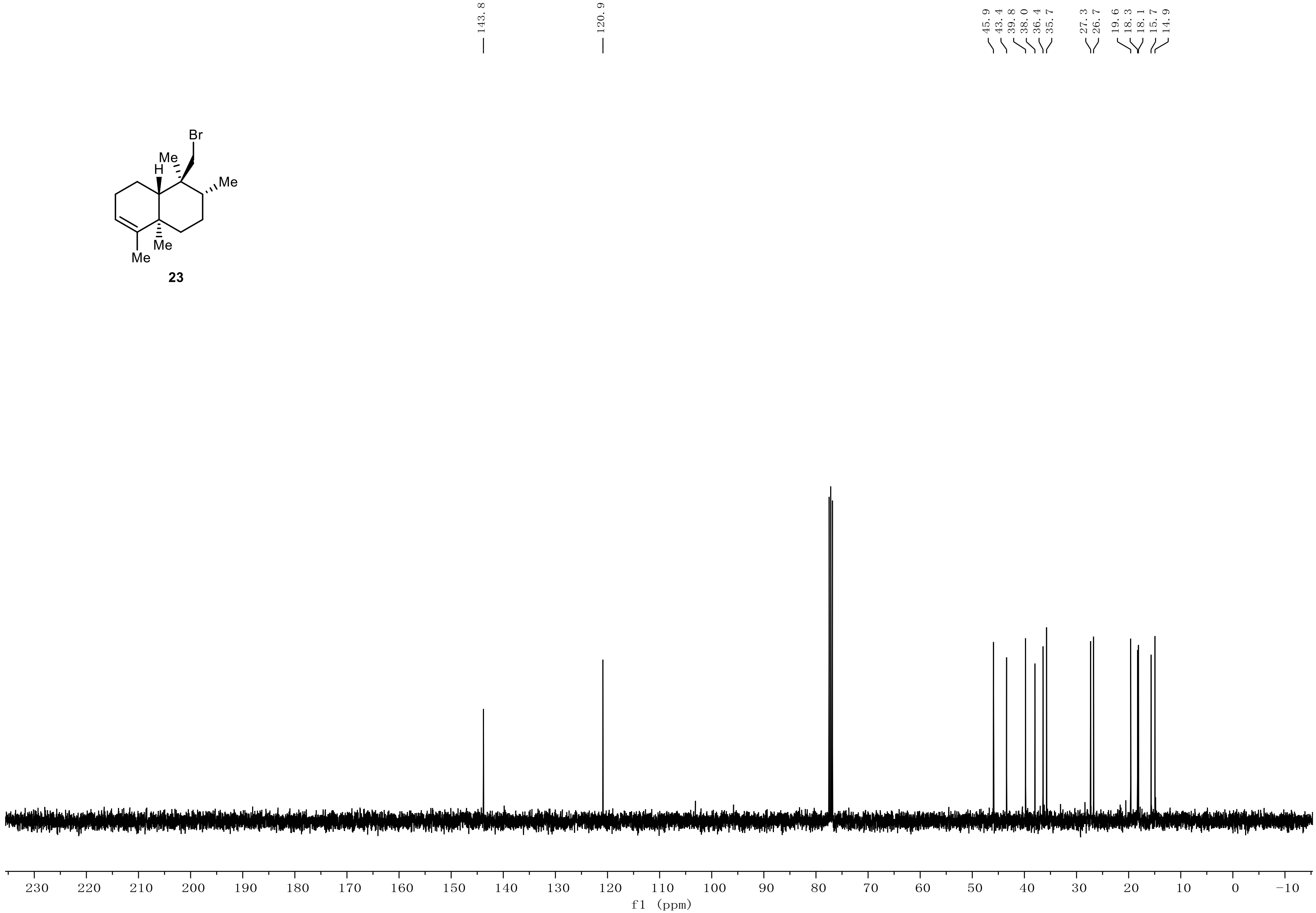

Supplementary Figure 19. ^{1}H NMR Spectrum of Annonene (3) (400 MHz, $CDCl_3$)

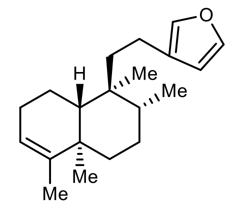

annonene (3)

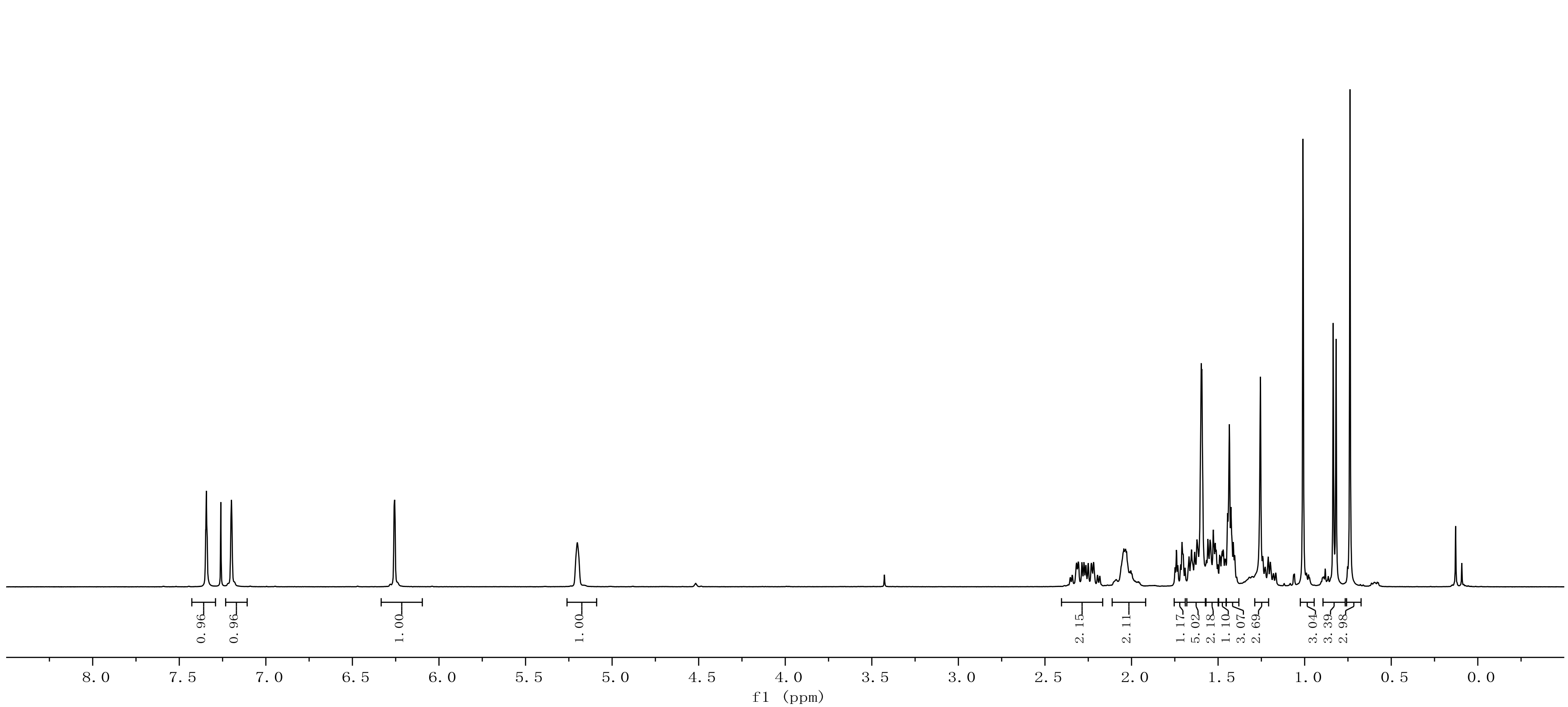

Supplementary Figure 20. ^{13}C NMR Spectrum of Annonene (3) (101 MHz, $CDCl_3$)

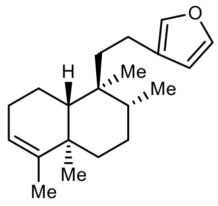

annonene (3)

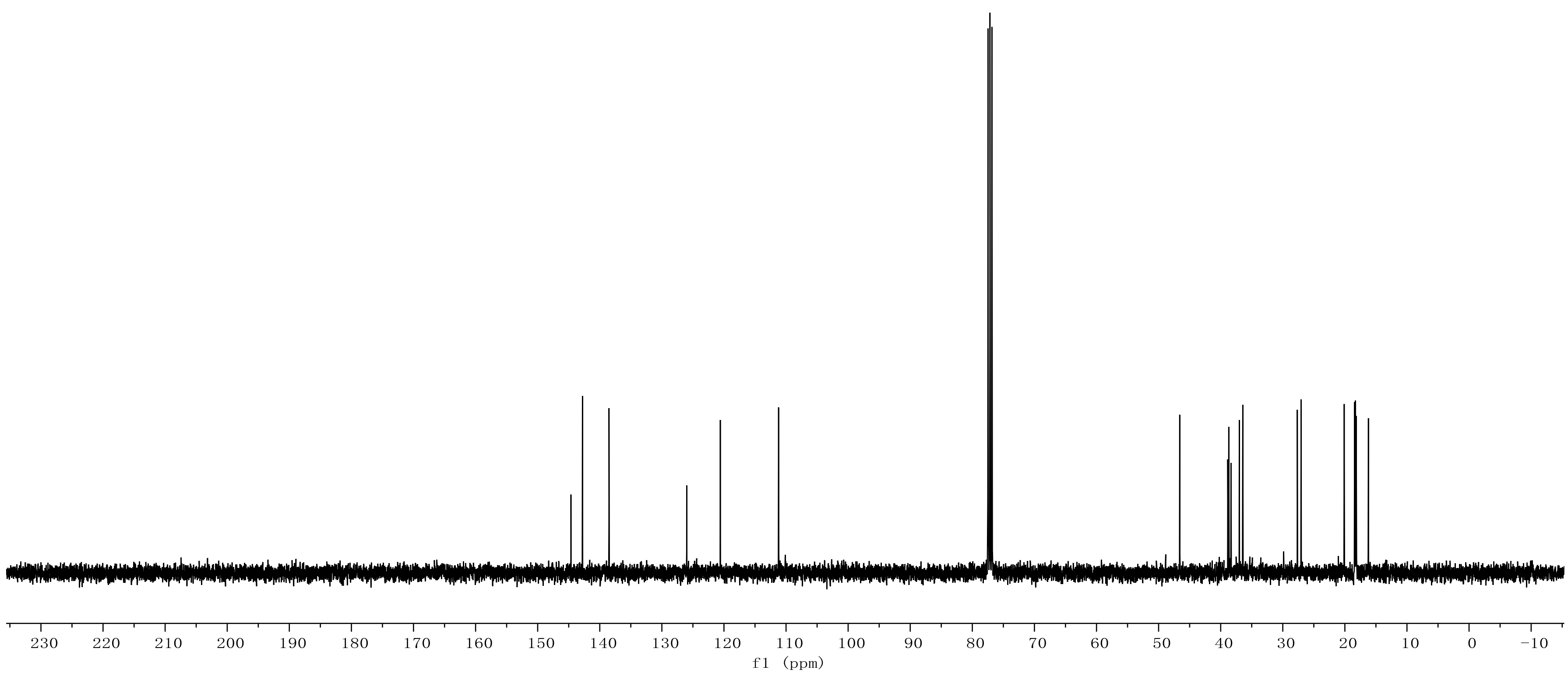

Supplementary Figure 21. ^{1}H NMR Spectrum of PL3 (4) (400 MHz, $CDCl_3$)

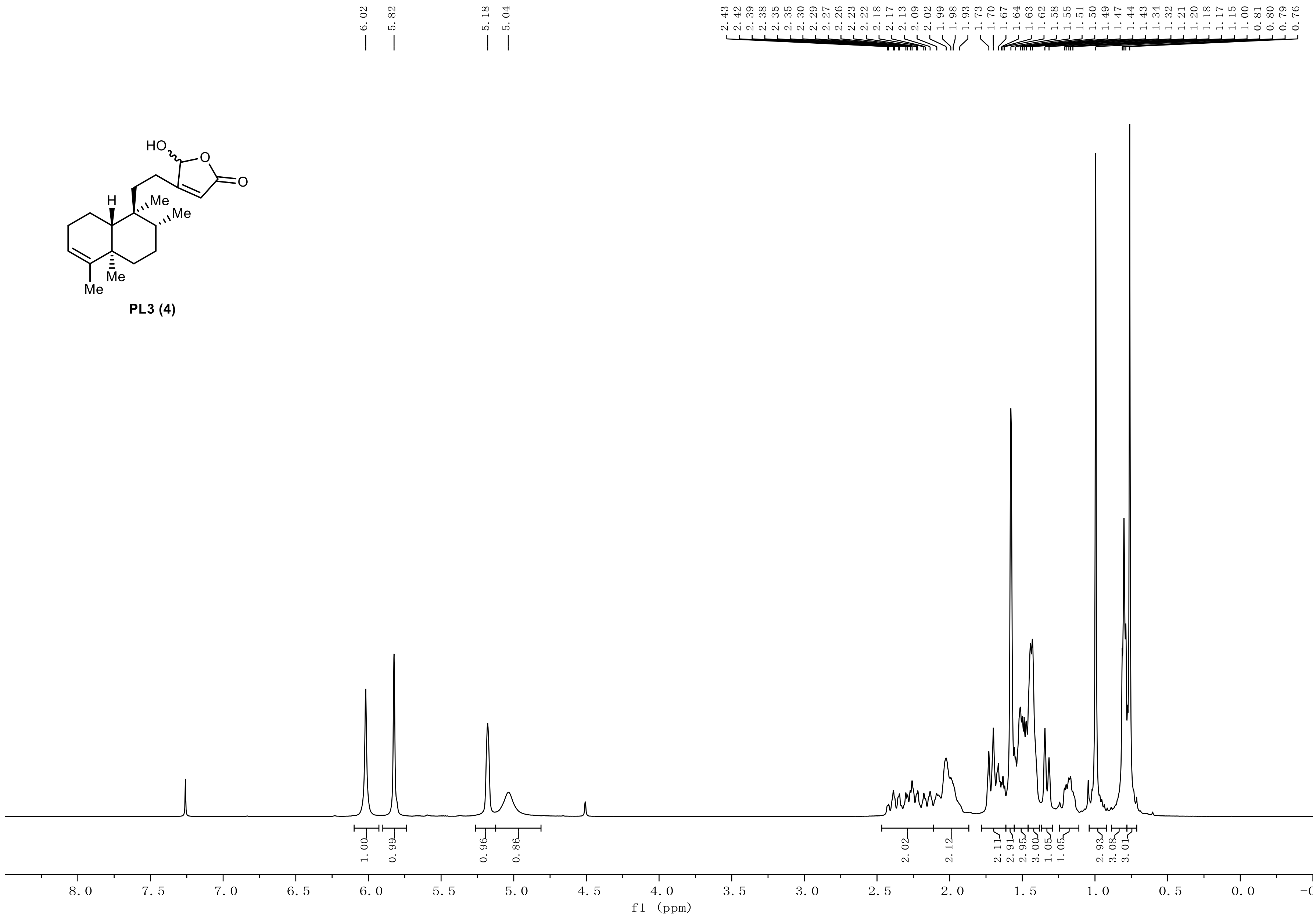

Supplementary Figure 22. ^{13}C NMR Spectrum of PL3 (4) (101 MHz, $CDCl_3$)

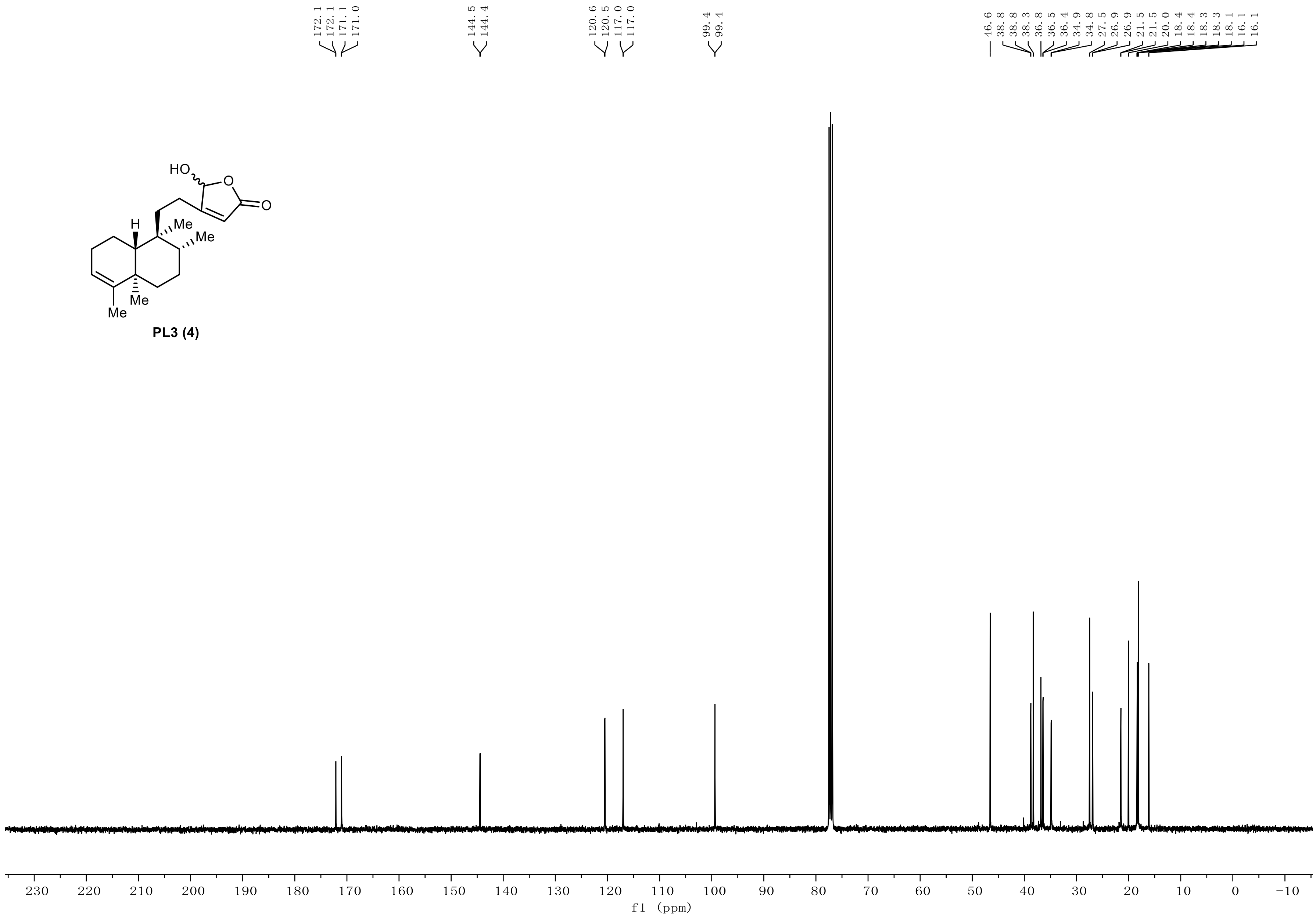

Supplementary Figure 23. ^{1}H NMR Spectrum of 26 (600 MHz, $CDCl_3$)

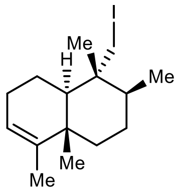

26

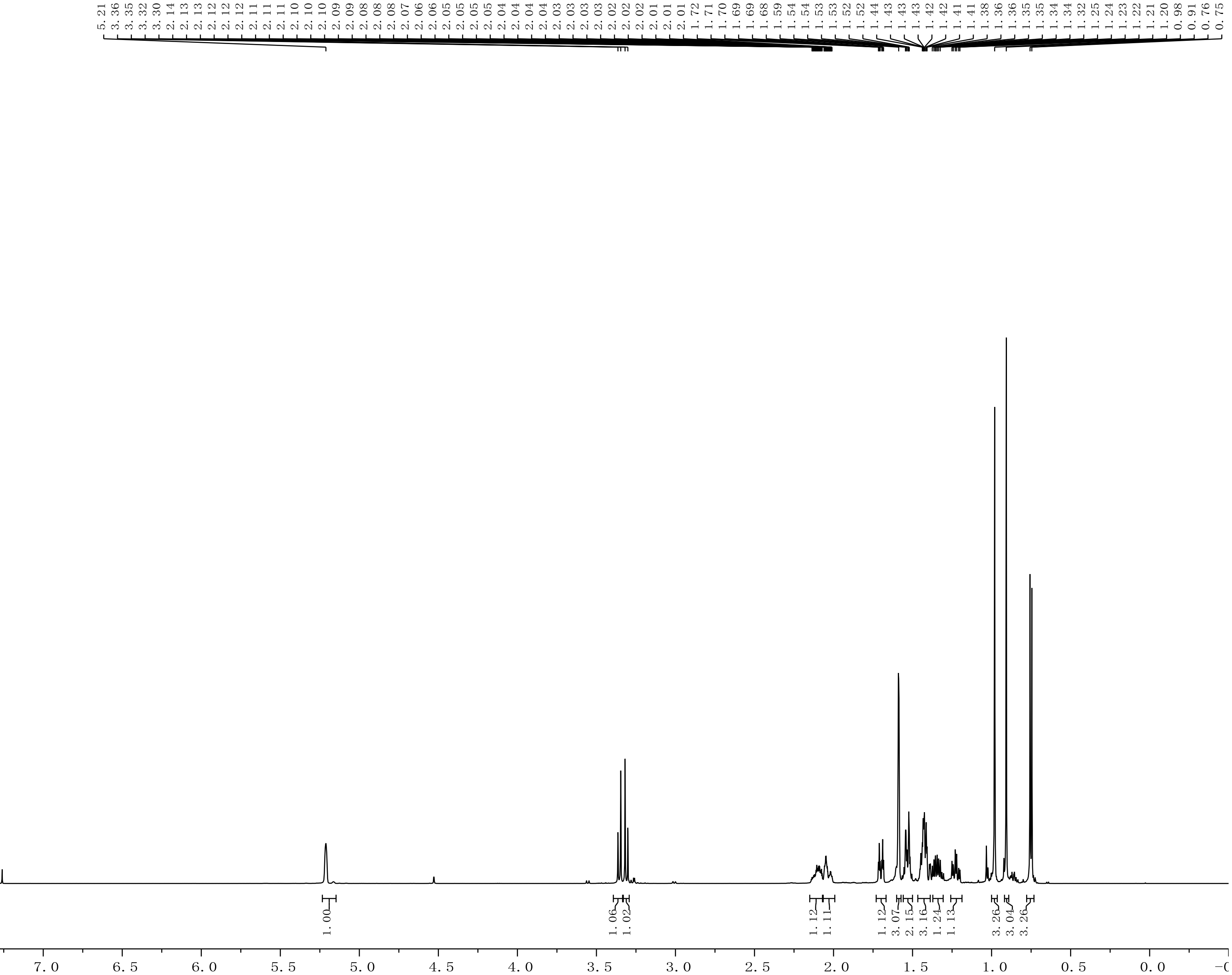

Supplementary Figure 24. ^{13}C NMR Spectrum of 26 (151 MHz, $CDCl_3$)

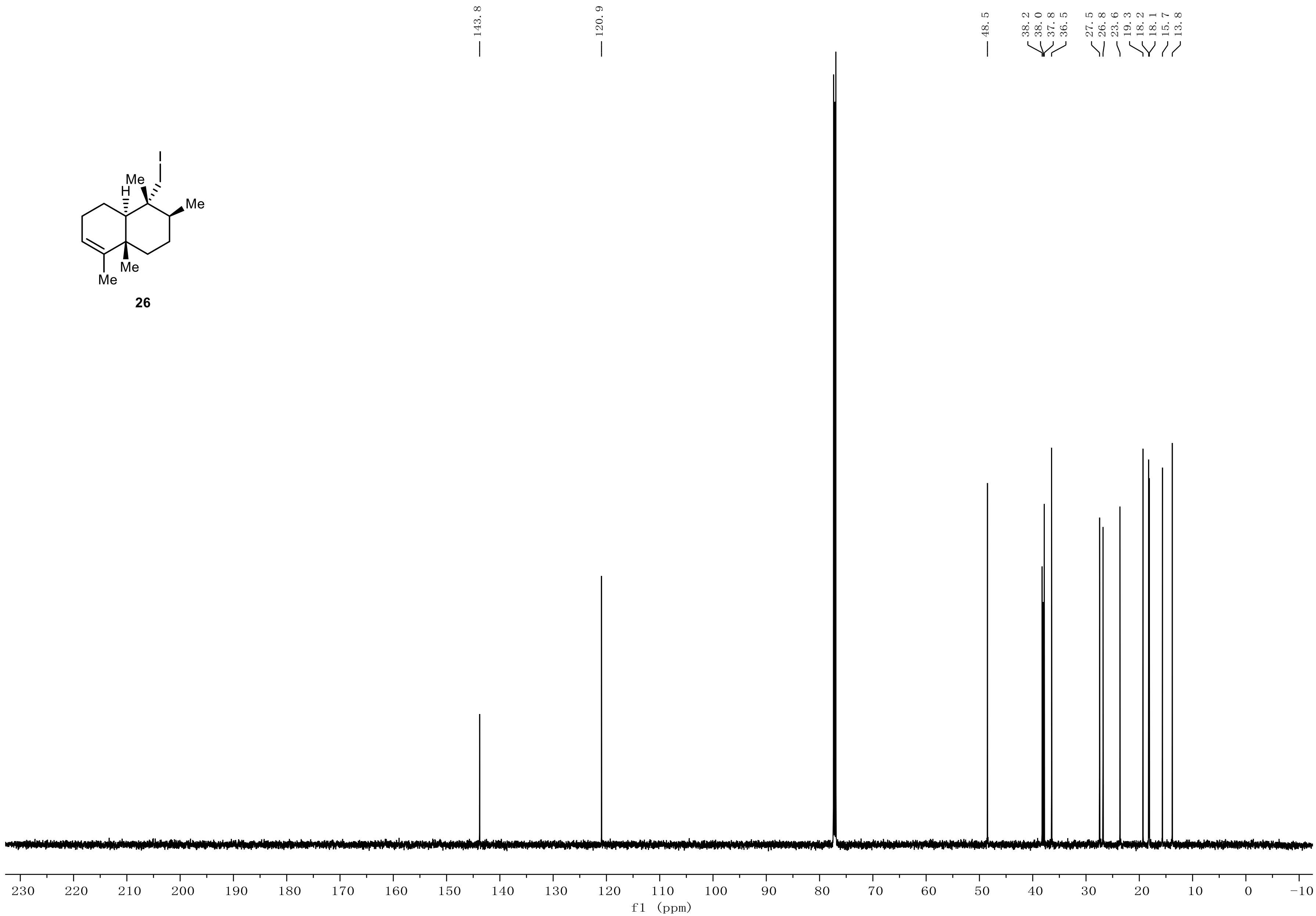

Supplementary Figure 25. ^{1}H NMR Spectrum of 29 (400 MHz, $CDCl_3$)

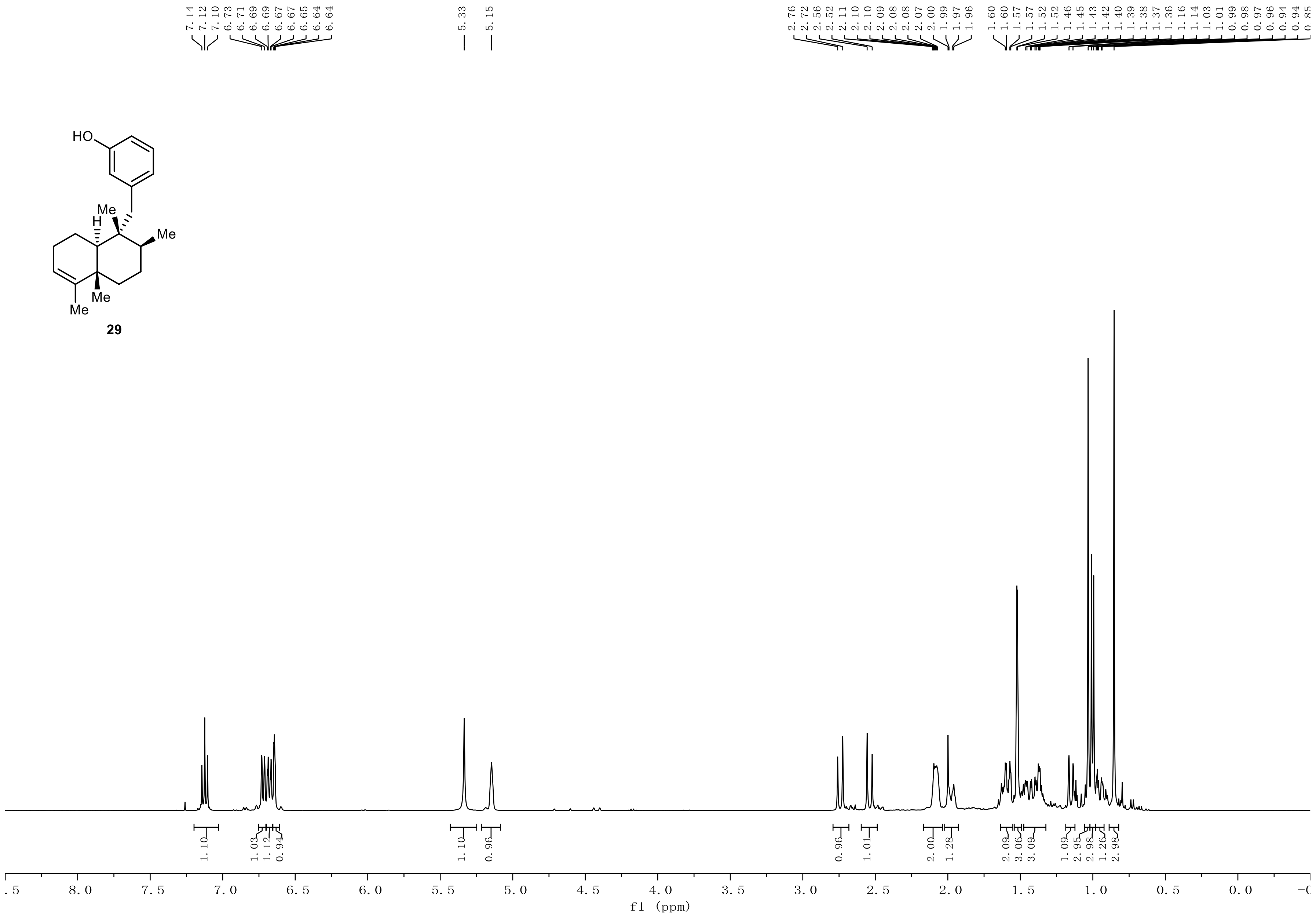

Supplementary Figure 26. ^{13}C NMR Spectrum of 29 (101 MHz, $CDCl_3$)

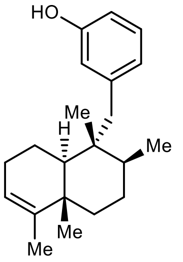

29

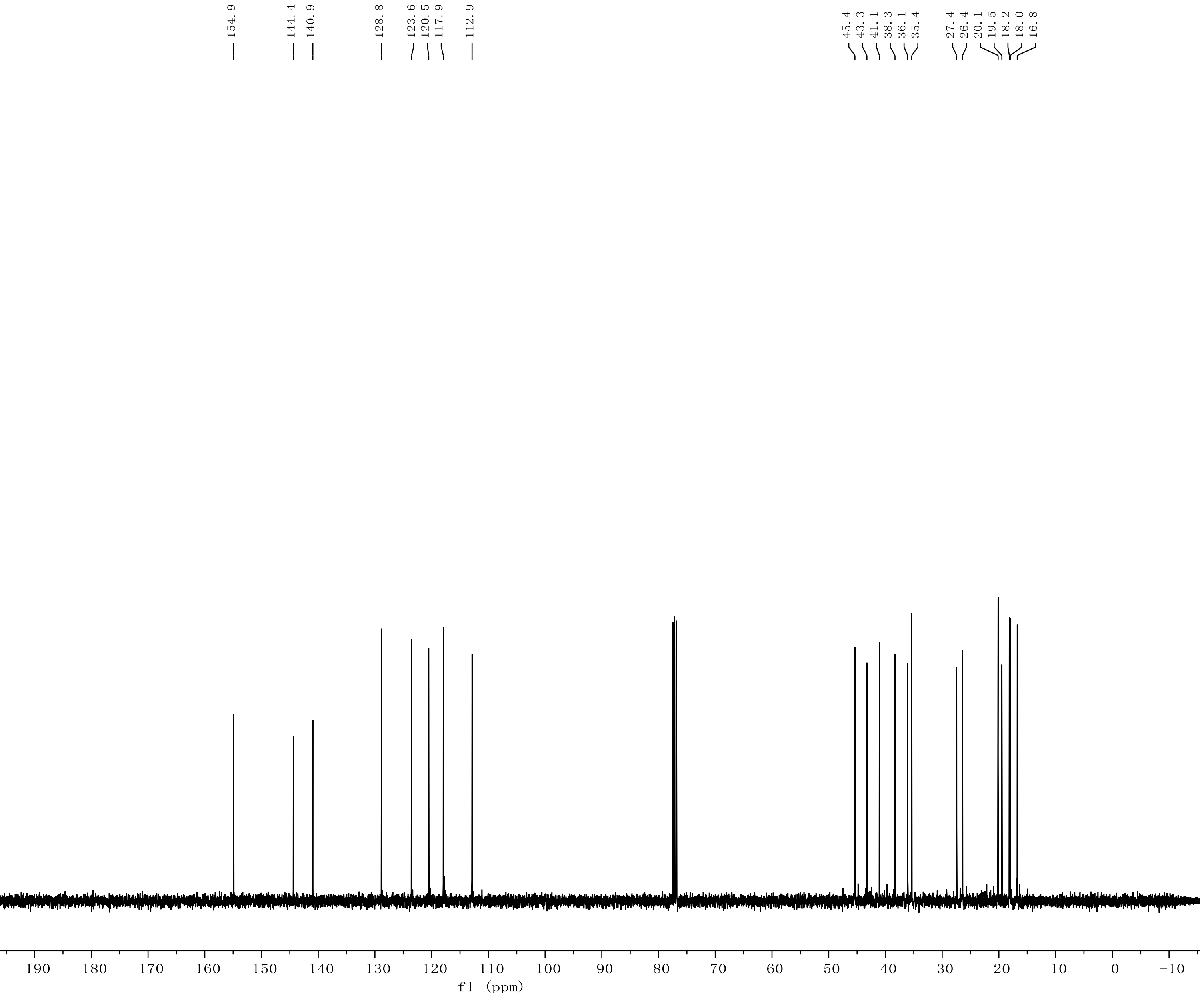

Supplementary Figure 27. ^{1}H NMR Spectrum of Avarone (6) (400 MHz, $CDCl_3$)

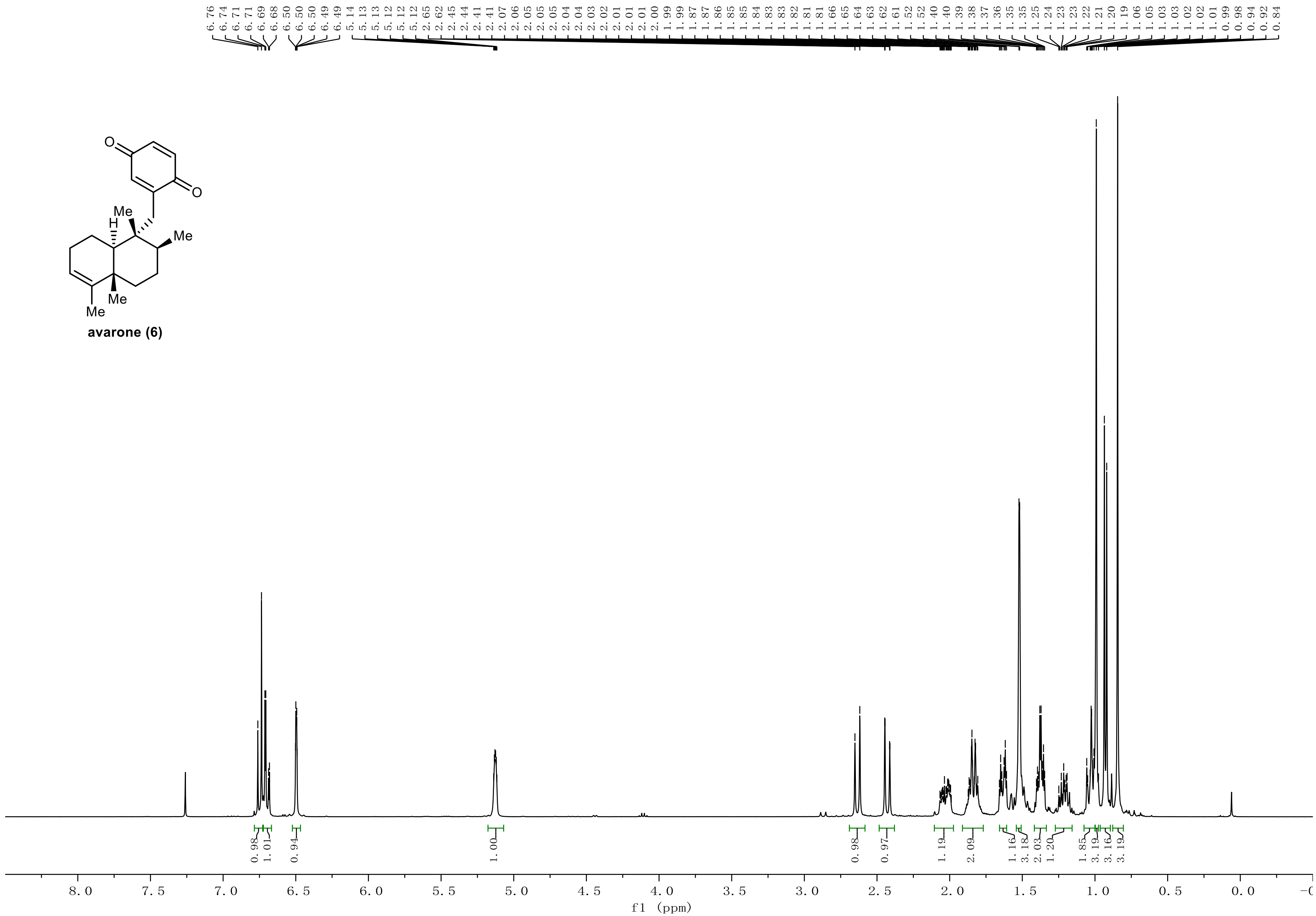

Supplementary Figure 28. ^{13}C NMR Spectrum of Avarone (6) (101 MHz, $CDCl_3$)

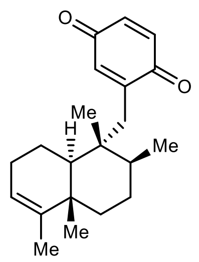

avarone (6)

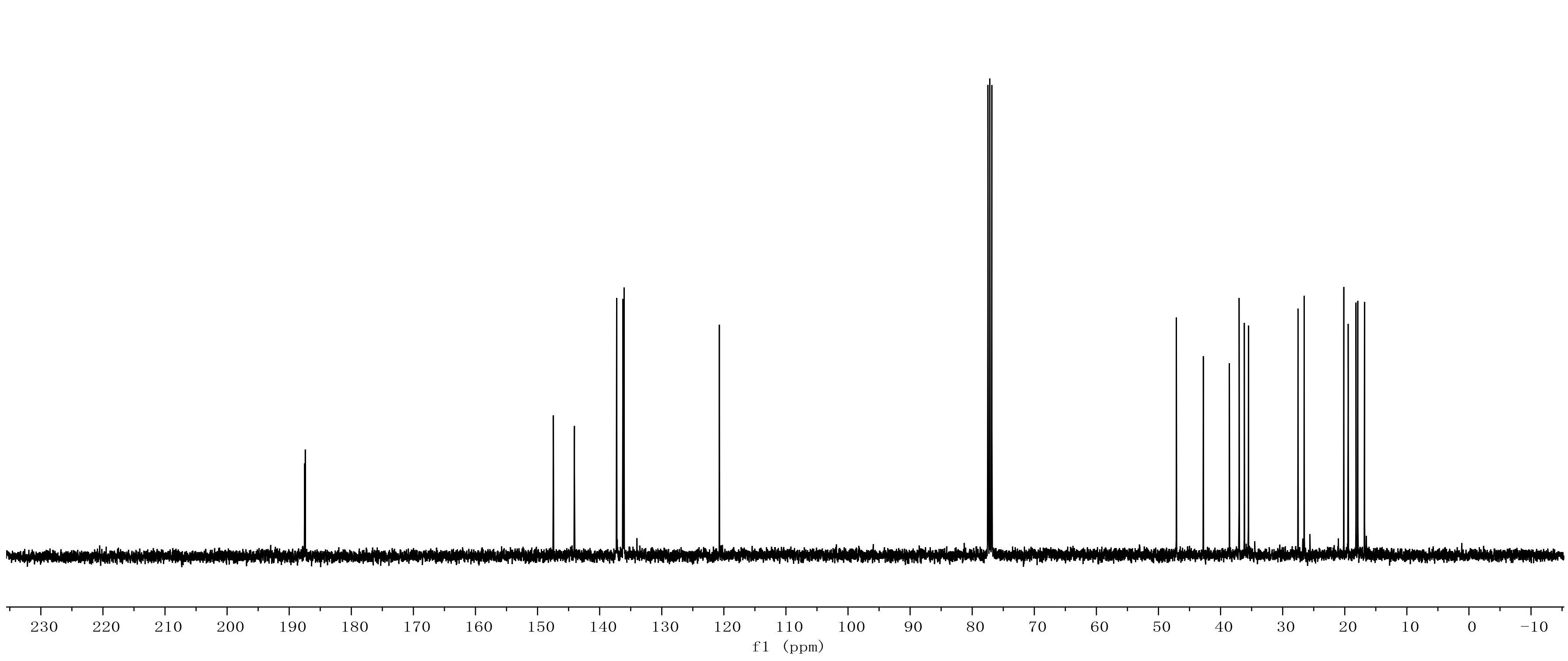

Supplementary Figure 29. ^{1}H NMR Spectrum of Avarol (5) (600 MHz, $CDCl_3$)

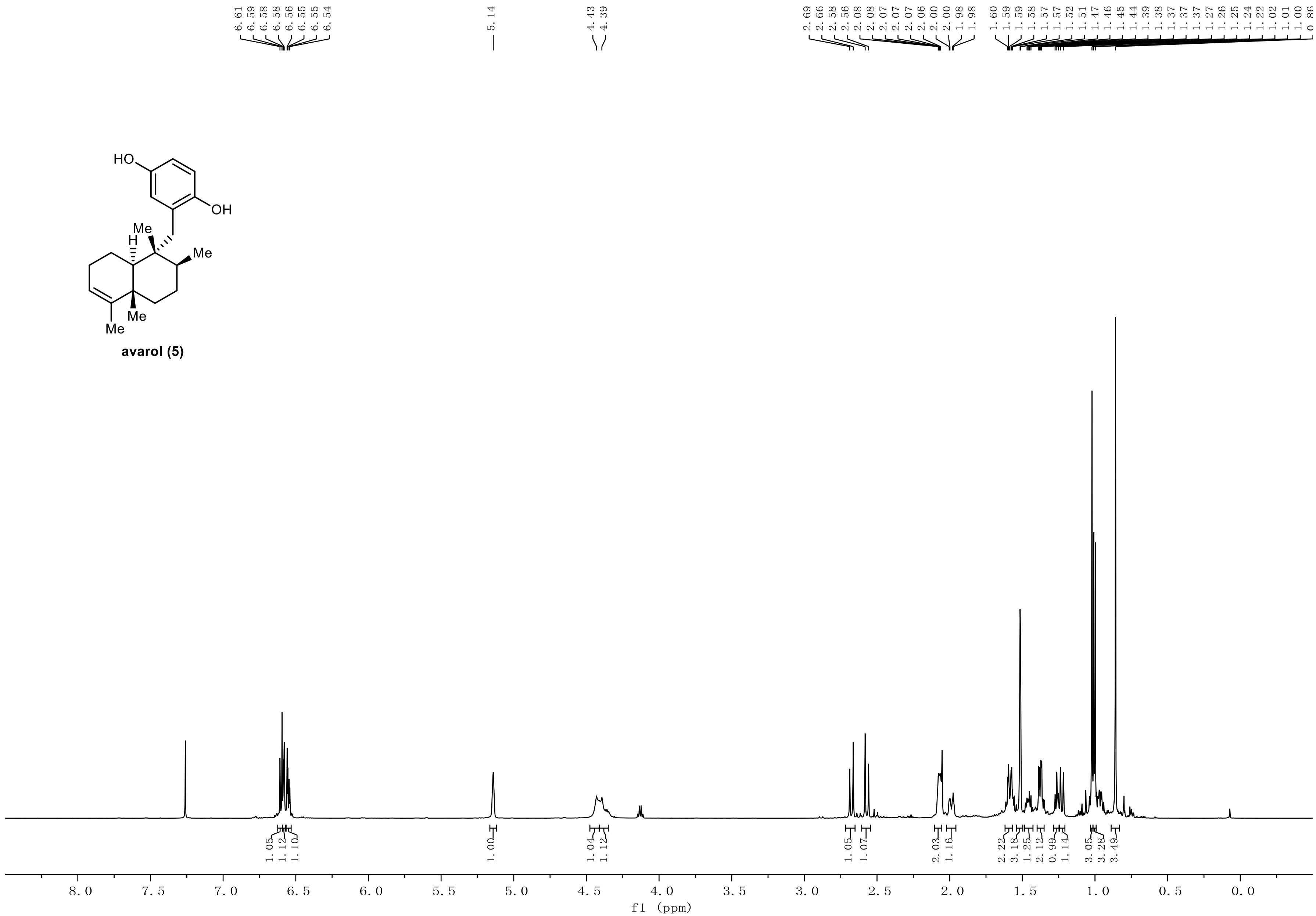

Supplementary Figure 30. ^{13}C NMR Spectrum of Avarol (5) (101 MHz, $CDCl_3$)

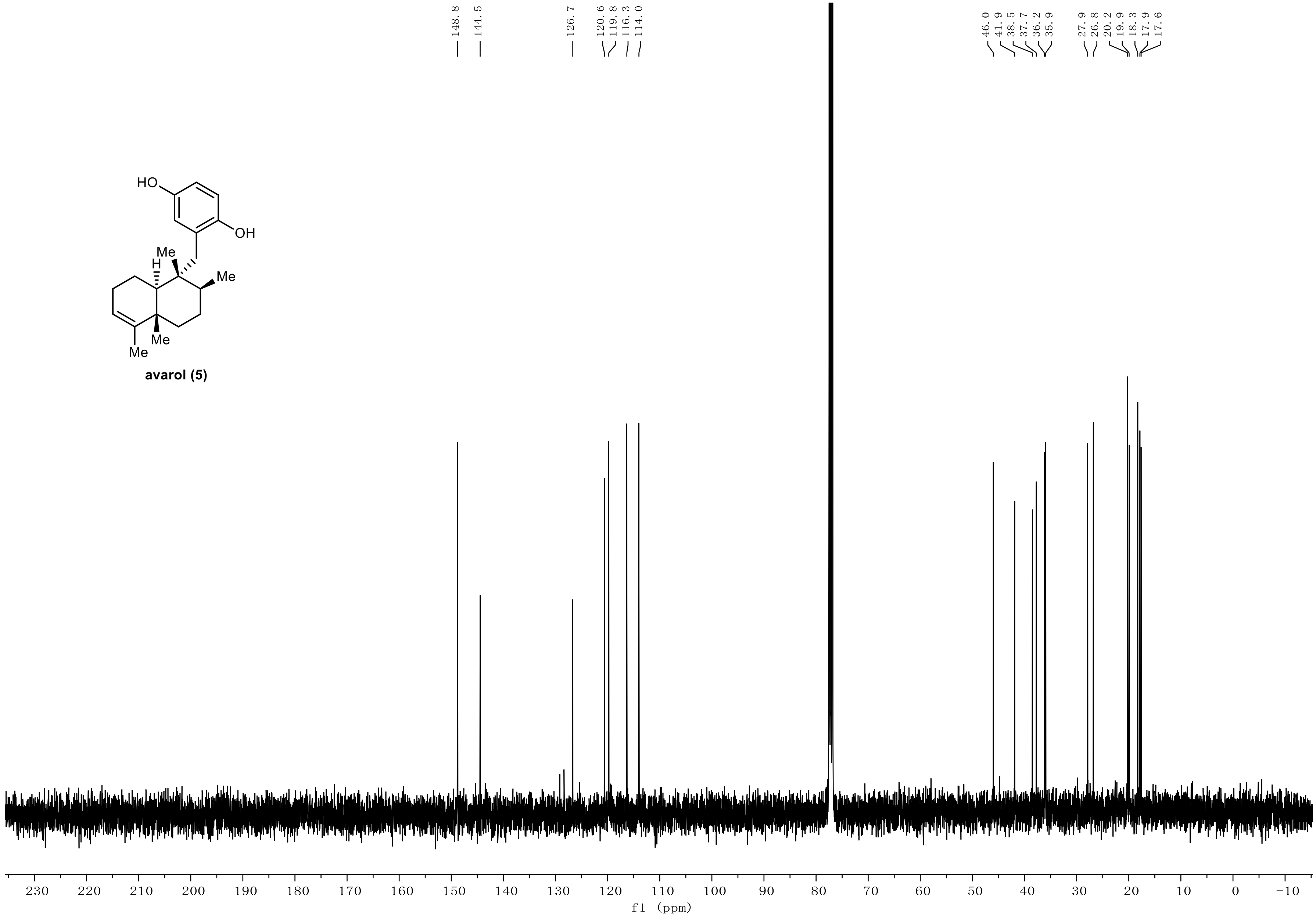

Supplementary Figure 31. ^{1}H NMR Spectrum of SI-7 (400 MHz, $CDCl_3$)

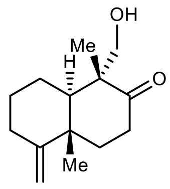

SI-7

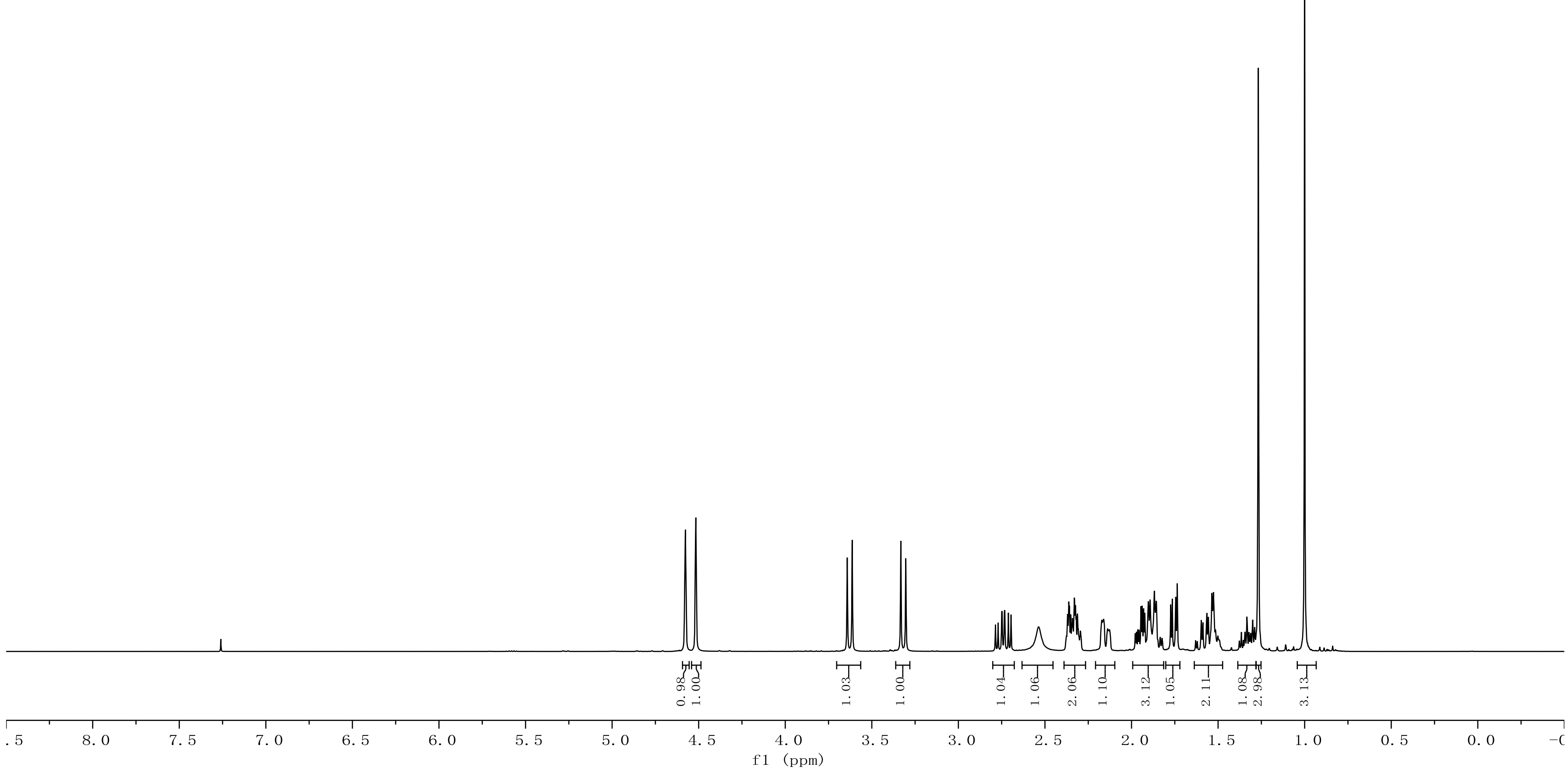

Supplementary Figure 32. ^{13}C NMR Spectrum of SI-7 (101 MHz, $CDCl_3$)

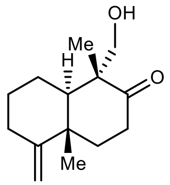

SI-7

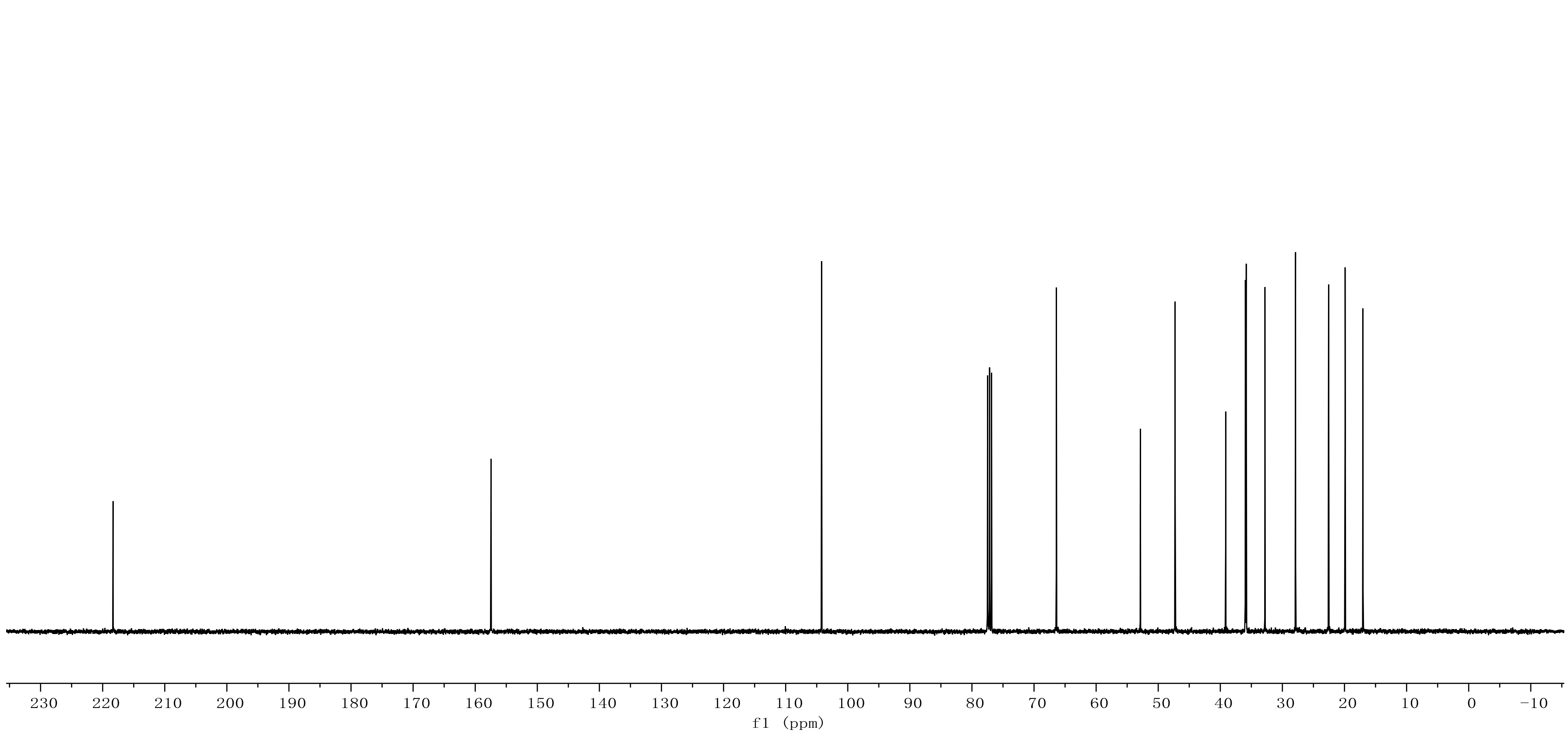

Supplementary Figure 33. ^{1}H NMR Spectrum of 31 (400 MHz, $CDCl_3$)

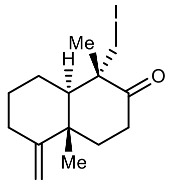

31

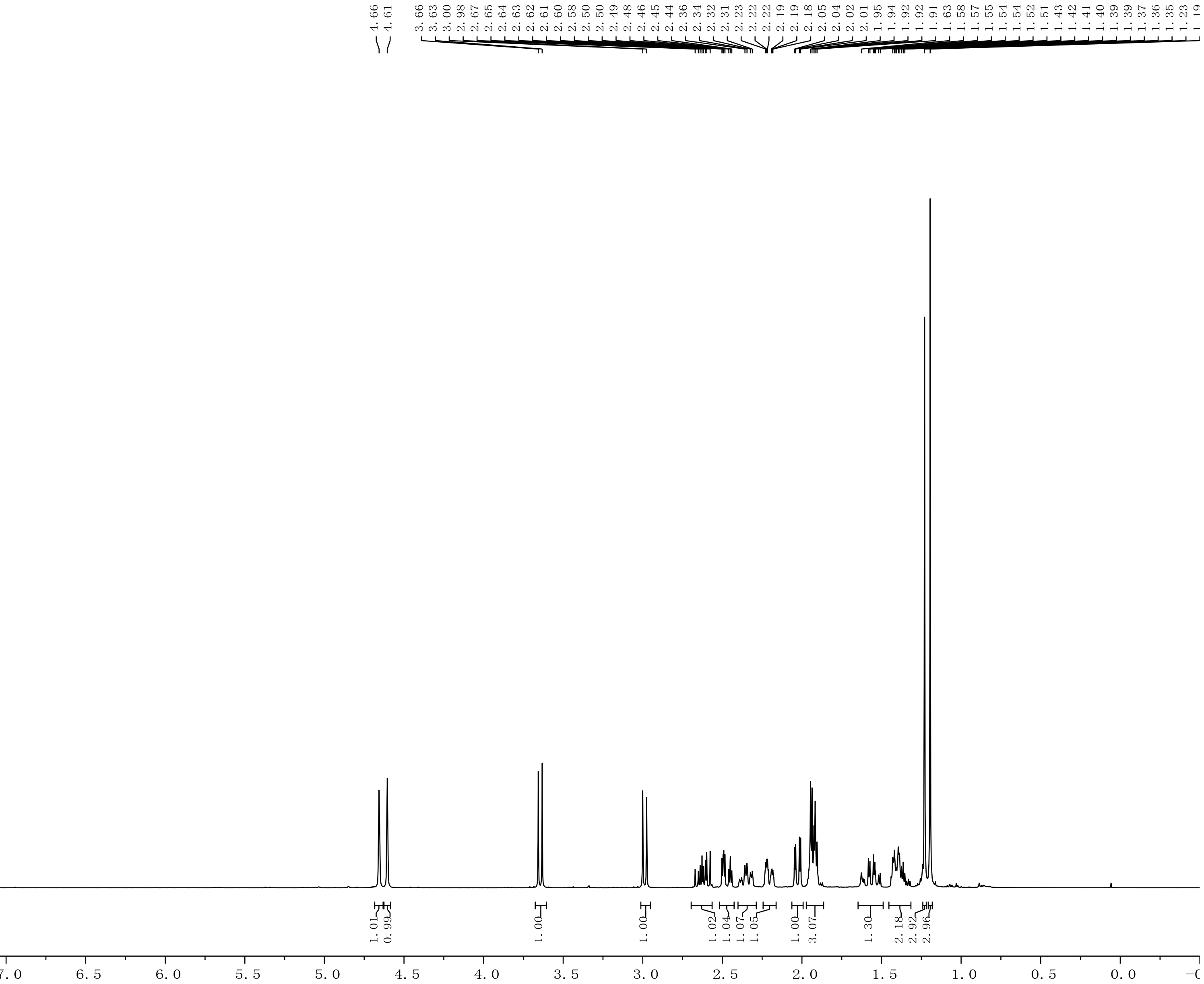

Supplementary Figure 34. ^{13}C NMR Spectrum of 31 (101 MHz, $CDCl_3$)

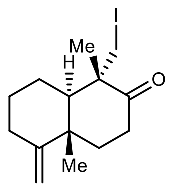

31

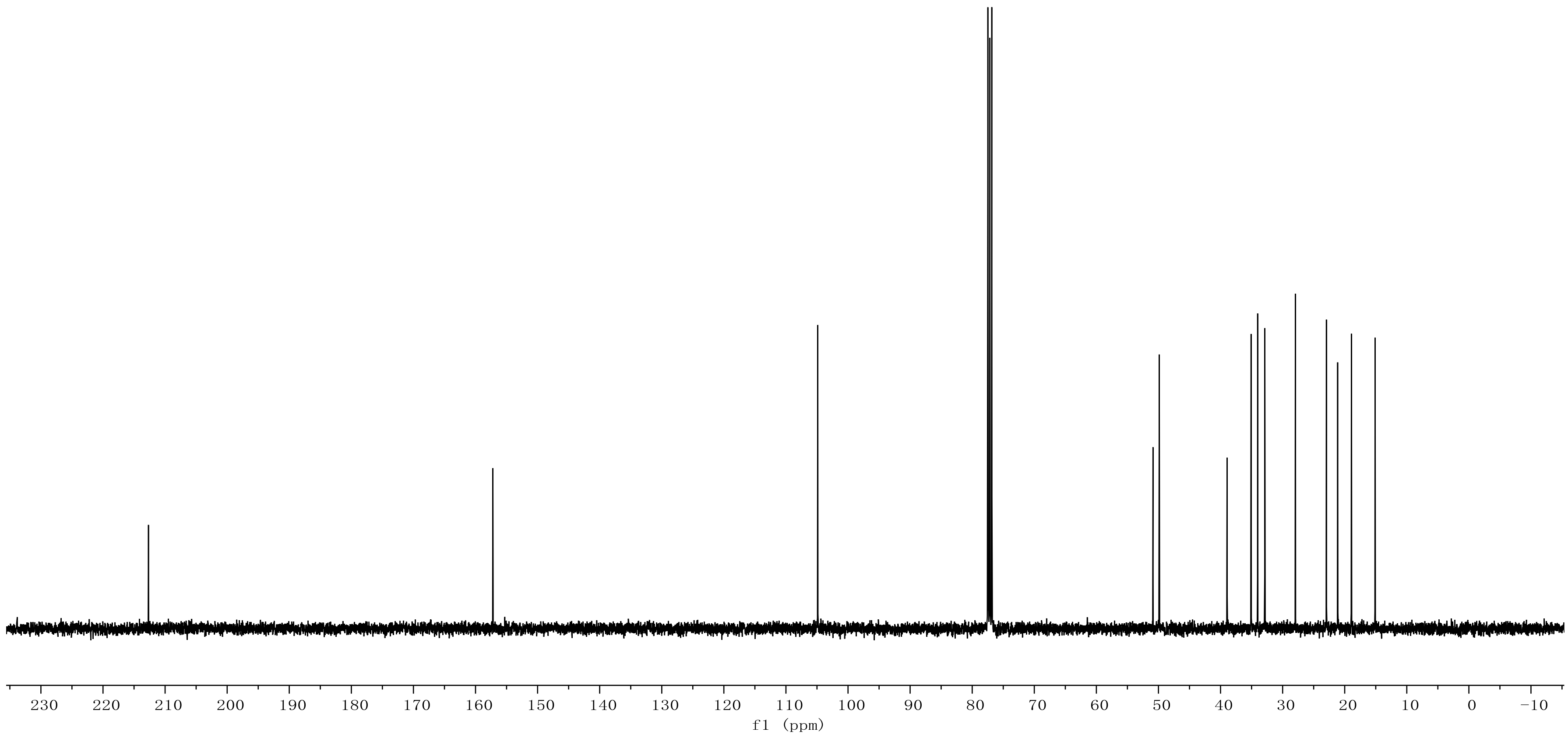

Supplementary Figure 35. ^{1}H NMR Spectrum of 33 (600 MHz, $CDCl_3$)

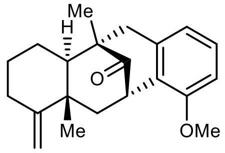

33

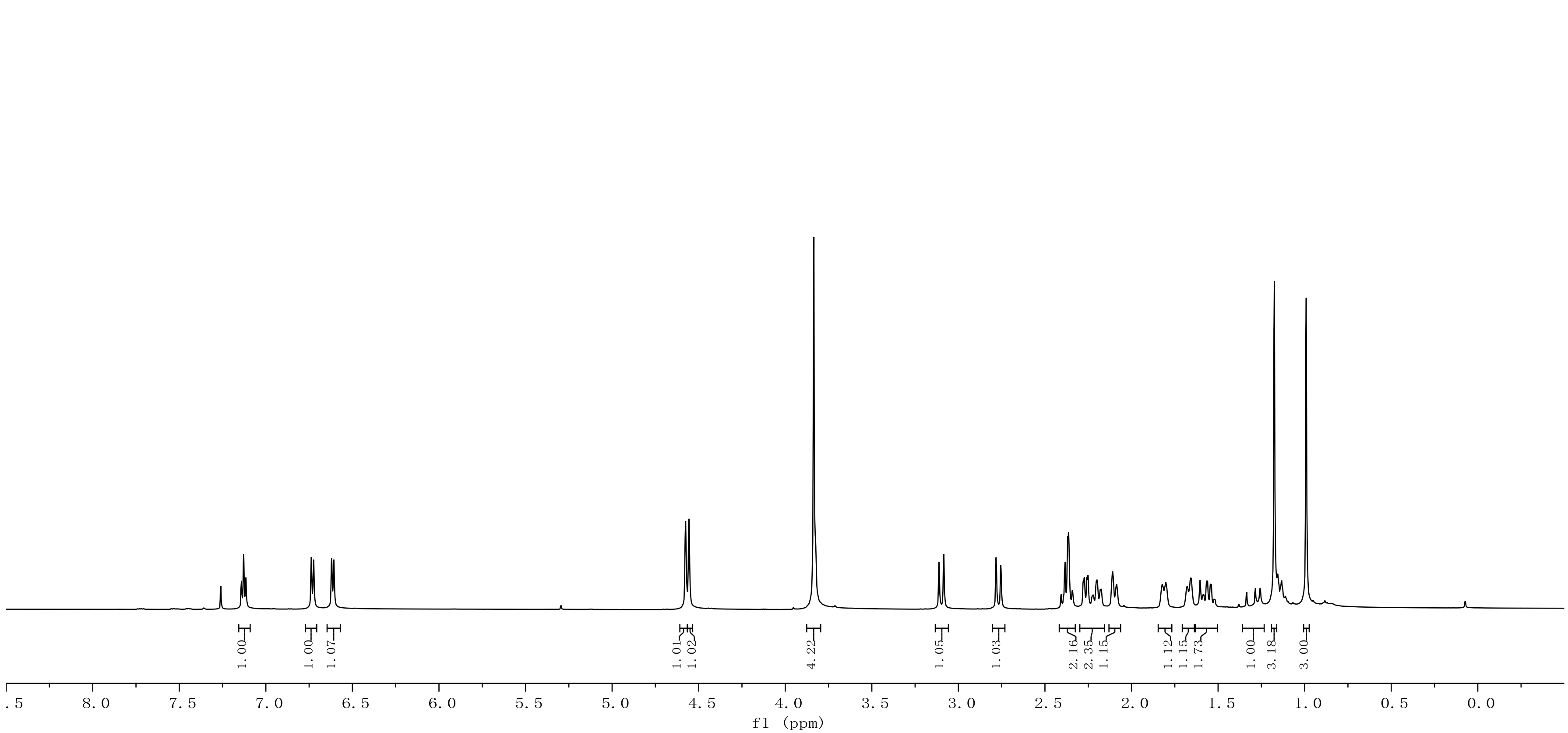

Supplementary Figure 36. ^{13}C NMR Spectrum of 33 (151 MHz, $CDCl_3$)

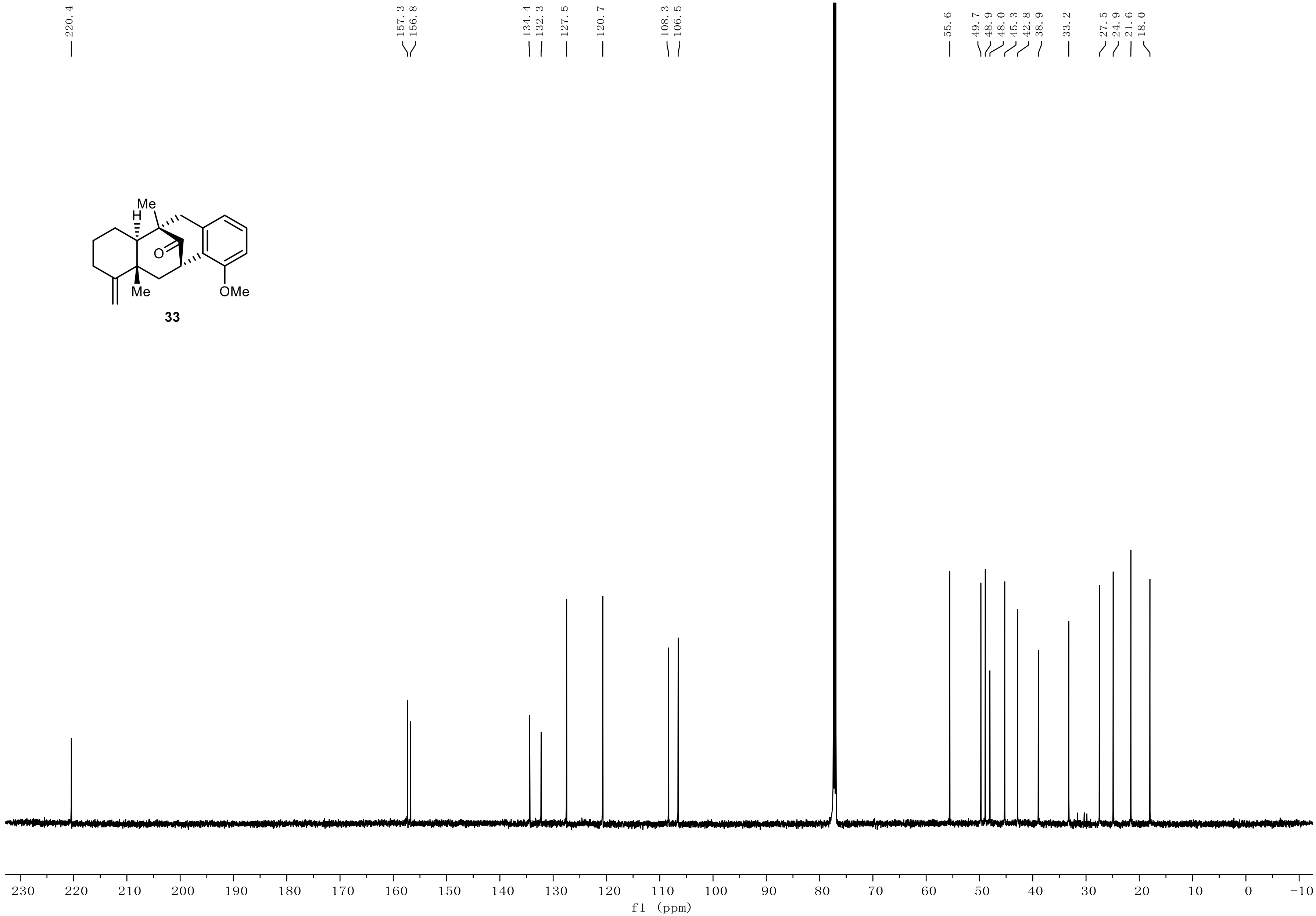

Supplementary Figure 37. ^{1}H NMR Spectrum of SI-8 (400 MHz, $CDCl_3$)

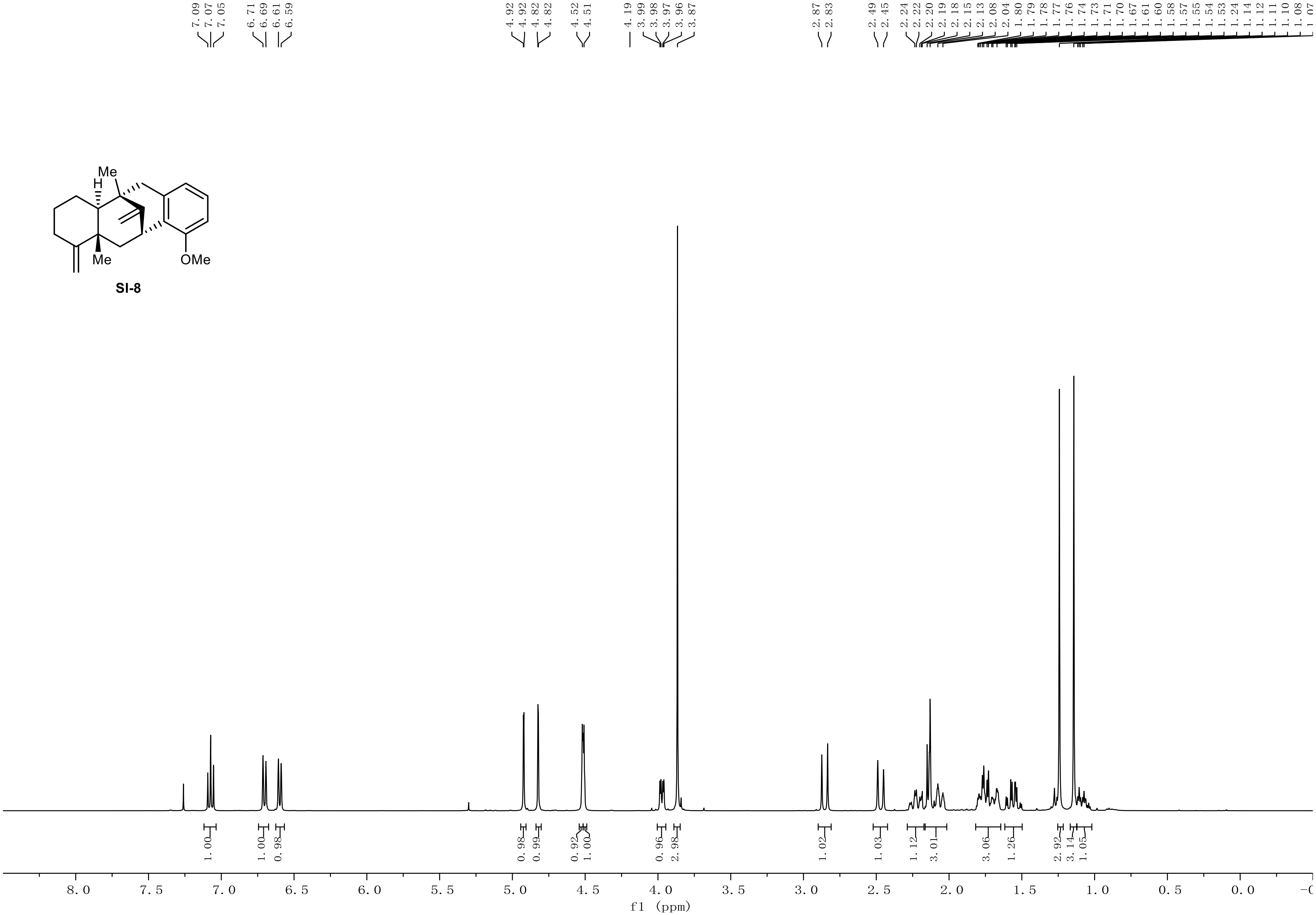

Supplementary Figure 38. ^{13}C NMR Spectrum of SI-8 (101 MHz, $CDCl_3$)

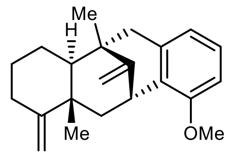

SI-8

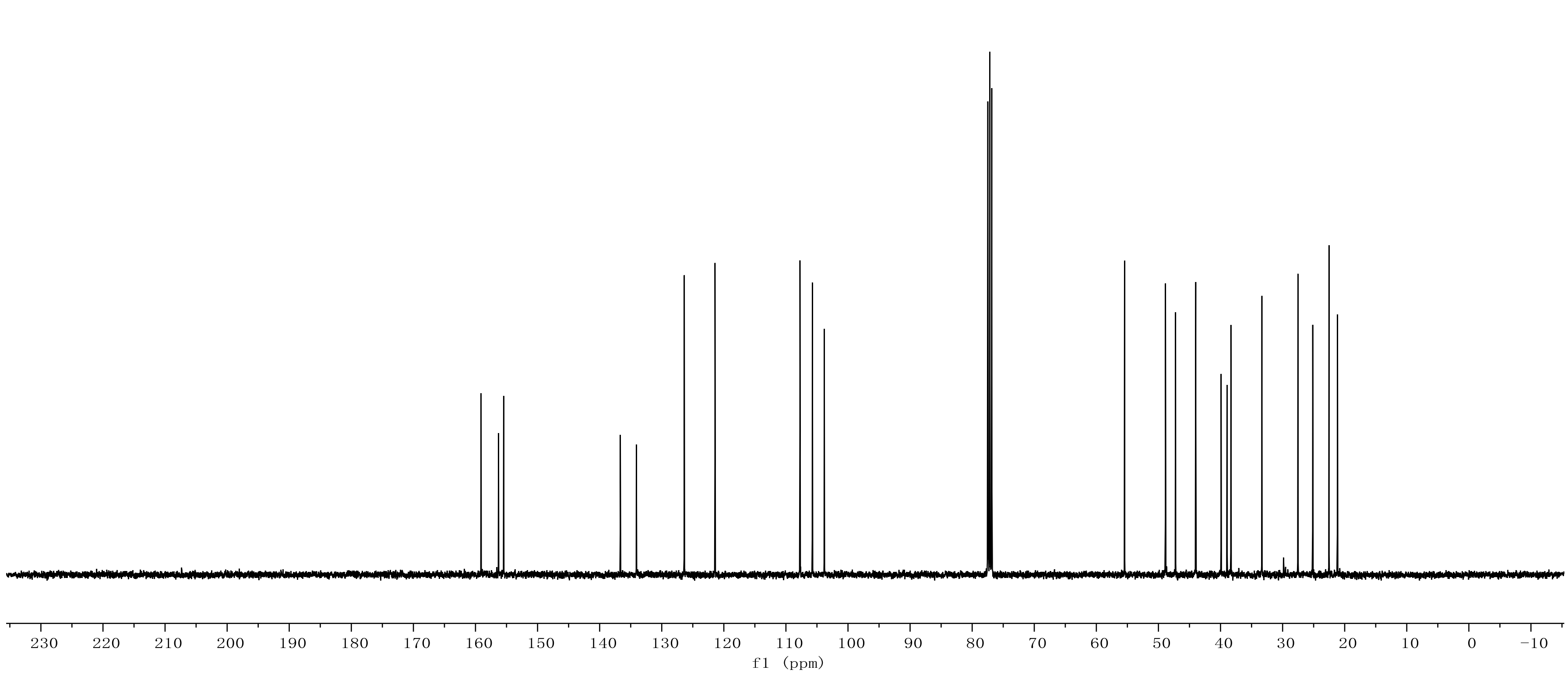

Supplementary Figure 39. ^{1}H NMR Spectrum of SI-9 (400 MHz, $CDCl_3$)

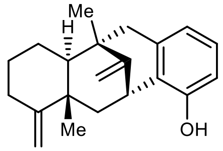

SI-9

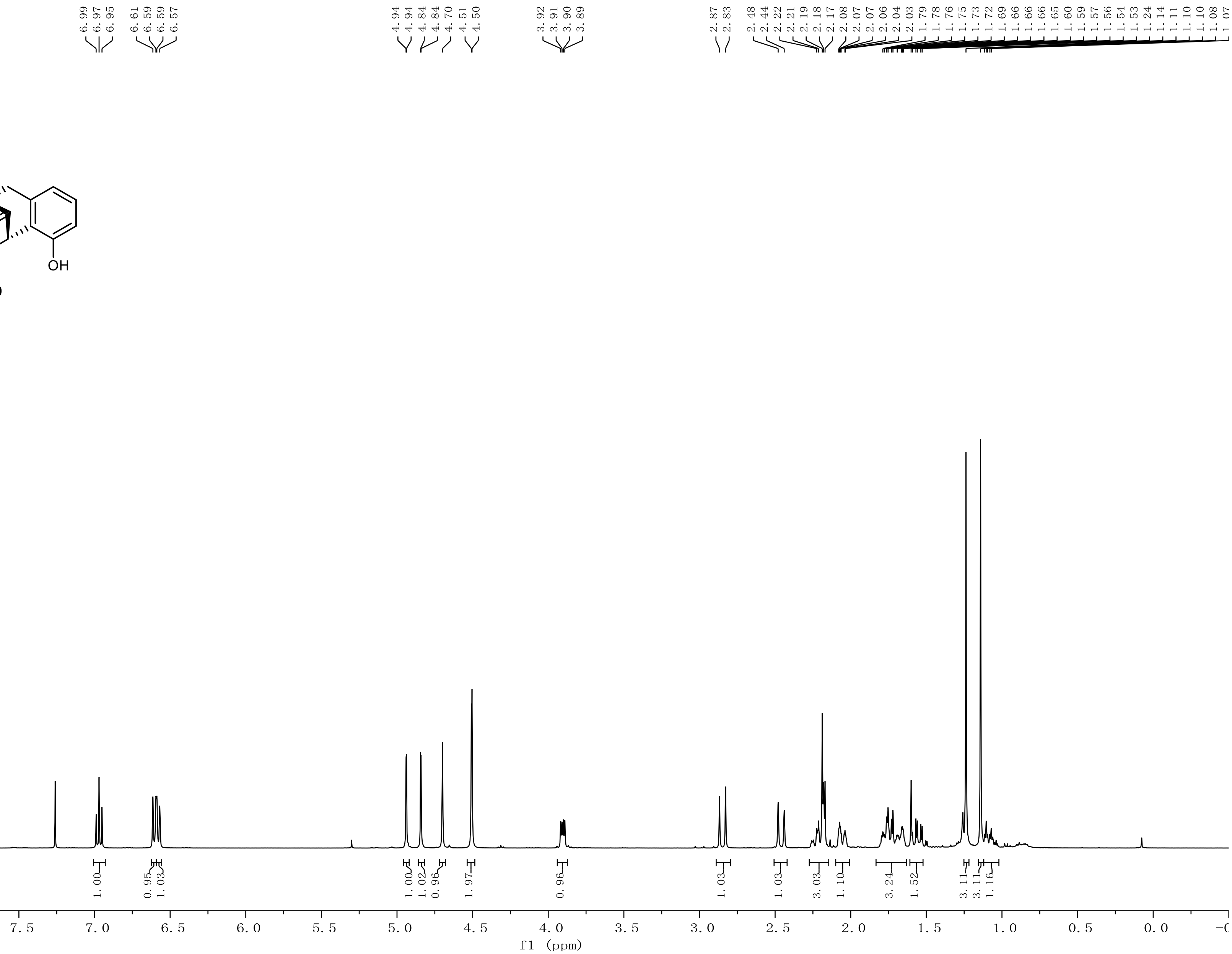

Supplementary Figure 40. ^{13}C NMR Spectrum of SI-9 (101 MHz, $CDCl_3$)

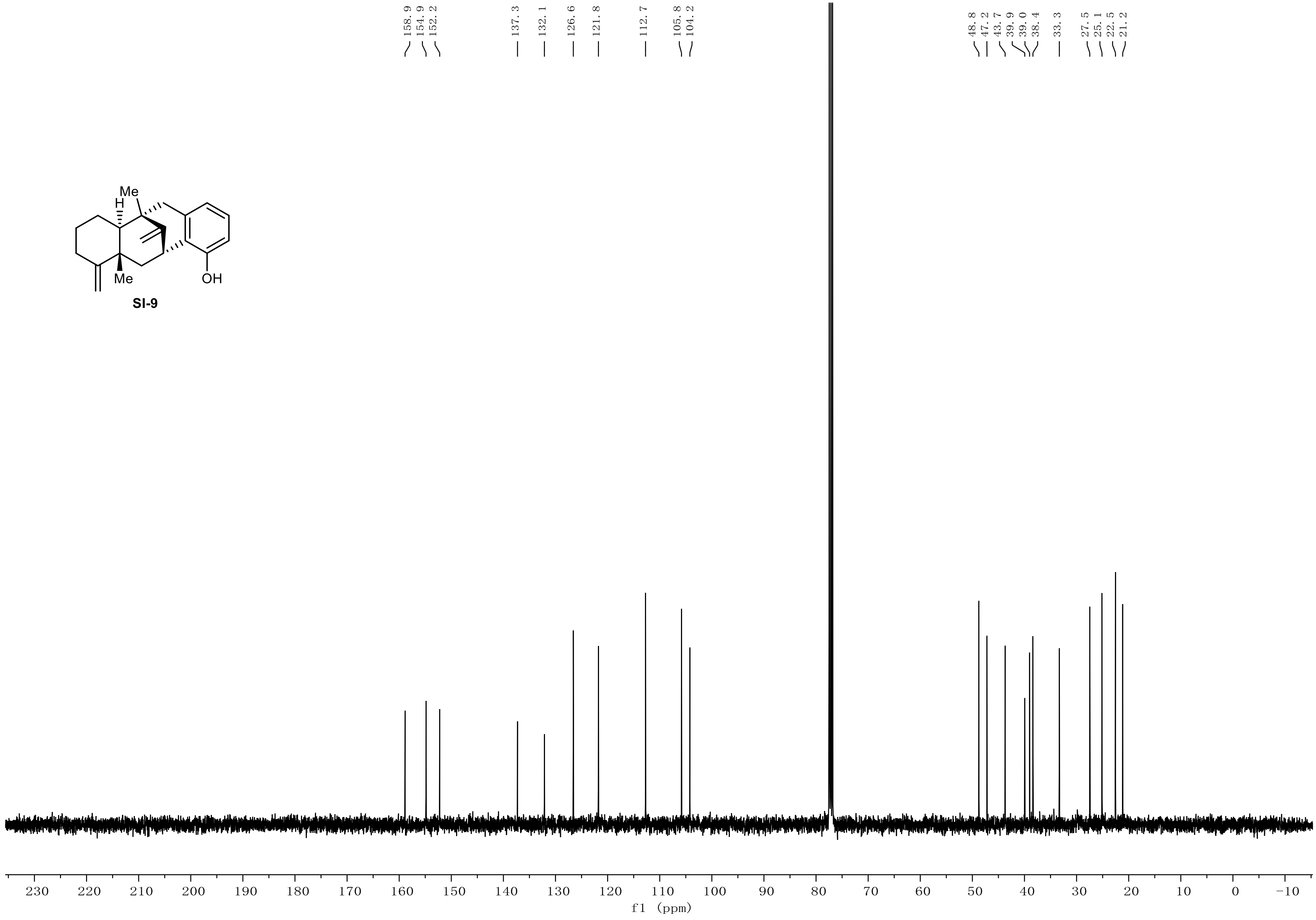

Supplementary Figure 41. ^{1}H NMR Spectrum of 34 (400 MHz, $CDCl_3$)

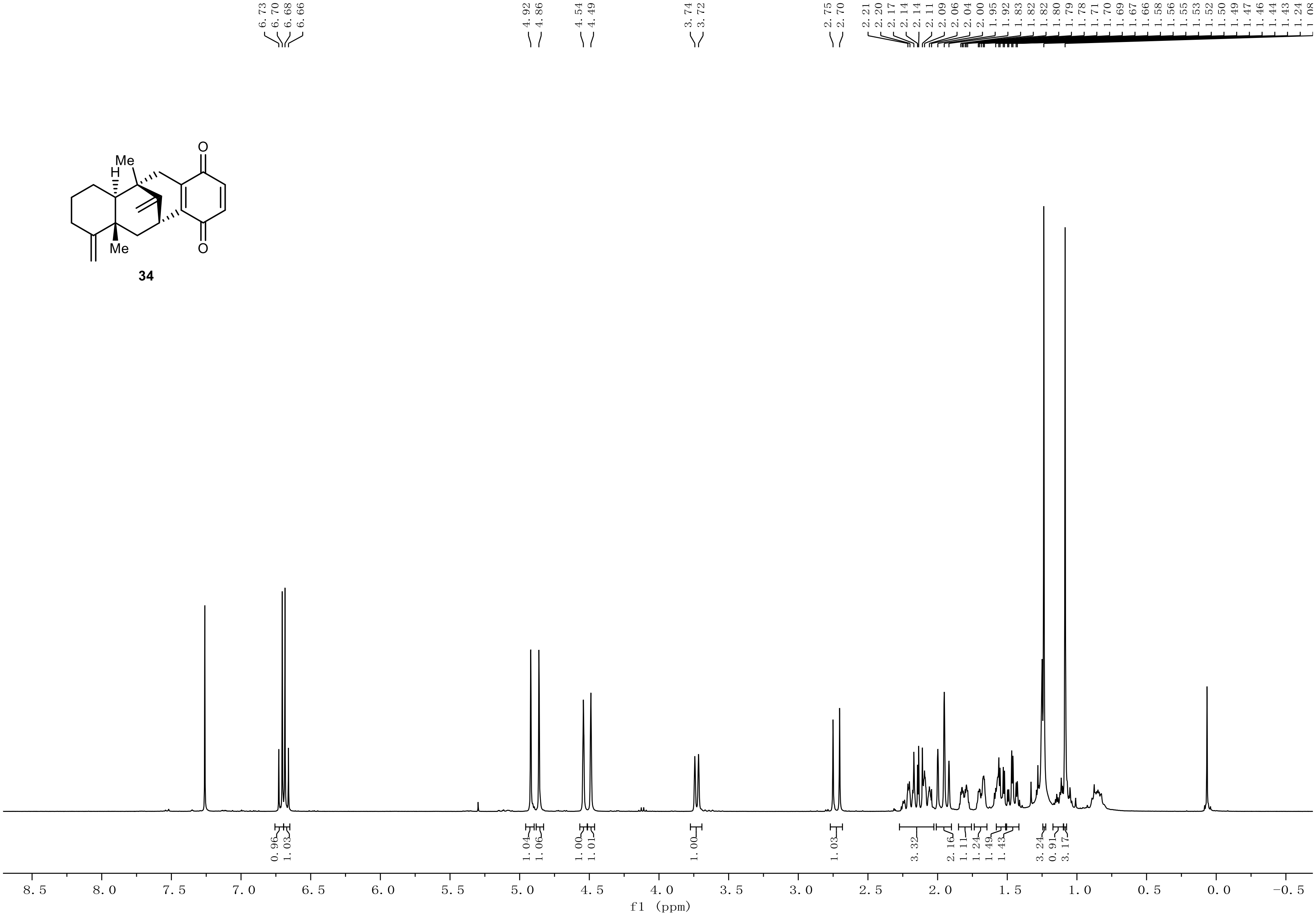

34

Supplementary Figure 42. ^{13}C NMR Spectrum of 34 (101 MHz, $CDCl_3$)

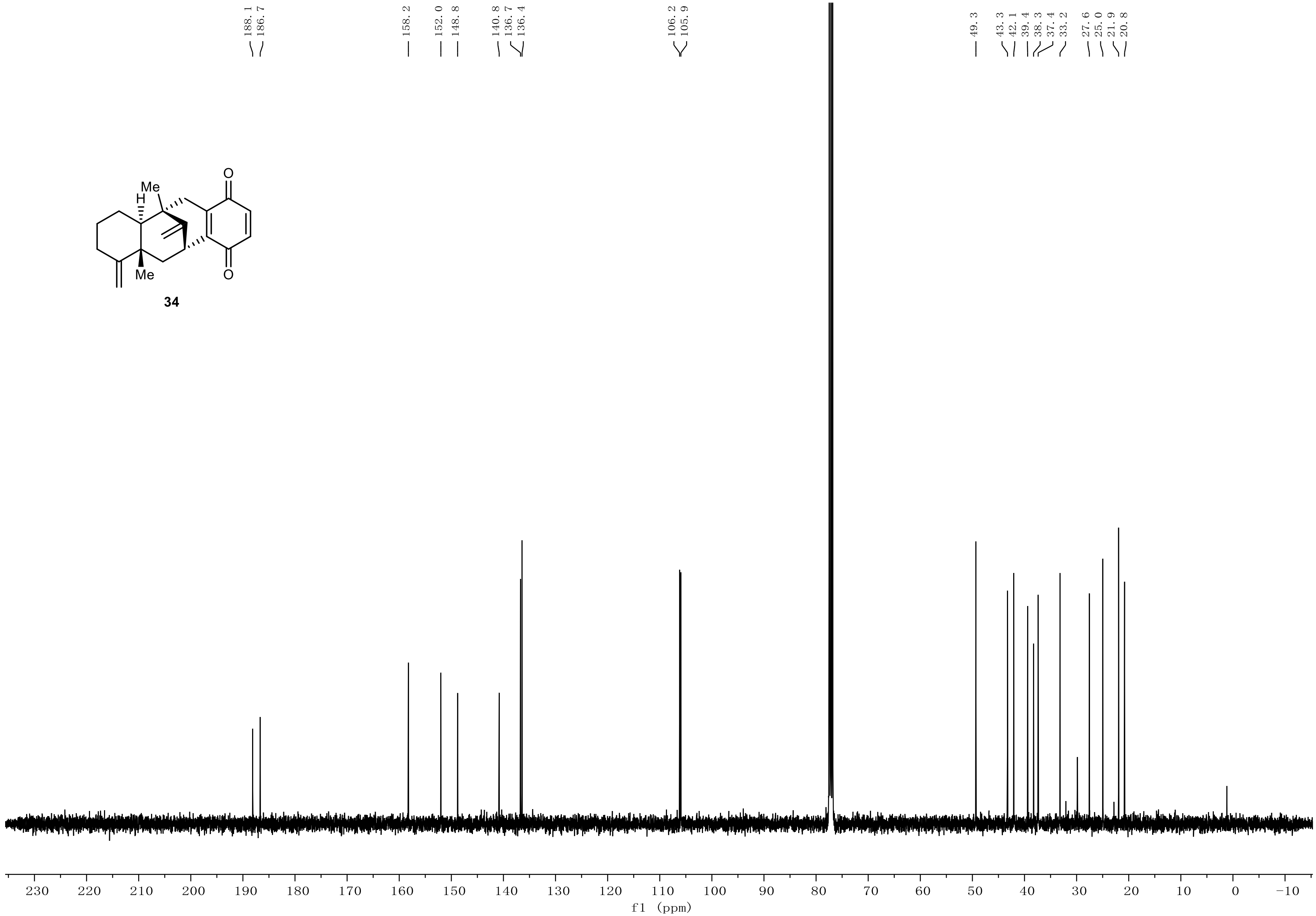

Supplementary Figure 43. ^{1}H NMR Spectrum of 35 (400 MHz, $CDCl_3$)

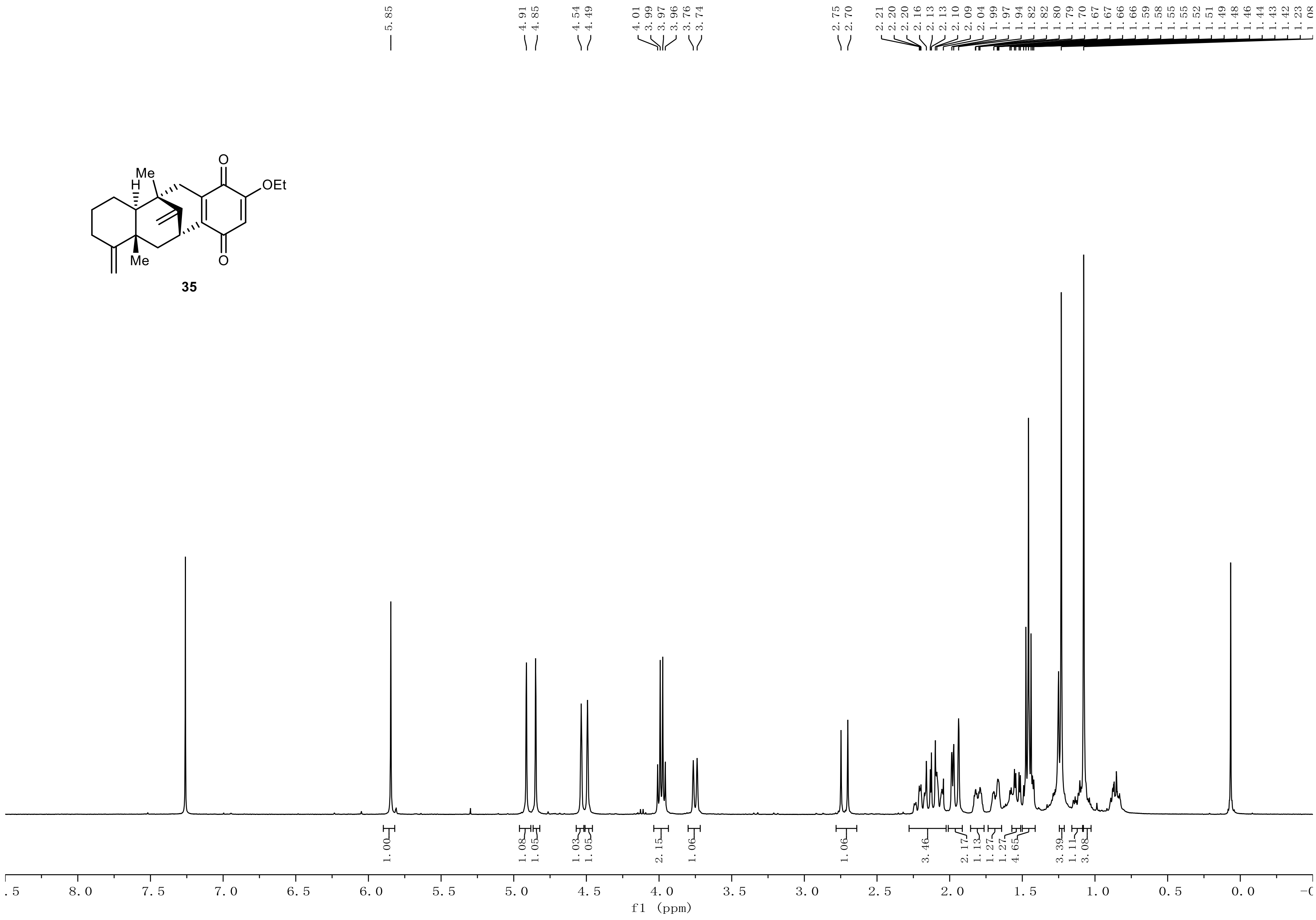

Supplementary Figure 44. ^{13}C NMR Spectrum of 35 (101 MHz, $CDCl_3$)

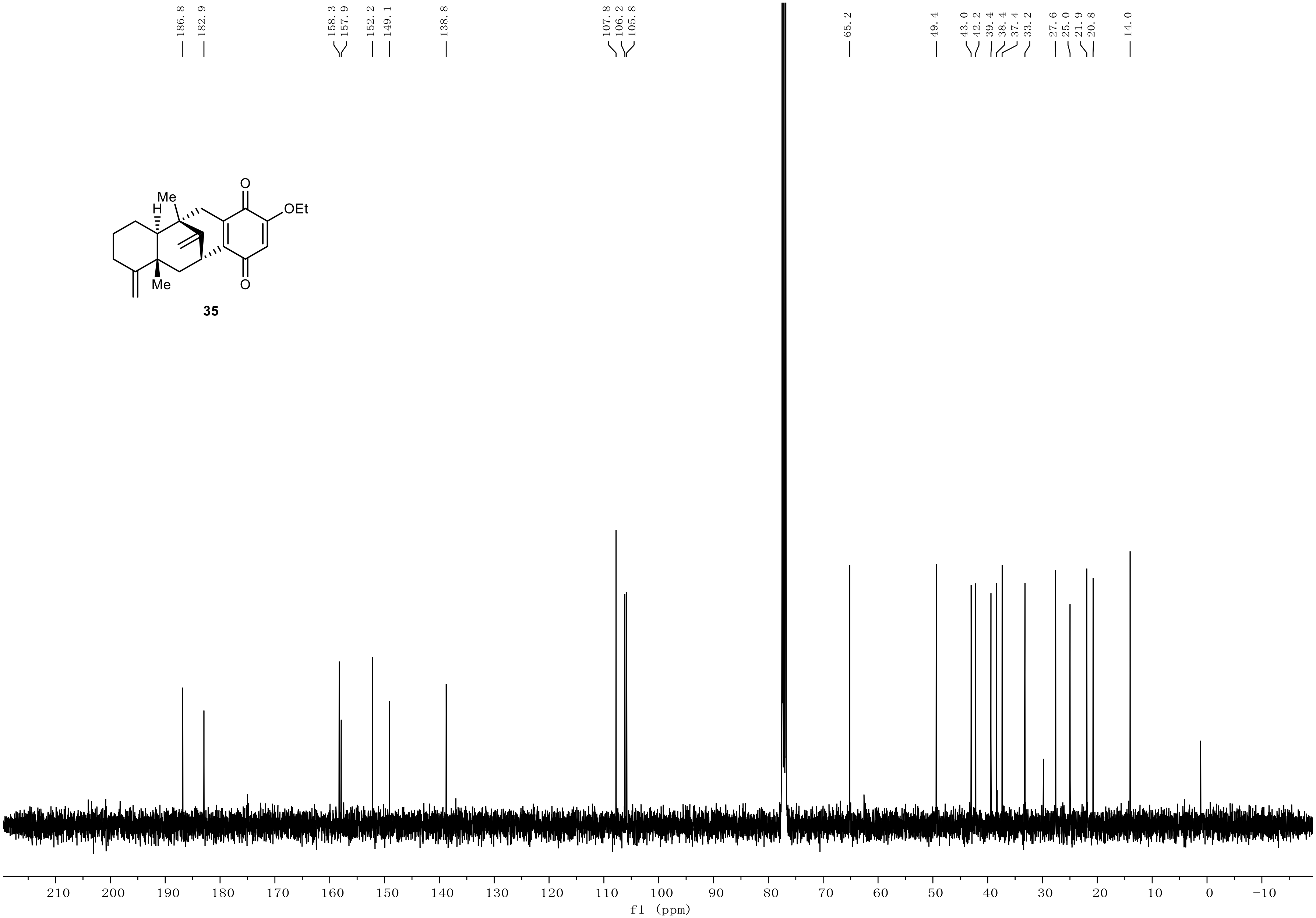

Supplementary Figure 45. ^{1}H NMR Spectrum of Dysidavarone C (9) (400 MHz, $CDCl_3$)

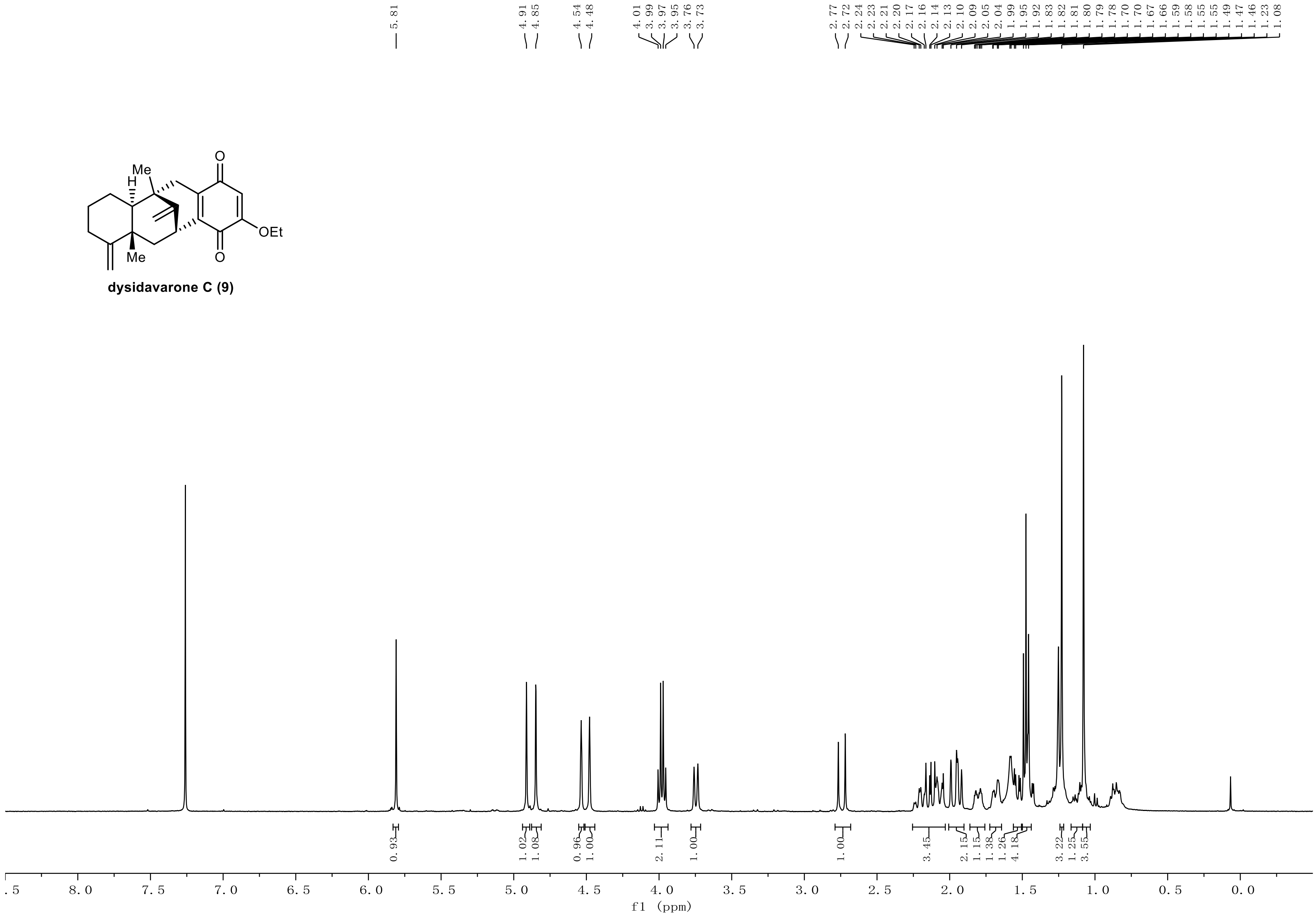

Supplementary Figure 46. ^{13}C NMR Spectrum of Dysidavarone C (9) (101 MHz, CDCl$_3$)

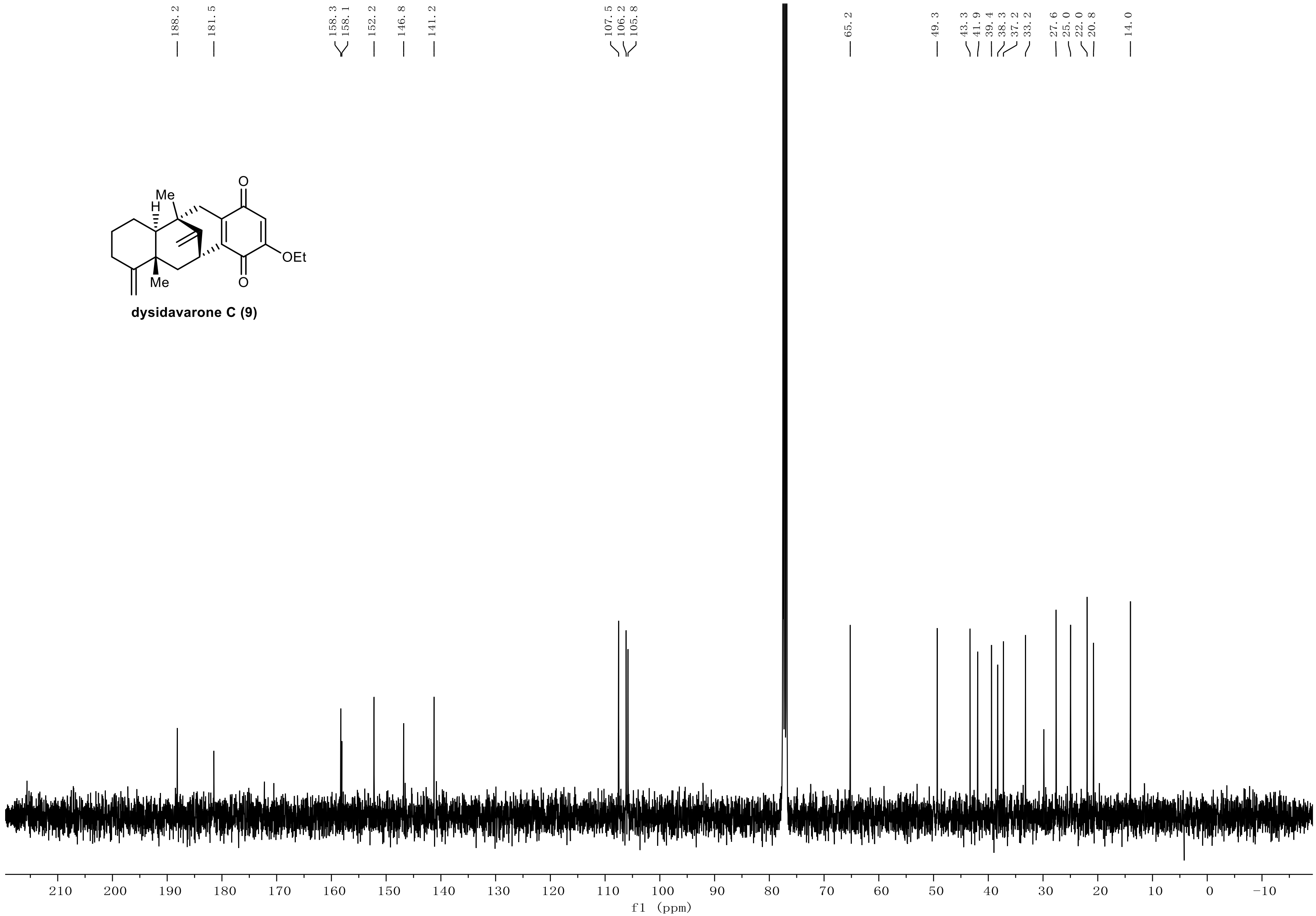

Supplementary Figure 47. ^{1}H NMR Spectrum of Dysidavarone A (7) (400 MHz, $CDCl_3$)

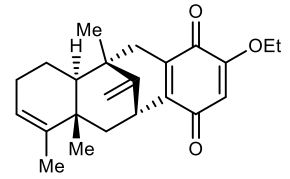

dysidavarone A (7)

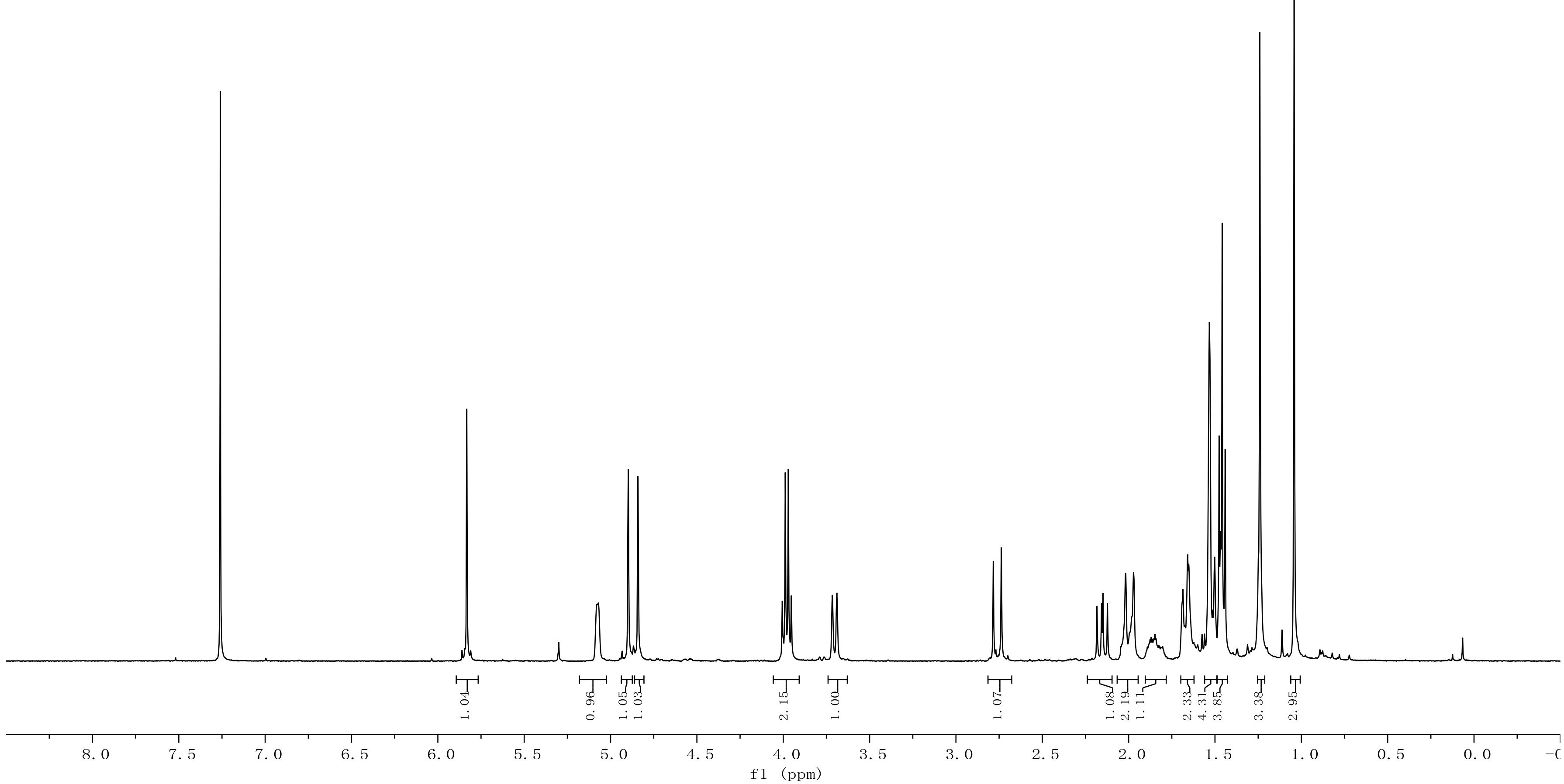

Supplementary Figure 48. ^{13}C NMR Spectrum of Dysidavarone A (7) (151 MHz, $CDCl_3$)

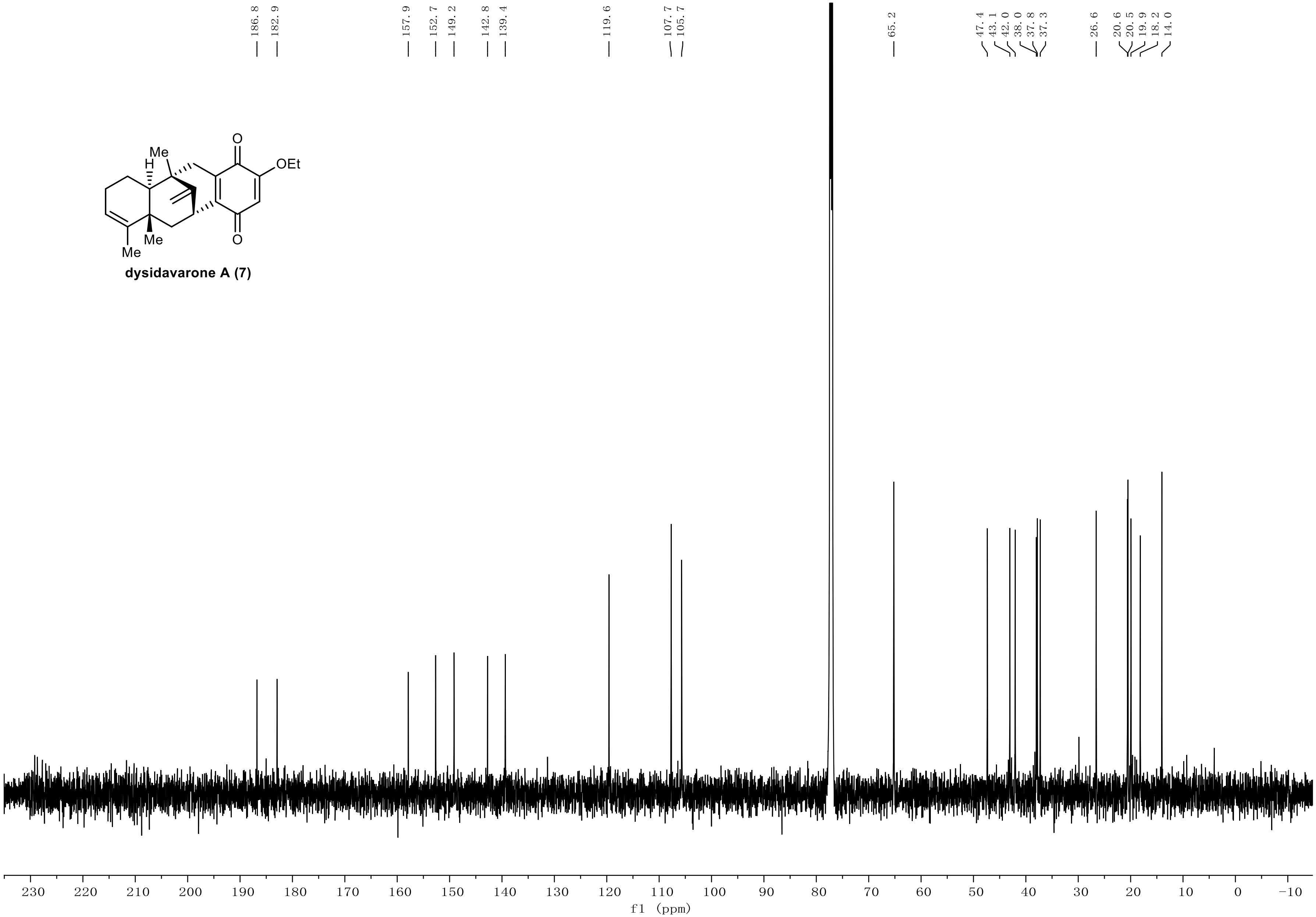

Supplementary Figure 49. ^{1}H NMR Spectrum of Dysidavarone B (8) (600 MHz, $CDCl_3$)

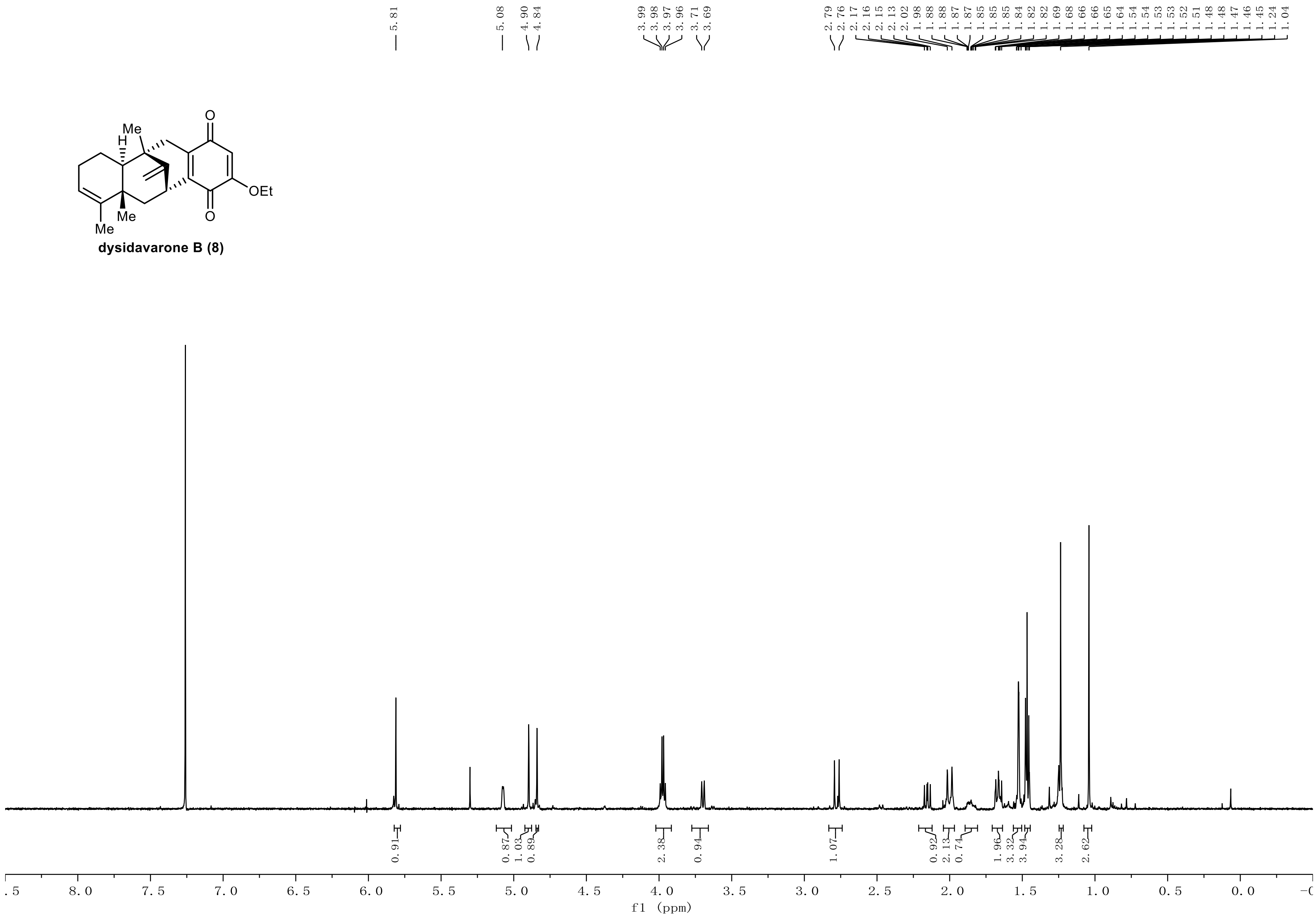

Supplementary Figure 50. ^{13}C NMR Spectrum of Dysidavarone B (8) (151 MHz, $CDCl_3$)

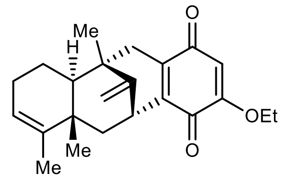

dysidavarone B (8)

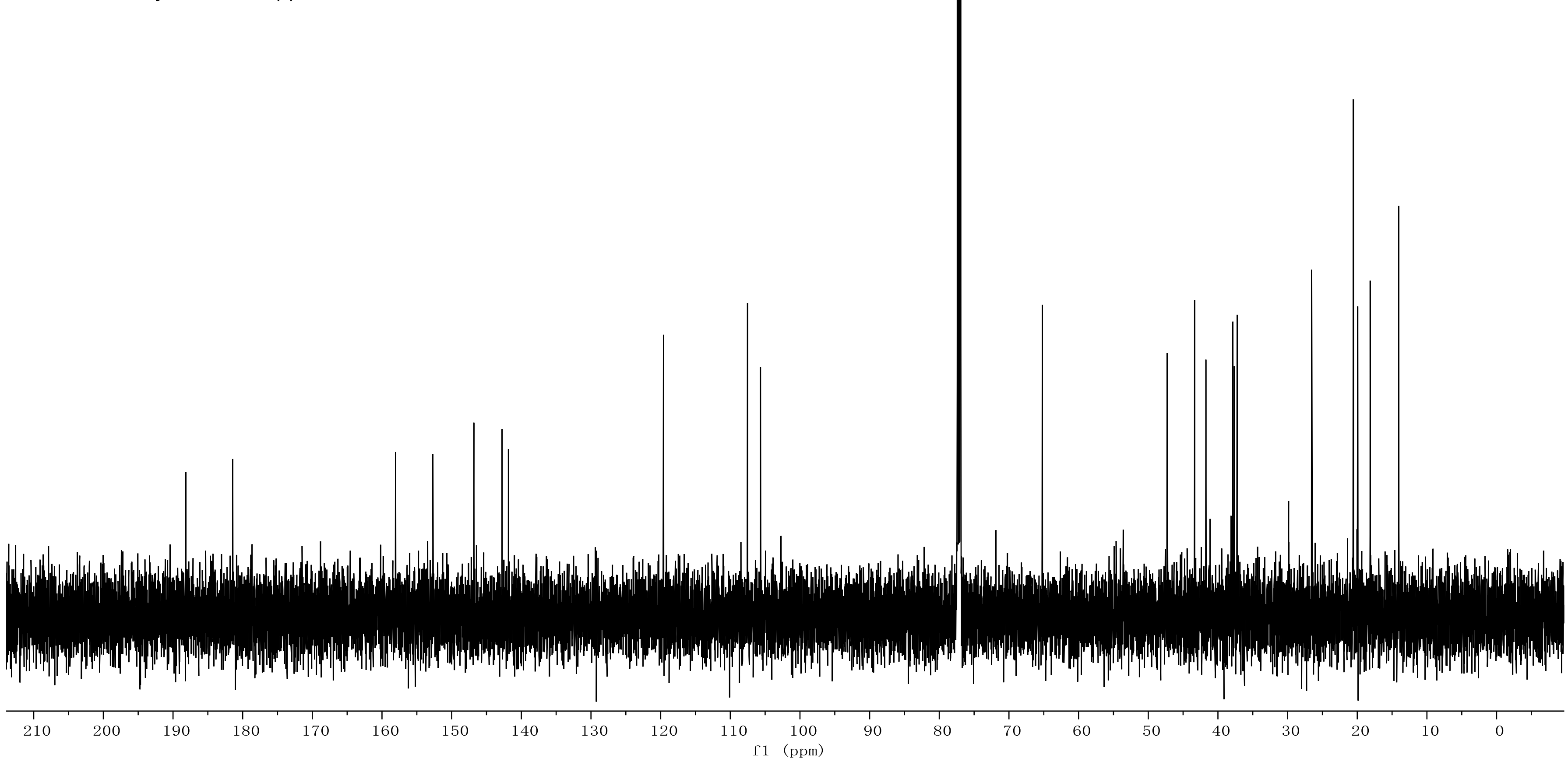

Supplementary Figure 51. ^{1}H NMR Spectrum of 38 (600 MHz, $CDCl_3$)

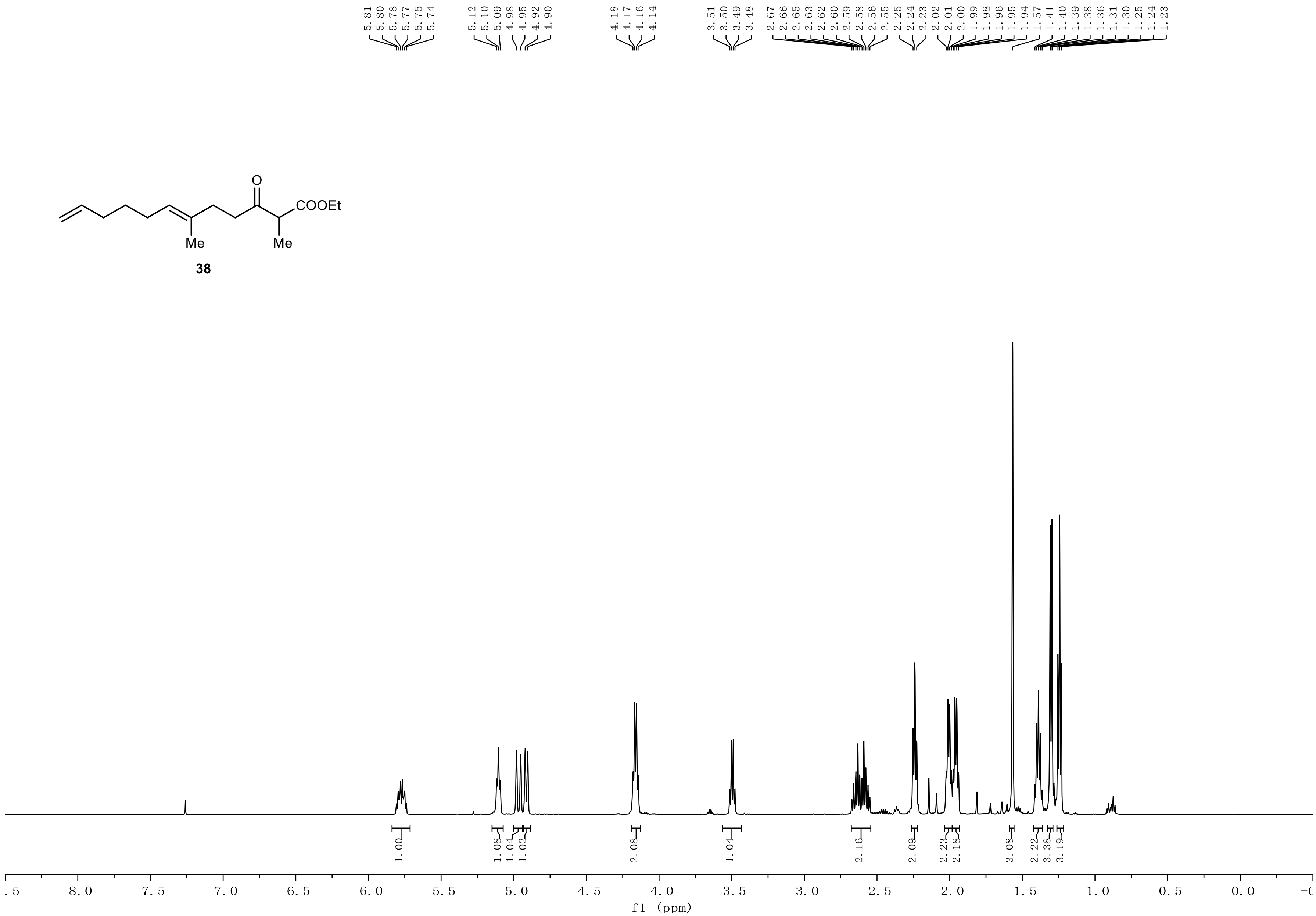

Supplementary Figure 52. ^{13}C NMR Spectrum of 38 (151 MHz, $CDCl_3$)

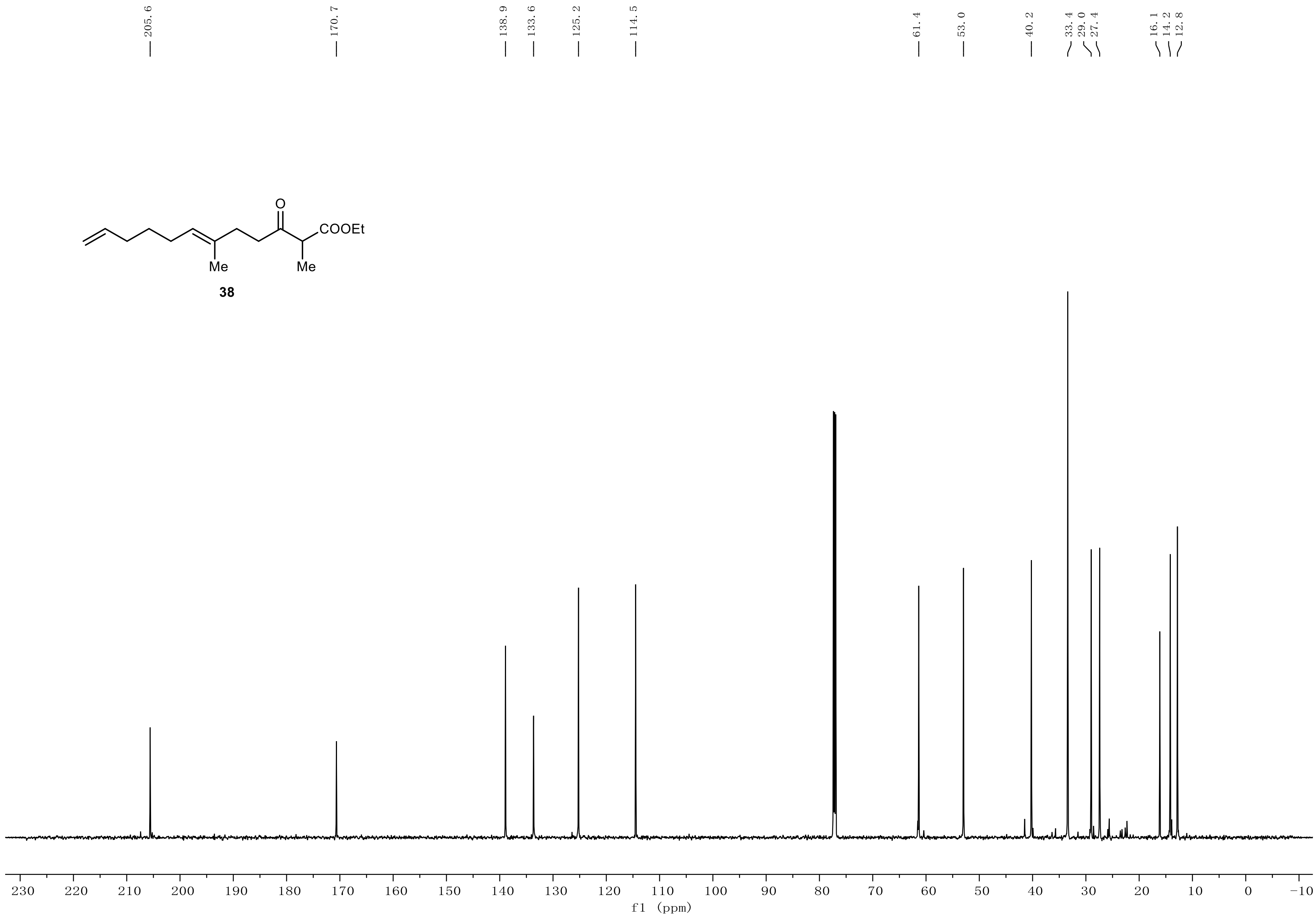

Supplementary Figure 53. ^{1}H NMR Spectrum of 39 (400 MHz, $CDCl_3$)

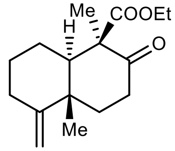

39

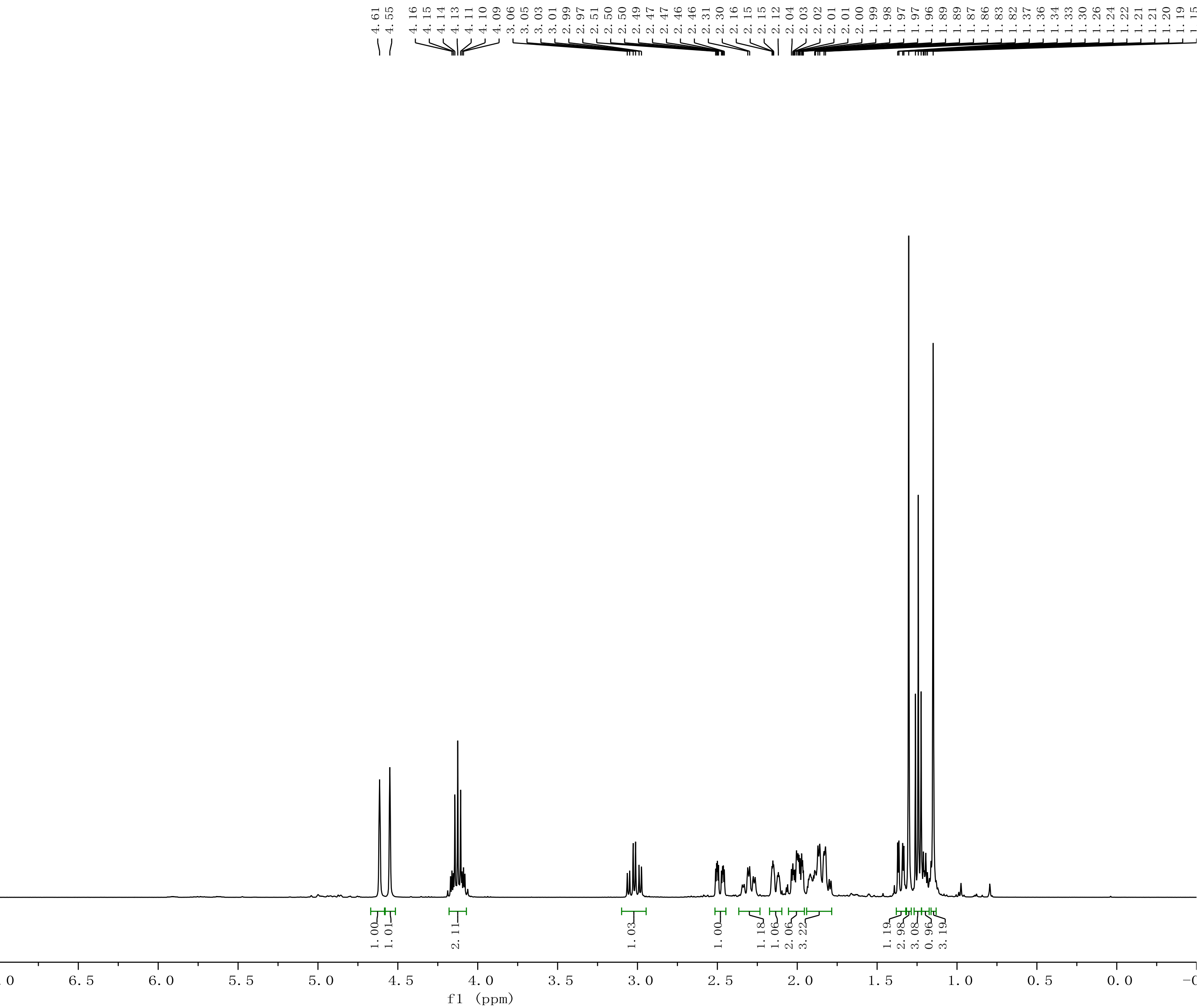

Supplementary Figure 54. ^{13}C NMR Spectrum of 39 (101 MHz, $CDCl_3$)

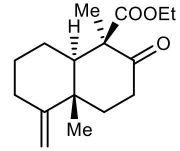

39

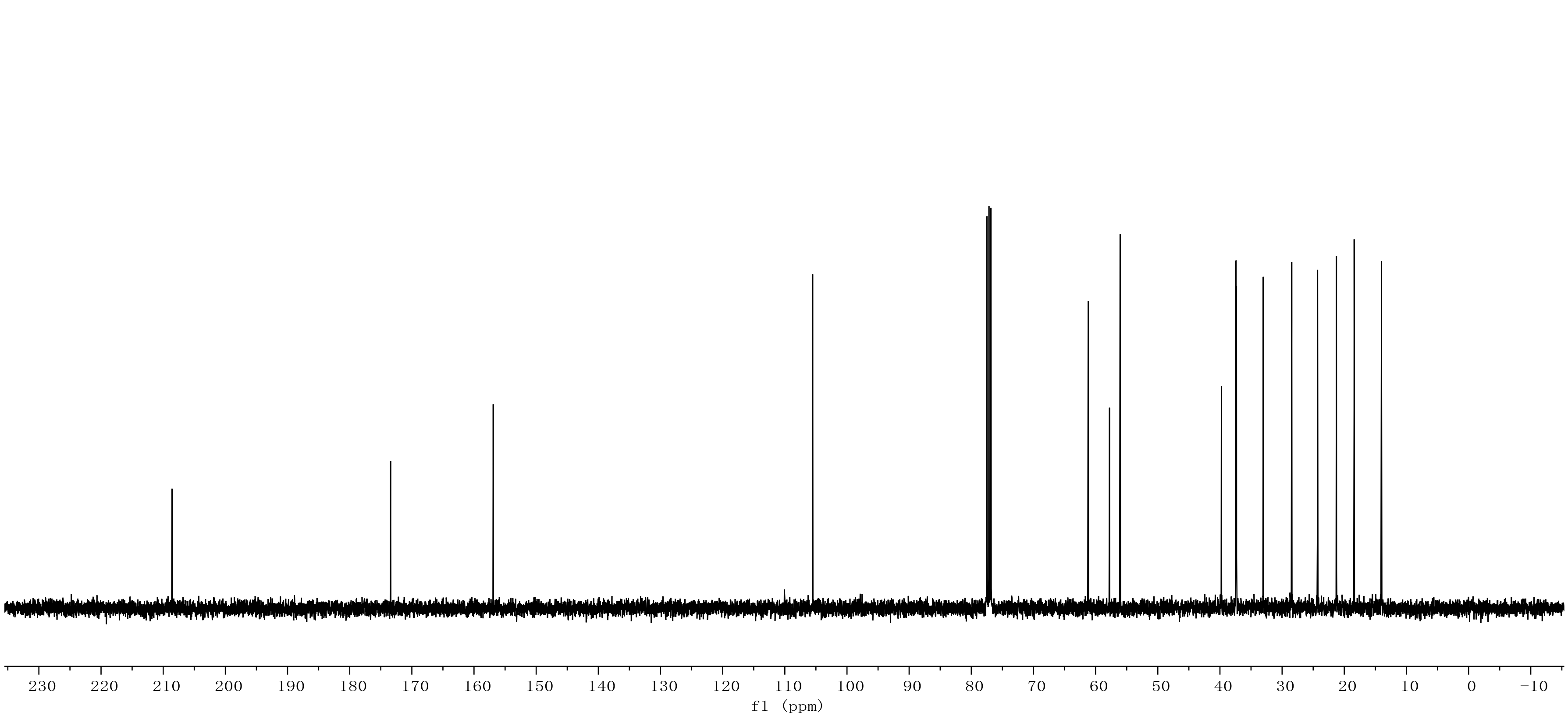

Supplementary Figure 55. ¹H NMR Spectrum of SI-10 (400 MHz, $CDCl_3$)

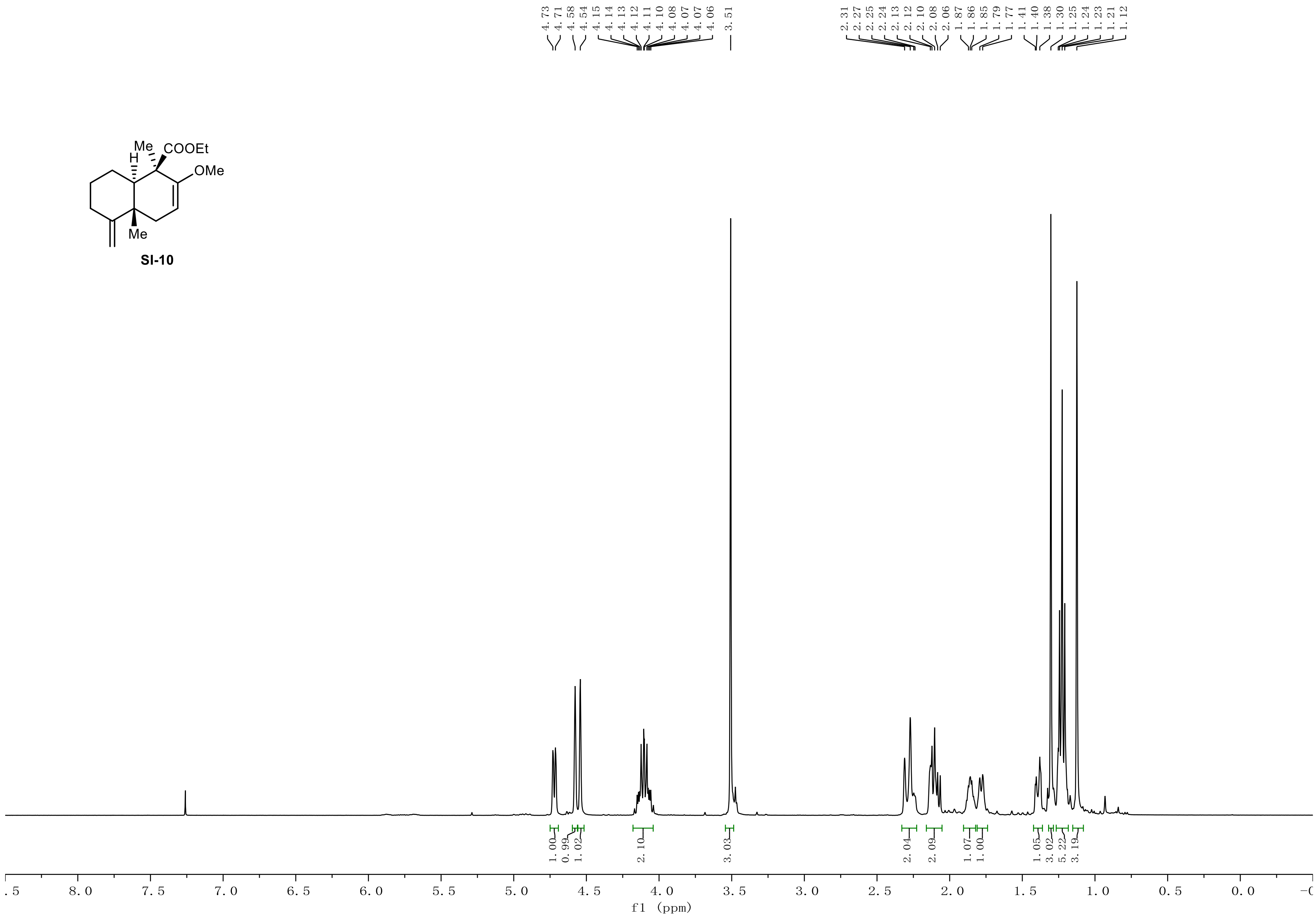

Supplementary Figure 56. ^{13}C NMR Spectrum of SI-10 (101 MHz, $CDCl_3$)

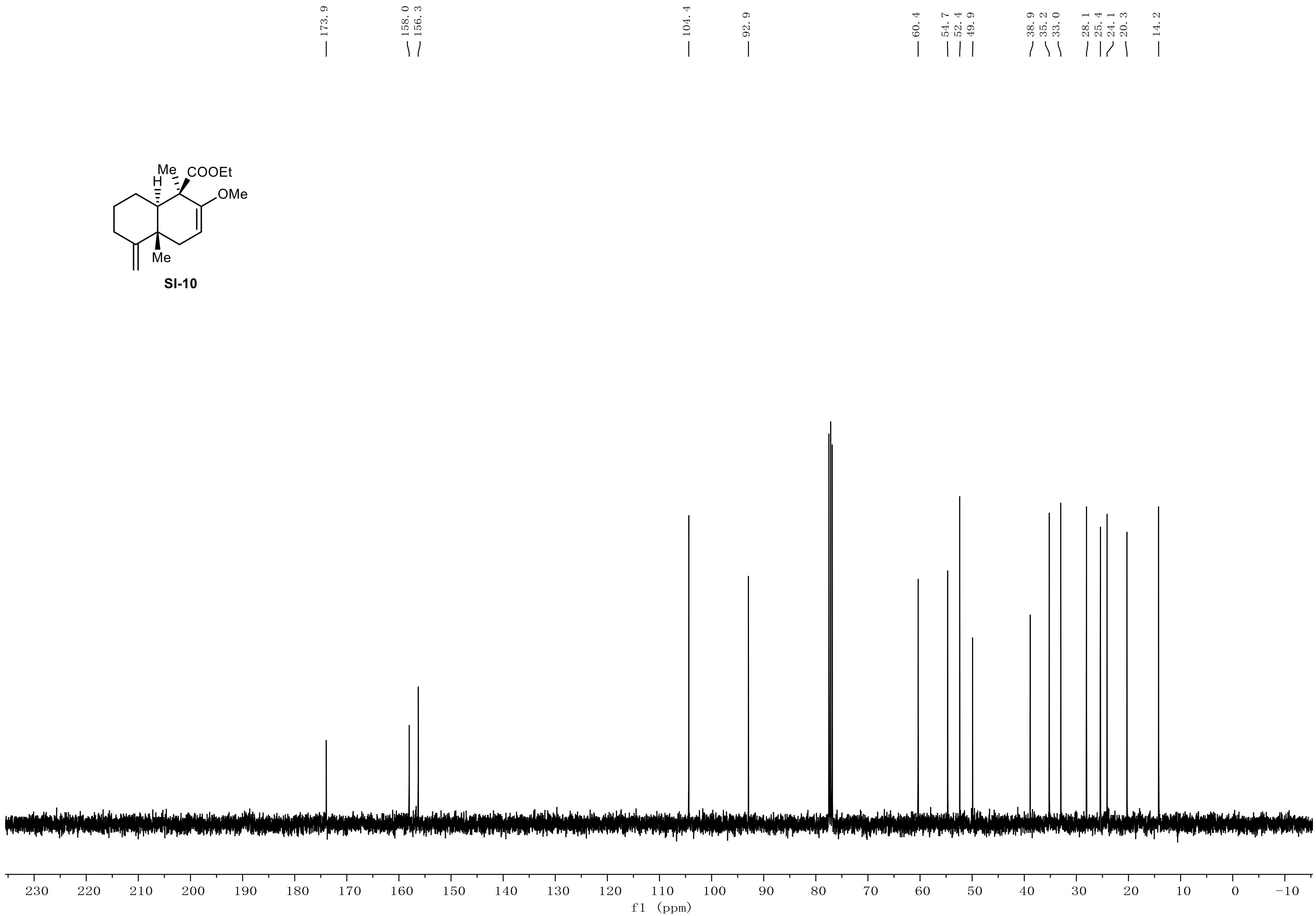

Supplementary Figure 57. ^{1}H NMR Spectrum of SI-11 (600 MHz, $CDCl_3$)

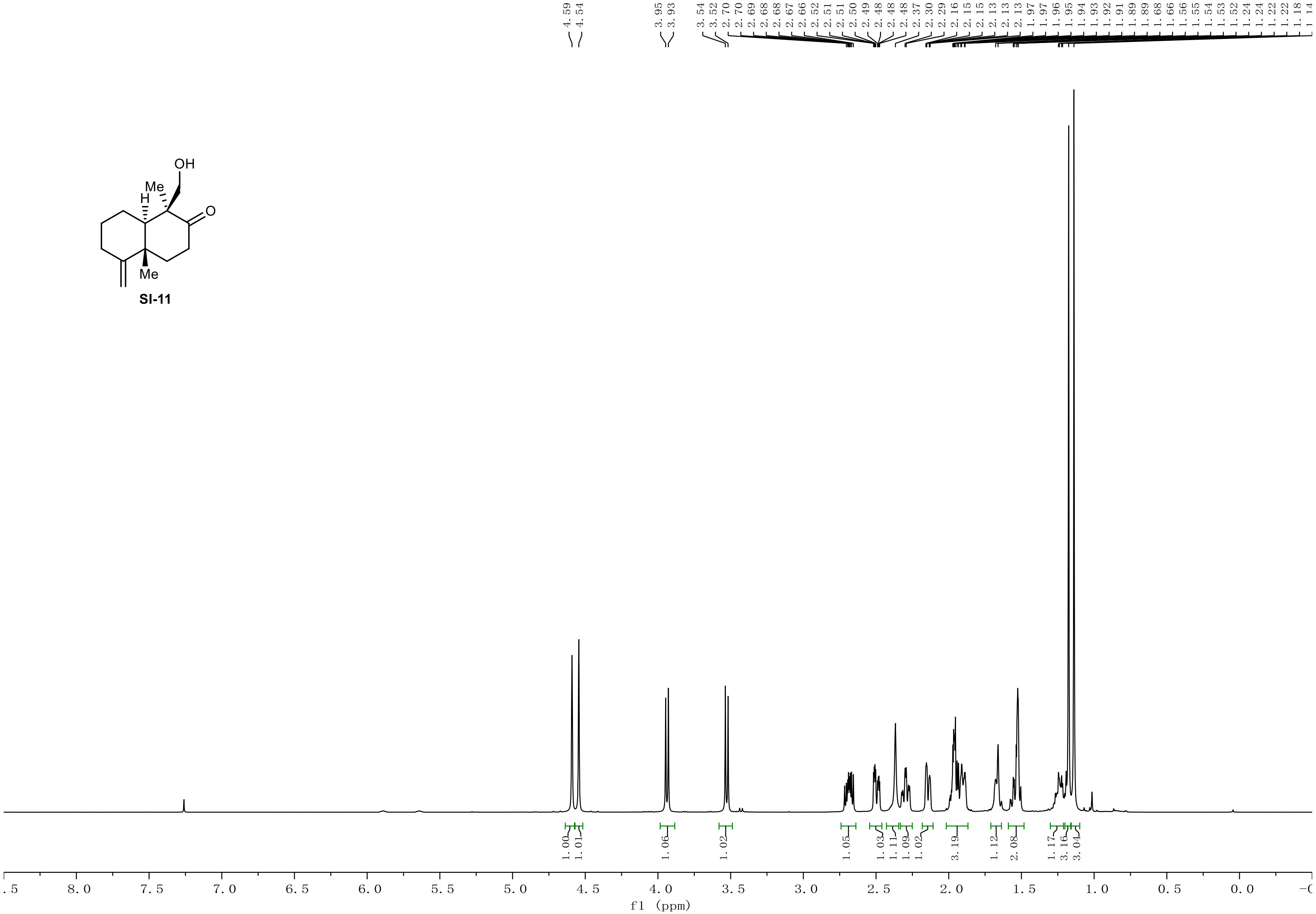

Supplementary Figure 58. ^{13}C NMR Spectrum of SI-11 (151 MHz, $CDCl_3$)

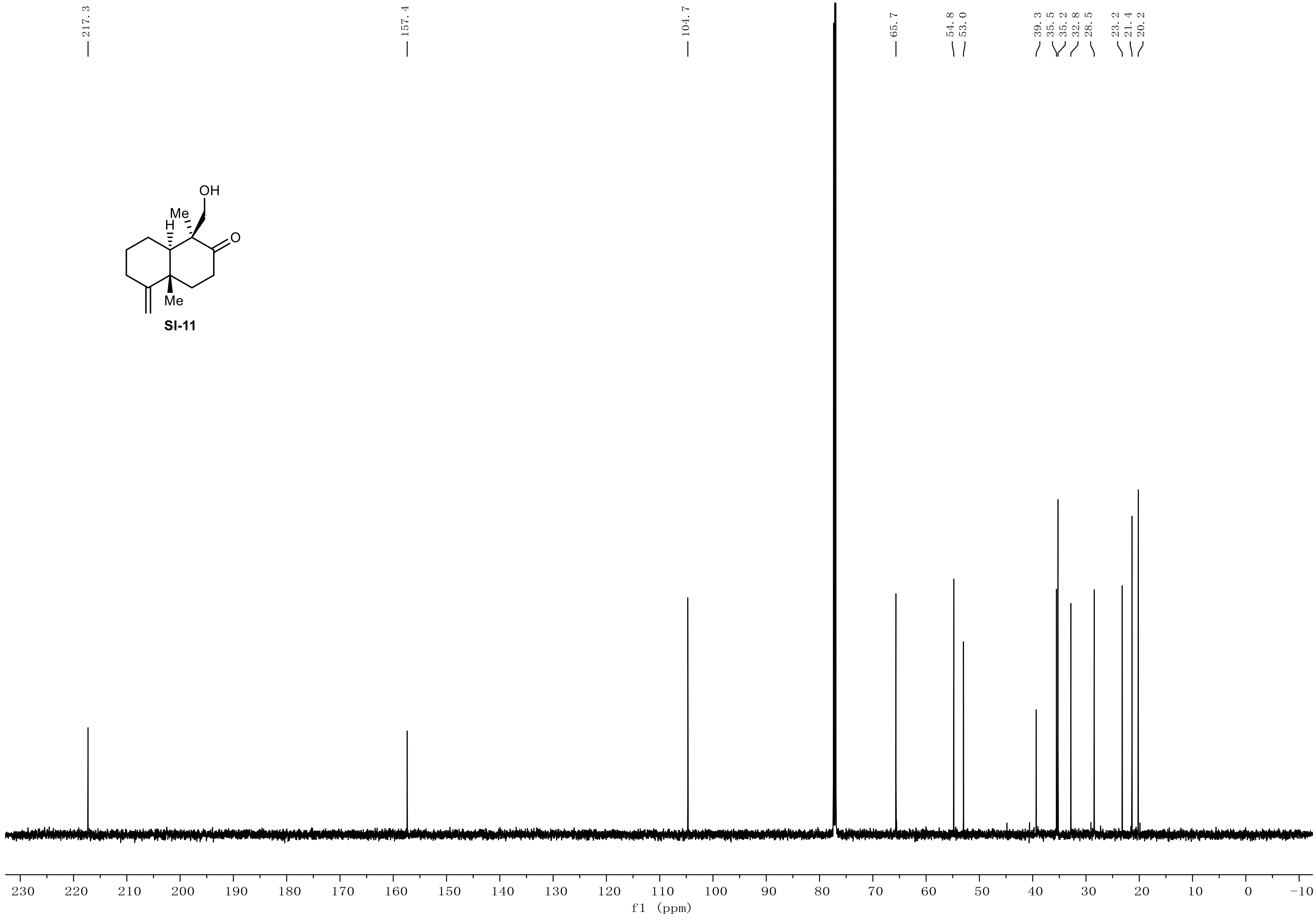

Supplementary Figure 59. ^{1}H NMR Spectrum of 40 (400 MHz, $CDCl_3$)

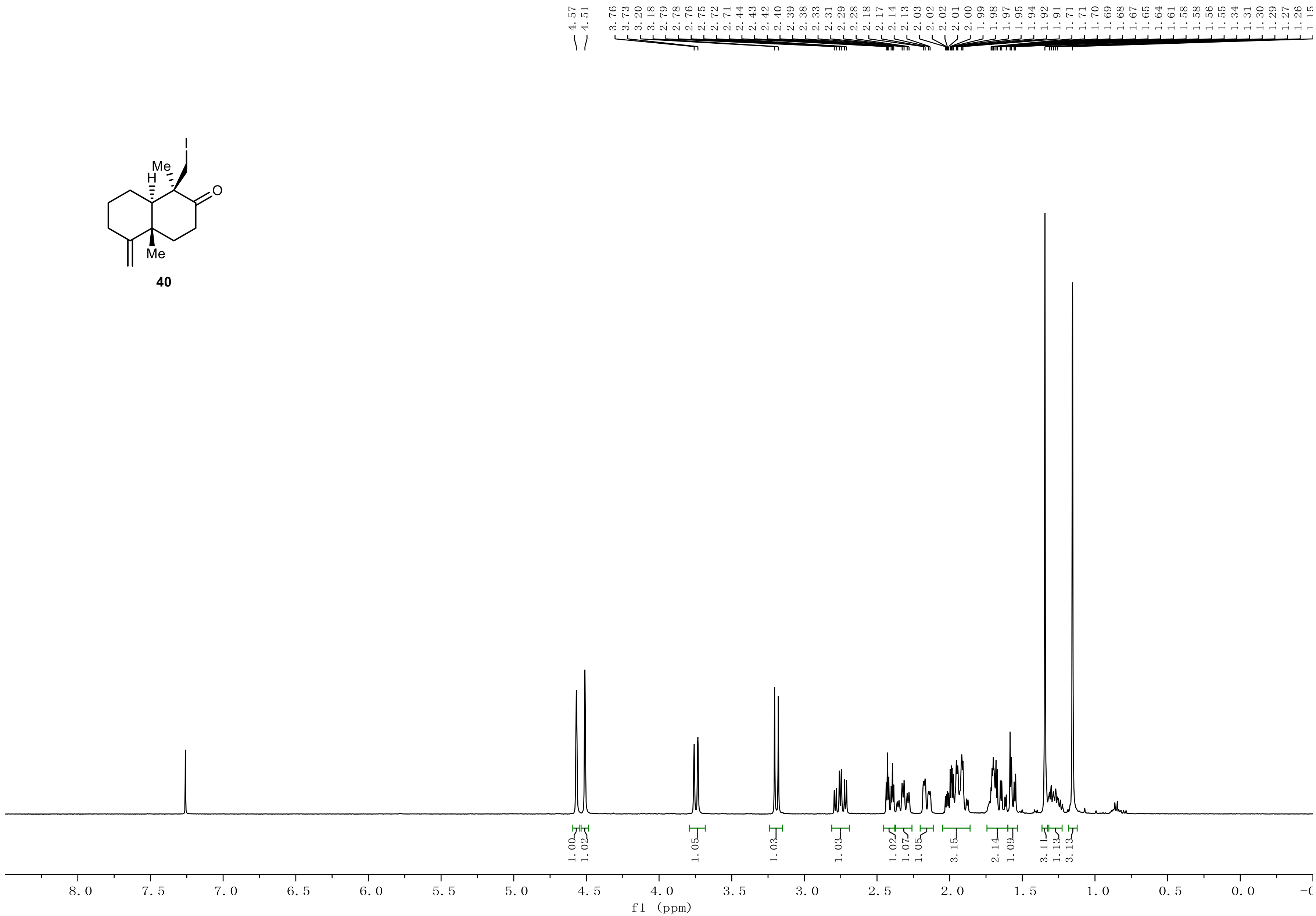

Supplementary Figure 60. ^{13}C NMR Spectrum of 40 (151 MHz, $CDCl_3$)

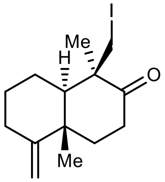

40

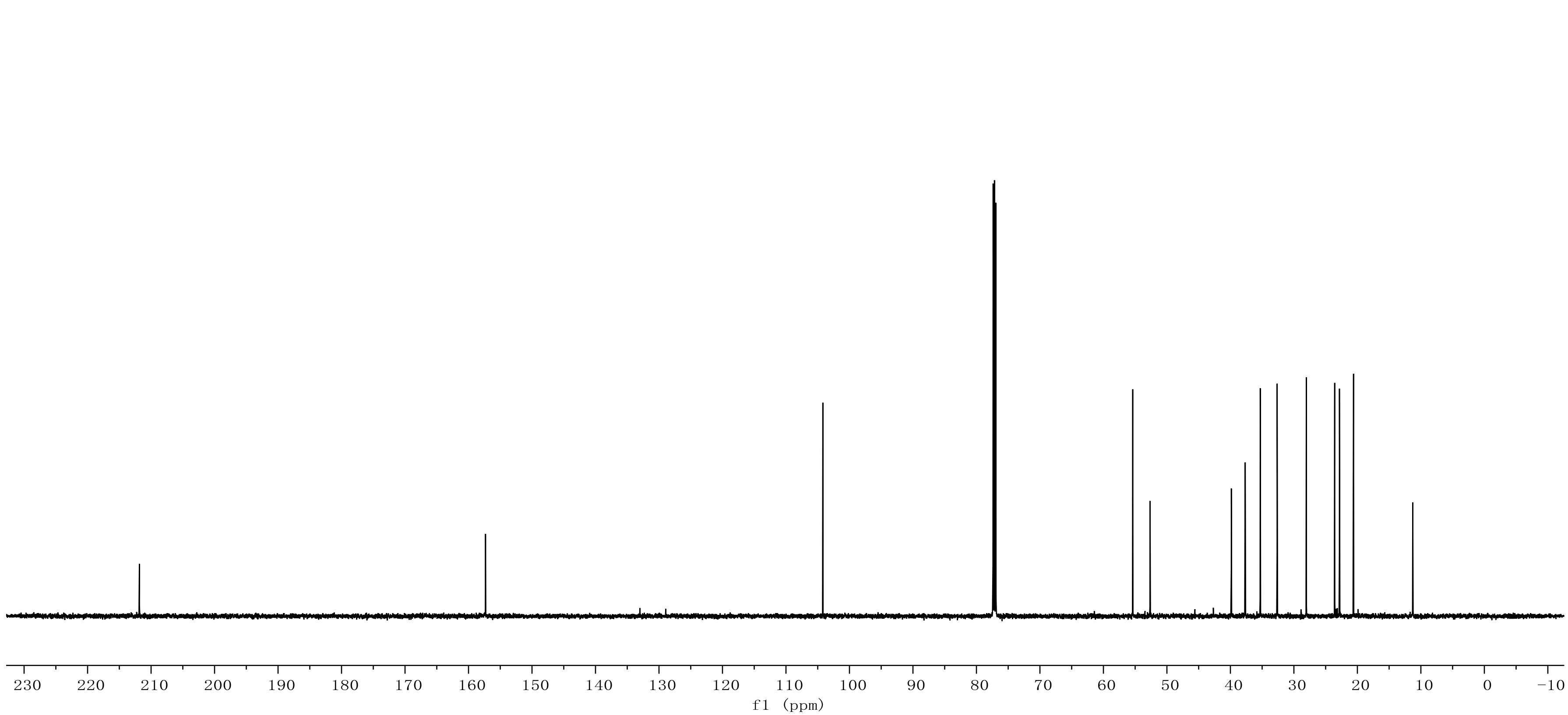

Supplementary Figure 61. ^{1}H NMR Spectrum of 41 (400 MHz, $CDCl_3$)

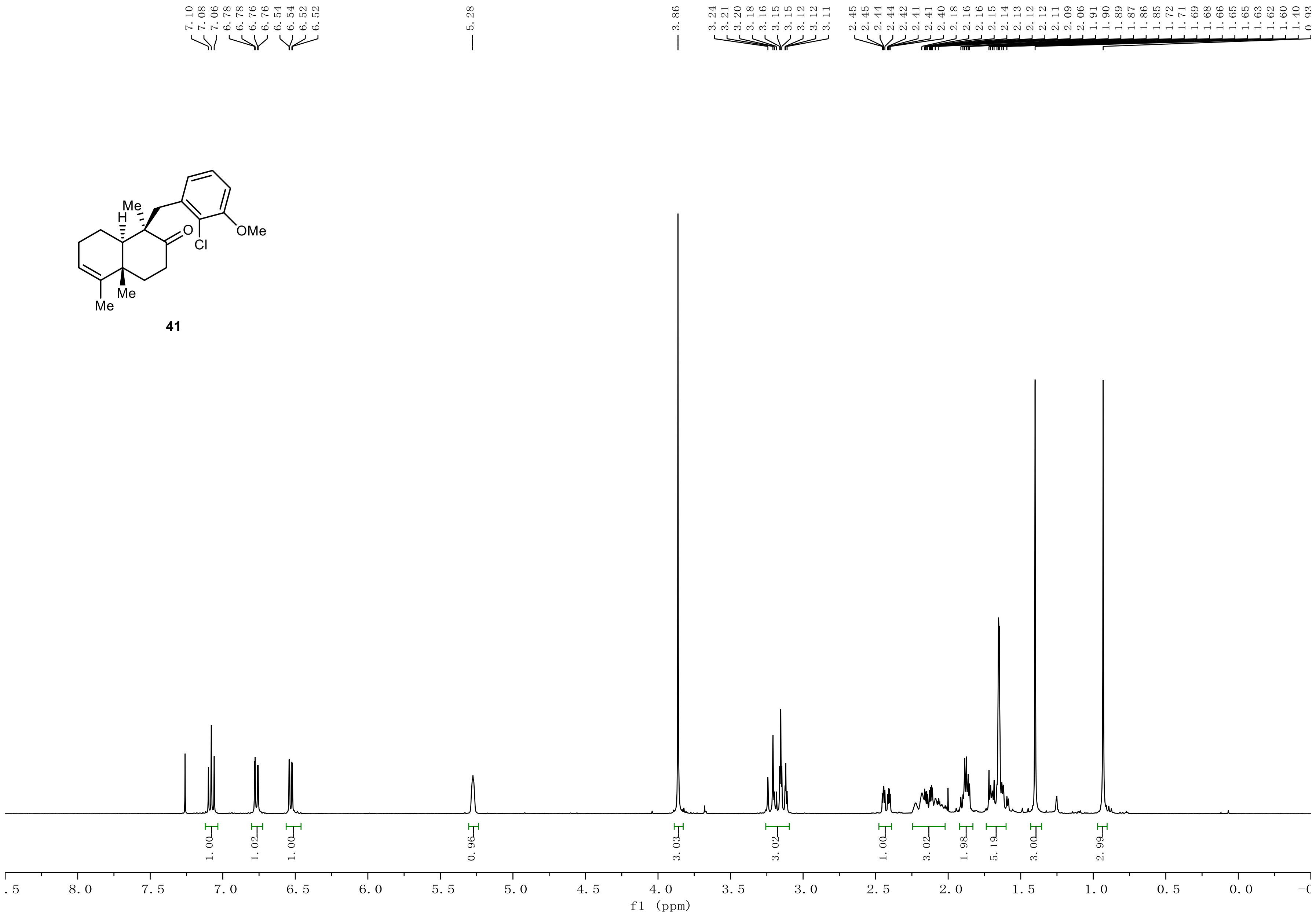

Supplementary Figure 62. ^{13}C NMR Spectrum of 41 (101 MHz, $CDCl_3$)

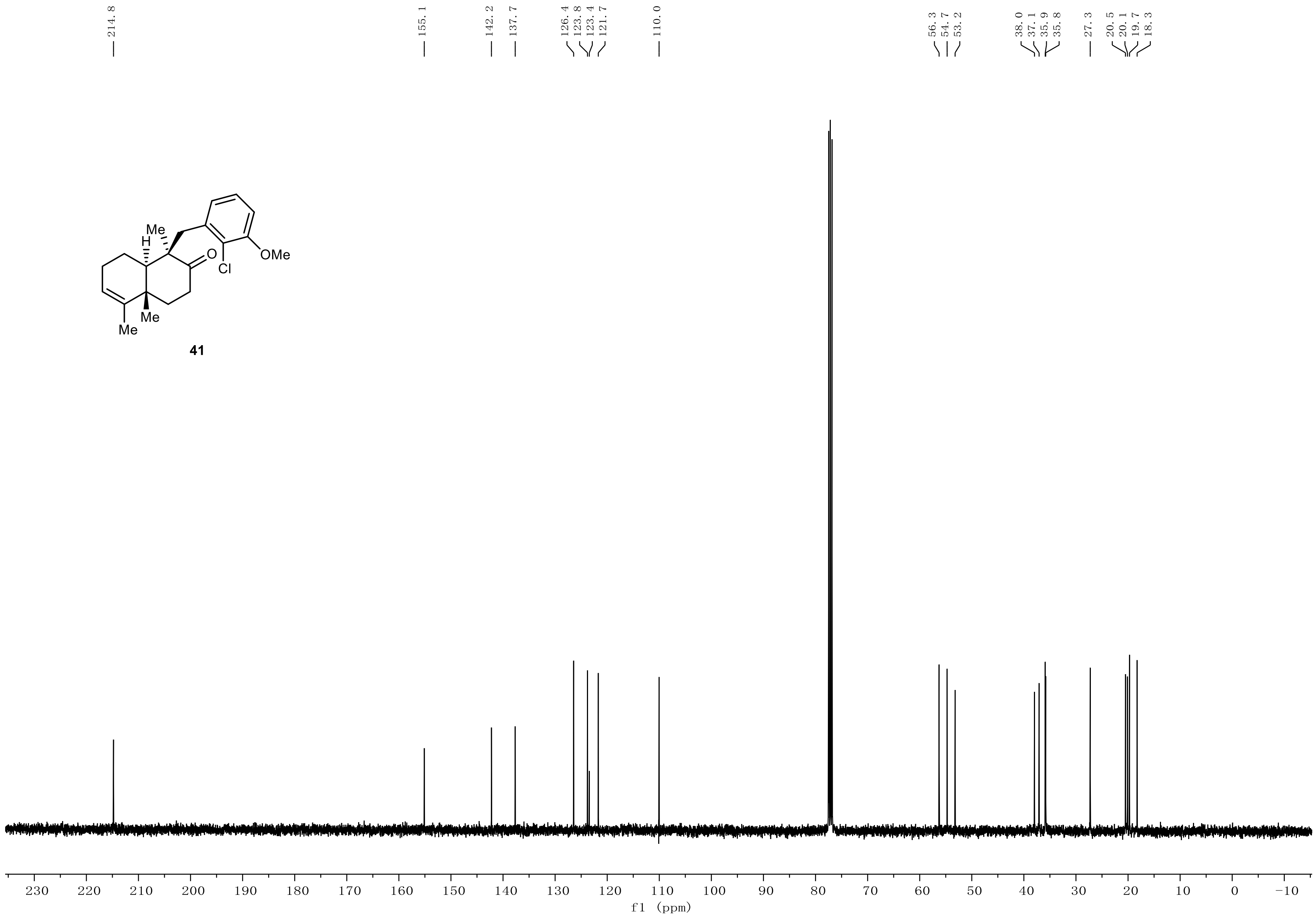

Supplementary Figure 63. ^{1}H NMR Spectrum of SI-12 (600 MHz, $CDCl_3$)

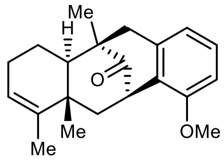

SI-12

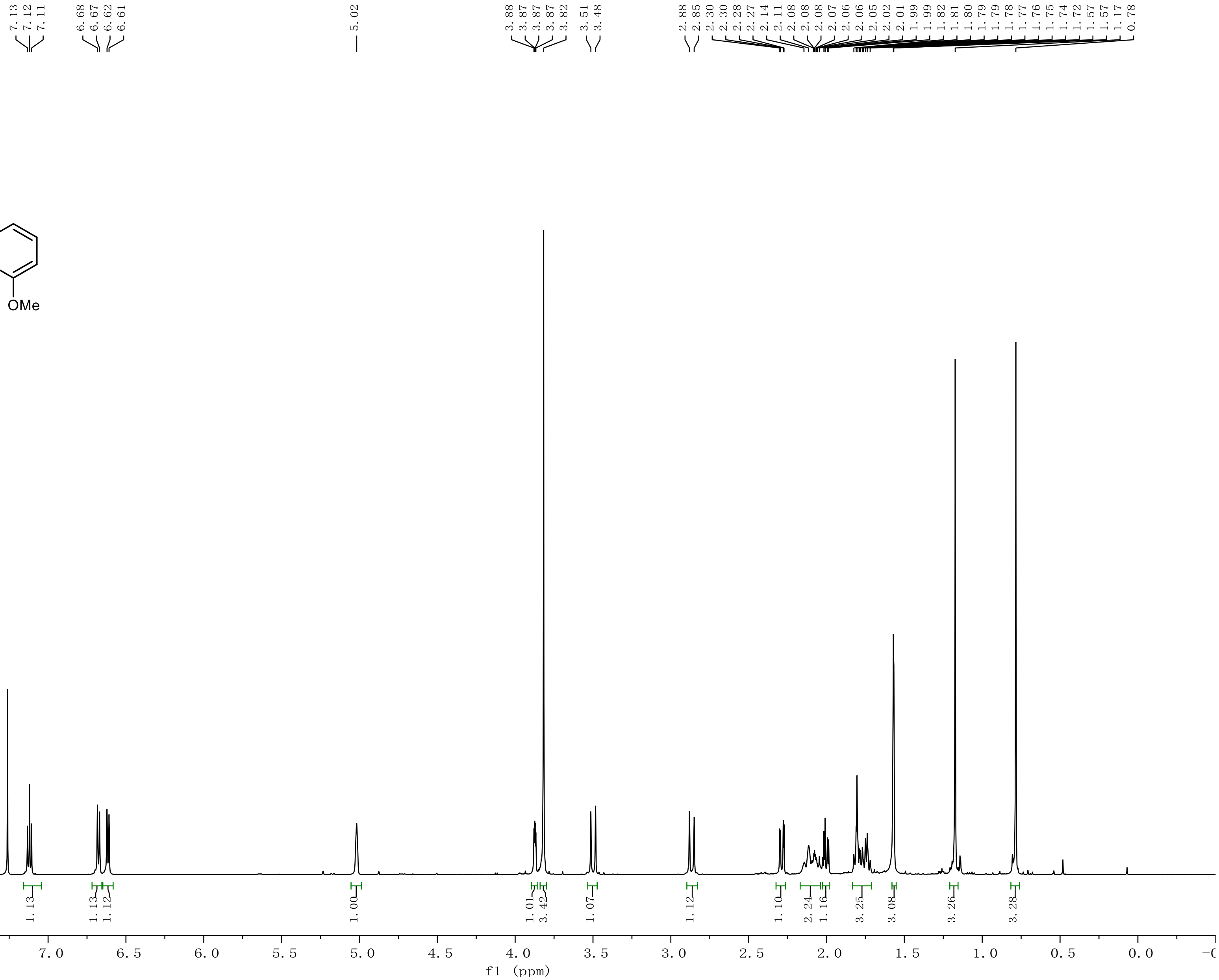

Supplementary Figure 64. ^{13}C NMR Spectrum of SI-12 (151 MHz, $CDCl_3$)

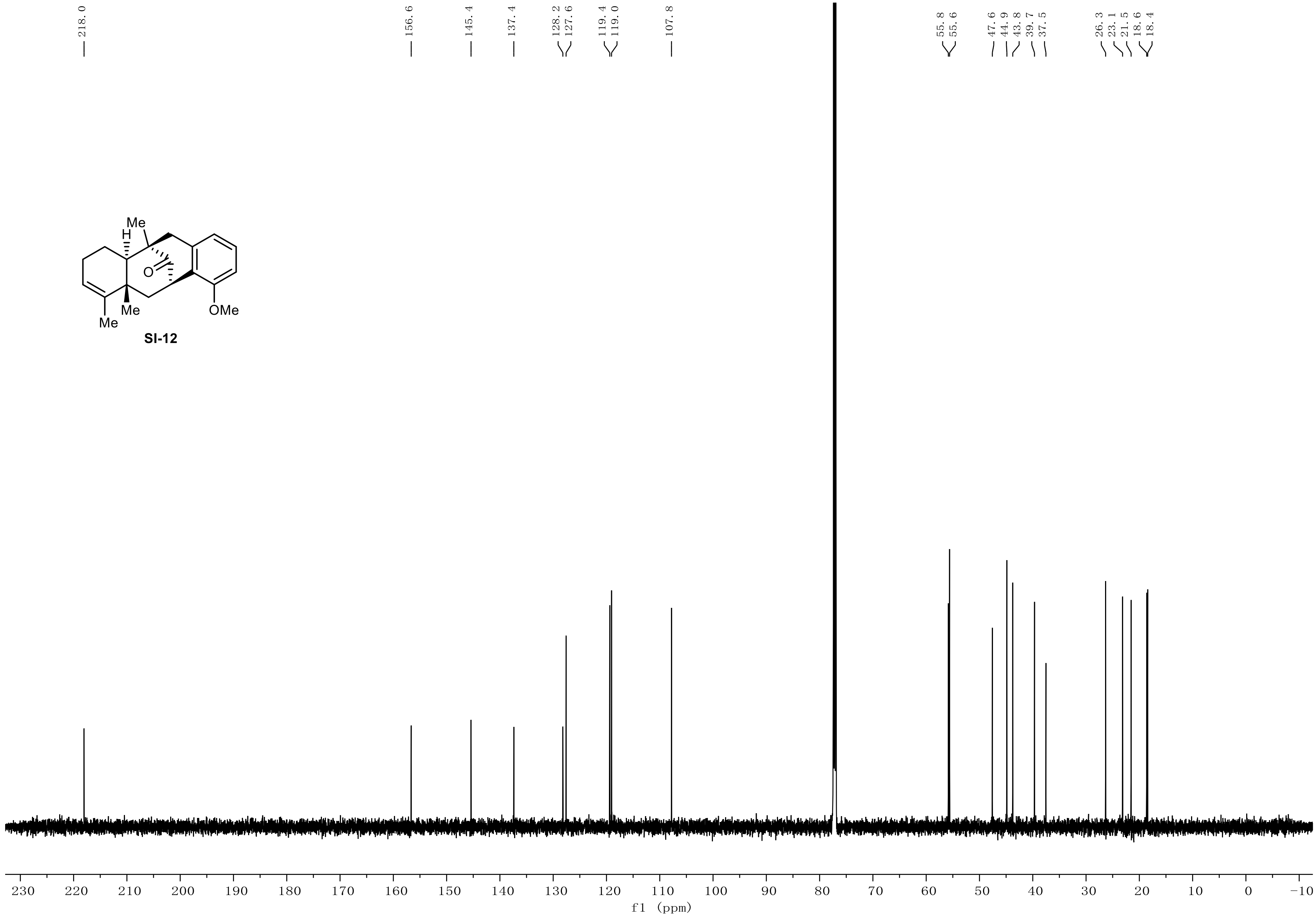

Supplementary Figure 65. ^{1}H NMR Spectrum of SI-13 (400 MHz, $CDCl_3$)

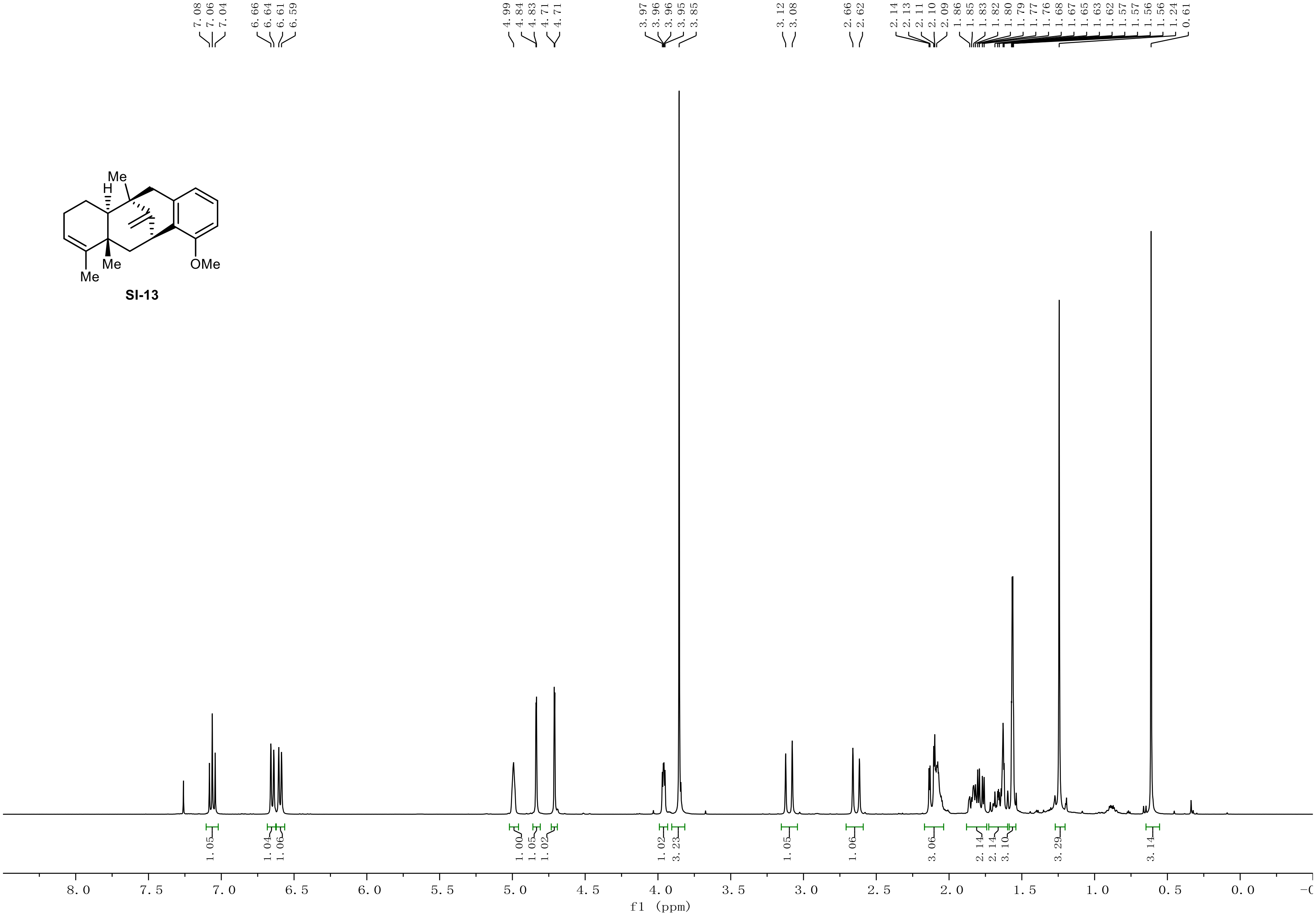

Supplementary Figure 66. ^{13}C NMR Spectrum of SI-13 (101 MHz, $CDCl_3$)

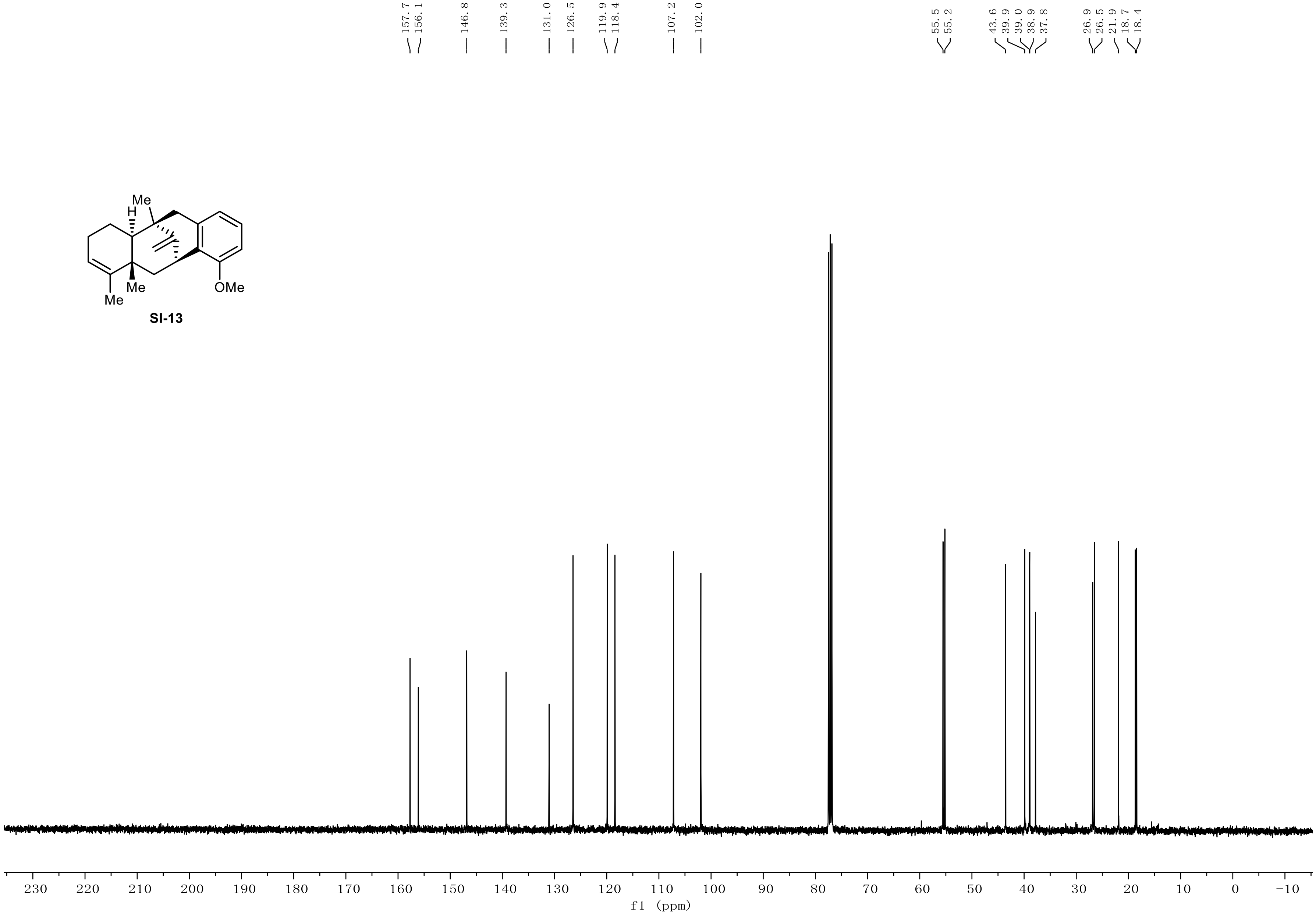

Supplementary Figure 67. ^{1}H NMR Spectrum of SI-14 (600 MHz, $CDCl_3$)

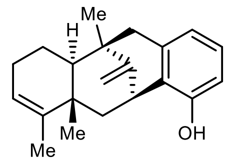

SI-14

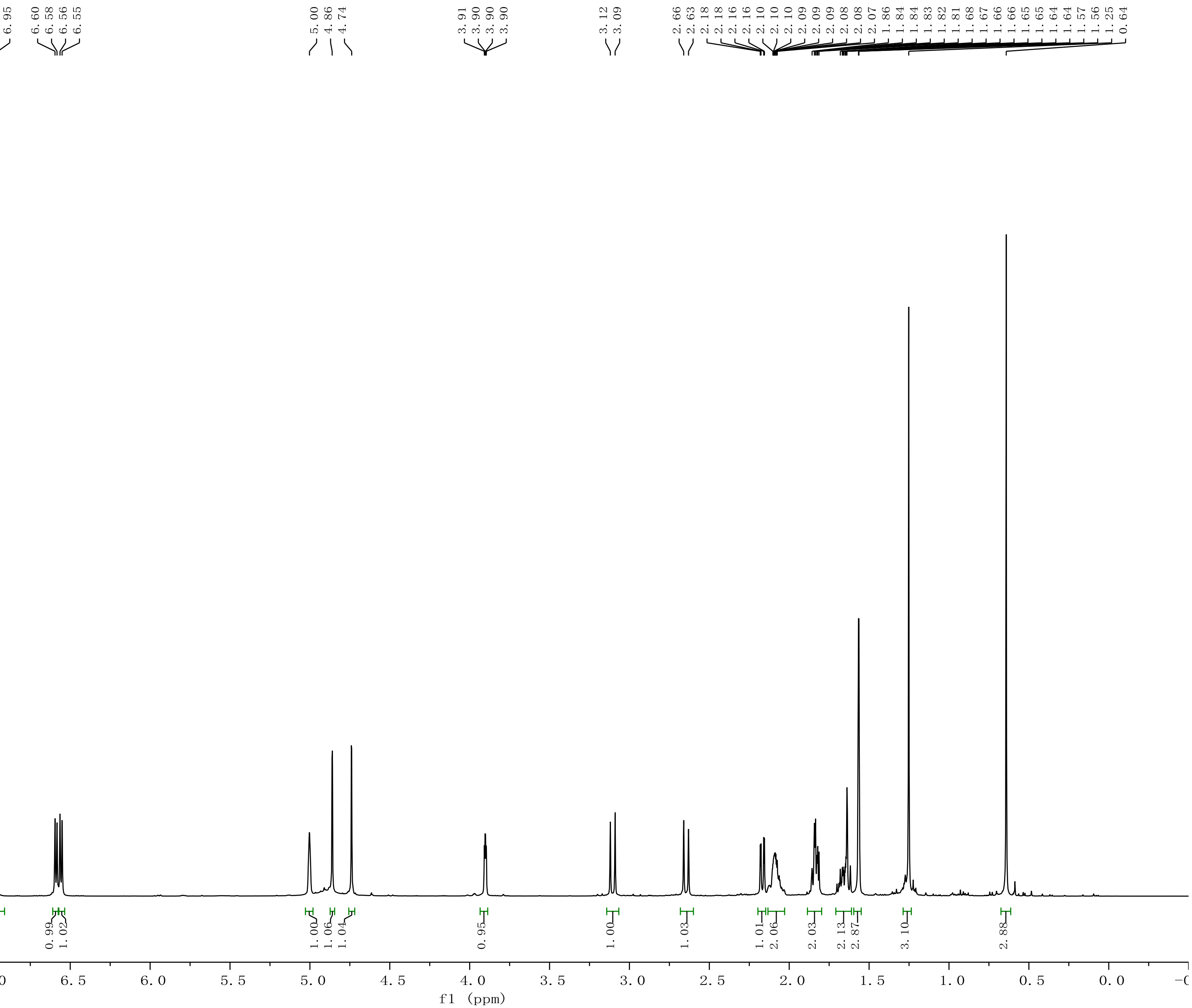

Supplementary Figure 68. ^{13}C NMR Spectrum of SI-14 (151 MHz, $CDCl_3$)

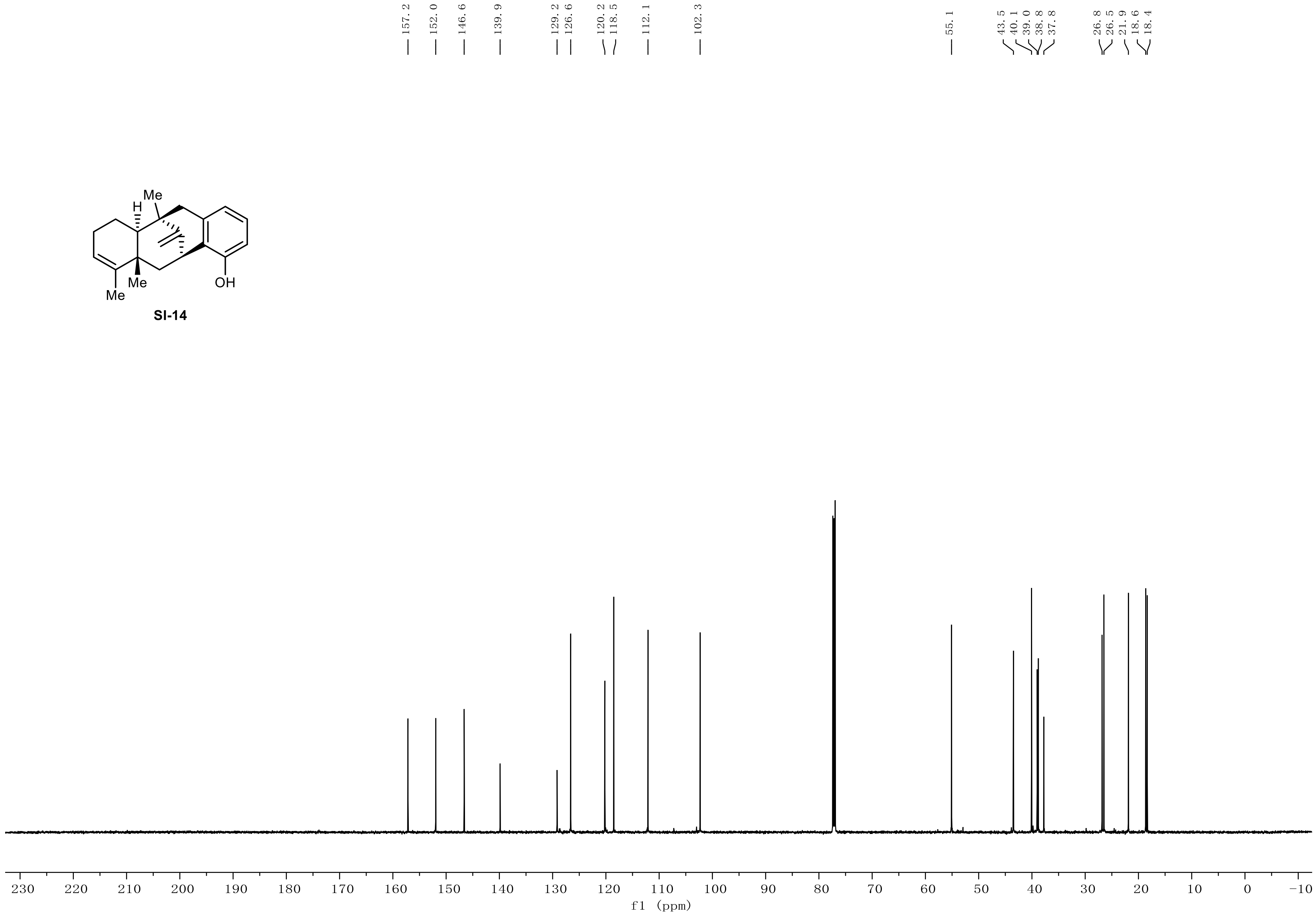

SI-14

Supplementary Figure 69. ^{1}H NMR Spectrum of 42 (400 MHz, $CDCl_3$)

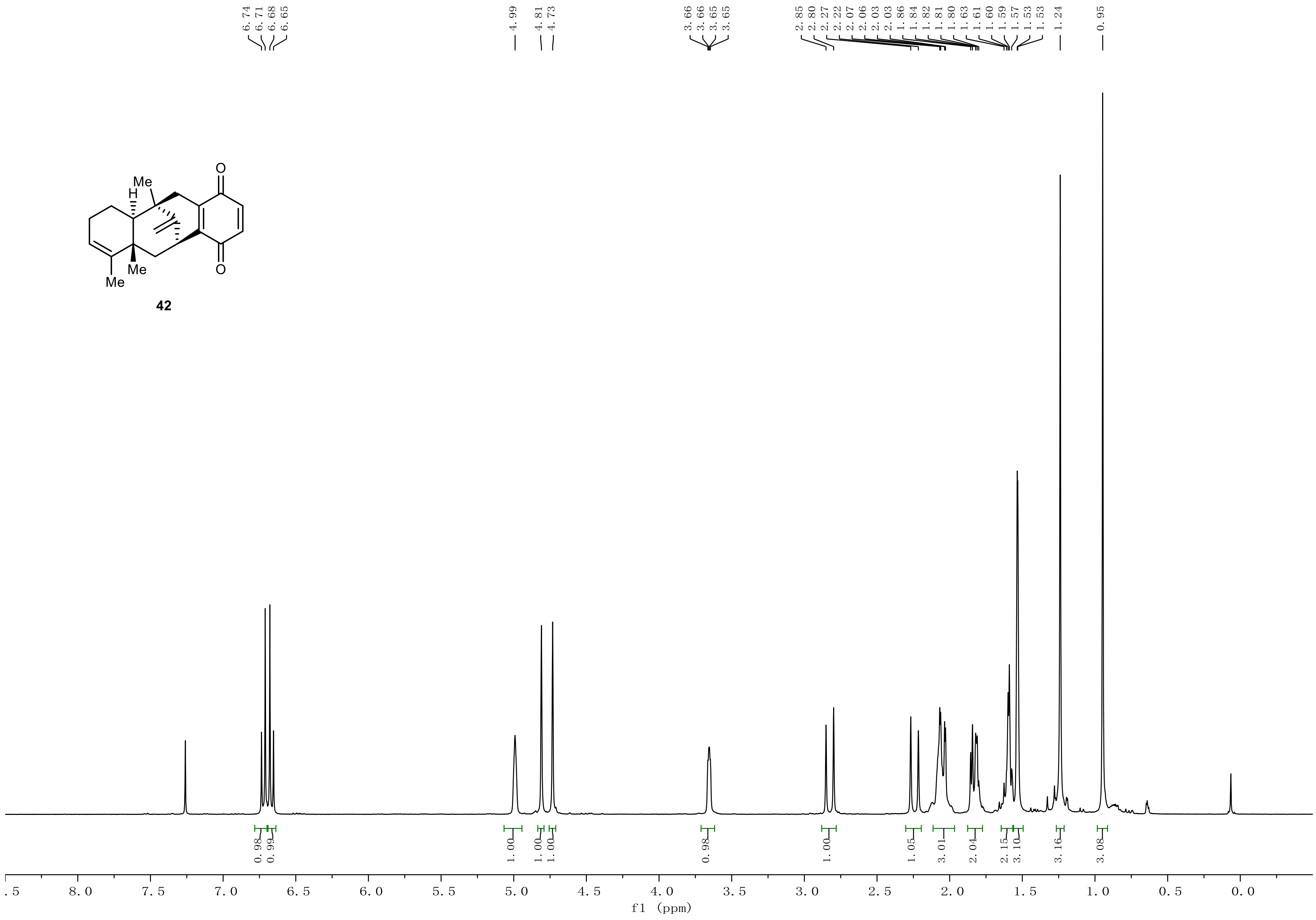

Supplementary Figure 70. ^{13}**C NMR Spectrum of 42 (101 MHz, CDCl$_3$)**

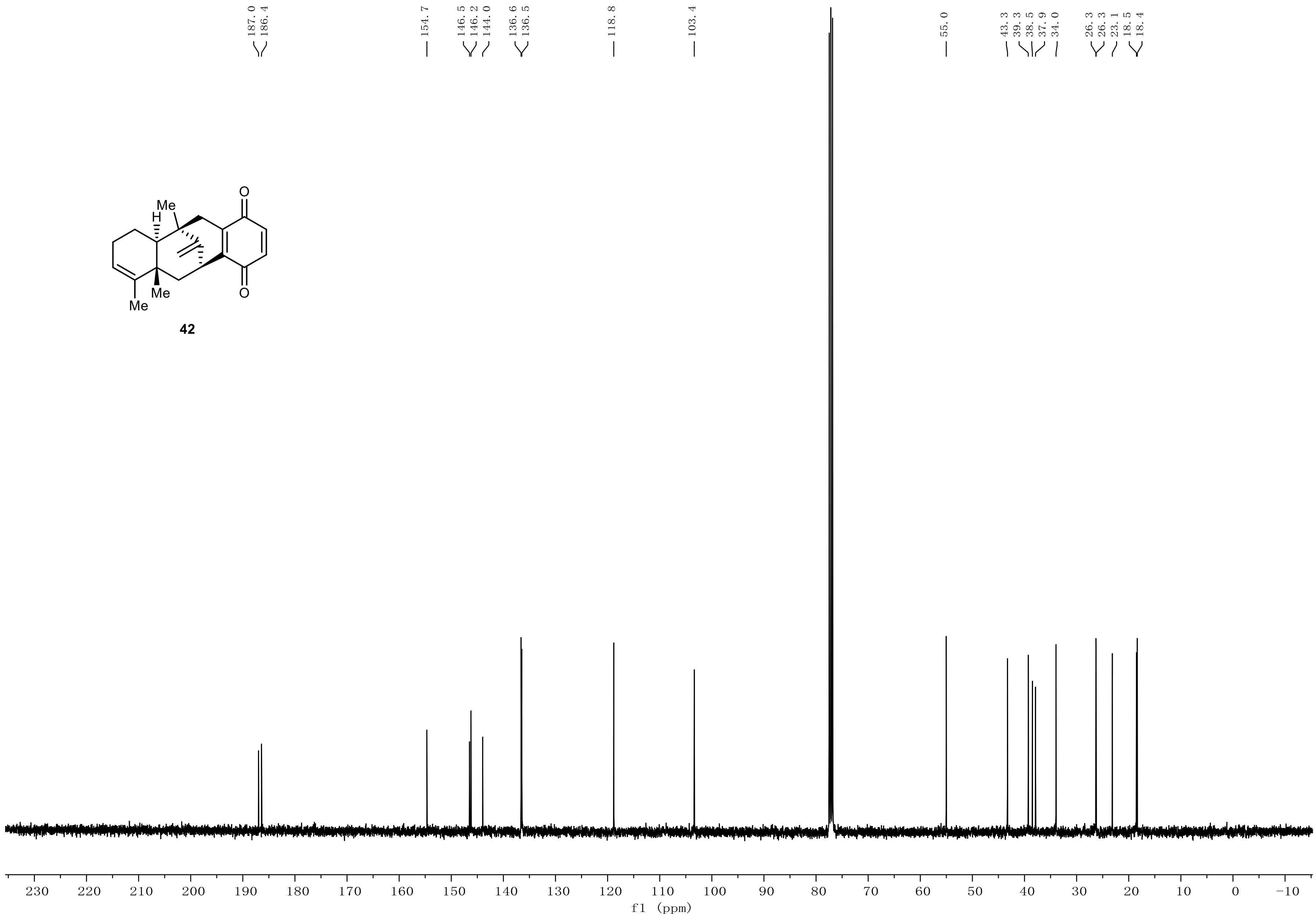

Supplementary Figure 71. ^{1}H NMR Spectrum of 43 (600 MHz, $CDCl_3$)

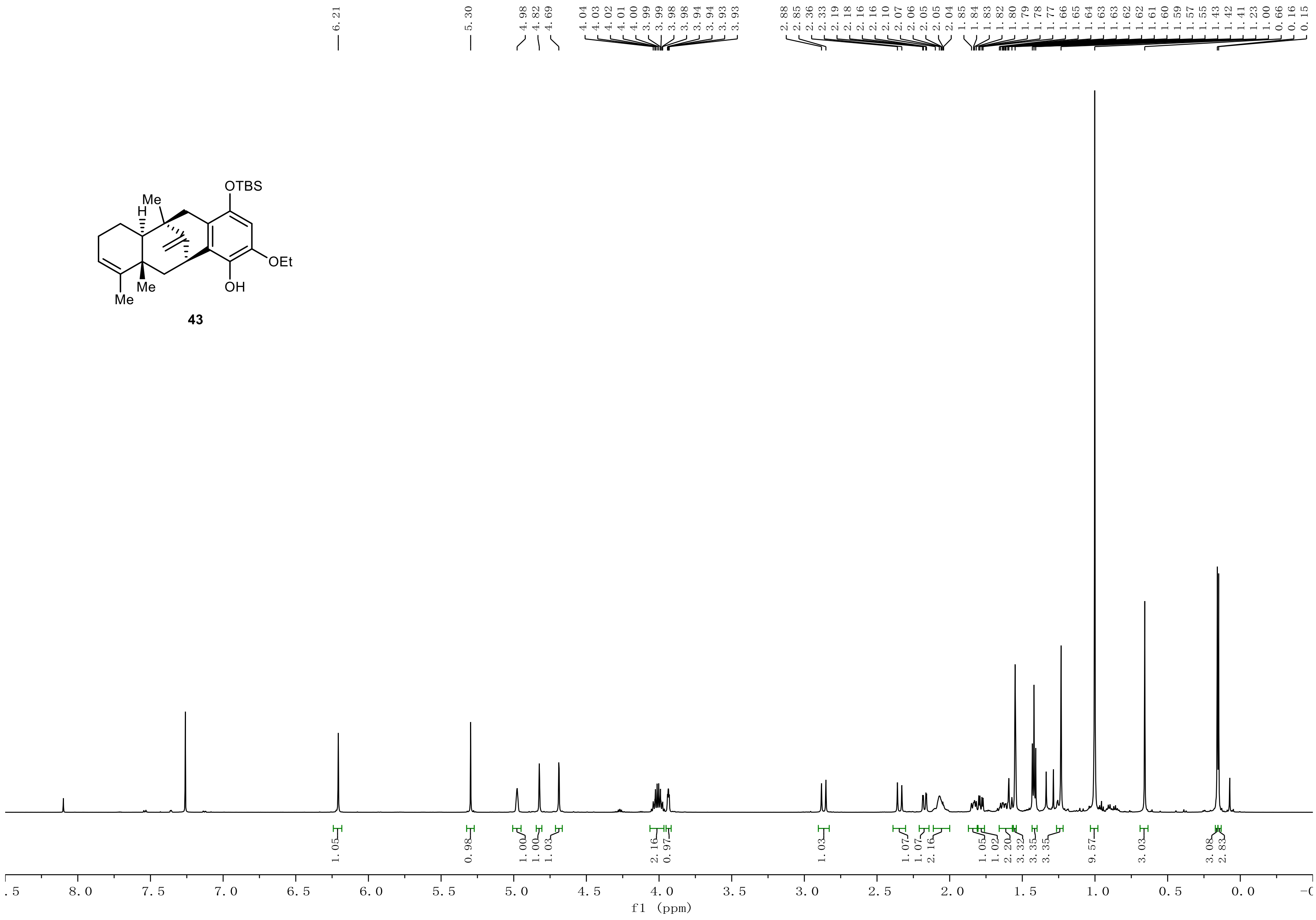

Supplementary Figure 72. ^{13}C NMR Spectrum of 43 (151 MHz, $CDCl_3$)

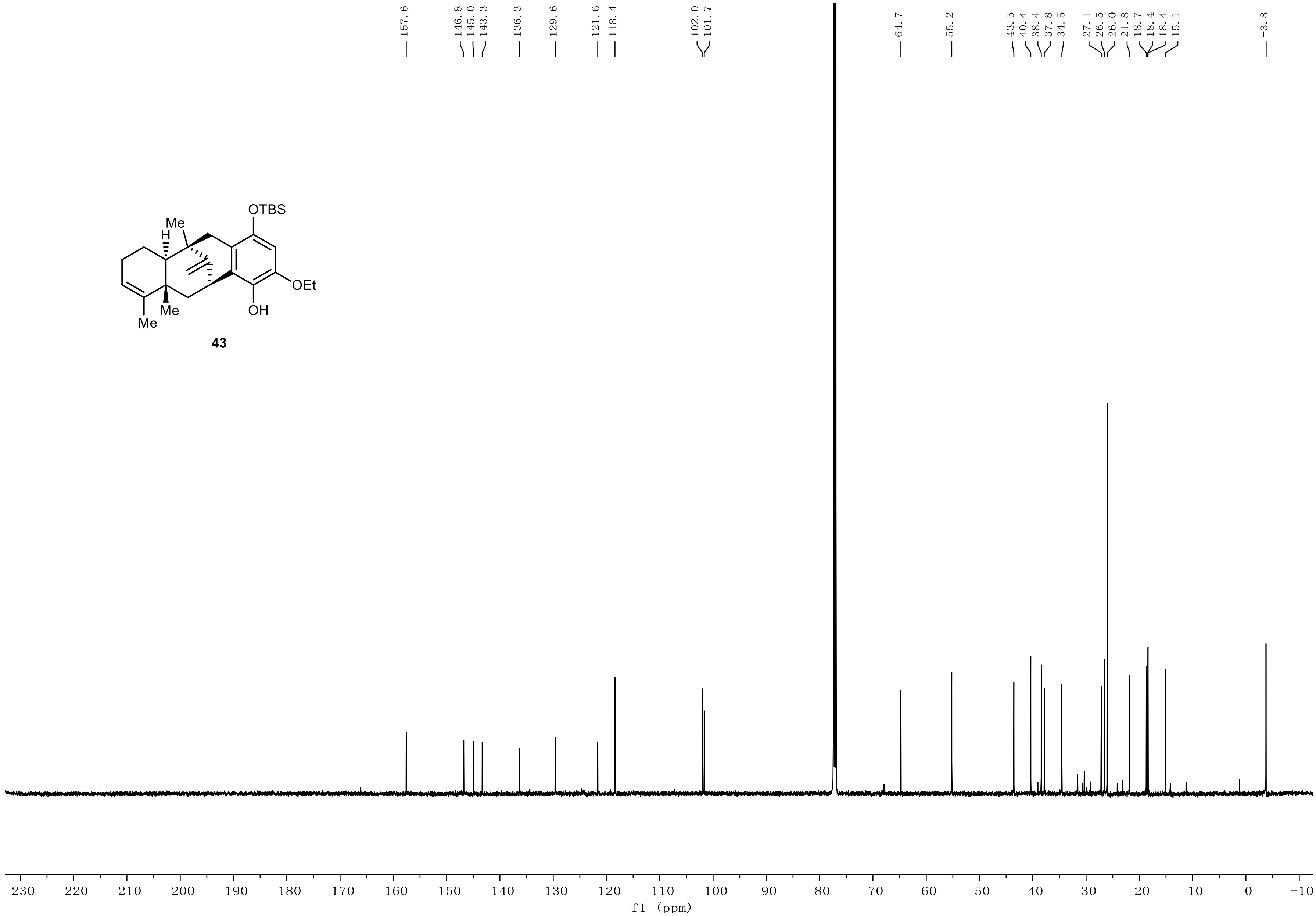

Supplementary Figure 73. COSY Spectrum of 43 ($CDCl_3$)

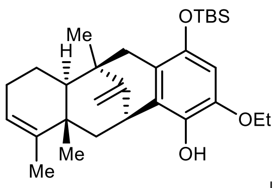

43

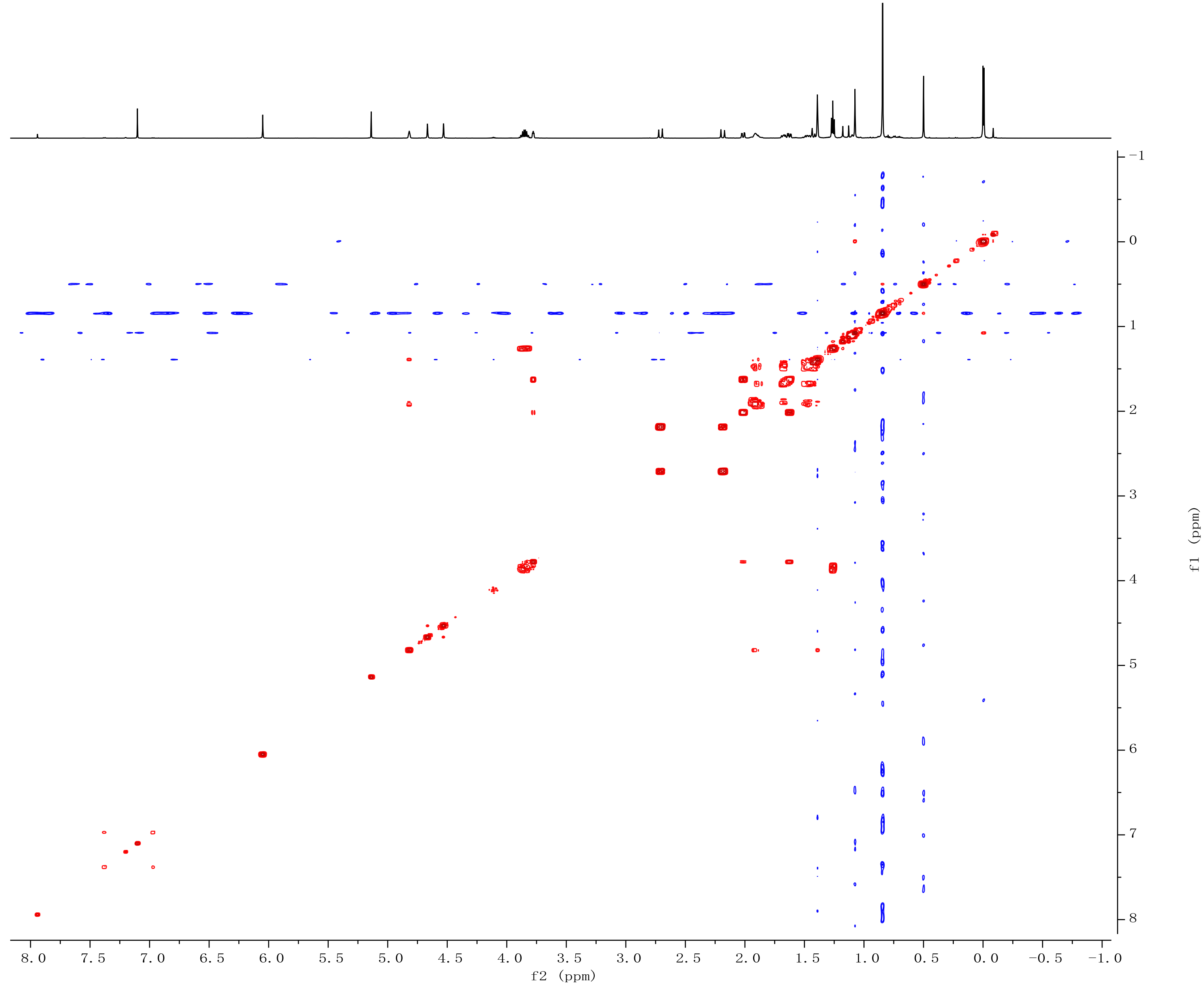

Supplementary Figure 74. NOESY Spectrum of 43 ($CDCl_3$)

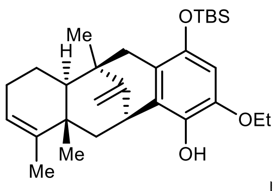

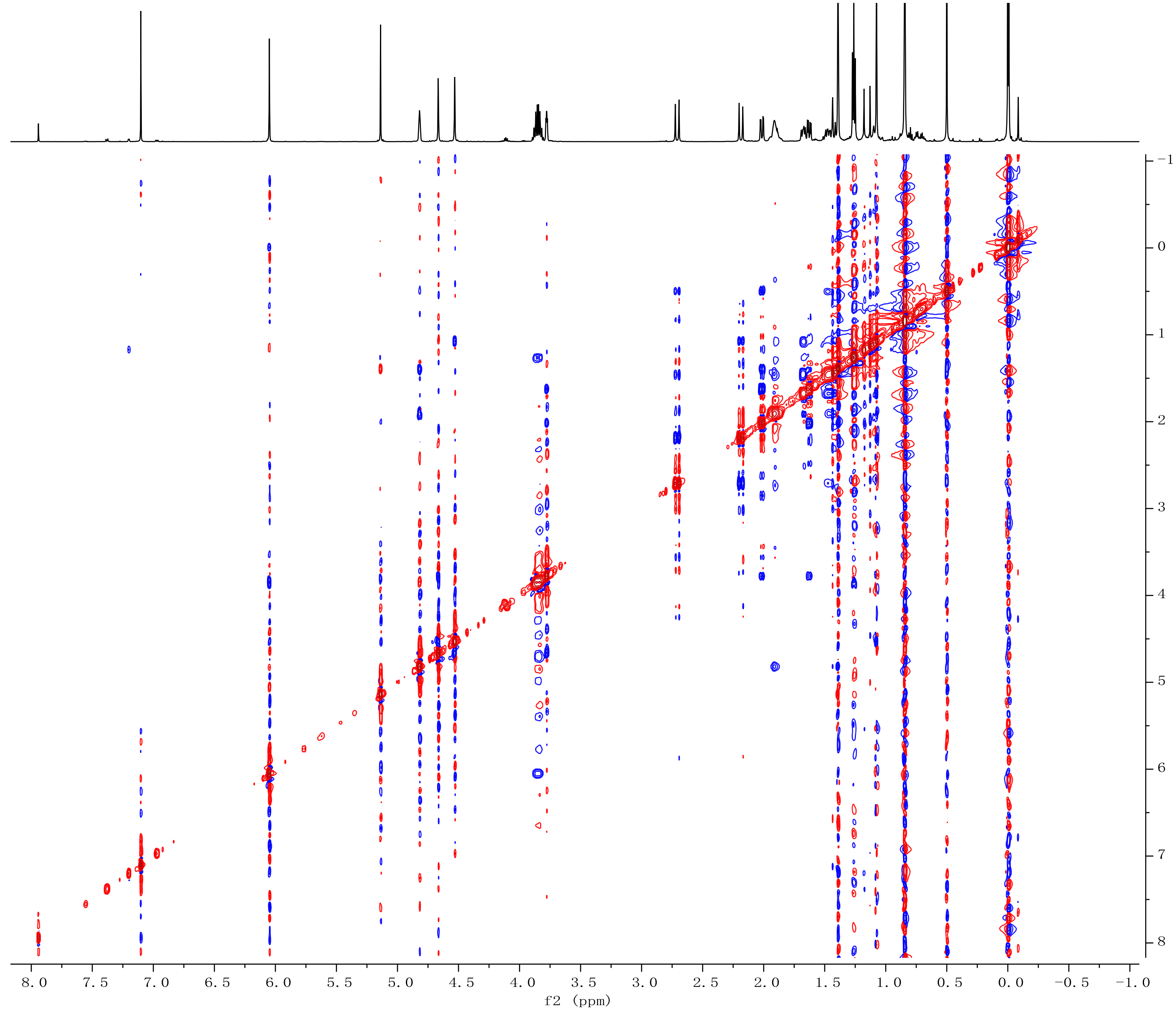

Supplementary Figure 75. HSQC Spectrum of 43 ($CDCl_3$)

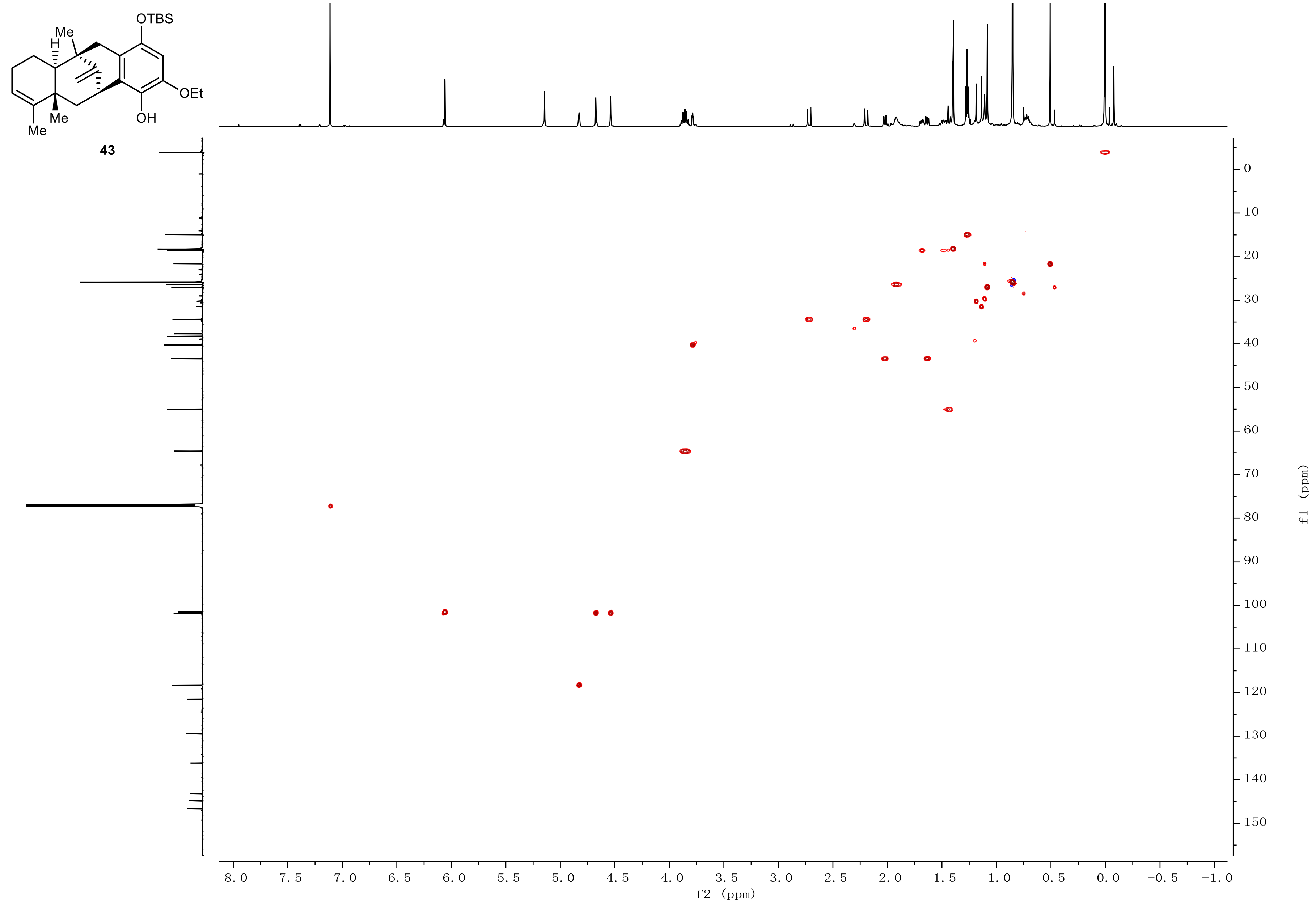

Supplementary Figure 76. HMBC Spectrum of 43 ($CDCl_3$)

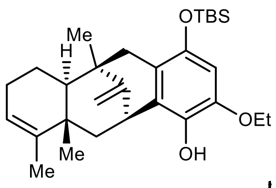

43

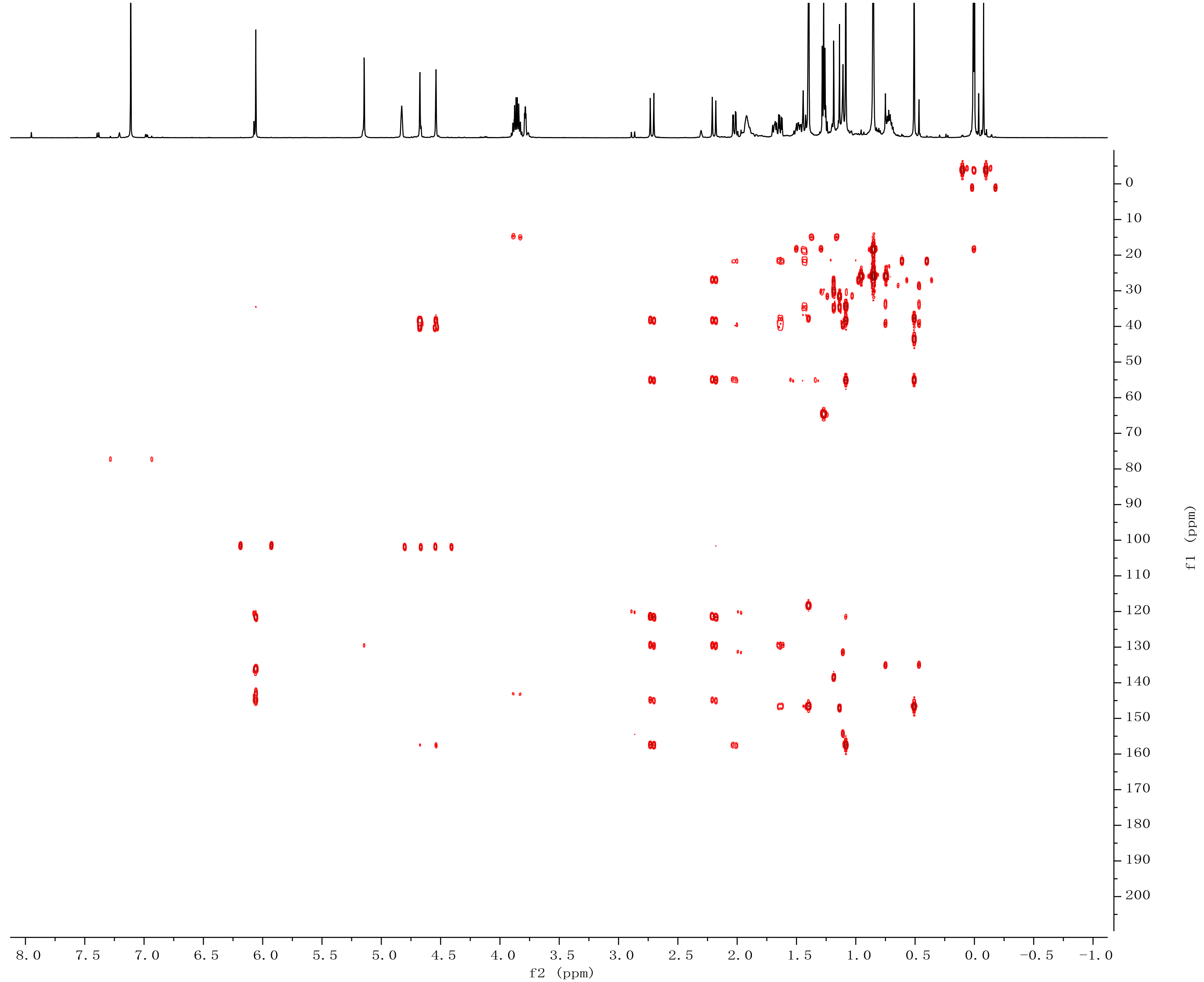

Supplementary Figure 77. ^{1}H NMR Spectrum of 44 (600 MHz, $CDCl_3$)

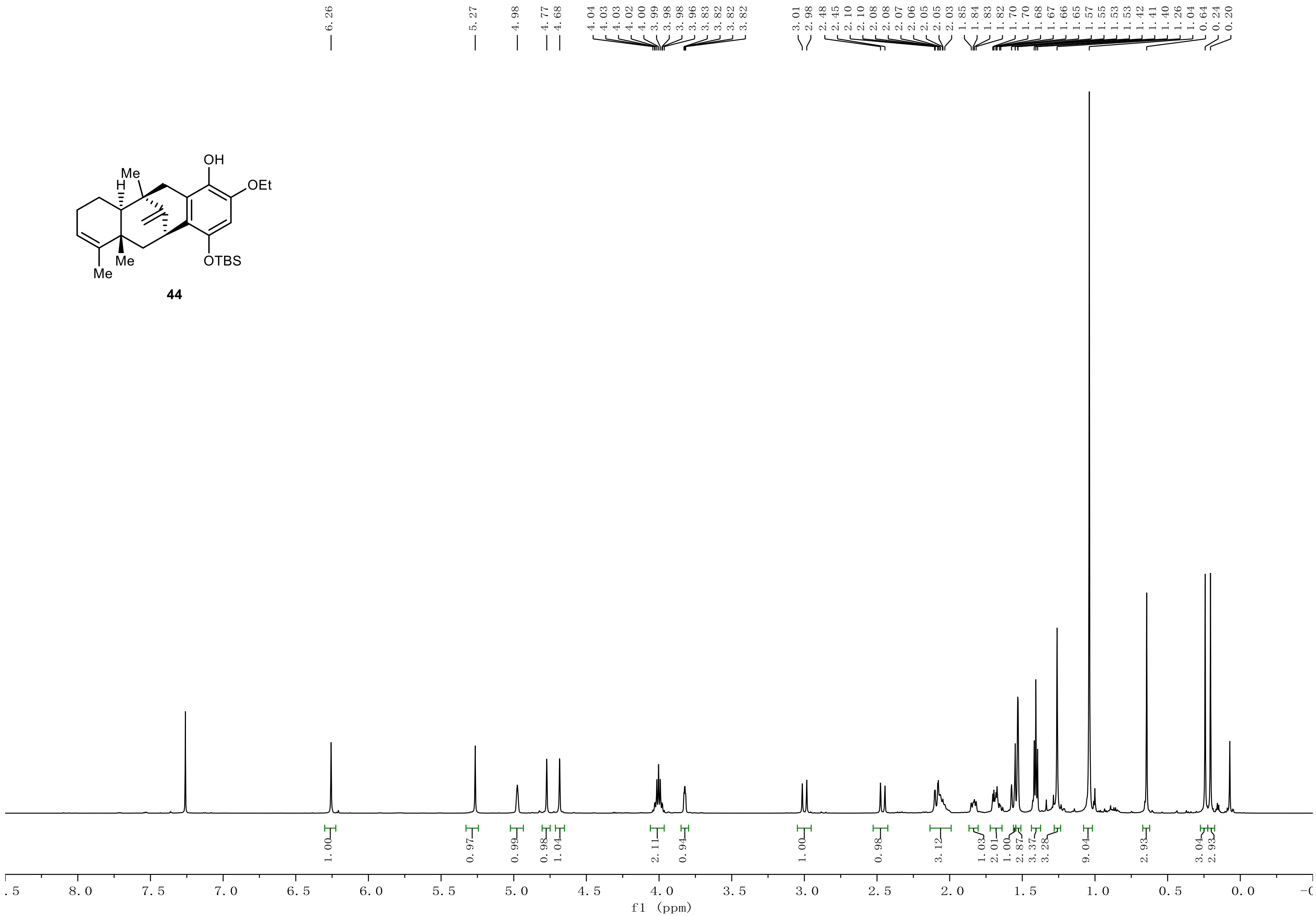

Supplementary Figure 78. ^{13}C NMR Spectrum of 44 (151 MHz, $CDCl_3$)

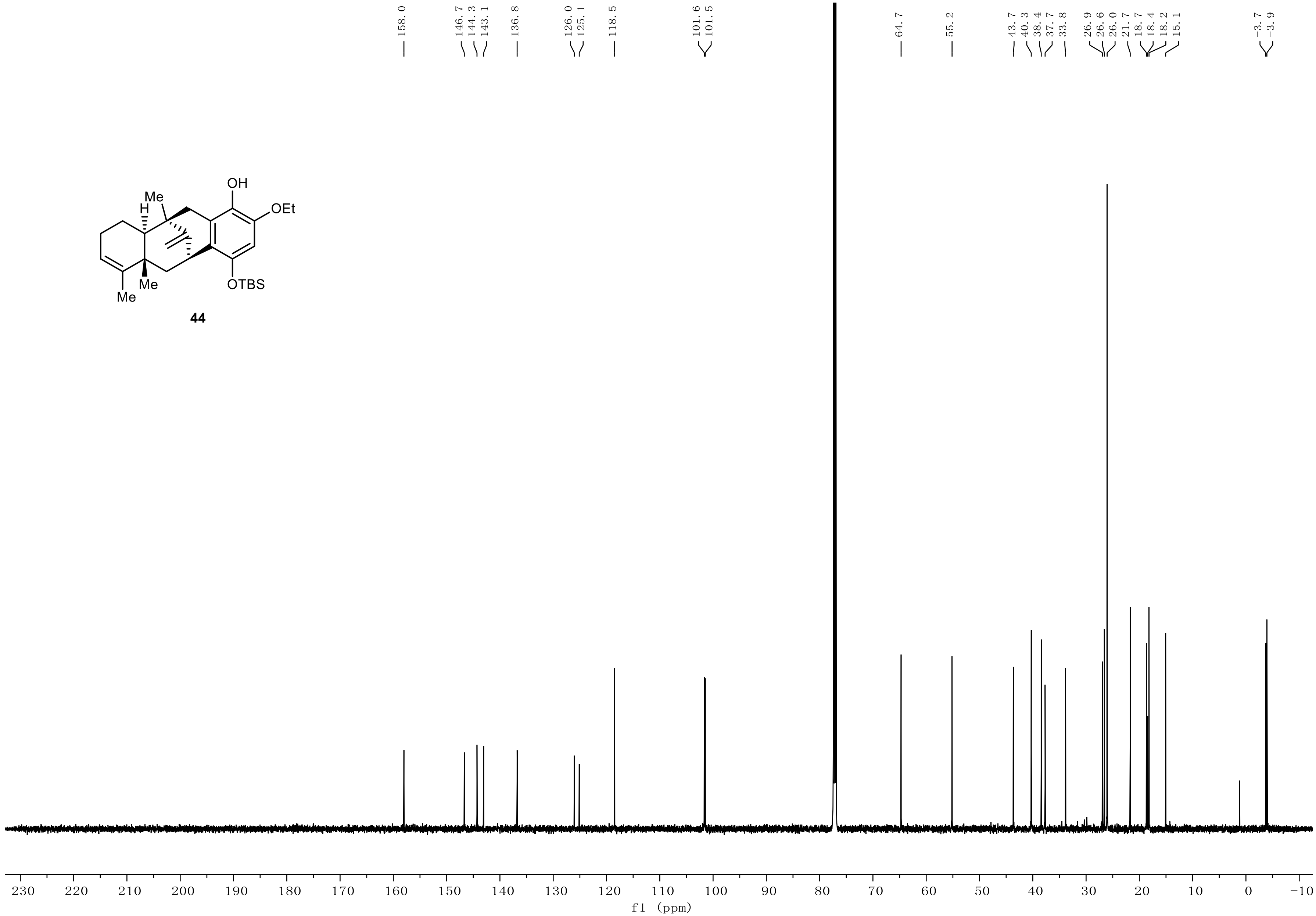

Supplementary Figure 79. COSY Spectrum of 44 ($CDCl_3$)

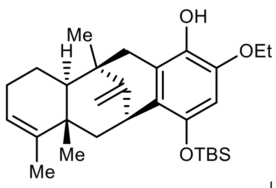

44

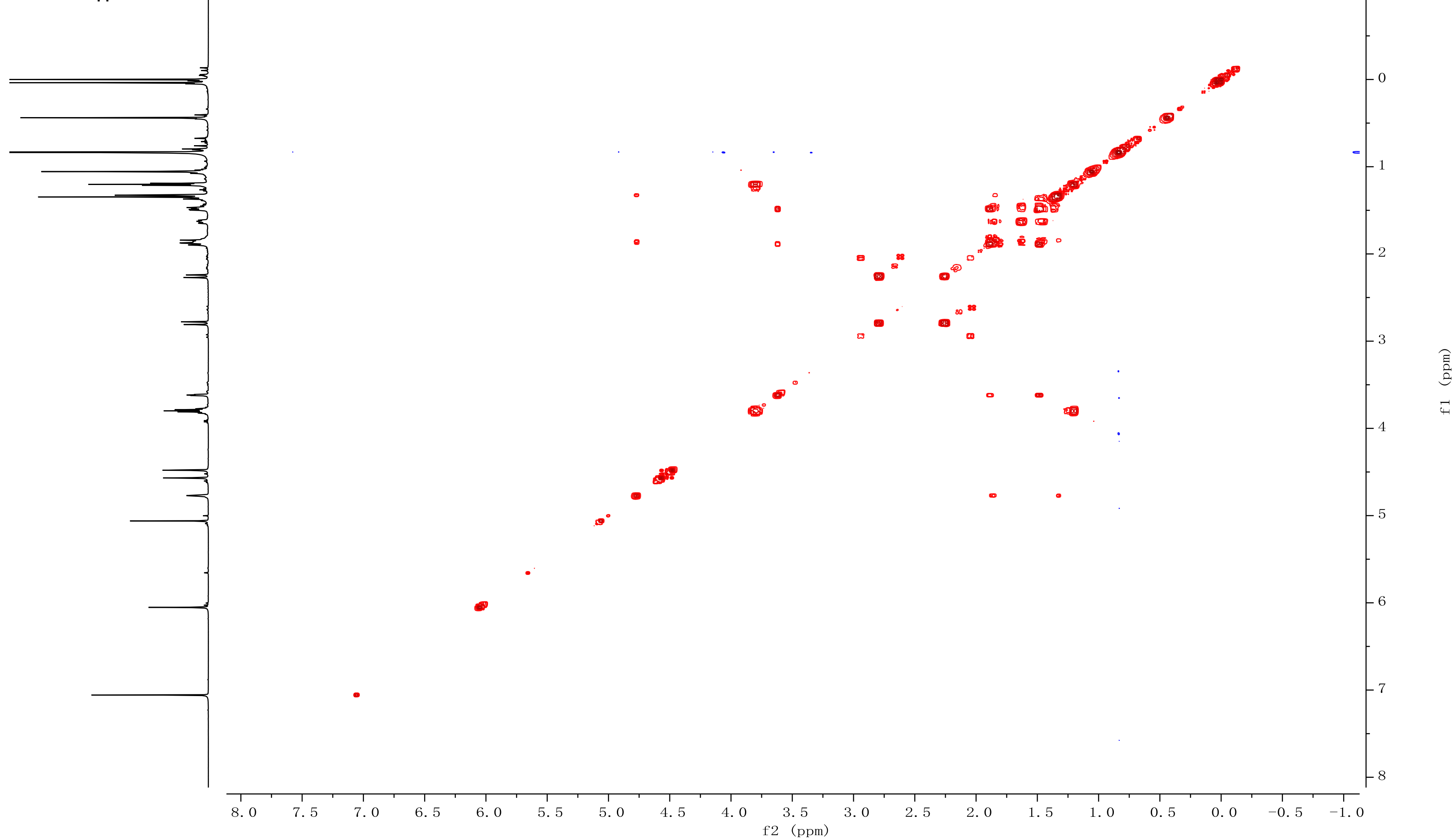

Supplementary Figure 80. NOESY Spectrum of 44 ($CDCl_3$)

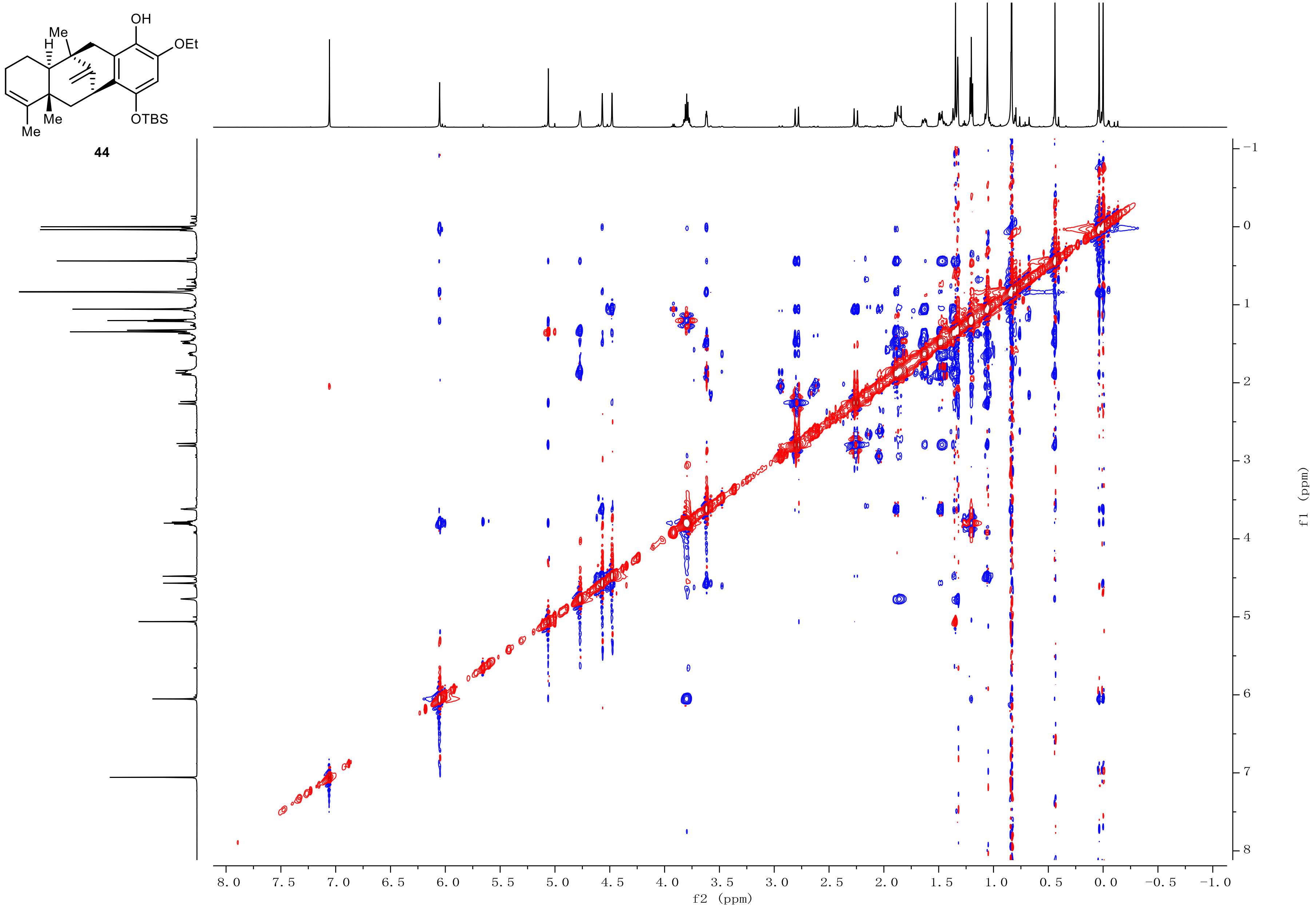

Supplementary Figure 81. HSQC Spectrum of 44 ($CDCl_3$)

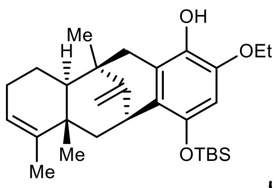

44

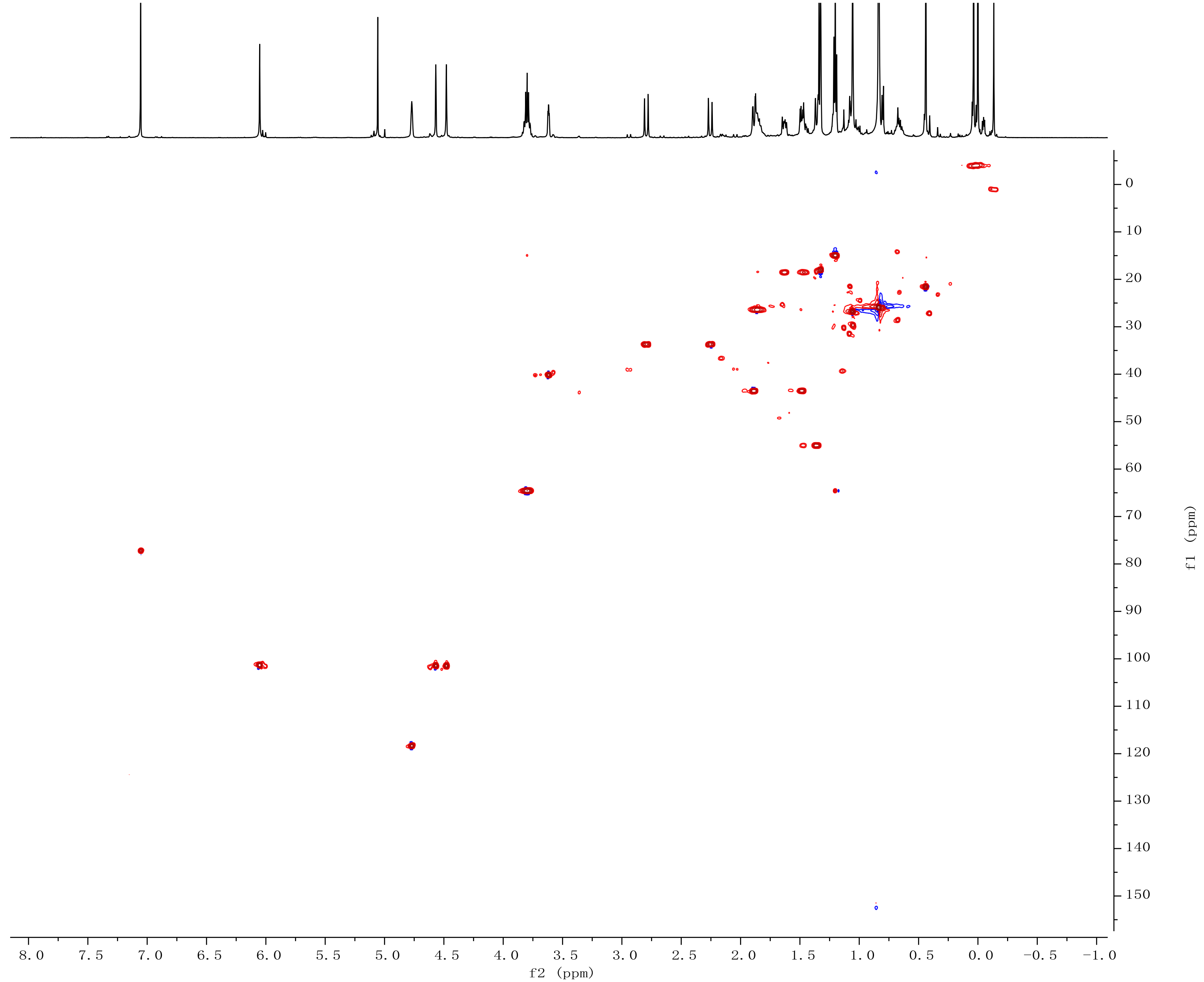

Supplementary Figure 82. HMBC Spectrum of 44 ($CDCl_3$)

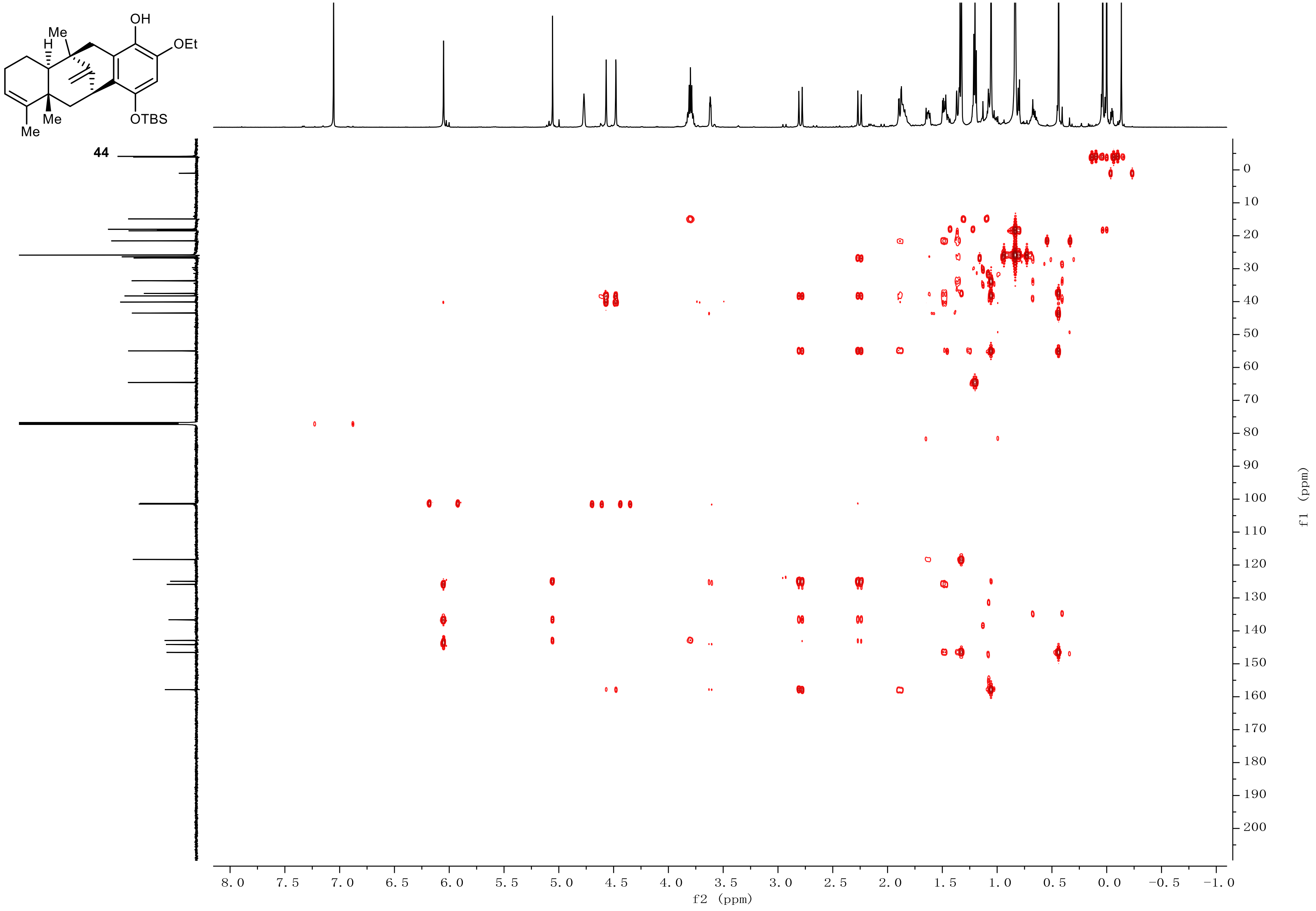

Supplementary Figure 83. ^{1}H NMR Spectrum of 45 (400 MHz, $CDCl_3$)

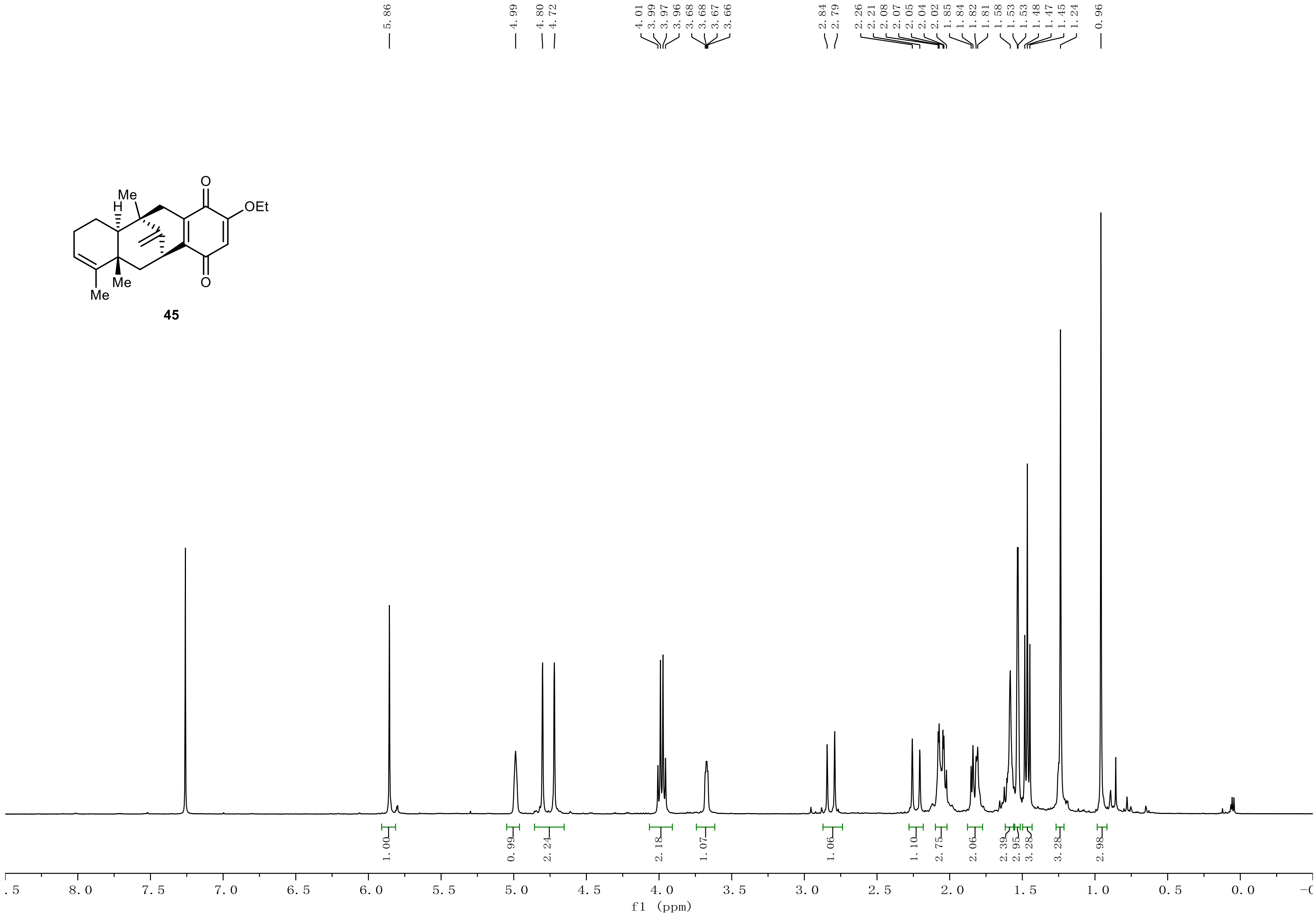

Supplementary Figure 84. ^{13}C NMR Spectrum of 45 (101 MHz, CDCl$_3$)

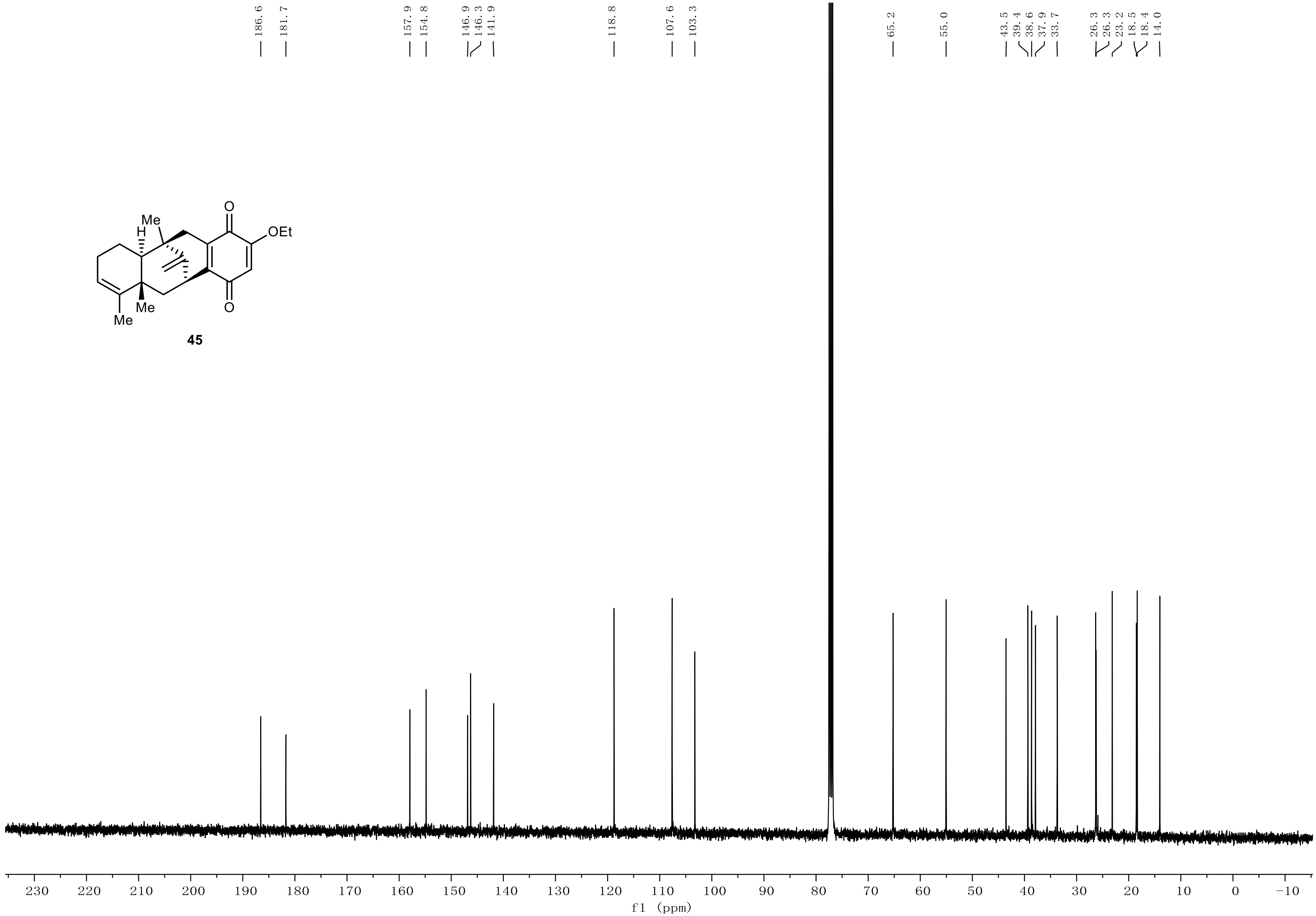

Supplementary Figure 85. ^{1}H NMR Spectrum of Proposed Structure of Dysidavarone D (10) (400 MHz, $CDCl_3$)

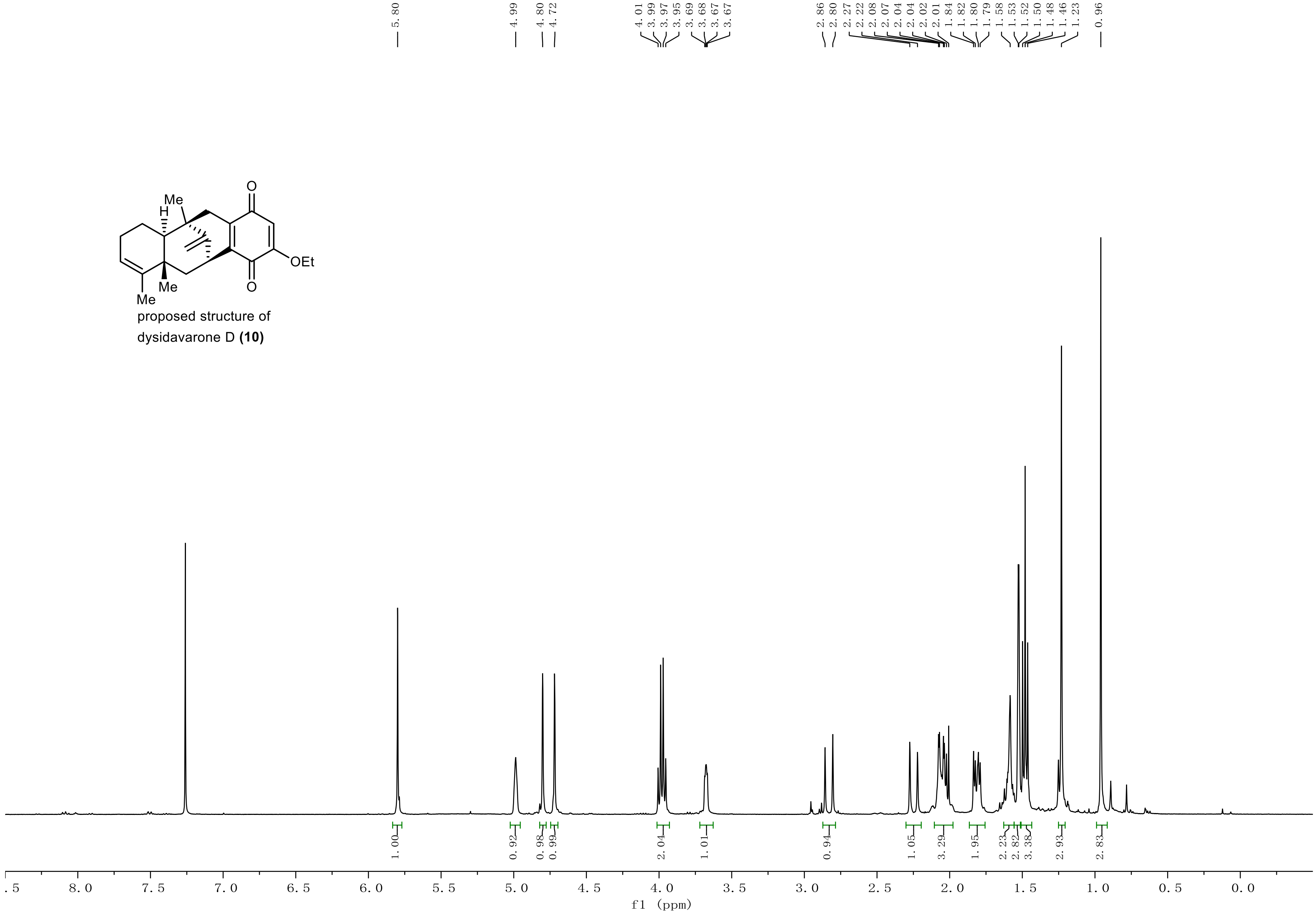

Supplementary Figure 86. ^{13}C NMR Spectrum of Proposed Structure of Dysidavarone D (10) (101 MHz, $CDCl_3$)

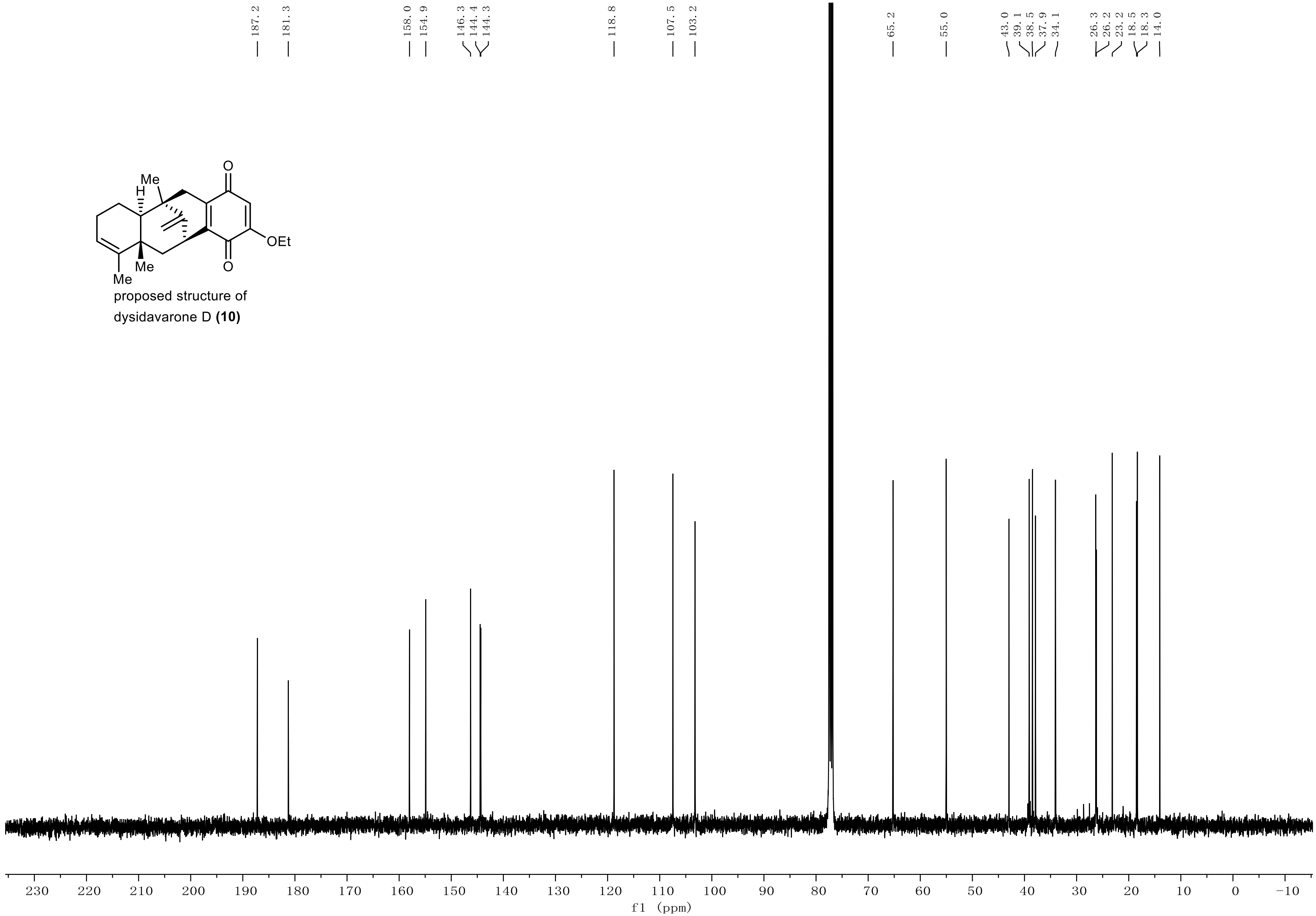

Comparison of the Spectra of Dysidavarone D and Proposed Structure of Dysidavarone D

Supplementary Table 1. Comparison of the ^{1}H NMR ($CDCl_3$) data of dysidavarone D and proposed structure of dysidavarone D.

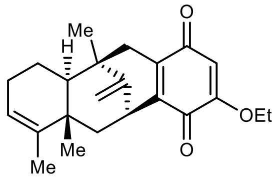

proposed structure of dysidavarone D (**10**)

**Dysidavarone D (Nature) δH [ppm, mult, J (Hz)] 500 MHz**		**Proposed structure of dysidavarone D (Synthetic) δH [ppm, mult, J (Hz)] 400 MHz**		**Err (Natural−Synthetic) ΔδH (ppm)**
5.78	1 H, s	5.80	1 H, s	−0.02
5.31	1 H, s	4.99	1 H, s	0.32
5.07	1 H, s	4.80	1 H, s	0.27
4.87	1 H, s	4.72	1 H, s	0.15
3.99	2 H, q, 7.0	3.98	2 H, q, 7.0	0.01
2.93	1 H, dt, 6.5, 1.5	3.69-3.67	1 H, m	−
2.90	1 H, d, 18.5	2.83	1 H, d, 20.7	0.07
2.42	1 H, d, 18.5	2.25	1 H, d, 20.7	0.17
2.34	1 H, dd, 6.5			
2.11	1H, m	2.08-2.01	3 H, m	−
2.08	1 H, m			
1.98	1 H, m	1.81	2 H, dd, 13.3, 4.8	−
1.72	1 H, d, 8.0			
1.52	1 H, m	1.62-1.56	2 H, m	−
1.61	3 H, d, 1.0	1.53	3 H, d, 1.4	0.08
1.47	3 H, t, 7.0	1.48	3 H, t, 7.0	−0.01
1.20	3 H, s	1.23	3 H, s	−0.03

0.95	1 H, d, 6.5	–	–	–
0.81	3 H, s	0.96	3 H, s	−0.15

Supplementary Table 2. Comparison of the ^{13}C NMR ($CDCl_3$) data of dysidavarone D and synthetic proposed structure of dysidavarone D.

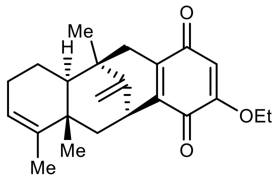

proposed structure of dysidavarone D (**10**)

**Dysidavarone D (Nature)**<br>δ ^{13}C (ppm)<br>125 MHz	**Proposed structure of dysidavarone D (Synthetic)**<br>δ ^{13}C (ppm)<br>101 MHz	**Err**<br>(Natural–Synthetic)<br>Δδ (ppm)
186.8	187.2	−0.4
180.4	181.2	−0.8
158.8	158.0	0.8
153.1	154.9	−1.8
150.4	146.3	4.1
147.0	144.4	2.6
141.0	144.3	−3.3
121.8	118.8	3.0
108.4	107.5	0.9
107.4	103.2	4.2
65.3	65.2	0.1
48.8	55.0	−6.2
45.5	43.0	2.5
45.1	39.1	6.0
42.5	38.5	4.0
41.2	37.9	3.3
38.0	34.1	3.9
26.6	26.3	0.3
25.8	26.2	−0.4

21.6	23.2	-1.6
18.5	18.5	0
17.0	18.3	−1.3
13.9	14.0	−0.1

X-ray Crystallographic Data

X-Ray Crystal Structure of **33** (CCDC2184139)

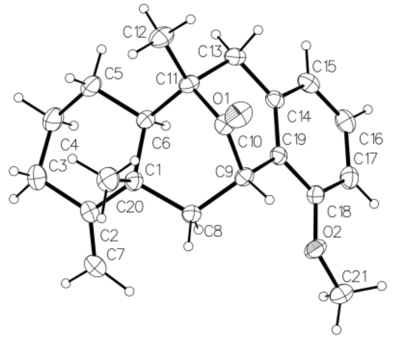

Supplementary Table 3 Crystal data and structure refinement for zhuwenming_0726_auto.

Identification code	**zhuwenming_0726_auto**
Empirical formula	$C_{21}H_{26}O_2$
Formula weight	310.42
Temperature/K	300.84(10)
Crystal system	orthorhombic
Space group	$P2_12_12_1$
a/Å	7.23772(8)
b/Å	13.77391(15)
c/Å	17.4041(2)
α/°	90
β/°	90
γ/°	90
Volume/Å^3	1735.04(3)
Z	4
ρ_{calc}g/cm^3	1.188
μ/mm^{-1}	0.579
F(000)	672.0
Crystal size/mm^3	0.18 × 0.15 × 0.12
Radiation	Cu Kα (λ = 1.54184)
2Θ range for data collection/°	8.186 to 154.898
Index ranges	$-8 \le h \le 9$, $-17 \le k \le 11$, $-19 \le l \le 22$
Reflections collected	9665
Independent reflections	3503 [R_{int} = 0.0166, R_{sigma} = 0.0166]
Data/restraints/parameters	3503/0/219
Goodness-of-fit on F^2	1.065
Final R indexes [I>=2σ (I)]	R_1 = 0.0327, wR_2 = 0.0879
Final R indexes [all data]	R_1 = 0.0335, wR_2 = 0.0883
Largest diff. peak/hole / e Å^{-3}	0.14/-0.13
Flack parameter	-0.02(6)

Supplementary Table 4 Fractional Atomic Coordinates (×10^4) and Equivalent Isotropic Displacement Parameters (Å^2×10^3) for zhuwenming_0726_auto. U_{eq} is defined as 1/3 of of the trace of the orthogonalised U_{IJ} tensor.

Atom	*x*	*y*	*z*	U(eq)
O1	9496(2)	3644.8(10)	6134.4(10)	68.7(4)
O2	7941.3(19)	6713.0(10)	7278.2(7)	54.9(3)
C1	8560(2)	5669.8(12)	4791.1(9)	43.0(3)
C2	8311(3)	6524.7(14)	4236.8(10)	53.4(4)
C3	7530(4)	6275.7(19)	3458.7(12)	75.1(7)
C4	5737(4)	5712.3(18)	3530.5(12)	71.7(6)
C5	6036(3)	4799.7(15)	4001.9(11)	57.4(5)
C6	6769(2)	5044.3(11)	4803.3(9)	39.6(3)
C7	8795(4)	7429.9(17)	4401.2(14)	68.8(6)
C8	8892(2)	6038.1(12)	5617.3(9)	43.5(4)
C9	8444(2)	5279.2(11)	6253.4(9)	38.4(3)
C10	8450(2)	4272.5(12)	5923.9(9)	43.0(4)
C11	6928(2)	4123.5(11)	5336.6(9)	41.6(3)
C12	7193(3)	3158.2(13)	4916.5(13)	58.9(5)
C13	5190(2)	4063.5(12)	5847.8(10)	44.9(4)
C14	5033(2)	4873.8(11)	6429.1(9)	38.3(3)
C15	3350(2)	5054.4(12)	6793.4(11)	47.9(4)
C16	3187(3)	5782.2(14)	7328.6(11)	53.5(4)
C17	4691(3)	6360.7(13)	7513.1(10)	50.3(4)
C18	6368(2)	6193.4(11)	7151.5(9)	40.5(3)
C19	6560(2)	5440.1(10)	6612.3(8)	35.6(3)
C20	10262(3)	5088.0(17)	4511.7(13)	62.5(5)
C21	7794(4)	7601.1(16)	7679.6(13)	72.0(7)

Supplementary Table 5 Anisotropic Displacement Parameters (Å^2×10^3) for zhuwenming_0726_auto. The Anisotropic displacement factor exponent takes the form: -$2\pi^2[h^2a^{*2}U_{11}+2hka^*b^*U_{12}+\ldots]$.

Atom	U_{11}	U_{22}	U_{33}	U_{23}	U_{13}	U_{12}
O1	70.5(9)	54.8(8)	80.9(10)	-1.9(7)	-24.2(8)	24.7(7)
O2	56.7(7)	57.2(7)	50.9(7)	-17.7(5)	-2.6(6)	-11.3(6)
C1	41.7(8)	47.9(8)	39.6(7)	-4.8(7)	3.5(6)	-2.2(7)
C2	57.9(10)	58.9(10)	43.5(9)	4.5(8)	8.0(8)	-9.3(9)
C3	102.9(19)	79.3(14)	43.0(10)	11.0(10)	-3.2(11)	-15.6(14)
C4	93.4(17)	75.8(13)	46.0(10)	5.6(9)	-24.7(10)	-12.0(12)
C5	69.5(12)	58.4(10)	44.3(9)	-5.9(8)	-14.0(9)	-8.9(9)
C6	41.6(7)	39.8(7)	37.3(7)	-3.6(6)	-4.2(6)	-0.4(6)
C7	89.0(16)	58.8(12)	58.6(12)	11.2(10)	7.2(12)	-18.3(11)

C8	40.3(8)	48.2(8)	42.1(8)	-4.2(7)	1.9(6)	-10.4(6)
C9	32.9(7)	43.7(8)	38.7(7)	-2.5(6)	-6.8(6)	0.8(6)
C10	40.2(8)	42.7(8)	46.0(8)	1.1(7)	-2.9(6)	6.8(7)
C11	45.0(8)	33.6(7)	46.2(8)	-4.5(6)	-5.5(7)	0.3(6)
C12	70.6(13)	40.0(8)	66.1(12)	-13.3(8)	-6.3(10)	5.3(8)
C13	44.7(8)	37.0(7)	53.0(9)	1.0(7)	-3.6(7)	-7.4(6)
C14	38.9(7)	35.4(7)	40.7(8)	7.6(6)	-3.3(6)	-1.5(6)
C15	38.9(8)	47.7(8)	57.1(10)	9.8(8)	2.0(7)	-3.4(7)
C16	47.3(9)	59.4(10)	53.8(10)	8.0(8)	13.2(8)	4.8(8)
C17	60.9(11)	50.2(9)	39.8(8)	-1.0(7)	7.0(8)	5.8(8)
C18	46.4(8)	41.2(7)	33.9(7)	1.9(6)	-2.7(6)	-2.2(6)
C19	36.6(7)	36.5(7)	33.8(7)	4.7(5)	-3.4(6)	1.7(6)
C20	50.3(10)	75.0(13)	62.2(12)	-11.5(10)	13.5(9)	3.4(9)
C21	97.5(17)	56.7(11)	61.9(12)	-20.4(9)	8.5(12)	-23.1(12)

Supplementary Table 6 Bond Lengths for zhuwenming_0726_auto.

Atom	Atom	Length/Å	Atom	Atom	Length/Å
O1	C10	1.206(2)	C8	C9	1.557(2)
O2	C18	1.363(2)	C9	C10	1.500(2)
O2	C21	1.413(2)	C9	C19	1.516(2)
C1	C2	1.533(2)	C10	C11	1.517(2)
C1	C6	1.557(2)	C11	C12	1.529(2)
C1	C8	1.543(2)	C11	C13	1.543(2)
C1	C20	1.548(3)	C13	C14	1.511(2)
C2	C3	1.507(3)	C14	C15	1.395(2)
C2	C7	1.326(3)	C14	C19	1.390(2)
C3	C4	1.517(4)	C15	C16	1.373(3)
C4	C5	1.517(3)	C16	C17	1.387(3)
C5	C6	1.530(2)	C17	C18	1.387(3)
C6	C11	1.576(2)	C18	C19	1.406(2)

Supplementary Table 7 Bond Angles for zhuwenming_0726_auto.

Atom	Atom	Atom	Angle/°	Atom	Atom	Atom	Angle/°
C18	O2	C21	118.15(16)	O1	C10	C11	124.31(16)
C2	C1	C6	109.61(14)	C9	C10	C11	112.35(13)
C2	C1	C8	110.62(14)	C10	C11	C6	109.94(13)
C2	C1	C20	107.07(15)	C10	C11	C12	110.41(15)
C8	C1	C6	107.38(13)	C10	C11	C13	102.17(13)

C8	C1	C20	109.82(15)	C12	C11	C6	115.29(14)
C20	C1	C6	112.37(14)	C12	C11	C13	109.36(14)
C3	C2	C1	115.78(17)	C13	C11	C6	108.86(13)
C7	C2	C1	123.73(18)	C14	C13	C11	114.09(13)
C7	C2	C3	120.5(2)	C15	C14	C13	120.13(15)
C2	C3	C4	111.29(17)	C19	C14	C13	120.54(14)
C5	C4	C3	110.3(2)	C19	C14	C15	119.33(15)
C4	C5	C6	111.10(15)	C16	C15	C14	120.87(16)
C1	C6	C11	113.12(13)	C15	C16	C17	120.61(16)
C5	C6	C1	113.48(14)	C16	C17	C18	119.11(16)
C5	C6	C11	112.64(13)	O2	C18	C17	124.80(15)
C1	C8	C9	114.16(13)	O2	C18	C19	114.39(14)
C10	C9	C8	110.37(13)	C17	C18	C19	120.81(16)
C10	C9	C19	107.18(13)	C14	C19	C9	122.59(13)
C19	C9	C8	112.47(12)	C14	C19	C18	119.25(14)
O1	C10	C9	123.24(15)	C18	C19	C9	118.16(13)

Supplementary Table 8 Torsion Angles for zhuwenming_0726_auto.

A	B	C	D	Angle/°	A	B	C	D	Angle/°
O1	C10	C11	C6	-141.68(19)	C8	C9	C19	C14	102.88(16)
O1	C10	C11	C12	-13.4(3)	C8	C9	C19	C18	-77.18(16)
O1	C10	C11	C13	102.8(2)	C9	C10	C11	C6	41.76(18)
O2	C18	C19	C9	1.15(19)	C9	C10	C11	C12	170.06(15)
O2	C18	C19	C14	-178.91(14)	C9	C10	C11	C13	-73.71(16)
C1	C2	C3	C4	53.2(3)	C10	C9	C19	C14	-18.60(19)
C1	C6	C11	C10	21.48(18)	C10	C9	C19	C18	161.33(13)
C1	C6	C11	C12	-104.08(17)	C10	C11	C13	C14	49.26(17)
C1	C6	C11	C13	132.63(14)	C11	C13	C14	C15	164.03(14)
C1	C8	C9	C10	20.38(18)	C11	C13	C14	C19	-16.6(2)
C1	C8	C9	C19	-99.26(16)	C12	C11	C13	C14	166.24(14)
C2	C1	C6	C5	47.92(19)	C13	C14	C15	C16	179.38(16)
C2	C1	C6	C11	177.85(13)	C13	C14	C19	C9	-0.5(2)
C2	C1	C8	C9	158.53(14)	C13	C14	C19	C18	179.56(13)
C2	C3	C4	C5	-56.9(3)	C14	C15	C16	C17	0.6(3)
C3	C4	C5	C6	58.4(3)	C15	C14	C19	C9	178.87(14)
C4	C5	C6	C1	-55.0(2)	C15	C14	C19	C18	-1.1(2)
C4	C5	C6	C11	174.83(18)	C15	C16	C17	C18	-0.2(3)
C5	C6	C11	C10	151.83(15)	C16	C17	C18	O2	179.59(16)
C5	C6	C11	C12	26.3(2)	C16	C17	C18	C19	-0.9(3)
C5	C6	C11	C13	-97.02(17)	C17	C18	C19	C9	-178.42(15)

C6	C1	C2	C3	-47.6(2)	C17	C18	C19	C14	1.5(2)
C6	C1	C2	C7	134.7(2)	C19	C9	C10	O1	-118.59(19)
C6	C1	C8	C9	38.96(18)	C19	C9	C10	C11	58.01(17)
C6	C11	C13	C14	-66.99(17)	C19	C14	C15	C16	0.0(2)
C7	C2	C3	C4	-129.0(2)	C20	C1	C2	C3	74.5(2)
C8	C1	C2	C3	-165.82(18)	C20	C1	C2	C7	-103.2(2)
C8	C1	C2	C7	16.5(3)	C20	C1	C6	C5	-71.0(2)
C8	C1	C6	C5	168.13(15)	C20	C1	C6	C11	58.92(19)
C8	C1	C6	C11	-61.94(17)	C20	C1	C8	C9	-83.50(17)
C8	C9	C10	O1	118.62(19)	C21	O2	C18	C17	-13.7(3)
C8	C9	C10	C11	-64.78(17)	C21	O2	C18	C19	166.78(16)

Supplementary Table 9 Hydrogen Atom Coordinates (Å×10^4) and Isotropic Displacement Parameters (Å^2×10^3) for zhuwenming_0726_auto.

Atom	*x*	*y*	*z*	U(eq)
H3A	8422.19	5890.94	3175.3	90
H3B	7308.77	6868.71	3172.56	90
H4A	4809.54	6115.44	3775.36	86
H4B	5291.93	5538.56	3023.11	86
H5A	6910.34	4380.58	3740.49	69
H5B	4876.63	4451.18	4048.79	69
H6	5822.09	5455.51	5040.15	48
H7A	9330(40)	7640(20)	4910(18)	79(8)
H7B	8610(50)	7940(20)	4027(19)	98(10)
H8A	8137.99	6610.51	5701.74	52
H8B	10175.38	6230.9	5666.34	52
H9	9387.62	5320.15	6655.71	46
H12A	8269.97	3195.46	4595.86	88
H12B	6127.96	3026.98	4604.49	88
H12C	7347.34	2646.36	5285.27	88
H13A	4104.85	4073.55	5520.91	54
H13B	5199.55	3447.92	6118.47	54
H15	2325.15	4676.97	6672.28	57
H16	2058	5887.92	7569.43	64
H17	4576.15	6854.38	7874.52	60
H20A	11331.47	5500.54	4514.58	94
H20B	10045.21	4856.72	3999.21	94
H20C	10466.16	4545.93	4847.86	94
H21A	6949.51	8021.7	7414.84	108
H21B	8986.54	7904.41	7706.58	108

H21C	7346.35	7480.67	8189.94	108

X-Ray Crystal Structure of **SI-12** (CCDC2205566)

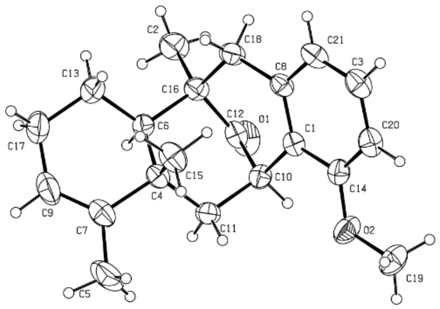

Supplementary Table 10 Crystal data and structure refinement for zhuwenm_0831_auto.

Identification code	zhuwenm_0831_auto
Empirical formula	$C_{21}H_{26}O_2$
Formula weight	310.42
Temperature/K	301.63(10)
Crystal system	orthorhombic
Space group	$P2_12_12_1$
a/Å	6.9908(2)
b/Å	9.0925(3)
c/Å	27.2122(9)
α/°	90
β/°	90
γ/°	90
Volume/Å^3	1729.71(9)
Z	4
ρ_{calc}g/cm^3	1.192
μ/mm^{-1}	0.581
F(000)	672.0
Crystal size/mm^3	0.18 × 0.16 × 0.12
Radiation	Cu Kα (λ = 1.54184)
2Θ range for data collection/°	6.496 to 154.422
Index ranges	$-4 \le h \le 8$, $-11 \le k \le 11$, $-33 \le l \le 33$
Reflections collected	6191
Independent reflections	3114 [R_{int} = 0.0861, R_{sigma} = 0.0753]
Data/restraints/parameters	3114/0/212
Goodness-of-fit on F^2	1.054
Final R indexes [I>=2σ (I)]	R_1 = 0.0815, wR_2 = 0.1962
Final R indexes [all data]	R_1 = 0.0859, wR_2 = 0.2006
Largest diff. peak/hole / e Å^{-3}	0.34/-0.42

Flack parameter 0.1(3)

Supplementary Table 11 Fractional Atomic Coordinates (×10^4) and Equivalent Isotropic Displacement Parameters (Å^2×10^3) for zhuwenm_0831_auto. U_{eq} is defined as 1/3 of the trace of the orthogonalised U_{IJ} tensor.

Atom	*x*	*y*	*z*	U(eq)
O1	-3480(5)	-5564(3)	-4142.4(12)	75.2(8)
O2	-2067(3)	-417(3)	-3962.2(13)	69.9(8)
C1	-4686(4)	-1899(4)	-4173.6(11)	45.5(6)
C2	-7350(7)	-6198(5)	-4199.9(18)	74.6(11)
C3	-6189(6)	311(4)	-4769.9(14)	63.3(9)
C4	-6440(4)	-3169(4)	-3180.1(11)	50.5(7)
C5	-5440(11)	-2684(9)	-2271.9(16)	106(2)
C6	-7192(4)	-4549(4)	-3451.8(11)	48.9(7)
C7	-6678(5)	-3479(6)	-2628.5(14)	68.7(10)
C8	-6420(4)	-2103(4)	-4420.3(10)	47.7(7)
C9	-8094(6)	-4371(7)	-2465.2(15)	84.4(14)
C10	-3835(4)	-3094(4)	-3857.9(12)	48.4(7)
C11	-4295(4)	-2970(4)	-3303.7(12)	52.8(7)
C12	-4540(5)	-4564(4)	-4027.6(12)	51.6(7)
C13	-9287(4)	-4796(6)	-3319.6(16)	72.9(12)
C14	-3735(4)	-539(4)	-4226.1(12)	51.0(7)
C15	-7568(6)	-1741(6)	-3278.5(15)	69.0(10)
C16	-6696(4)	-4694(4)	-4014.3(12)	50.0(7)
C17	-9448(6)	-5160(9)	-2778.4(18)	94.0(18)
C18	-7547(5)	-3504(4)	-4355.2(13)	56.2(8)
C19	-1005(6)	914(6)	-4016(2)	79.8(12)
C20	-4487(5)	558(4)	-4519.1(14)	59.6(8)
C21	-7132(5)	-1013(5)	-4720.1(12)	59.1(8)

Supplementary Table 12 Anisotropic Displacement Parameters (Å^2×10^3) for zhuwenm_0831_auto. The Anisotropic displacement factor exponent takes the form: $-2\pi^2[h^2a^{*2}U_{11}+2hka^*b^*U_{12}+\ldots]$.

Atom	U_{11}	U_{22}	U_{33}	U_{23}	U_{13}	U_{12}
O1	91.2(17)	51.1(16)	83.4(18)	-0.9(15)	23.5(14)	21.5(13)
O2	58.5(11)	56.8(16)	94.3(19)	13.9(16)	-6.2(12)	-14.4(11)
C1	52.2(13)	41.3(15)	43.0(13)	1.1(13)	5.0(10)	5.2(11)
C2	105(3)	49(2)	70(2)	-3(2)	-12(2)	-14(2)
C3	91(2)	42.9(17)	56.4(17)	6.9(16)	-0.7(17)	10.6(17)
C4	57.7(14)	52.6(18)	41.2(14)	-2.6(13)	2.1(11)	3.1(13)
C5	155(4)	120(5)	42.9(17)	-10(3)	-10(2)	-13(5)
C6	46.6(12)	53.0(18)	47.3(15)	6.7(14)	-0.3(10)	-3.0(11)

Supplementary Table 12 Anisotropic Displacement Parameters ($Å^2×10^3$) for zhuwenm_0831_auto. The Anisotropic displacement factor exponent takes the form: $-2\pi^2[h^2a^{*2}U_{11}+2hka^*b^*U_{12}+…]$.

Atom	U_{11}	U_{22}	U_{33}	U_{23}	U_{13}	U_{12}
C7	80(2)	80(3)	45.3(16)	-0.9(19)	3.1(15)	7(2)
C8	59.2(14)	45.5(16)	38.4(12)	-3.5(13)	-1.0(11)	2.9(12)
C9	91(2)	110(4)	52.6(19)	19(3)	18.3(17)	5(3)
C10	43.1(11)	47.1(16)	55.2(16)	6.2(14)	1.6(10)	2.7(12)
C11	56.4(14)	51.7(18)	50.2(15)	1.6(15)	-10.1(12)	-4.1(13)
C12	64.2(15)	46.1(17)	44.4(13)	3.8(14)	9.0(12)	8.8(13)
C13	49.9(15)	97(3)	72(2)	16(2)	0.1(14)	-11.5(18)
C14	55.8(13)	45.0(17)	52.1(14)	-0.6(14)	8.8(12)	-1.9(12)
C15	81(2)	70(3)	55.4(18)	-2.8(18)	9.4(16)	22(2)
C16	61.2(14)	39.9(16)	49.0(15)	-3.5(14)	-4.7(12)	-1.8(12)
C17	65.8(19)	136(5)	80(3)	34(3)	13.6(18)	-16(3)
C18	68.4(17)	51.2(19)	49.0(15)	1.7(15)	-14.0(14)	-6.7(14)
C19	75(2)	59(2)	105(3)	5(3)	2(2)	-21.4(19)
C20	79.0(18)	41.1(18)	58.6(18)	0.3(15)	10.5(15)	1.6(15)
C21	78.1(17)	54(2)	44.8(15)	-0.8(15)	-8.1(13)	10.0(16)

Supplementary Table 13 Bond Lengths for zhuwenm_0831_auto.

Atom	Atom	Length/Å	Atom	Atom	Length/Å
O1	C12	1.214(4)	C5	C7	1.488(7)
O2	C14	1.374(4)	C6	C13	1.524(4)
O2	C19	1.427(5)	C6	C16	1.575(4)
C1	C8	1.398(4)	C7	C9	1.355(7)
C1	C10	1.508(4)	C8	C18	1.508(5)
C1	C14	1.411(5)	C8	C21	1.377(5)
C2	C16	1.528(5)	C9	C17	1.462(8)
C3	C20	1.390(6)	C10	C11	1.546(4)
C3	C21	1.379(6)	C10	C12	1.497(5)
C4	C6	1.548(5)	C12	C16	1.512(4)
C4	C7	1.536(4)	C13	C17	1.514(6)
C4	C11	1.548(4)	C14	C20	1.381(5)
C4	C15	1.542(5)	C16	C18	1.545(5)

Supplementary Table 14 Bond Angles for zhuwenm_0831_auto.

Atom	Atom	Atom	Angle/˚	Atom	Atom	Atom	Angle/˚
C14	O2	C19	117.2(3)	C1	C10	C11	114.9(3)

Supplementary Table 14 Bond Angles for zhuwenm_0831_auto.

Atom	Atom	Atom	Angle/°	Atom	Atom	Atom	Angle/°
C8	C1	C10	121.3(3)	C12	C10	C1	109.7(3)
C8	C1	C14	118.4(3)	C12	C10	C11	107.3(3)
C14	C1	C10	120.2(3)	C10	C11	C4	113.9(2)
C21	C3	C20	120.1(4)	O1	C12	C10	123.2(3)
C7	C4	C6	106.3(3)	O1	C12	C16	123.8(4)
C7	C4	C11	109.8(3)	C10	C12	C16	113.0(3)
C7	C4	C15	105.6(3)	C17	C13	C6	109.5(3)
C11	C4	C6	108.6(3)	O2	C14	C1	114.7(3)
C15	C4	C6	115.2(3)	O2	C14	C20	124.5(3)
C15	C4	C11	111.1(3)	C20	C14	C1	120.8(3)
C4	C6	C16	117.2(3)	C2	C16	C6	109.3(3)
C13	C6	C4	109.4(3)	C2	C16	C18	108.2(3)
C13	C6	C16	115.4(3)	C12	C16	C2	111.1(3)
C5	C7	C4	119.0(4)	C12	C16	C6	103.7(2)
C9	C7	C4	120.6(4)	C12	C16	C18	108.4(3)
C9	C7	C5	120.1(4)	C18	C16	C6	116.1(3)
C1	C8	C18	120.6(3)	C9	C17	C13	114.3(4)
C21	C8	C1	120.2(3)	C8	C18	C16	117.4(2)
C21	C8	C18	119.3(3)	C14	C20	C3	119.5(3)
C7	C9	C17	125.1(4)	C8	C21	C3	120.9(3)

Supplementary Table 15 Torsion Angles for zhuwenm_0831_auto.

A	B	C	D	Angle/°	A	B	C	D	Angle/°
O1	C12	C16	C2	-4.2(5)	C10	C1	C14	O2	-1.6(4)
O1	C12	C16	C6	113.0(4)	C10	C1	C14	C20	179.2(3)
O1	C12	C16	C18	-123.1(4)	C10	C12	C16	C2	178.9(3)
O2	C14	C20	C3	180.0(3)	C10	C12	C16	C6	-63.8(4)
C1	C8	C18	C16	-0.8(4)	C10	C12	C16	C18	60.1(4)
C1	C8	C21	C3	-2.2(5)	C11	C4	C6	C13	177.9(3)
C1	C10	C11	C4	65.4(4)	C11	C4	C6	C16	-48.3(4)
C1	C10	C12	O1	124.8(3)	C11	C4	C7	C5	38.6(6)
C1	C10	C12	C16	-58.3(4)	C11	C4	C7	C9	-146.7(4)
C1	C14	C20	C3	-0.8(5)	C11	C10	C12	O1	-109.7(4)
C2	C16	C18	C8	-149.9(3)	C11	C10	C12	C16	67.2(3)
C4	C6	C13	C17	-65.2(5)	C12	C10	C11	C4	-56.9(4)
C4	C6	C16	C2	173.4(3)	C12	C16	C18	C8	-29.3(4)
C4	C6	C16	C12	54.8(4)	C13	C6	C16	C2	-55.5(4)
C4	C6	C16	C18	-63.9(3)	C13	C6	C16	C12	-174.1(4)

Supplementary Table 15 Torsion Angles for zhuwenm_0831_auto.

A	B	C	D	Angle/˚	A	B	C	D	Angle/˚
C4	C7	C9	C17	2.7(8)	C13	C6	C16	C18	67.2(4)
C5	C7	C9	C17	177.4(6)	C14	C1	C8	C18	-176.7(3)
C6	C4	C7	C5	155.9(5)	C14	C1	C8	C21	2.3(4)
C6	C4	C7	C9	-29.4(5)	C14	C1	C10	C11	84.2(3)
C6	C4	C11	C10	47.6(4)	C14	C1	C10	C12	-154.8(3)
C6	C13	C17	C9	36.1(7)	C15	C4	C6	C13	-56.7(4)
C6	C16	C18	C8	86.8(4)	C15	C4	C6	C16	77.1(3)
C7	C4	C6	C13	59.8(3)	C15	C4	C7	C5	-81.2(5)
C7	C4	C6	C16	-166.4(3)	C15	C4	C7	C9	93.5(5)
C7	C4	C11	C10	163.5(3)	C15	C4	C11	C10	-80.1(4)
C7	C9	C17	C13	-5.6(9)	C16	C6	C13	C17	160.1(4)
C8	C1	C10	C11	-95.8(3)	C18	C8	C21	C3	176.8(3)
C8	C1	C10	C12	25.2(4)	C19	O2	C14	C1	177.7(3)
C8	C1	C14	O2	178.5(3)	C19	O2	C14	C20	-3.1(5)
C8	C1	C14	C20	-0.8(4)	C20	C3	C21	C8	0.5(5)
C10	C1	C8	C18	3.3(4)	C21	C3	C20	C14	1.0(5)
C10	C1	C8	C21	-177.7(3)	C21	C8	C18	C16	-179.8(3)

Supplementary Table 16 Hydrogen Atom Coordinates (Å×10^4) and Isotropic Displacement Parameters (Å^2×10^3) for zhuwenm_0831_auto.

Atom	*x*	*y*	*z*	U(eq)
H2A	-8672.72	-6147.87	-4293.67	112
H2B	-7196.63	-6914.46	-3943.71	112
H2C	-6593.39	-6477.97	-4479.06	112
H3	-6693.13	1039.99	-4971.72	76
H5A	-5618.59	-1643.65	-2309.5	159
H5B	-4124.38	-2927.43	-2332.52	159
H5C	-5777.17	-2969.32	-1943.42	159
H6	-6516.55	-5371.21	-3296.58	59
H9	-8214.2	-4491.02	-2127.17	101
H10	-2442.02	-3070.19	-3897.73	58
H11A	-3882.06	-2013.14	-3187.4	63
H11B	-3564.64	-3708.79	-3127.63	63
H13A	-10019.76	-3916.2	-3391.98	88
H13B	-9801.5	-5597.58	-3513.93	88
H15A	-8876.01	-1873.56	-3180.26	103
H15B	-7518.55	-1512.53	-3622.66	103
H15C	-7012.69	-949.78	-3093.85	103

Supplementary Table 16 Hydrogen Atom Coordinates (Å×10^4) and Isotropic Displacement Parameters (Å^2×10^3) for zhuwenm_0831_auto.

**Atom**	***x***	***y***	***z***	**U(eq)**
H17A	-10736.98	-4935.11	-2669.59	113
H17B	-9250.89	-6207.88	-2735.84	113
H18A	-7732.07	-3939.06	-4677.18	67
H18B	-8801.78	-3244.33	-4230.05	67
H19A	171.35	838.7	-3835.68	120
H19B	-1742.71	1721.84	-3890.61	120
H19C	-730.36	1075.92	-4357.16	120
H20	-3859.51	1455.83	-4548.34	71
H21	-8264.81	-1171.48	-4891.44	71

III. Supplementary References

1. Oberg K. M.; Cochran B. M.; Cook M. J.; Rovis T. *Synthesis* **2018**, *50*, 4343.
2. Lee Y.; Rochette E. M.; Kim J.; Chen D. Y.-K. *Angew. Chem., Int. Ed.* **2017**, *56*, 12250.
3. Chaudhury S.; Li S.; Donaldson W. A. *Chem. Commun.***,** **2006**, *19*, 2069.
